# Multiple-component covalent organic frameworks

**DOI:** 10.1038/ncomms12325

**Published:** 2016-07-27

**Authors:** Ning Huang, Lipeng Zhai, Damien E. Coupry, Matthew A. Addicoat, Keiko Okushita, Katsuyuki Nishimura, Thomas Heine, Donglin Jiang

**Affiliations:** 1 Field of Energy and Environment, School of Materials Science, Japan Advanced Institute of Science and Technology, 1-1 Asahidai, Nomi 923-1292, Japan; 2 Scientific Computing and Modelling NV, Vrije Universiteit, Theoretical Chemistry De Boelelaan 1083, 1081 HV Amsterdam, The Netherlands; 3 Wilhelm-Ostwald-Institut für Physikalische und Theoretische Chemie, Universität Leipzig, Linnéstrasse 2, 04103 Leipzig, Germany; 4 Department of Materials Molecular Science, Institute for Molecular Science, National Institutes of Natural Sciences, 38 Nishigo-Naka, Myodaiji, Okazaki 444-8585, Japan

## Abstract

Covalent organic frameworks are a class of crystalline porous polymers that integrate molecular building blocks into periodic structures and are usually synthesized using two-component [1+1] condensation systems comprised of one knot and one linker. Here we report a general strategy based on multiple-component [1+2] and [1+3] condensation systems that enable the use of one knot and two or three linker units for the synthesis of hexagonal and tetragonal multiple-component covalent organic frameworks. Unlike two-component systems, multiple-component covalent organic frameworks feature asymmetric tiling of organic units into anisotropic skeletons and unusually shaped pores. This strategy not only expands the structural complexity of skeletons and pores but also greatly enhances their structural diversity. This synthetic platform is also widely applicable to multiple-component electron donor–acceptor systems, which lead to electronic properties that are not simply linear summations of those of the conventional [1+1] counterparts.

## Abstract (web summary)

Covalent organic frameworks are crystalline porous polymers integrating molecular building blocks into periodic structures. Here, the authors report a general multiple-component condensation strategy that enables the use of one knot and two or three linkers to synthesize complex, anisotropic frameworks.

## Graphical abstract

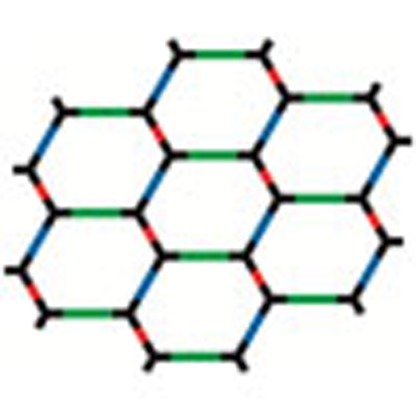

Covalent organic frameworks (COFs) are a class of crystalline polymers in which organic building blocks are topologically linked into extended lattice structures with periodic skeletons and ordered pores^1^,^2^,^3^,^4^,^5^. In contrast with other crystalline porous materials, a distinct feature of COFs is that they allow precise design of and control over both skeletons and pores^6^,^7^,^8^,^9^. Remarkably, two-dimensional (2D) COFs can integrate building blocks into 2D polymer sheets and further layered frameworks, which constitute periodic columnar *π*-arrays and orientated one-dimensional (1D) channels^1^,^2^,^3^,^4^,^5^,^10^. By virtue of their ordered *π*-structure and high porosity, COFs have emerged as a powerful platform for designing functional materials and have shown outstanding performance in various fields, including gas adsorption^11^,^12^,^13^,^14^,^15^,^16^,^17^,^18^,^19^, light emitters^20^,^21^,^22^,^23^, catalysis^24^,^25^,^26^,^27^,^28^,^29^, semiconductors^30^,^31^,^32^,^33^,^34^, proton conductions^35^,^36^,^37^ and energy conversion and storage^38^,^39^,^40^,^41^.

COFs are typically synthesized via topologically directed [1+1] condensation reactions between a knot component and another linker unit. As a result, for example, only a maximum of 10 different COFs can be synthesized from a library of one knot and 10 linkers. Under this conventional [1+1] design scheme, development of new COFs is largely dependent on the exploration of new knot and linkers, which is however, tedious and unproductive. Here we report a general multiple-component (MC) strategy that allows for the use of more than two components for the topological design and practical synthesis of MC-COFs, which are formed in a single phase with permanent porosity and high crystallinity, irrespective of their components and topologies. Notably, this MC strategy is exceptionally effective at increasing the structural diversity of COFs, and a collection of one C_3_-symmetric vertex and 10 C_2_-symmetric linkers can generate 210 new hexagonal MC-COFs according to the law of combinatorics^42^. To demonstrate the effectiveness of various combinations, 53 MC-COFs were synthesized by condensing one knot with two or three linkers to produce hexagonal MC-COFs and two linkers to prepare tetragonal MC-COFs. Furthermore, unlike conventional [1+1] COFs, which undergo symmetric tiling and produce regular polygon pores, MC-COFs considerably enhanced complexity in both skeletons and pores by creating sequenced anisotropic tiling and unusually shaped yet ordered pores. Interestingly, this MC strategy is also applicable to the synthesis of multiple-component electron donor–acceptor COFs in which sequenced donor and acceptor *π*-arrays trigger strong electronic correlations among the latticed *π*-components. As a result, MC-COFs exhibit greatly enhanced electronic properties that are not simple linear summation of these of the conventional [1+1] two-component COFs. Therefore, the multiple-component COFs provide a new platform that considerably expands the designability of structures and functions of porous organic materials.

## Results

### Design principle of MC-COFs

Recently, COFs with complicated lattices and porous structures have been developed by using several different approaches (Supplementary Fig. 1). The development of the C_2_+C_2_ topology diagram using one knot and two linkers enables the synthesis of imine-linked kagome-type COFs with triple pores^43^. Two examples of such COFs were demonstrated although their crystallinity and porosity are quite low; SIOC-COF-1 has the surface area of 478 m^2^ g^–1^ with the total pore volume of 0.30 cm^3^ g^–1^ and SIOC-COF-2 has much low surface area of 46 m^2^ g^–1^ and decreased pore volume of only 0.09 cm^3^ g^–1^. This condensation reaction is interesting for creating triple pores, but the low porosity and limited crystallinity suggest that the tetraphenylethene knot-based reaction systems are likely very sensitive to the length of the linkers while the reason for the extremely low porosity remains unclear. On the other hand, the use of a bifunctional linker of 4-formylphenyl boronic acid allows for the synthesis of COFs with two different boronate and imine linkages in the skeletons that are not available for conventional [1+1] based COFs^6^,^7^. The exploration of desymmetric knot for the [1+1] condensation reaction leads to the synthesis of COFs that consist of co-existed two different crystalline structures^44^. The desymmetric strategy thus focuses on the exploration of [1+1] combination, while the desymmetric knot is the key building block. The desymmetric approach was exemplified for the hexagonal dual-pore COFs, but it did not show its capability of synthesising tetragonal COFs. These approaches are interesting as specific cases of complicated COFs and demonstrate that COFs are capable of complicated structural formation. Herein, we report the multiple-component (MC) [1+2] and [1+3] strategies based on the general topology diagrams of the C_3_+C_2_ and C_4_+C_2_ schemes for the synthesis of hexagonal and tetragonal COFs. We highlight that these MC-COFs cannot be predicted and synthesized by using the above three approaches (Supplementary Fig. 1).

**Figure 1:**
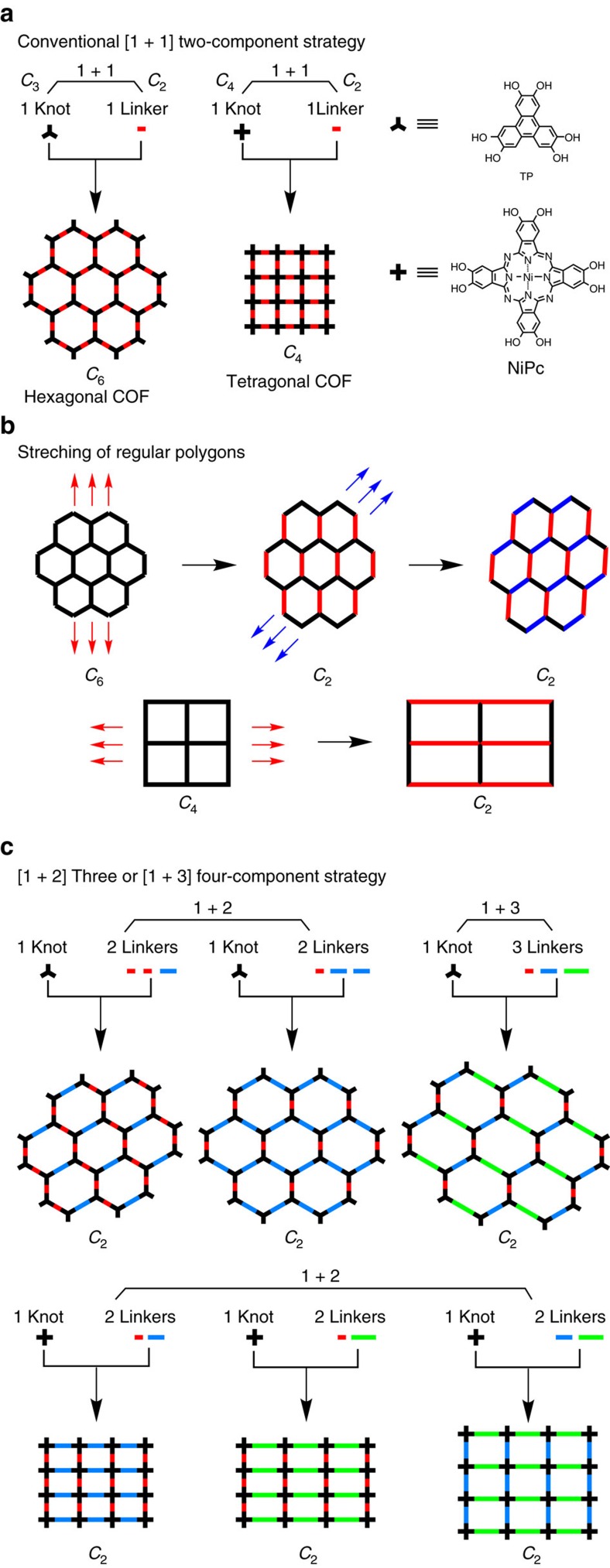
Topology diagrams for the design of COFs. (**a**) Conventional [1+1] two-component diagram with one knot and one linker for the synthesis of hexagonal and tetragonal COFs. The knots are shown as C_3_ and C_4_-symmetric bars, and the linkers are shown as C_2_-symmetric bars. TP and NiPc are typical C_3_ and C_4_-symmetric knots. (**b**) Polygon geometric transformation on stretching of regular C_6_-symmetric hexagons and C_4_-symmetric tetragons into their corresponding C_2_-symmetric polygons. (**c**) Our multiple-component [1+3] or [1+4] strategy for the synthesis of hexagonal and tetragonal multiple-component COFs (MC-COFs). Three linkers shown in different colours and with different lengths are used to illustrate the typical knot-linker combinations in the MC strategy. The linkers are asymmetrically tiled to generate sequenced networks and specially shaped pores that are completely unlike from those of conventional COFs.

Our idea is based on the following polygon geometric transformation mechanism: The regular hexagon (C_6_) and tetragon (C_4_) have three and two pairs of same-sized parallel sides, respectively. From the perspective of polygon geometry, stretching or shrinking along one pair of parallel sides can produce C_2_-symmetric hexagons and tetragons while retaining original 120° and 90° angles (Fig. 1b). This geometric transformation makes it possible to develop the C_2_-symmetric hexagonal and tetragonal pores through the topology design of COFs by developing multiple-component reaction systems. On the basis of the above idea, our concept is to integrate two or three different linkers into the frameworks while keeping one-knot structure. Owing to the high reversibility of the boronate-linkage reaction, the 2D polygons are capable of quick structural self-healing. The disordered 2D polygon layers if any formed are difficult to induce effective *π*–*π* interactions that are essential for the formation of layered crystalline frameworks; such unstable disordered polygons would decompose and finally leaves ordered 2D layers in which linker units are statistically balanced and are integrated into an ordered lattice. For the synthetic reactions, we utilized the conventional solvothermal conditions that are similar to those for the synthesis of [1+1] boronate-linked COFs. The 10 different linkers were selected as they have similar solubility and reactivity under the solvothermal conditions.

**Figure 2:**
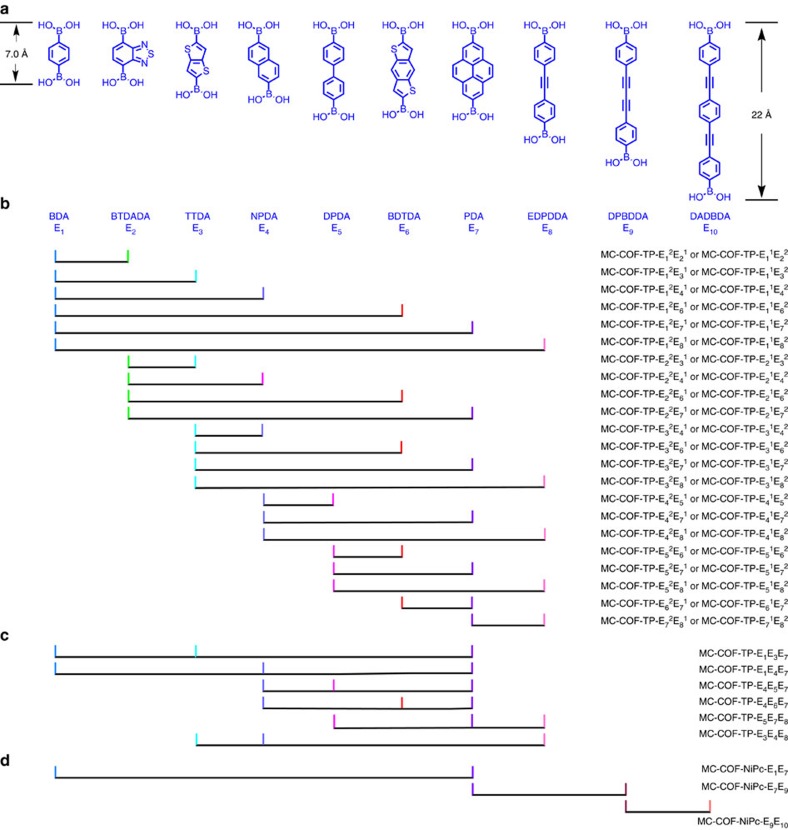
Schematic of linker combinations for MC-COFs. (**a**) 10 linkers from E_1_ to E_10._ These linkers vary in length and consist of simple phenyl, heterocycles and large *π*-units with different electron-rich and deficient structures. These linkers were chosen to demonstrate the scope of the possible combinations. (**b**) Experimental [1+2] combinations for the synthesis of 44 three-component hexagonal MC-COFs with TP knots. (**c**) Experimental [1+3] combinations for the synthesis of six four-component hexagonal MC-COFs with TP knots. (**d**) Experimental [1+2] combinations for the synthesis of three-component tetragonal MC-COFs with NiPc knots.

Figure 1a shows the conventional hexagonal and tetragonal topologies of covalent organic frameworks (COFs) and their design schemes based on the two-component [1+1] copolymerisation of a C_3_ or C_4_-symmetric knot and a C_2_-symmetric linker^1^,^2^. The C_3_-symmetric triphenylene (TP) and C_4_-symmetric nickel phthalocyanine (NiPc) units are representative knots used for the synthesis of hexagonal and tetragonal COFs^1^,^2^,^45^,^46^, respectively. The crystalline ordered structures of COFs are error-checked and repaired via self-healing through reversible covalent bonding reactions^1^,^2^,^47^,^48^. Figure 1b shows the multiple-component (MC) [1+2] or [1+3] strategy that we developed for the synthesis of MC-COFs, using one knot and three different linkers to showcase their diversities and transitions in their network lattice and pore size and shape. In the hexagonal topology (Fig. 1c), each TP knot was connected to three linker units arranged with intervals of 120°. For the three-component systems, this geometry required the stoichiometric ratio of the two linkers to be 1:2 or 2:1 for the formation of closed hexagons and extended lattice structures. We developed two different [1+2] three-component copolymerisation systems in which the molar ratio of the two linker units was 1:2 or 2:1 (Fig. 1c). We further varied all three linkers and explored [1+3] copolymerisation systems to achieve four-component hexagonal MC-COFs. We applied this synthetic strategy to create tetragonal MC-COFs in which each knot was connected to two sets of the linker units (Fig. 1c). To demonstrate the rational design of MC-COFs, we synthesized 10 different linkers (Fig. 2a) for [1+2] or [1+3] copolymerization with TP and NiPc (Fig. 1c). Notably, these hexagonal and tetragonal MC-COFs (Fig. 2b–d) were obtained as crystalline porous materials in a single phase that featured the asymmetric tiling of organic units and specifically shaped polygon channels. Their lattice and porous structures were distinct from those of the conventional [1+1] two-component COFs (Fig. 1a) and were characterized using various analytical methods (Supplementary Figs 2–193; Supplementary Tables 1–18).

### Three-component [1+2] MC-COFs

**Figure 3:**
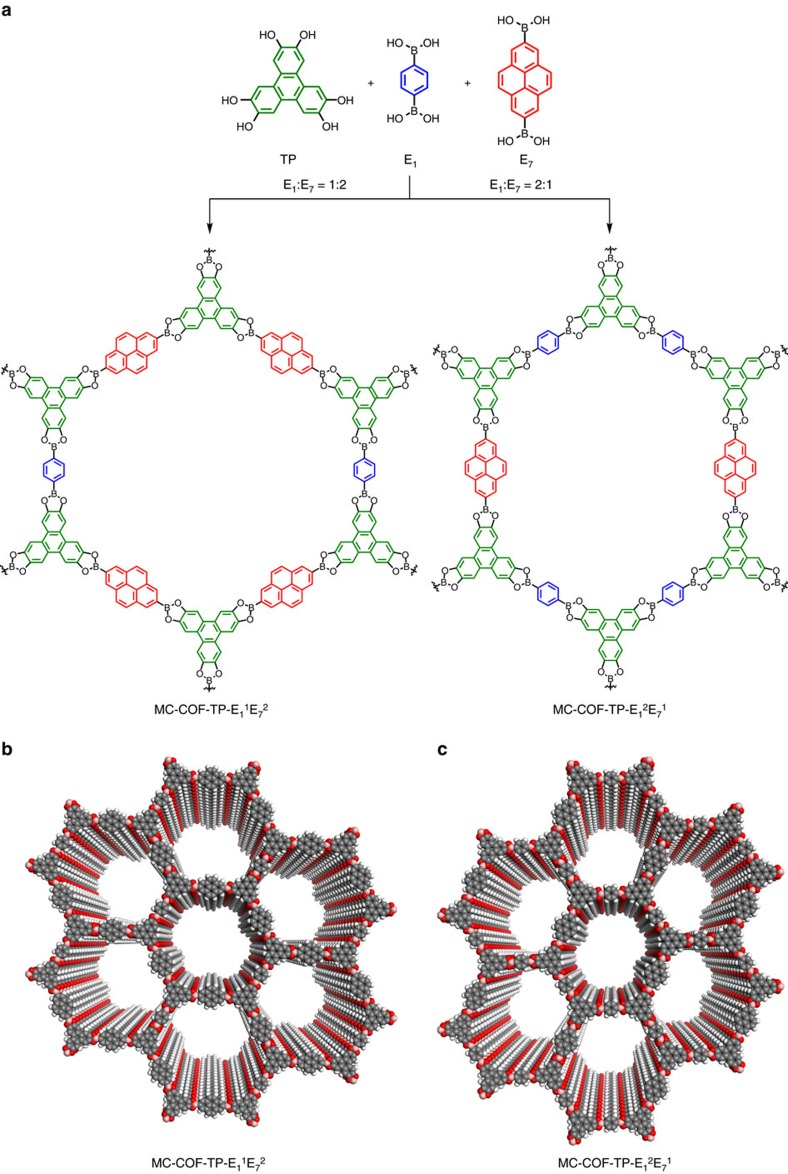
Synthesis and structures of typical [1+2] hexagonal MC-COFs. (**a**) Schematic representation of the synthesis of MC-COF-TP-E_1_^1^E_7_^2^ and MC-COF-TP-E_1_^2^E_7_^1^. (**b**) View of the slipped AA stacking crystal structure of MC-COF-TP-E_1_^1^E_7_^2^. (**c**) View of the slipped AA stacking crystal structure of MC-COF-TP-E_1_^2^E_7_^1^.

**Figure 4:**
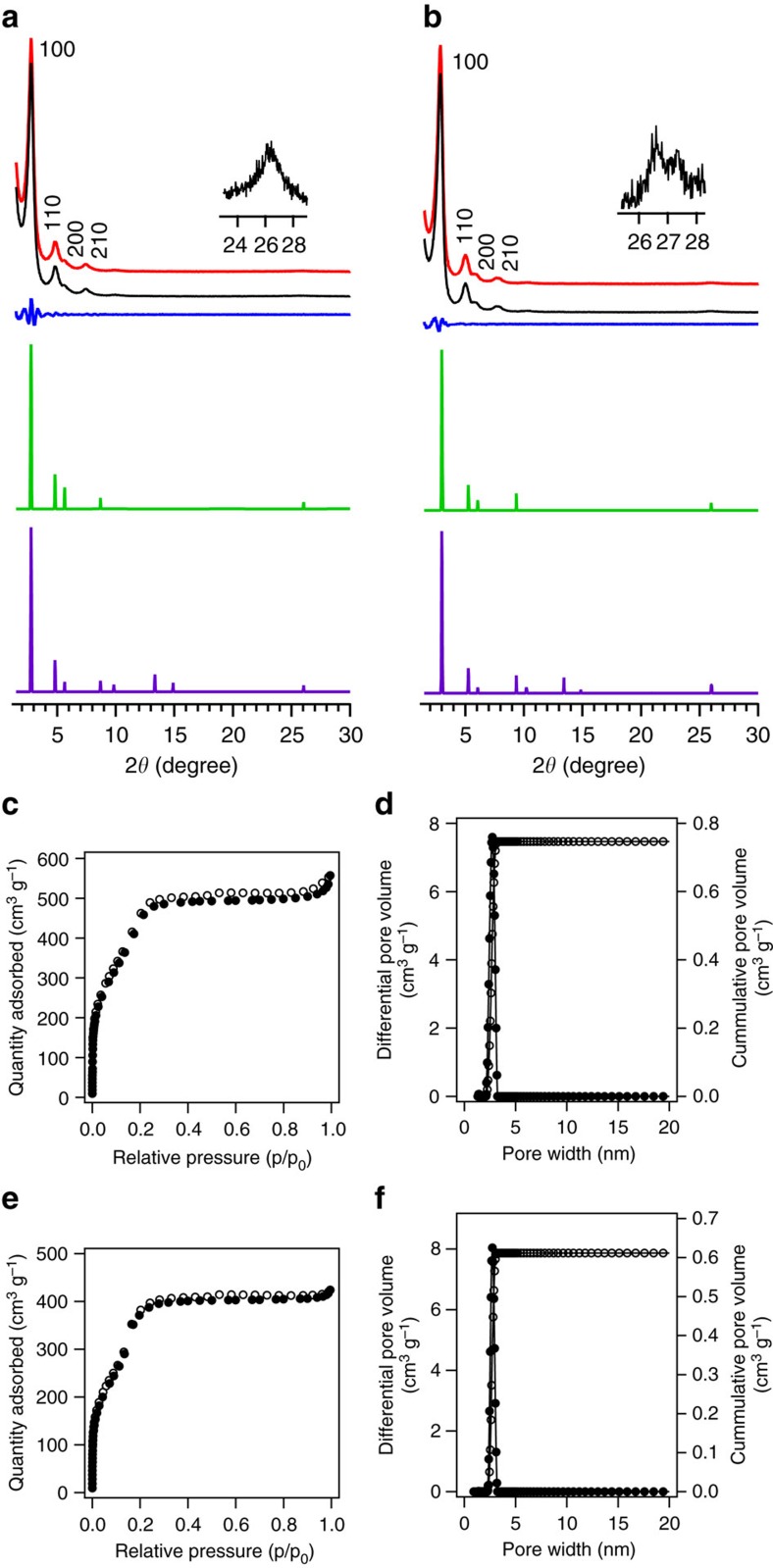
Characterisation of typical [1+2] hexagonal MC-COFs. (**a**,**b**) PXRD patterns of experimentally observed (red curve), Pawley-refined pattern (black curve), their difference (blue curve), slipped AA stacking mode (green curve) and staggered AB stacking mode (purple curve). The crystal facets are shown with indices on the peaks of (**a**) MC-COF-TP-E_1_^1^E_7_^2^ and (**b**) MC-COF-TP-E_1_^2^E_7_^1^. (**c**,**e**) Nitrogen sorption isotherm curves of (**c**) MC-COF-TP-E_1_^1^E_7_^2^ and (**e**) MC-COF-TP-E_1_^2^E_7_^1^ at 77 K (solid dots for adsorption and open circles for desorption). (**d**,**f**) Pore size and its distribution profiles of (**d**) MC-COF-TP-E_1_^1^E_7_^2^ and (**f**) MC-COF-TP-E_1_^2^E_7_^1^.

We conducted three-component [1+2] copolymerisations by using the shortest linker (E_1_) and a long linker (E_7_) at a molar ratio of 1:2 or 2:1 as the linkers to condense with 2, 3, 6, 7, 10, 11-hexahydroxytriphenylene (TP) as the knots (Fig. 2a,b). These two sets of [1+2] three-component reactions were performed in a mixed solvent consisting of mesitylene and dioxane under solvothermal conditions and yielded two MC-COFs (Fig. 3; MC-COF-TP-E_1_^1^E_7_^2^ and MC-COF-TP-E_1_^2^E_7_^1^, whereas E_1_^1^E_7_^2^ is defined as the 1:2 molar ratio for the E_1_ and E_7_ units in the COF; the same definition was used for the other MC-COFs). Their structures were characterized through various methods (Fig. 4; Supplementary Figs 6, 52, 53, 100, 127, 148, 149, 179, 180, 182 and 183; Supplementary Tables 1–6, 12 and 13).

Powder X-ray diffraction (PXRD) measurements of MC-COF-TP-E_1_^1^E_7_^2^ (Fig. 3a) revealed a series of strong peaks at 2*θ* of 2.76°, 4.90°, 5.38°, 7.52°, and 26.04° (Fig. 4a, red curve; Supplementary Table 1), which were assigned to the (100), (110), (200), (210), and (001) facets, respectively. By contrast, MC-COF-TP-E_1_^2^E_7_^1^ exhibited different PXRD peaks at 2.94°, 5.32°, 5.84°, 7.84°, and 26.44° (Fig. 4b, red curve; Supplementary Table 1). Notably, these two sets of PXRD peaks were different from those of the [1+1] counterparts COF-5 (3.48°, 5.99°, 6.96°, 9.18°, and 26.24°)^2^ and TP-COF (2.70°, 4.74°, 5.46°, 7.26°, and 26.32°)^23^. In control experiments, we measured the PXRD patterns of simple mixtures of COF-5 and TP-COF at weight ratios of 1:2 and 2:1. These mixtures showed different PXRD peaks (Supplementary Fig. 6). The Pawley-refined patterns (Fig. 4a,b, black curves) confirmed the PXRD peak assignments because the differences from the observed PXRD pattern were negligible (Fig. 4a,b, blue curves). Structural simulations using self-consistent charge density-functional tight-binding (SCC-DFTB) method^49^ with starting structures created by AuToGraFS and preoptimized using a topology-preserving force field were used to optimize the monolayer and were further extended to layered frameworks with different stacking modes. In both MC-COFs, the slipped AA stacking modes were the most stable structures among the various stacking modes, including eclipsed AA and staggered AB (Fig. 4a,b; Supplementary Tables 5 and 6). We used the slipped AA stacking mode to reconstruct the crystal structures of the two MC-COFs and the resulting PXRD patterns (Fig. 4a,b, green curves) were in agreement with the experimentally observed profiles. MC-COF-TP-E_1_^1^E_7_^2^ assumed a space group of *P*2 with *a*=33.5 Å, *b*=34.0 Å, *c*=6.8 Å, *α*=*β*=90°, and *γ*=66° (for atomic coordinates see Supplementary Table 12), and MC-COF-TP-E_1_^2^E_7_^1^ adopted a *P*2 space group with different lattice parameter of *a*=33.1 Å, *b*=34.0 Å, *c*=6.8 Å, and *α*=*β*=90°, and *γ*=53° (for atomic coordinates see Supplementary Table 13). The cell parameters of MC-COF-TP-E_1_^1^E_7_^2^ were larger than those of MC-COF-TP-E_1_^2^E_7_^1^, because the former contained much longer E_7_ linkers in its lattice. Moreover, the difference in the *a* and *b* values observed for the two MC-COFs was in good agreement with the asymmetric MC tiling of the lattice because the lengths of two sets of parallel linker pairs were different from that of another set of linker pairs (Fig. 3b,c). These space groups and lattice parameters were also different from those of the [1+1] counterparts of COF-5 and TP-COF^2^,^23^. Moreover, the staggered AB stacking modes could not reproduce the PXRD patterns (Fig. 4a,b, purple curves).

To quantitatively determine the ratio of the two linkers in the MC-COFs, we hydrolysed the MC-COF samples with HCl and measured their ^1^H nuclear magnetic resonance (NMR) spectra. Resonances with the predicted coupling patterns were observed in the expected regions for each of the linkers' unique protons. By integrating the resonance peak intensities, the E_1_ and E_7_ linkers were present in ratios of 1:2 for MC-COF-TP-E_1_^1^E_7_^2^ (Supplementary Fig. 52) and 2:1 for MC-COF-TP-E_1_^2^E_7_^1^ (Supplementary Fig. 53). These proton integrations quantitatively confirmed the lattice components of these MC-COFs.

Field emission scanning electron microscopy revealed that MC-COF-TP-E_1_^1^E_7_^2^ and MC-COF-TP-E_1_^2^E_7_^1^ exhibited completely different morphologies (that is, belts and flakes, respectively) (Supplementary Fig. 127). The belts were as large as several micrometres and the flakes were extended to several hundred nanometres. High-resolution transmission electron microscopy was used to visualize their order structures (Supplementary Figs 148 and 149). These observations again confirmed that the resulting MC-COFs were obtained as single phases.

MC-COF-TP-E_1_^1^E_7_^2^ and MC-COF-TP-E_1_^2^E_7_^1^ were highly porous and exhibited typical type-IV sorption isotherm profiles (Fig. 4c,e). The Brunauer–Emmett–Teller (BET) surface areas were 1,892 and 1,534 m^2^ g^–1^ for MC-COF-TP-E_1_^1^E_7_^2^ and MC-COF-TP-E_1_^2^E_7_^1^, respectively (Supplementary Table 3). The pore size distribution profiles revealed that MC-COF-TP-E_1_^1^E_7_^2^ and MC-COF-TP-E_1_^2^E_7_^1^ possessed only one type of mesopore but different pore sizes of 3.2 and 2.9 nm, respectively (Fig. 4d,f). These pore sizes differed from those of the [1+1] counterparts (that is, COF-5 (2.7 nm)^2^ and TP-COF (3.2 nm)^23^). Thermogravimetric analysis (TGA) revealed that MC-COF-TP-E_1_^1^E_7_^2^ and MC-COF-TP-E_1_^2^E_7_^1^ under nitrogen were stable up to 550 and 400 °C, respectively (Supplementary Fig. 100).

### Four-component [1+3] MC-COFs

**Figure 5:**
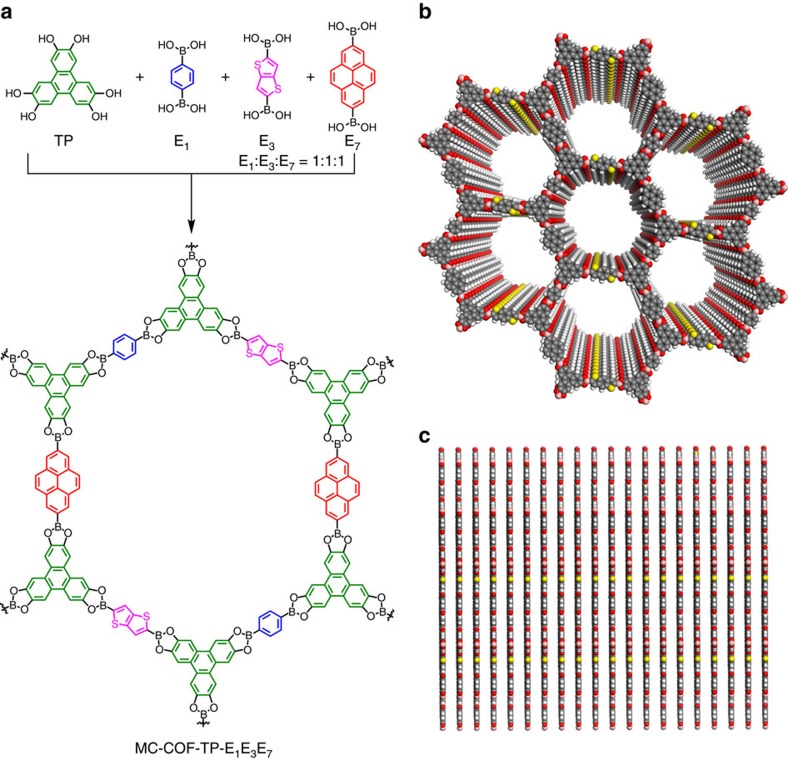
Synthesis and ordered structures of [1+3] hexagonal MC-COFs. (**a**) Schematic of the synthesis of MC-COF-TP-E_1_E_3_E_7_. (**b**) Top view of the slipped AA-stacking structure of MC-COF-TP-E_1_E_3_E_7_. (**c**) Side view of the slipped AA stacking structure of MC-COF-TP-E_1_E_3_E_7_.

**Figure 6:**
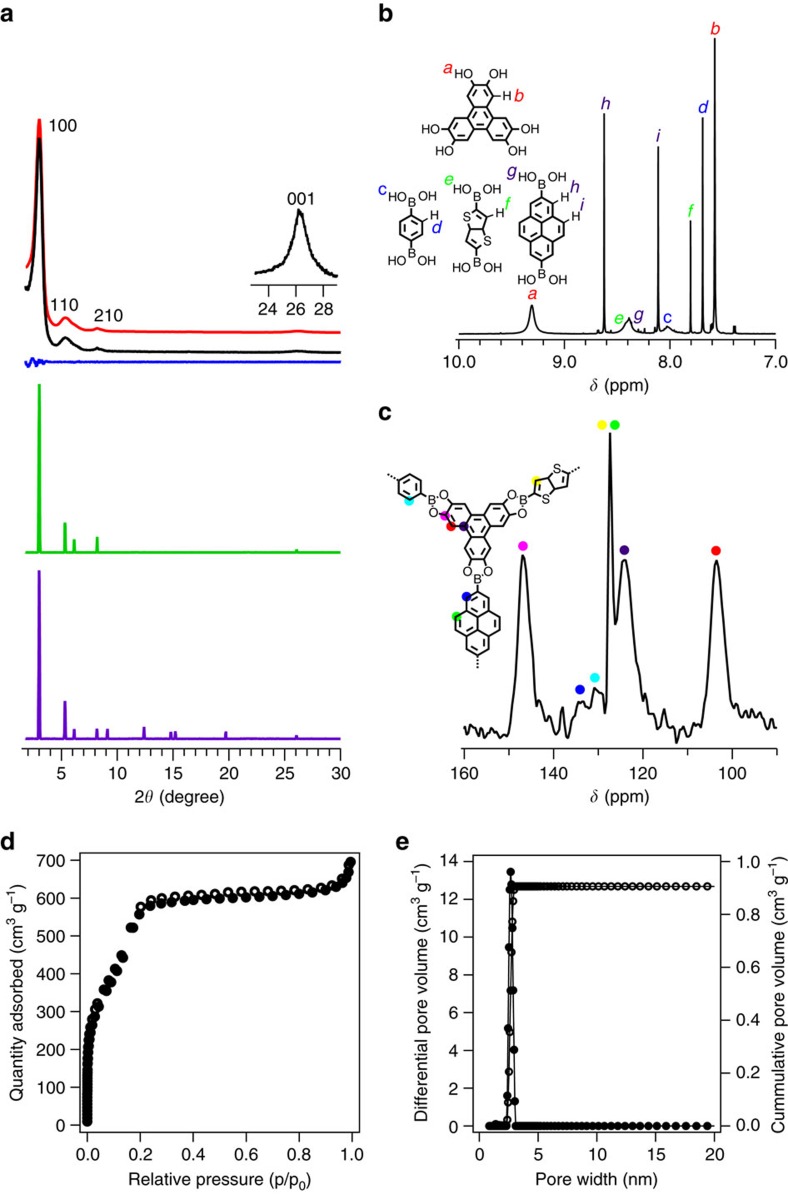
Characterisation of typical [1+3] hexagonal MC-COFs. (**a**) PXRD patterns of experimentally observed (red curve), Pawley-refined pattern (black curve), their difference (blue curve), slipped AA stacking mode (green curve) and staggered AB stacking mode (purple curve). The crystal facets are shown with indices on the peaks of MC-COF-TP-E_1_E_3_E_7_. (**b**) ^1^H NMR spectrum of digested MC-COF-TP-E_1_E_3_E_7_. The proton signals were assigned to different monomers. The proton integral of each peak was used for the quantitative evaluation of the molar ratio of components in the COF. (**c**) Solid-state ^13^C NMR spectrum and carbon signal assignment of MC-COF-TP-E_1_E_3_E_7_. (**d**) Nitrogen sorption isotherm curve of MC-COF-TP-E_1_E_3_E_7_ (solid dots for adsorption and open circles for desorption). (**e**) Pore size and its distribution profiles.

We extended the MC strategy to the [1+3] copolymerisation system for the design and synthesis of four-component hexagonal MC-COFs in which three linkers were integrated at the same molar ratio (Figs 1b and 2c). For example, MC-COF-TP-E_1_E_3_E_7_ (Fig. 5) consisted of TP knots and three different E_1_, E_3_, and E_7_ linkers, and its structure was characterized through various analytic methods (Fig. 6; Supplementary Figs 24, 118, 144, 150, 181, and 186; Supplementary Tables 1–3 and 9).

MC-COF-TP-E_1_E_3_E_7_ exhibited a PXRD pattern with peaks located at 2.84°, 4.92°, 5.58° and 26.24°, which were assigned to the (100), (110), (210) and (001) facets, respectively (Fig. 6a, red curve). These peaks were different from those of the [1+1] counterparts (that is, COF-5, TP-COF and TT-COF)^2^,^23^,^50^ and their mixture (Supplementary Fig. 23). Pawley refinement confirmed the peak assignments (Fig. 6a, black curve), as evident by their negligible differences (Fig. 6a, blue curve). The experimental PXRD pattern was consistent with the simulated pattern (Fig. 6a, green curve) for the most stable slipped AA stacking mode. The slipped AA stacking mode afforded lattice parameters of *a*=33.5 Å, *b*=34.1 Å, *c*=6.8 Å, *α*=*β*=90°, and *γ*=67° (for atomic coordinates see Supplementary Table 16). On the other hand, the staggered AB stacking mode could not reproduce the PXRD pattern (Fig. 6a, purple curve).

The ^1^H NMR spectrum of the digested sample of MC-COF-TP-E_1_E_3_E_7_ revealed that the molar ratio of TP/E_1_/E_3_/ E_7_ was 2/1/1/1 (Fig. 6b). To determine whether free units existed in the frameworks, we performed solid-state ^13^C NMR spectroscopy. The [1+2] MC-COF-TP-E_1_^1^E_7_^2^ and MC-COF-TP-E_1_^2^E_7_^1^ exhibited resonances at 147.5, 131.5, 127.4, 123.5 and 104.7 and at 147.1, 131.7, 126.9, 125.2 and 103.6 parts per million (p.p.m.), which are characteristic of the unique carbon atoms of the TP, E_1_, and E_7_ units (Supplementary Figs 179 and 180). Similarly, the [1+3] MC-COF-TP-E_1_E_3_E_7_ exhibited a series of peaks at 146.3, 131.1, 128.2, 124.8, and 104.4 p.p.m. (Fig. 6c; Supplementary Fig. 181). In contrast, a mixture of the constituent free units of TP, E_1_, E_3_, and E_7_ exhibited resonances at 144.3, 132.2, 128.7, 125.3, and 105.1 p.p.m. (Supplementary Table 4). A distinct shift (up to 3.2 p.p.m.) between the carbons of the free units and those of the units that were incorporated into the frameworks confirmed that no unlinked organic units were present within the MC-COFs. MC-COF-TP-E_1_E_3_E_7_ exhibited a very high BET surface area of 1,887 m^2^ g^–1^ and contained only one type of mesopore with a pore size of 2.9 nm (Fig. 6d,e; Supplementary Table 3).

## Discussion

**Figure 7:**
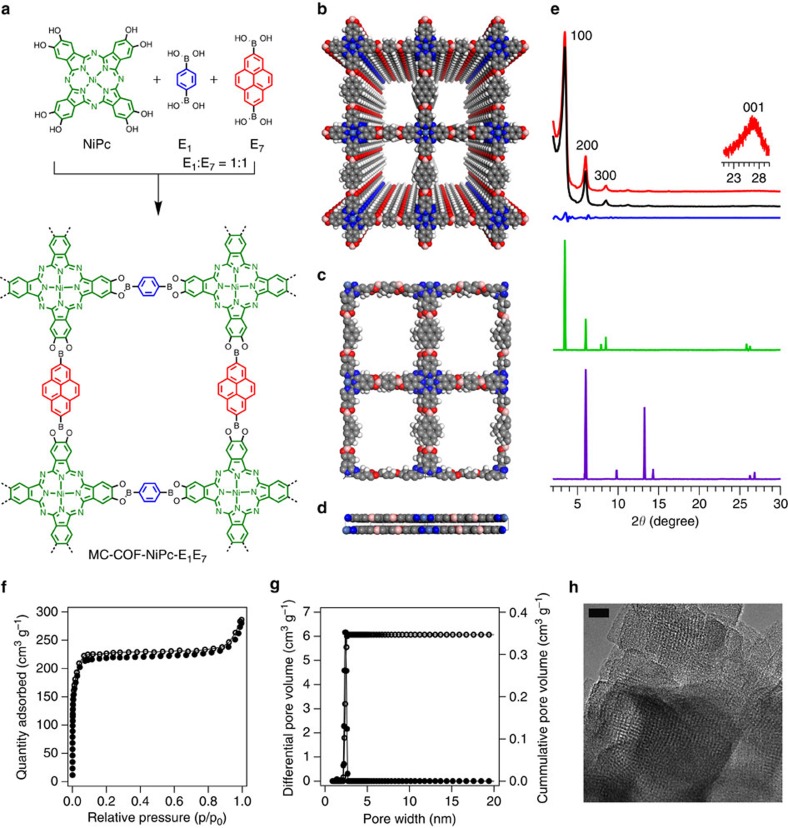
Synthesis and characterisation of tetragonal [1+2] MC-COFs. (**a**) Schematic representation of the synthesis of MC-COF-NiPc-E_1_E_7_. (**b**) Top view of the slipped AA stacking crystal structure of MC-COF-NiPc-E_1_E_7_. (**c**) Top view of the unit cell of MC-COF-NiPc-E_1_E_7_. (**d**) side view of the unit cell of MC-COF-NiPc-E_1_E_7_. (**e**) PXRD patterns of experimentally observed (red curve), Pawley-refined pattern (black curve), their difference (blue curve), slipped AA stacking mode (green curve) and staggered AB stacking mode (purple curve). The crystal facets are shown with indices on the PXRD peaks. (**f**) Nitrogen sorption isotherm curve of MC-COF-NiPc-E_1_E_7_ (solid dots for adsorption and open circles for desorption). (**g**) Pore size and its distribution profiles. (**h**) HR-TEM image of MC-COF-NiPc-E_1_E_7_. The scale bar is 10 nm. HR-TEM, High-resolution transmission electron microscopy.

We further explored this MC strategy by using a NiPc knot and two linkers for the construction of tetragonal MC-COFs (Figs 1b and 2d). For example, MC-COF-NiPc-E_1_E_7_ (Fig. 7a–d) consisted of E_1_ and E_7_ linkers that were parallel matched to form an oblong polygon lattice. The compositions, linkage, crystalline structures, morphology and porosity were unambiguously determined using various analytic methods (Fig. 7e–h; Supplementary Figs 30, 93, 121, 147, 187 and 188; Supplementary Tables 1–3, 10). The PXRD pattern of MC-COF-NiPc-E_1_E_7_ exhibited strong diffraction peaks at 3.44°, 6.02, 8.52 and 26.62°, which were assigned to the (100), (200), (300) and (001) facets, respectively (Fig. 7e, red curve). This PXRD pattern differed from those of [1+1] two-component COFs (that is, NiPc-COF^45^ and NiPc-Py-COF^46^) and their mixture (Supplementary Fig. 30). The negligible difference between the Pawley-refined PXRD pattern (Fig. 7e, black and blue curves) and the experimentally observed profile supported the peak assignments. The slipped AA stacking mode (Fig. 7c,d) was the most stable structure that reproduced the PXRD pattern (green curve) and adopted a *P*222 space group with *a*=23.0 Å, *b*=27.3 Å, *c*=6.7 Å, *α*=*β*=90°, and *γ*=90° (for atomic coordinates see Supplementary Table 17). This difference between the *a* and *b* values indicated asymmetric tiling of the two linkers in the tetragonal lattice. The staggered AB stacking mode gives rise to a PXRD pattern (Fig. 7e, purple curve) that is different from that of experimentally observed one. MC-COF-NiPc-E_1_E_7_ was highly porous, with a BET surface area of 672 m^2^ g^–1^ and included one kind of mesopore with a pore size of 2.6 nm (Fig. 7f,g; Supplementary Table 1).

The conventional [1+1] strategy generated 10 hexagonal and 10 tetragonal COFs for TP and NiPc knots combined with 10 linkers (Figs 1a and 2a). In contrast, the three-component [1+2] and four-component [1+3] systems yielded 90 and 120 hexagonal COFs, respectively, whereas the [1+2] tetragonal strategy yielded 45 different COFs. Therefore, the MC strategy greatly enhanced the number of COF structures from 20 to 255. Among these structures, we randomly choose 53 combinations and prepared 53 different COFs (Fig. 2b–d). Notably, the MC strategy was compatible with various linkers with lengths from 7 to 22 Å, structures ranging from simple arenes to heterocycles, and large *π*-systems that can be predesigned with electron-donating and accepting functions (Fig. 2).

In addition to considerably enhanced structural diversity, the MC strategy had two profound effects on the structural development of COFs. (1) This strategy provides a method for preparing tailor-made, specially shaped pores that are difficult to achieve with other porous materials and might have applications in shape-selective separation and catalysis^24^,^25^,^26^,^27^,^28^,^29^,^44^,^51^. (2) This strategy also enhances the structural complexity while retaining its *π*-periodicity. This effect expands the designability of the structures and functions of COFs. For example, the four-component COFs consist of periodic arrays of four different *π*-columns that are sequenced but have varied structures and functions.

**Figure 8:**
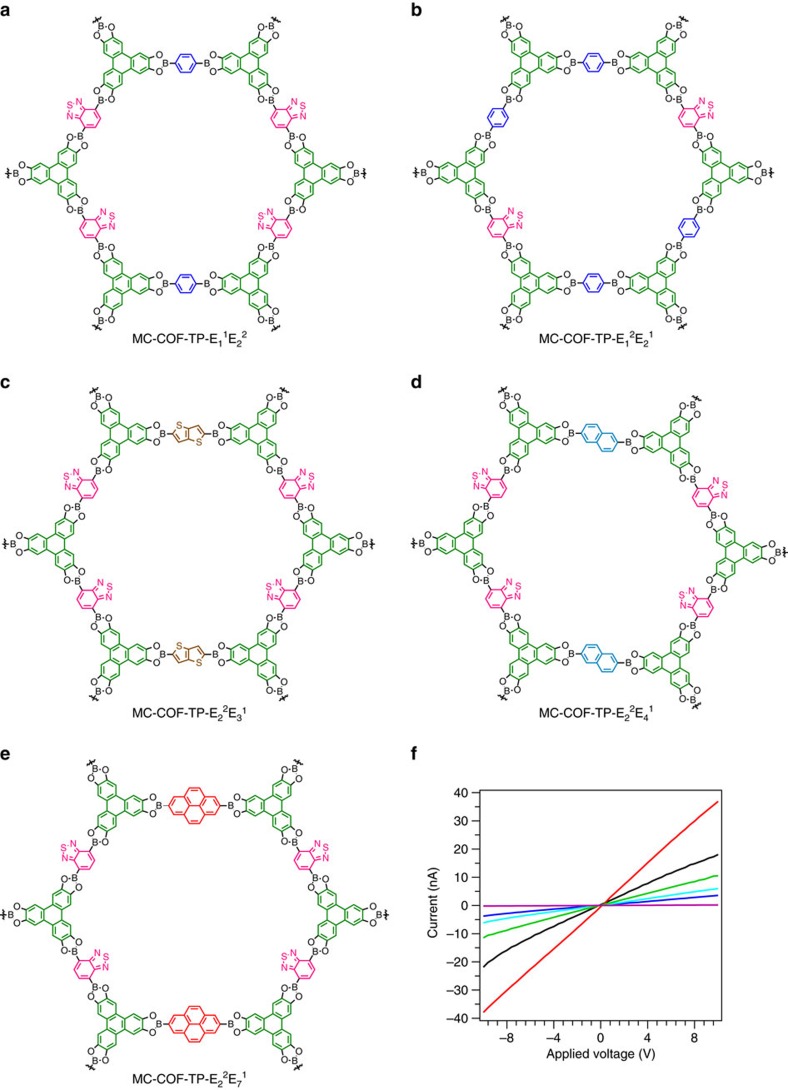
Electron donor–acceptor MC-COFs and conducting property. (**a**–**e**) Schematic of (**a**) MC-COF-TP-E_1_^2^E_2_^1^, (**b**) MC-COF-TP-E_1_^1^E_2_^2^, (**c**) MC-COF-TP-E_2_^2^E_3_^1^, (**d**) MC-COF-TP-E_2_^2^E_4_^1^, (**e**) MC-COF-TP-E_2_^2^E_7_^1^. (**f**) *I*–*V* curves of MC-COF-TP-E_1_^1^E_2_^2^ (blue; 3.8 nA at 10 V), MC-COF-TP-E_1_^2^E_2_^1^ (cyan; 6.1 nA at 10 V), MC-COF-TP-E_2_^2^E_3_^1^ (red; 37.9 nA at 10 V), MC-COF-TP-E_2_^2^E_4_^1^ (green; 11.47 nA at 10 V), MC-COF-TP-E_2_^2^E_7_^1^ (black; 21.9 nA at 10 V), TT-COF (pink; 0.037 nA at 10 V), and DA-COF (purple; 0.02 nA at 10 V).

Along this line of study, we further explored the possibility for the integration of MC electron-donating and -accepting units into MC-COFs in which the sequenced *π*-arrays may trigger strong electronic correlations among the latticed *π*-components. The [1+2] copolymerisation of electron-donating TP knots with an electron-accepting E_2_ linker and other electron-donating E_1_, E_3_, E_4_ and E_7_ linkers yielded a series of electron donor–acceptor MC-COFs, including MC-COF-TP-E_1_^1^E_2_^2^, MC-COF-TP-E_1_^2^E_2_^1^, MC-COF-TP-E_2_^2^E_3_^1^, MC-COF-TP-E_2_^2^E_4_^1^ and MC-COF-TP-E_2_^2^E_7_^1^ (Fig. 8a–e). These MC-COFs triggered charge transfer from the TP knots to the E_2_ linkers, while the lattice tiling patterns tuned the charge-transferring capability, as evidenced by the different degrees of red-shifting of the band in the near infrared region of the electronic absorption spectra (Supplementary Figs 190–193). Notably, these MC-COFs exhibited ohmic-type conducting profiles but different currents (Fig. 8f). An enhancement of nearly 180,000% was observed for MC-COF-TP-E_2_^2^E_3_^1^ (red) compared with the conventional [1+1] counterparts (that is, D-A COF (purple)^52^ and TT-COF (pink)^50^). These results confirmed that the properties of the MC-COFs are not simple linear sums of their [1+1] two-component counterparts, thus supporting the notion that the sequence and high order of the *π*-units within the MC-COFs may be useful for enhancing a specific property or achieving a new function.

In summary, we have developed a general strategy for the design and synthesis of crystalline porous COFs that enable the integration of multiple components into lattice structures with sequenced alignment. The multiple-component COFs greatly expand the structural complexity via asymmetric tiling of building blocks, providing a new platform for constructing anisotropic *π*-columnar arrays and unconventionally shaped pores. At the same time, this strategy considerably increases the structural diversity of COFs while retaining high crystallinity and porosity. We envisage that the MC-COFs constitute an important step towards various unprecedented molecular systems for functional exploration with enhanced structural complexity and diversity that are hardly available for conventional COFs architectures and other porous materials.

## Methods

### General procedure for three-component [1+2] hexagonal MC-COFs

A mesitylene/dioxane (0.5 ml/0.5 ml) mixture of TP (0.022 mmol, 14.9 mg) and two edge units (total 0.033 mmol) at different molar ratios of 1/2 and 2/1 in a Pyrex tube (10 ml) was degassed by using three freeze–pump–thaw cycles (Supplementary Note 1). The tube was sealed off using flame and heated at 120 °C for 3 days. The precipitate was collected via centrifuge and washed with anhydrous acetone for 5 times. The powder was dried at 120 °C under vacuum overnight to yield the corresponding MC-COFs. Yields between 87 and 96%.

### General procedure for four-component [1+3] hexagonal MC-COFs

A mesitylene/dioxane (0.5 ml/0.5 ml) mixture of TP (0.022 mmol, 14.9 mg) and three edge units (total 0.033 mmol) at molar ratio of 1/1/1 in a Pyrex tube (10 ml) was degassed by using three freeze–pump–thaw cycles. The tube was sealed off using flame and heated at 120 °C for 3 days. The precipitate was collected via centrifuge and washed with anhydrous acetone for five times. The powder was dried at 120 °C under vacuum overnight to yield the corresponding MC-COFs. Yields between 82 and 94%.

### General procedure for three-component [1+2] tetragonal MC-COFs

A mesitylene/dioxane (0.5 ml/0.5 ml) mixture of NiPc (0.022 mmol, 14.9 mg) and two edge units (total 0.044 mmol) at the ratio of 1/1 in a Pyrex tube (10 ml) was degassed by using three freeze–pump–thaw cycles. The tube was sealed off using flame and heated at 120 °C for 3 days. The precipitate was collected via centrifuge and washed with anhydrous acetone for five times. The powder was dried at 120 °C under vacuum overnight to yield the corresponding MC-COFs.

### ^1^H NMR spectroscopy

In general, dried MC-COF samples (10 mg) were dissolved in 1.0 ml HCl solution (20 wt% aqueous HCl solution) on sonication and digested for 12 h at 25 °C. The mixture was dried under vacuum for 16 h. Then *d*_6_-dimethyl sulfoxide (600 μl) was added to dissolve the resulting solid for ^1^H NMR spectroscopy. The proton signals and their integrations were used for the quantitative determination of the components of MC-COFs.

### Conductivity measurement

MC-COFs samples (5 mg) were dispersed in 2 ml anhydrous dichloromethane and sonicated for 10 min. The highly dispersed MC-COFs solution was dropped on the center of conducting electrodes as a film. The measurement was conducted on MC-COF films between 10 μm platinum electrodes at 25 °C in Ar using a two-probe method with a subfemtoamp sourcemeter (Keithley 6,430). *I*–*V* curves were recorded at bias voltages from −10 to 10 V.

### Structural characterization

^1^H NMR spectra were recorded on JEOL models JNM-LA400 NMR spectrometers, where chemical shifts (*δ* in p.p.m.) were determined with a residual proton of the solvent as standard. Solid-state ^13^C NMR spectra were recorded on JEOL model 920 MHz NMR spectrometer with a magnetic field of 21.62 Tesla. The frequency of the rotors was 15 kHz. For solid-state cross-polarization magic angle spinning ^13^C nuclear magnetic resonance (^13^C CP/MAS NMR), cross polarization with polarization inversion 1,808 scans (^13^C CPPI) and cross polarization with non-quaternary suppression (^13^C CPNQS) were performed with a delay time of 5 s. Ultraviolet–vis–infrared diffuse reflectance spectrum (Kubelka–Munk spectrum) was recorded on a JASCO model V-670 spectrometer equipped with integration sphere model IJN-727. Field-emission scanning electron microscopy was performed on a JEOL model JSM-6700 operating at an accelerating voltage of 5.0 kV. The sample was prepared by drop-casting a supersonicated solvents suspension onto mica substrate and then coated with gold. High-resolution transmission electron microscopy images were obtained on a JEOL model JEM-3200 microscopy. The sample was prepared by drop-casting a supersonicated tetrahydrofuran suspension of the COFs onto a copper grid. PXRD data were recorded on a Rigaku model RINT Ultima III diffractometer by depositing powder on glass substrate, from 2*θ*=1.5° up to 30° with 0.02° increment. Elemental analysis was performed on a Yanako CORDER MT-6 elemental analyser. Thermogravimetric analysis measurements were performed on a Mettler-Toledo model TGA/SDTA851 under N_2_, by heating to 800 °C at a rate of 10 °C min^–1^ with samples held in aluminium pans. Nitrogen sorption isotherms were measured at 77 K with Micromeritics Instrument Corporation model 3Flex surface characterization analyser. Before measurement, the samples were degassed in vacuum at 120 °C for more than 10 h. By using the non-local density functional theory (NLDFT) model, the pore volume was derived from the sorption curve.

### Computational calculations

The structures of COFs were calculated using the density-functional tight-binding (DFTB+) method including Lennard–Jones dispersion. The calculations were carried out with the DFTB+ program package version 1.2. DFTB is an approximate density functional theory method based on the tight-binding approach and utilizes an optimized minimal LCAO Slater-type all-valence basis set in combination with a two-center approximation for Hamiltonian matrix elements. The Coulombic interaction between partial atomic charges was determined using the self-consistent charge (SCC) formalism. Lennard–Jones type dispersion was employed in all calculations to describe van der Waals (vdW) and *π*-stacking interactions with starting structures created by AuToGraFS^53^ and preoptimized using a topology-preserving force field^54^ were used to optimize the monolayer and were further extended to layered frameworks with different stacking modes. The lattice dimensions were optimized simultaneously with the geometry. Standard DFTB parameters for X–Y element pair (X, Y=C, O, H and N) interactions were employed from the mio-0–1 set10. The accessible surface areas were calculated from the Monte Carlo integration technique using a nitrogen-size probe molecule (diameter=3.68 Å) roll over the framework surface with a grid interval of 0.25 Å. The X-ray diffraction pattern simulation was performed in a software package for crystal determination from PXRD pattern, implemented in MS modeling version 4.4 (Accelrys Inc.). We performed Pawley refinement to optimize the lattice parameters iteratively until the RP and RWP values converge. The pseudo-Voigt profile function was used for whole profile fitting and Berrar–Baldinozzi function was used for asymmetry correction during the refinement processes.

### Data availability

The data that support the findings of this study are available from the corresponding author on request.

## Additional information

**How to cite this article:** Huang, N. *et al.* Multiple-component covalent organic frameworks. *Nat. Commun.* 7:12325 doi: 10.1038/ncomms12325 (2016).

## Supplementary Material

Supplementary Information Supplementary Figures 1-193, Supplementary Tables 1-18, Supplementary Note 1 and Supplementary References
