# Supplementary material for: Multiple-component covalent organic frameworks
Source: Nat Commun. 2016 Jul 27;7:12325. doi: 10.1038/ncomms12325 (PMC4974470; doi:10.1038/ncomms12325)
Supplement: Supplementary Information — Supplementary Figures 1-193, Supplementary Tables 1-18, Supplementary Note 1 and Supplementary References [file ncomms12325-s1.pdf]

Supplemenatry Figures

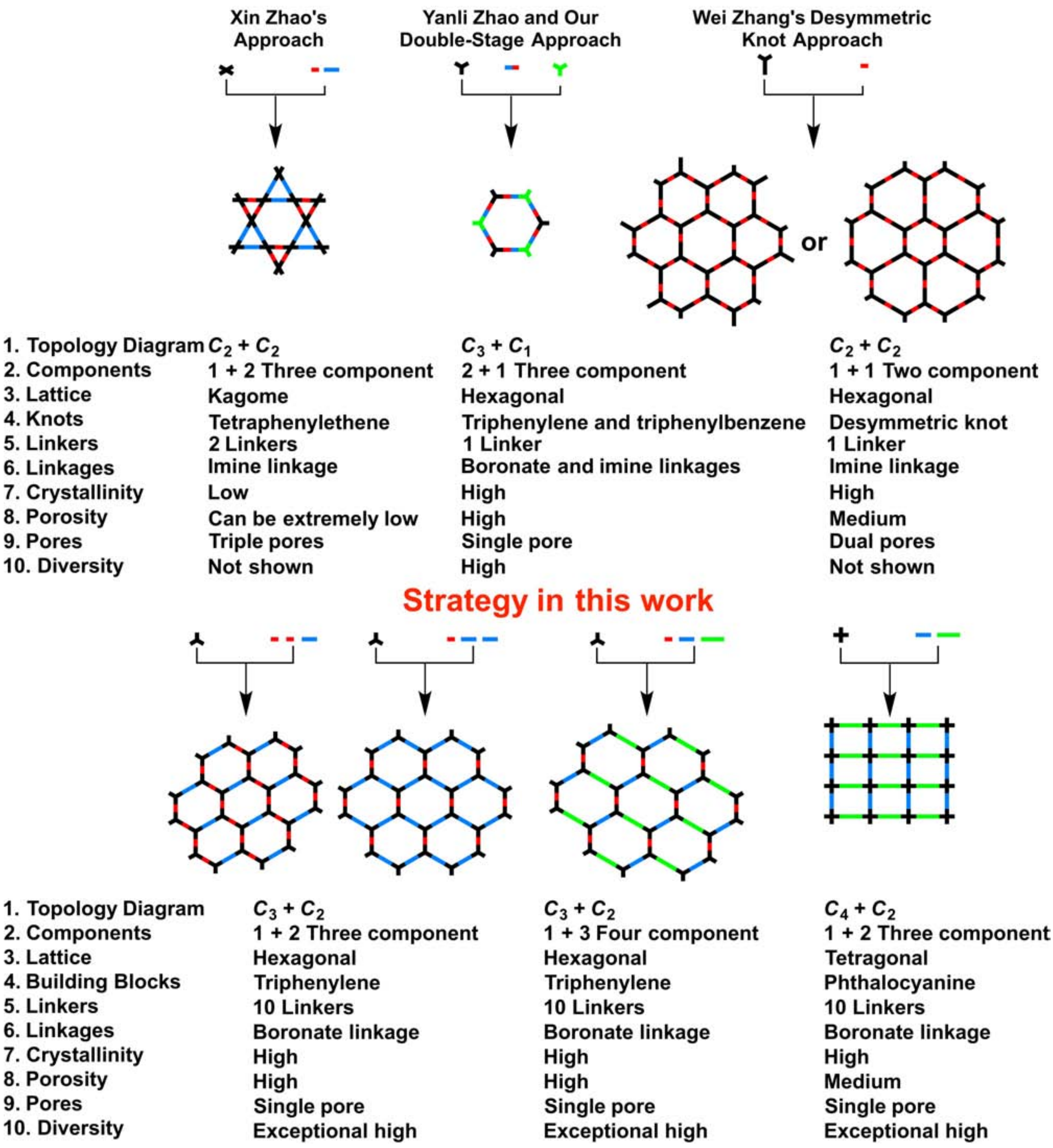

Supplementary Figure 1 | The comparison of our multiple-component covalent organic frameworks (MC-COFs) strategy with other approaches reported.

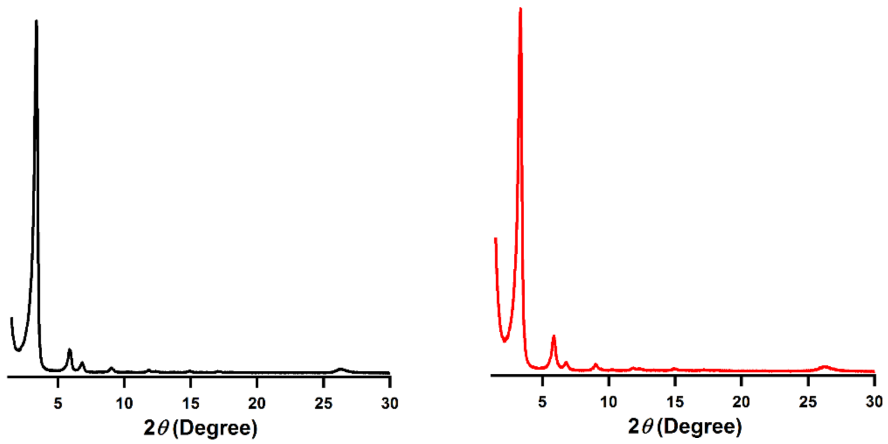

Supplementary Figure 2 | The experimental PXRD patterns of MC-COF-TP-$E_1^1E_2^2$ (black) and MC-COF-TP-$E_1^2E_2^1$ (red).

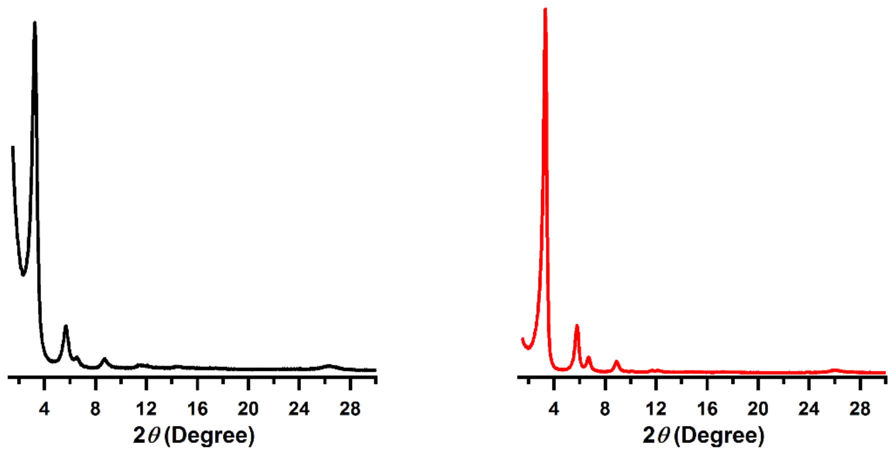

Supplementary Figure 3 | The experimental PXRD patterns of MC-COF-TP-$E_1^1E_3^2$ (black curve) and MC-COF-TP-$E_1^2E_3^1$ (red curve).

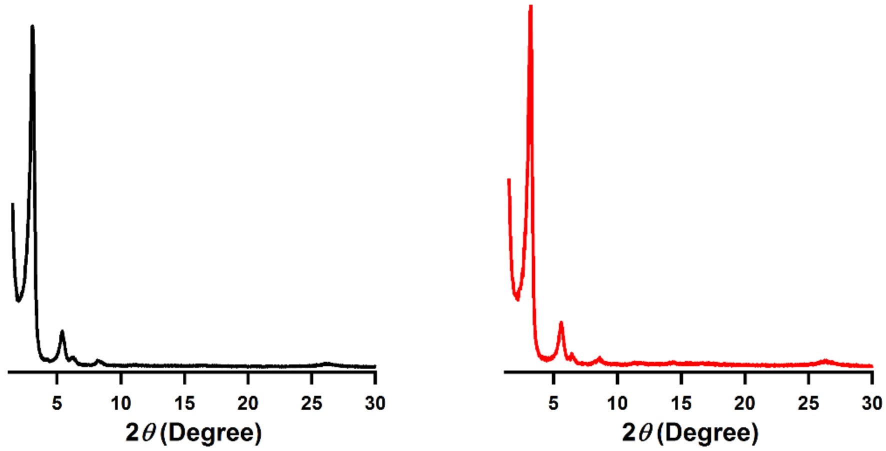

Supplementary Figure 4 | The experimental PXRD pattern of MC-COF-TP-$E_1^1E_4^2$ (black curve) and MC-COF- $E_1^2E_4^1$ (red curve).

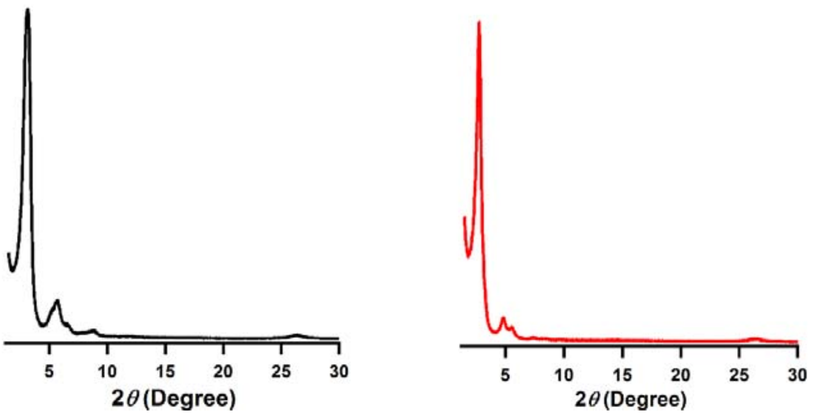

Supplementary Figure 5 | The experimental PXRD patterns of MC-COF-TP-$E_1{}^1E_6{}^2$ (black curve) and MC-COF- $E_1{}^2E_6{}^1$ (red curve).

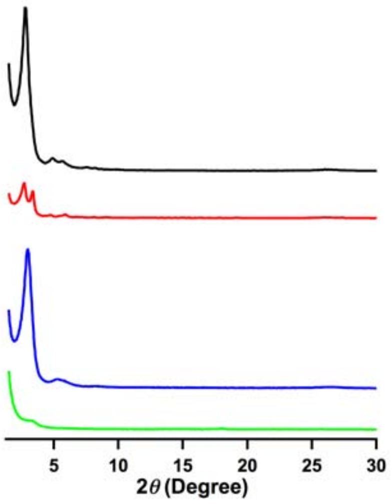

Supplementary Figure 6 | The experimental PXRD patterns of MC-COF-TP-$E_1{}^1E_7{}^2$ (black curve), the mixture of [1 + 1] two-component COF-5 and TP-COF at weight ratio of 1/2 (red curve), MC-COF-TP-$E_1{}^2E_7{}^1$ (blue curve), and the mixture of [1 + 1] two-component COF-5 and TP-COF at weight ratio of 2/1 (green curve).

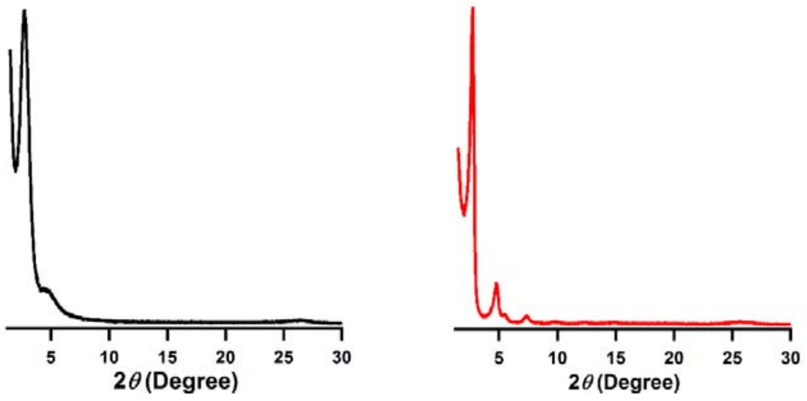

Supplementary Figure 7 | The experimental PXRD patterns of MC-COF-TP-$E_1{}^1E_8{}^2$ (black curve) and MC-COF- $E_1{}^2E_8{}^1$ (red curve).

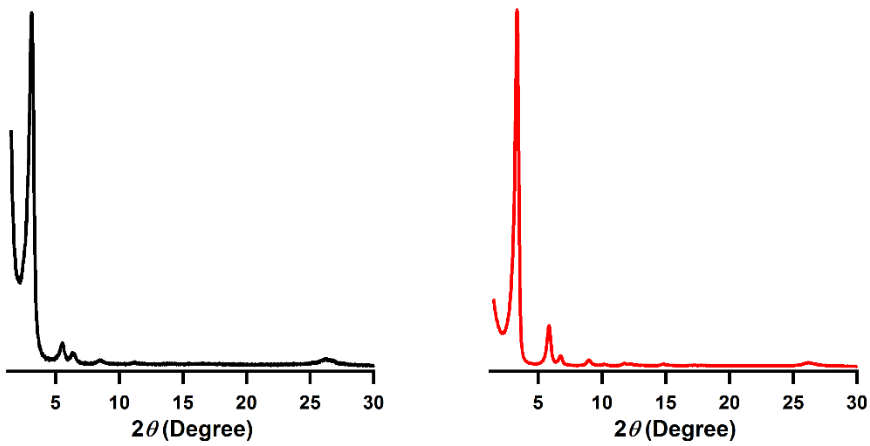

Supplementary Figure 8 | The experimental PXRD patterns of MC-COF-TP-$E_2^1E_3^2$ (black curve) and MC-COF- $E_2^2E_3^1$ (red curve).

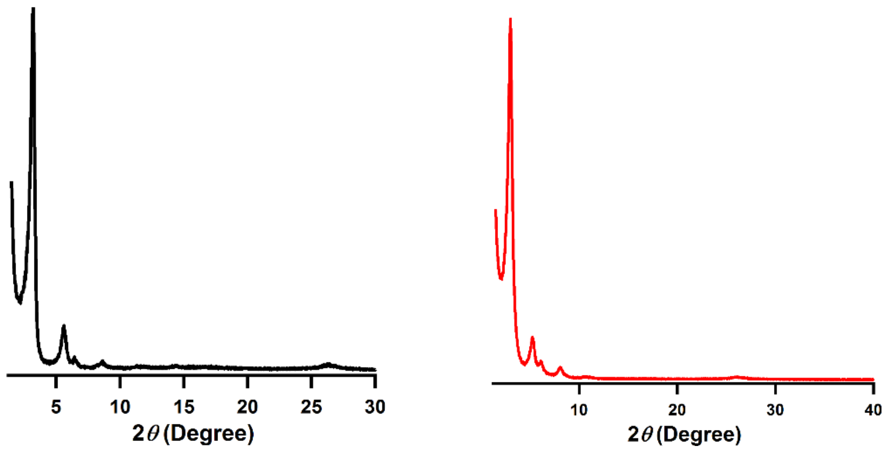

Supplementary Figure 9 | The experimental PXRD pattern of MC-COF-TP-$E_2^1E_4^2$ (black curve) and MC-COF- $E_2^2E_4^1$ (red curve).

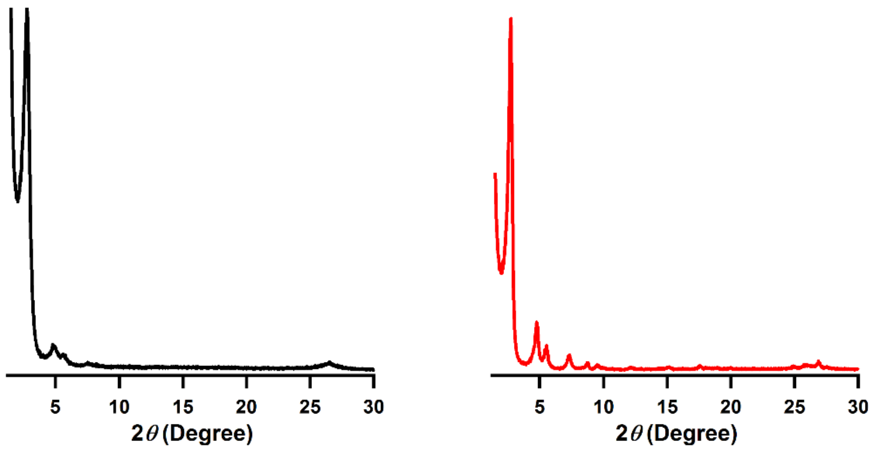

Supplementary Figure 10 | The experimental PXRD pattern of MC-COF-TP-$E_2^1E_6^2$ (black curve) and MC-COF- $E_2^2E_6^1$ (red curve).

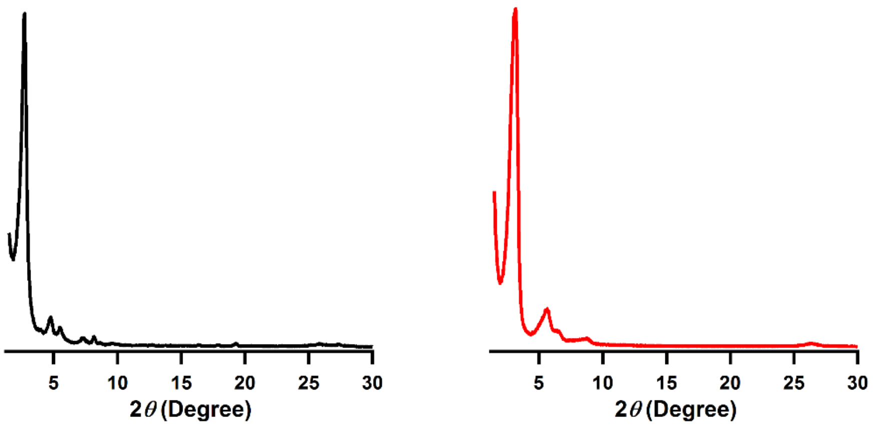

Supplementary Figure 11 | The experimental PXRD patterns of MC-COF-TP-$E_2^1E_7^2$ (black curve) and MC-COF-TP-$E_2^2E_7^1$ (red curve).

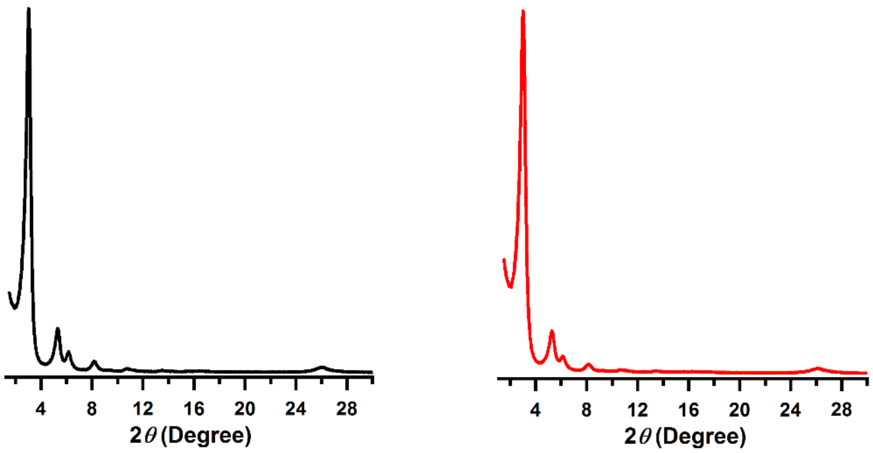

Supplementary Figure 12 | The experimental PXRD patterns of MC-COF-TP-$E_3^1E_4^2$ (black curve) and MC-COF-TP-$E_3^2E_4^1$ (red curve).

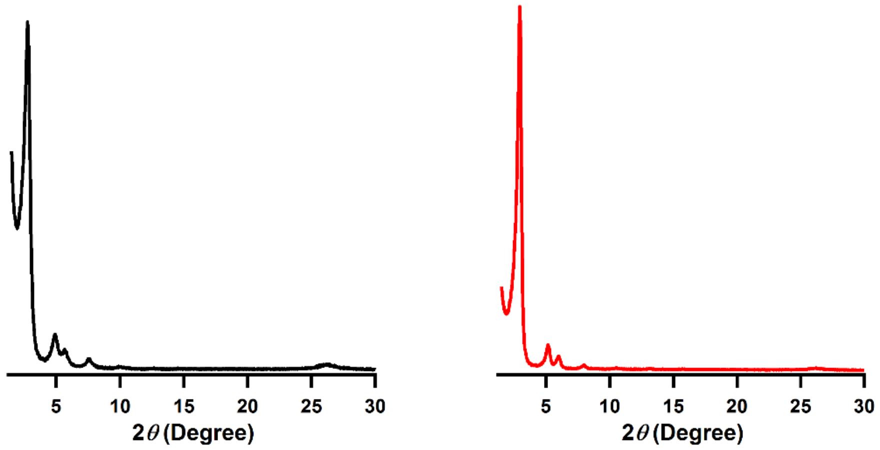

Supplementary Figure 13 | The experimental PXRD patterns of MC-COF-TP-$E_3^1E_6^2$ (black curve) and MC-COF-TP $E_3^2E_6^1$ (red curve).

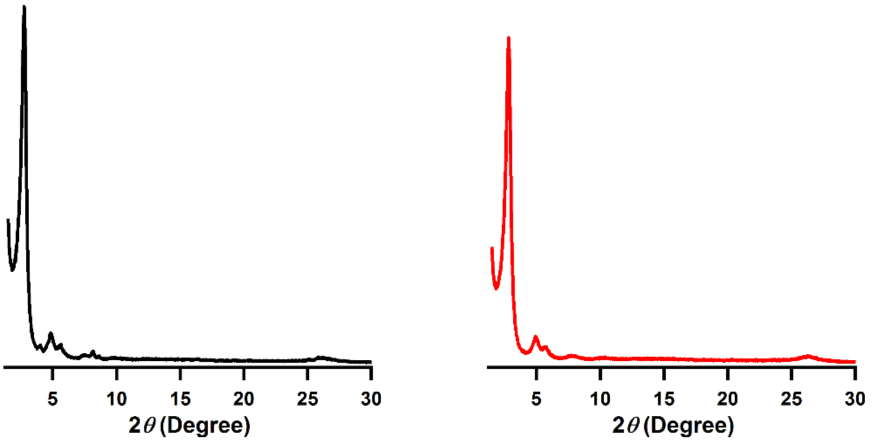

Supplementary Figure 14 | The experimental PXRD patterns of MC-COF-TP-$E_3^1E_7^2$ (black curve) and MC-COF-TP-$E_3^2E_7^1$ (red curve).

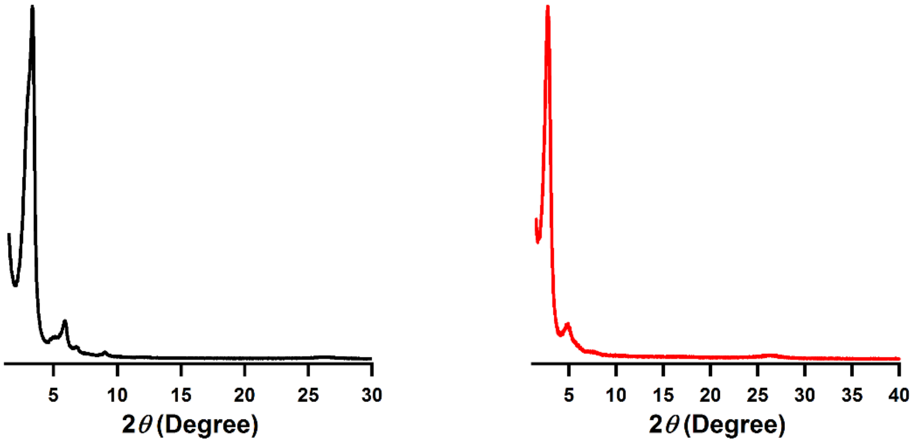

Supplementary Figure 15 | The experimental PXRD patterns of MC-COF-TP-$E_3^1E_8^2$ (black curve) and MC-COF-TP-$E_3^2E_8^1$ (red curve).

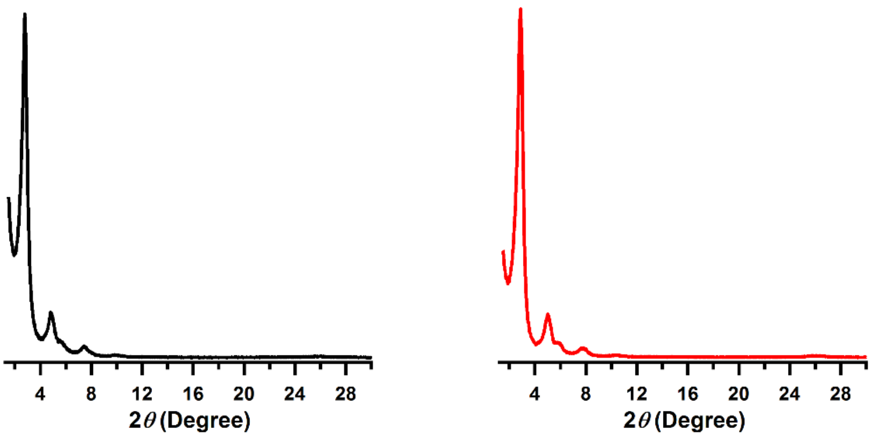

Supplementary Figure 16 | The experimental PXRD patterns of MC-COF-TP-$E_4^1E_5^2$ (black curve) and MC-COF-TP-$E_4^2E_5^1$ (red curve).

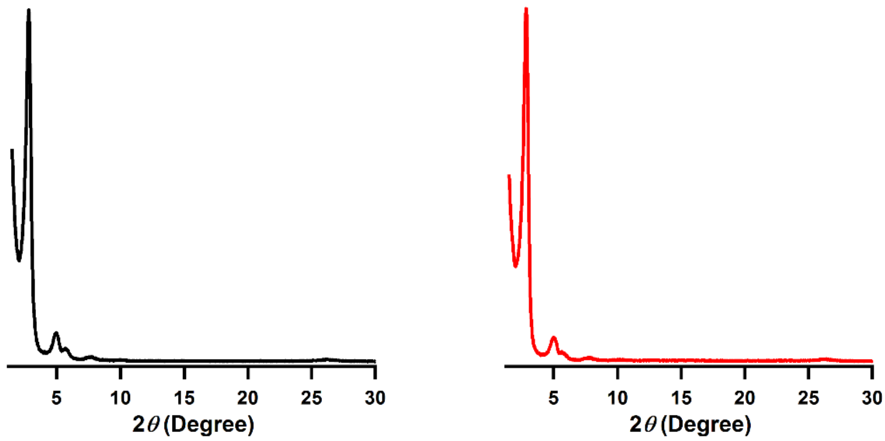

Supplementary Figure 17 | The experimental PXRD patterns of MC-COF-TP-$E_4^1E_7^2$ (black curve) and MC-COF-TP-$E_4^2E_7^1$ (red curve).

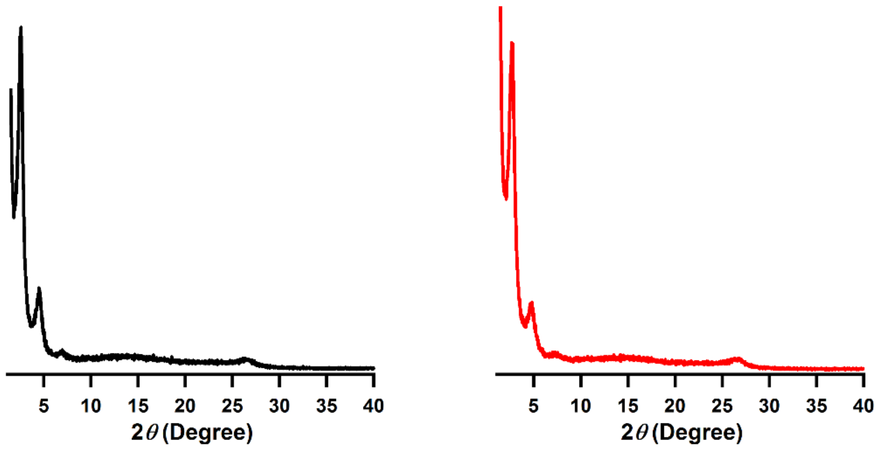

Supplementary Figure 18 | The experimental PXRD patterns of MC-COF-TP-$E_4^1E_8^2$ (black curve) and MC-COF-TP-$E_4^2E_8^1$ (red curve).

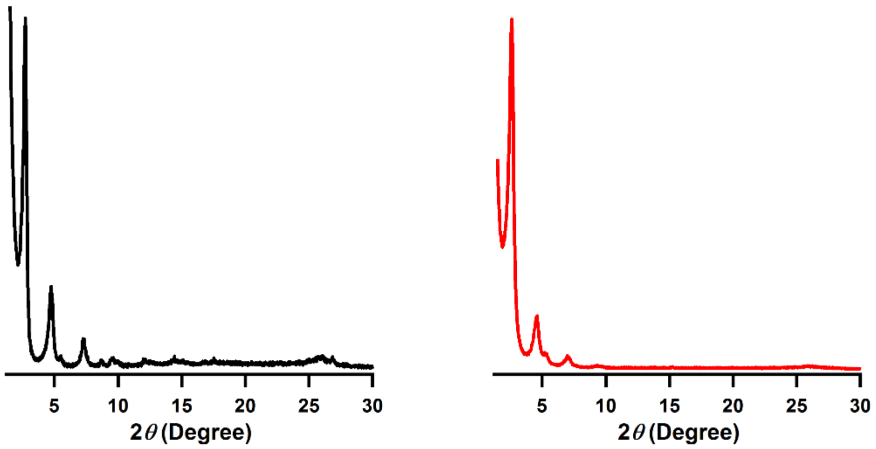

Supplementary Figure 19 | The experimental PXRD patterns of MC-COF-TP-$E_5^1E_6^2$ (black curve) and MC-COF-TP-$E_5^2E_6^1$ (red curve).

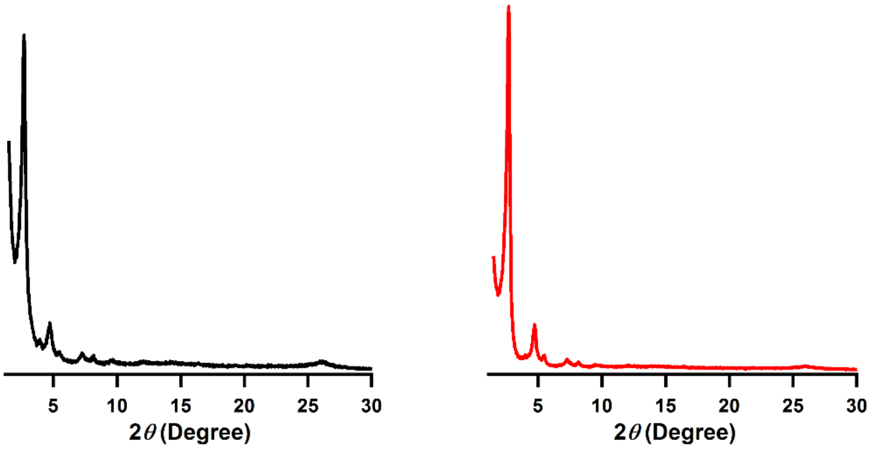

Supplementary Figure 20 | The experimental PXRD patterns of MC-COF-TP-$E_5^1E_7^2$ (black curve) and MC-COF-TP-$E_5^2E_7^1$ (red curve).

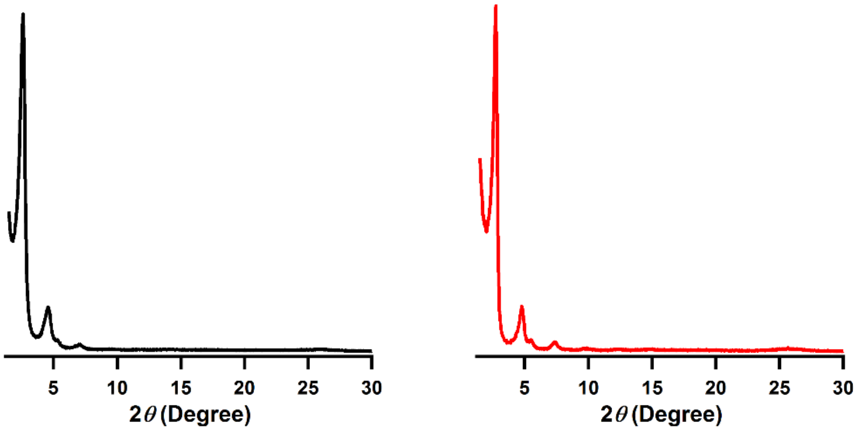

Supplementary Figure 21 | The experimental PXRD patterns of MC-COF-TP-$E_5^1E_8^2$ (black curve) and MC-COF-TP-$E_5^2E_8^1$ (red curve).

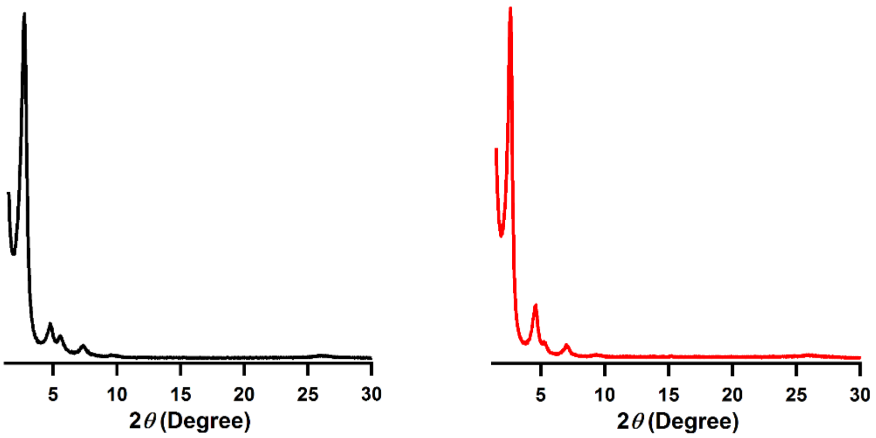

Supplementary Figure 22 | The experimental PXRD patterns of MC-COF-TP-$E_6^1E_7^2$ (black curve) and MC-COF-TP-$E_6^2E_7^1$ (red curve).

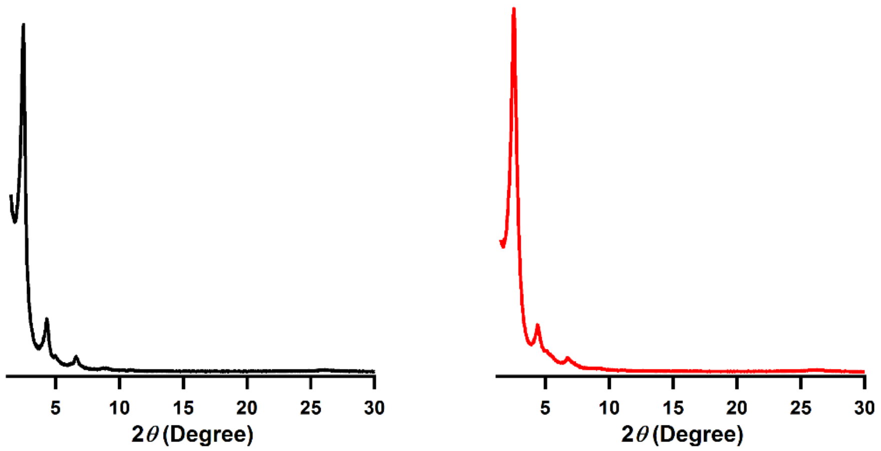

Supplementary Figure 23 | The experimental PXRD pattern of MC-COF-TP-$E_7^1E_8^2$ (black curve) and MC-COF-TP-$E_7^2E_8^1$ (red curve).

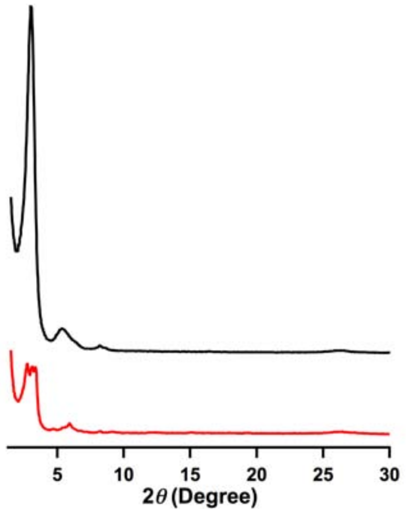

Supplementary Figure 24 | The experimental PXRD pattern of MC-COF-TP-$E_1E_3E_7$ (black curve) and the mixture of [1 + 1] two-component COF-5, TP-COF, and TT-COF at weight ratio of 1/1/1 (red curve).

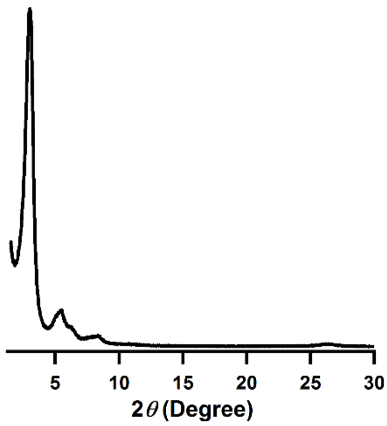

Supplementary Figure 25 | The experimental PXRD pattern of MC-COF-TP-$E_1E_4E_7$.

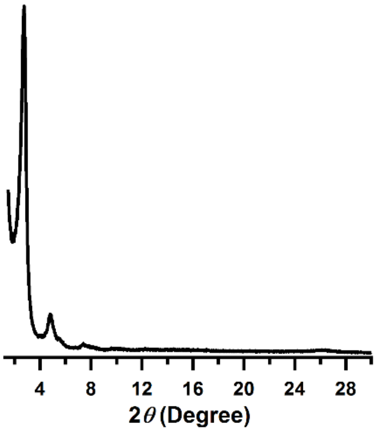

Supplementary Figure 26 | The experimental PXRD pattern of MC-COF-TP-$E_4E_5E_7$.

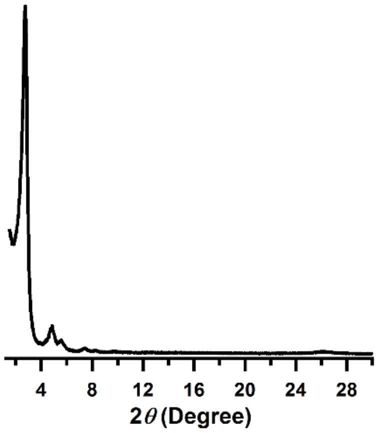

Supplementary Figure 27 | The experimental PXRD pattern of MC-COF-TP- $E_4E_6E_7$.

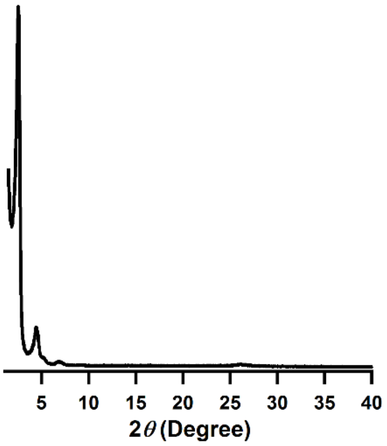

Supplementary Figure 28 | The experimental PXRD pattern of MC-COF-TP- $E_5E_7E_8$.

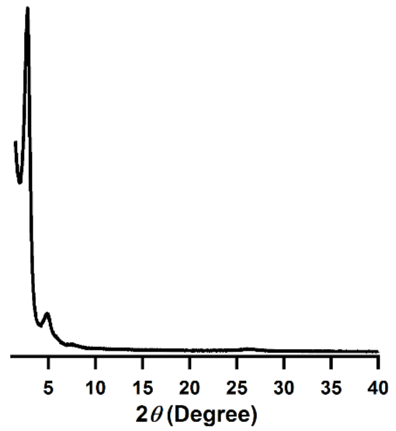

Supplementary Figure 29 | The experimental PXRD pattern of MC-COF-TP-$E_3E_4E_8$.

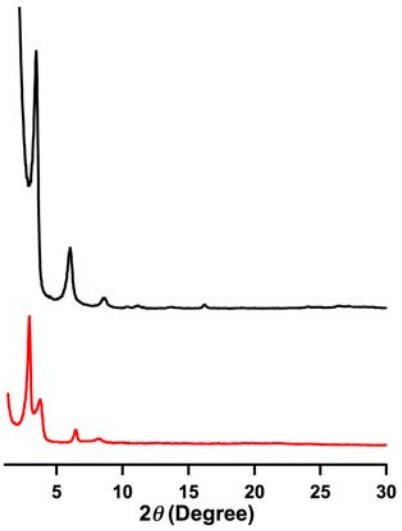

Supplementary Figure 30 | The experimental PXRD pattern of MC-COF-NiPc-E_1E_7 (black curve) and the mixture of [1 + 1] two-component NiPc-COF-E_1 and NiPc-COF-E_7 at weight ratio of 1/1(red curve).

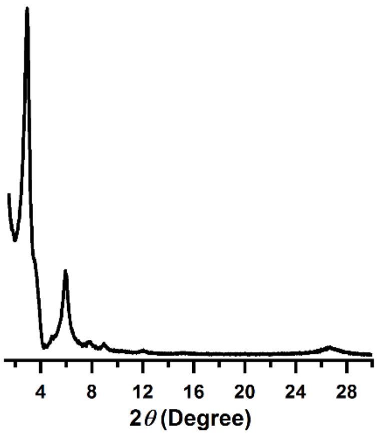

Supplementary Figure 31 | The experimental PXRD pattern of MC-COF-NiPc-E_7E_9.

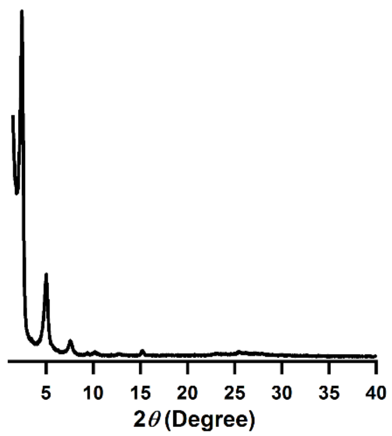

Supplementary Figure 32 | The experimental PXRD pattern of MC-COF-NiPc-E_9E_{10}.

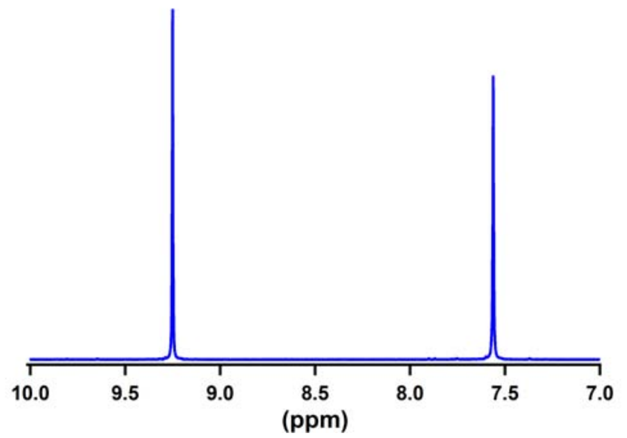

Supplementary Figure 33 | ^{1}H NMR spectrum of HHTP (400 MHz, d_6-DMSO). δ (ppm) 9.26 (s, 6H), 7.57 (s, 6H).

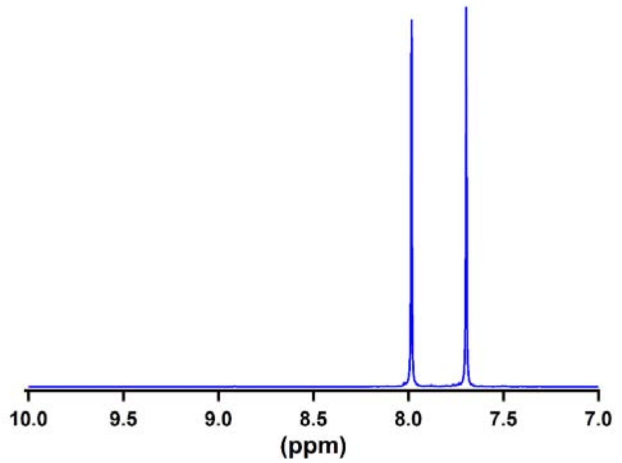

Supplementary Figure 34 | ^{1}H NMR spectrum of E_1 (400 MHz, d_6-DMSO). δ (ppm) 7.98 (s, 4H), 7.69 (s, 4H).

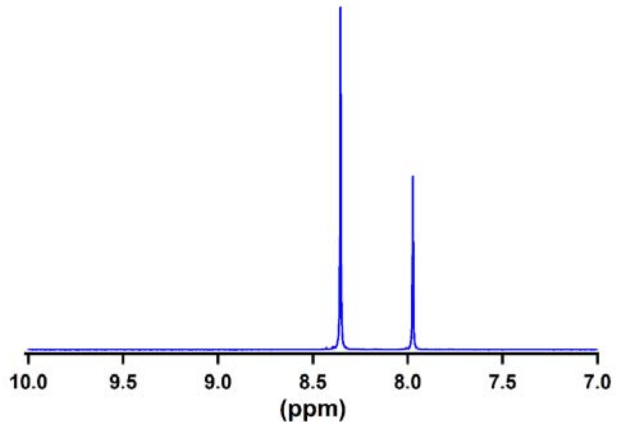

Supplementary Figure 35 | ^{1}H NMR spectrum of E_2 (400 MHz, d_6-DMSO). δ (ppm) 8.35 (s, 4H), 7.97 (s, 2H).

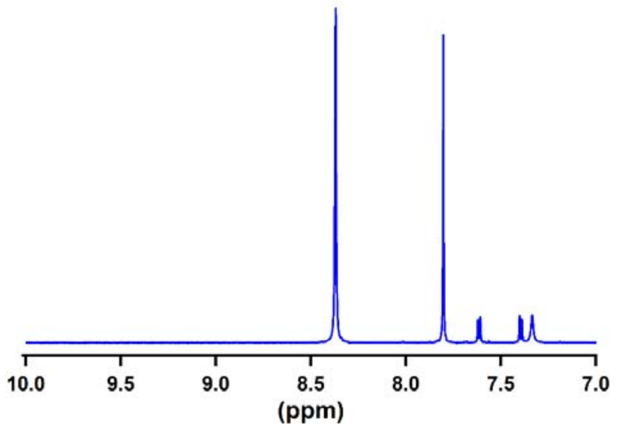

Supplementary Figure 36 | ^{1}H NMR spectrum of E_3 (400 MHz, d_6-DMSO). δ (ppm) 8.37 (s, 4H), 7.80 (s, 2H).

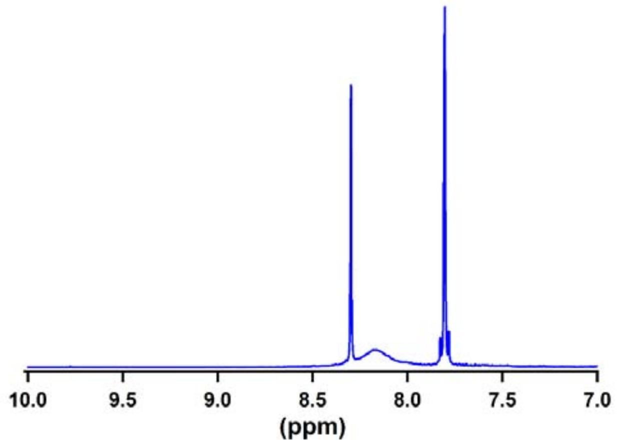

Supplementary Figure 37 | ^{1}H NMR spectrum of E_4 (400 MHz, d_6-DMSO). δ (ppm) 8.30 (s, 2H), 8.18 (s, 4H), 7.80 (t, 4H).

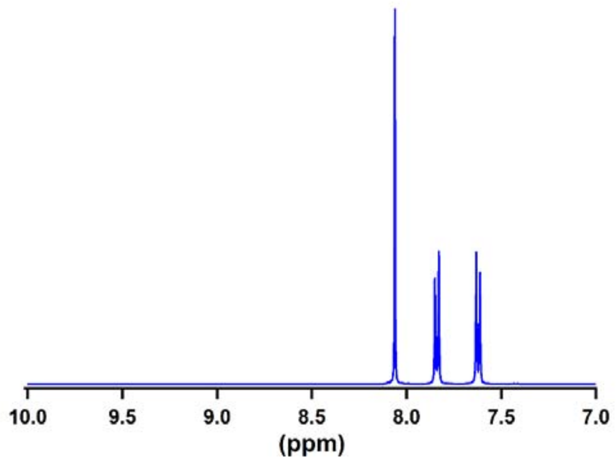

Supplementary Figure 38 | ^{1}H NMR spectrum of E_5 (400 MHz, d_6-DMSO). δ (ppm) 8.06 (s, 4H), 7.83 (d, 4H), 7.62 (d, 4H).

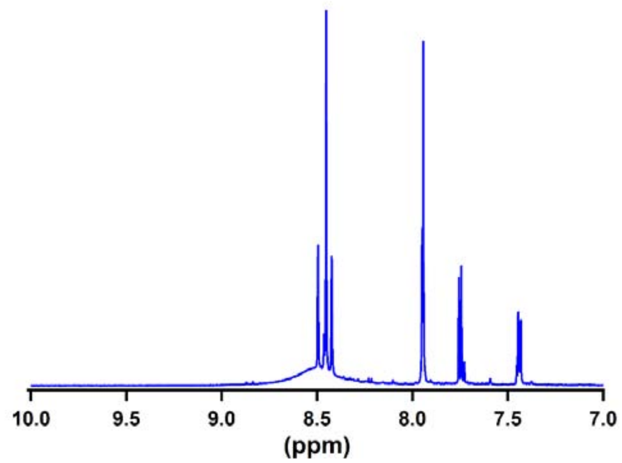

Supplementary Figure 39 | ^{1}H NMR spectrum of E_6 (400 MHz, d_6-DMSO). δ (ppm) 8.50-8.42 (m, 6H), 7.94 (s, 2H).

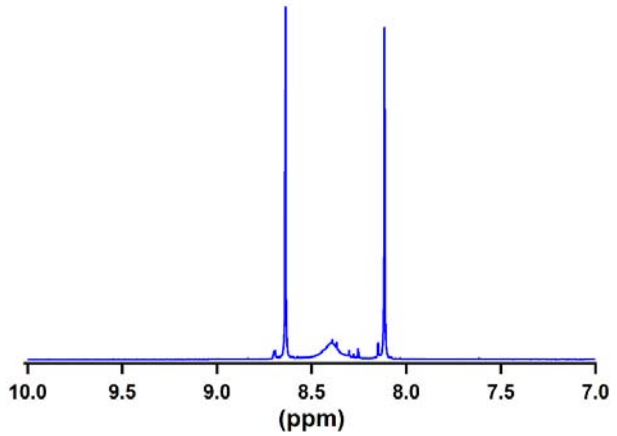

Supplementary Figure 40 | ^{1}H NMR spectrum of E_7 (400 MHz, d_6-DMSO). δ (ppm) 8.64 (s, 4H), 8.39 (s, 4H), 8.11 (s, 4H).

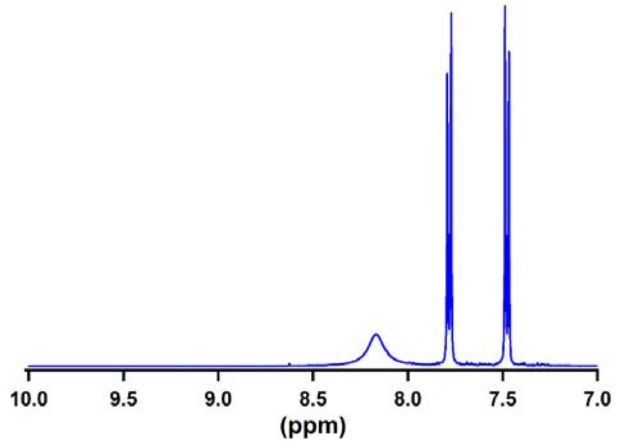

Supplementary Figure 41 | ^{1}H NMR spectrum of E_8 (400 MHz, d_6-DMSO). δ (ppm) 8.17 (s, 4H), 7.78 (s, 4H), 7.47 (s, 4H).

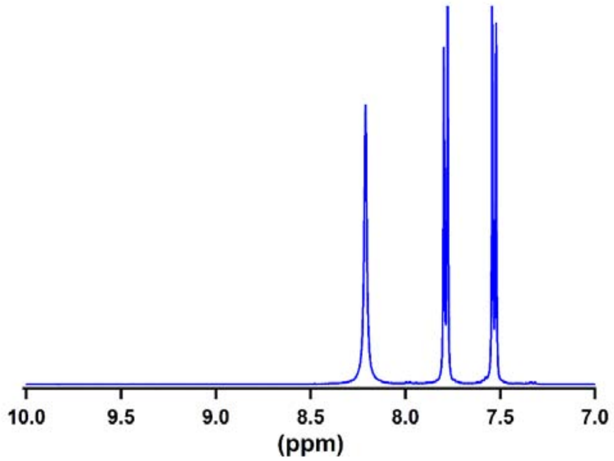

Supplementary Figure 42 | ^{1}H NMR spectrum of E_9 (400 MHz, d_6-DMSO). δ (ppm) 8.21 (s, 4H), 7.78 (d, 4H), 7.53 (d, 4H).

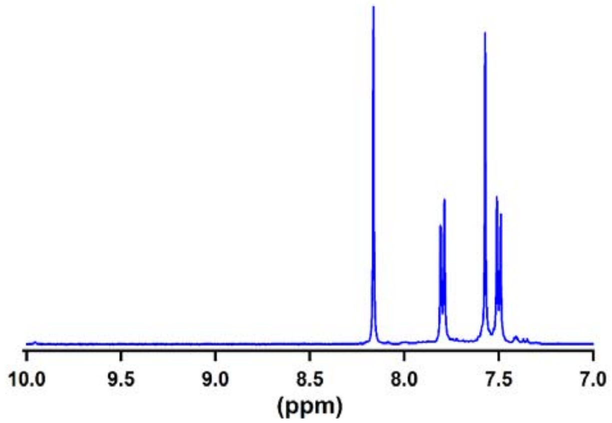

Supplementary Figure 43 | ^{1}H NMR spectrum of E_{10} (400 MHz, d_6-DMSO). δ (ppm) 8.16 (s, 4H), 7.79 (d, 4H), 7.57 (s, 4H), 7.49 (d, 4H).

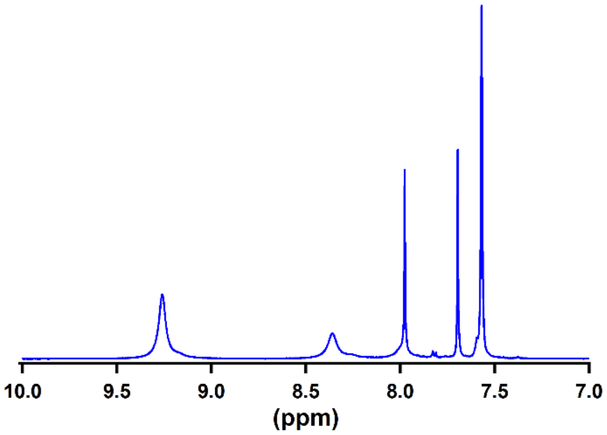

Supplementary Figure 44 | ^{1}H NMR spectrum of the digested MC-COF-TP-$E_1{}^1E_2{}^2$ (400 MHz, d_6-DMSO). δ (ppm) 9.26 (s, 6H), 8.36 (s, 4H), 7.97 (s, 4H), 7.70 (s, 2H), 7.57 (s, 6H). Molar ratio based on integration of the peaks: TP: E_1: E_2 = 1: 0.5: 1.

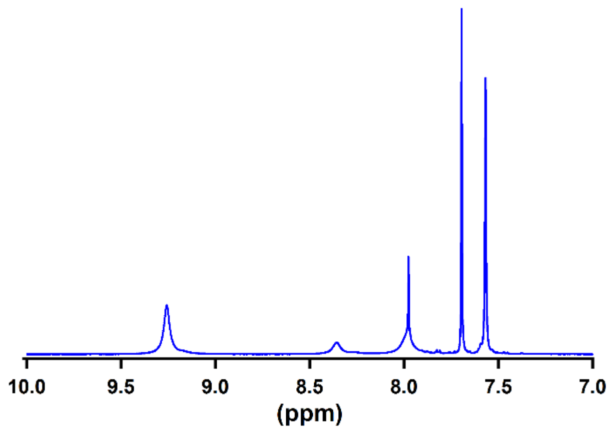

Supplementary Figure 45 | ^{1}H NMR spectrum of the digested MC-COF-TP-$E_1^2E_2^1$ (400 MHz, d_6-DMSO). δ (ppm) 9.26 (s, 6H), 8.36 (s, 2H), 7.97 (s, 5H), 7.70 (s, 4H), 7.57 (s, 6H). Molar ratio based on integration of the peaks: TP: E_1: E_2 = 1: 1: 0.5.

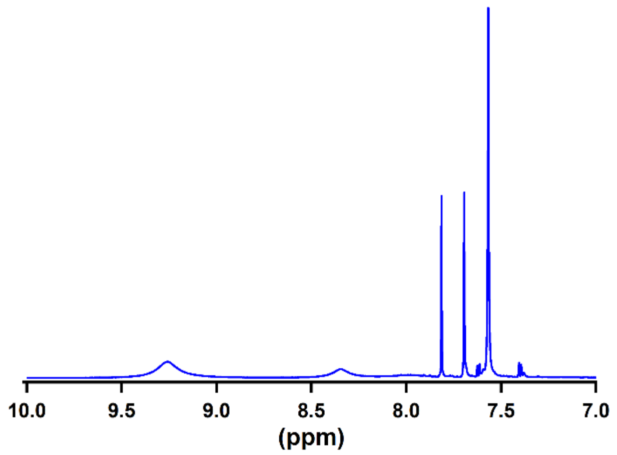

Supplementary Figure 46 | ^{1}H NMR spectrum of the digested MC-COF-TP- $E_1^1E_3^2$ (400 MHz, d_6-DMSO). δ (ppm) 9.26 (s, 6H), 8.36 (s, 2H), 7.81 (s, 2H), 7.70 (s, 2H), 7.57 (s, 6H). Molar ratio based on integration of the peaks: TP: E_1: E_3 = 1: 0.5: 1.

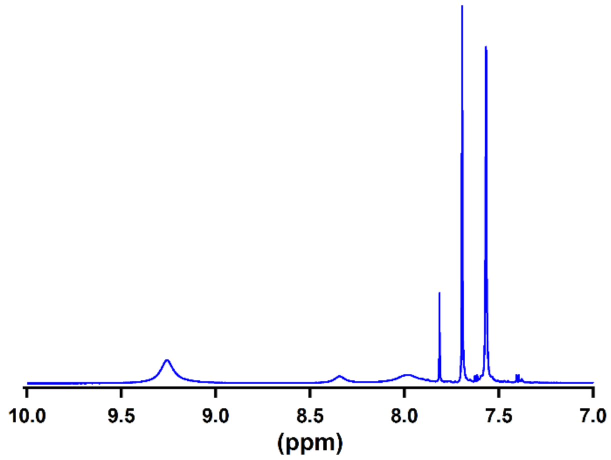

Supplementary Figure 47 | ^{1}H NMR spectrum of ^{1}H NMR of the digested MC-COF-TP- $E_1^2E_3^1$ (400 MHz, d_6-DMSO). δ (ppm) 9.26 (s, 6H), 8.36 (s, 2H), 7.98 (s, 3H), 7.81 (s, 1H), 7.70 (s, 4H), 7.57 (s, 6H). Molar ratio based on integration of the peaks: TP: E_1: E_3 = 1: 1: 0.5.

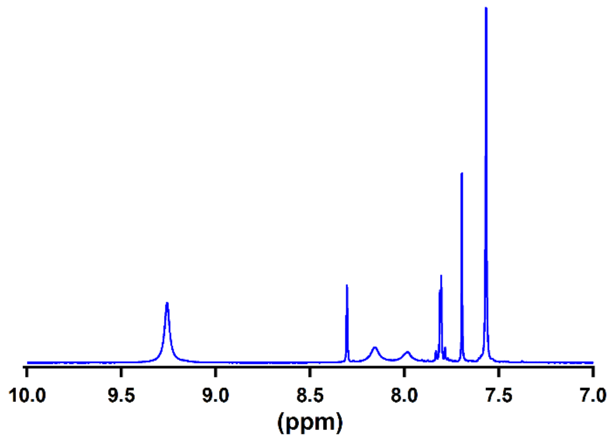

Supplementary Figure 48 | ^{1}H NMR spectrum ^{1}H NMR of the digested MC-COF-TP-$E_1^1E_4^2$ (400 MHz, d_6-DMSO). δ (ppm) 9.26 (s, 6H), 8.30 (s, 1H), 8.16 (s, 2H), 8.00 (s, 2H), 7.81 (s, 2H), 7.69 (s, 2H), 7.57 (s, 6H). Molar ratio based on integration of the peaks: TP: E_1: E_4 = 1: 0.5: 1.

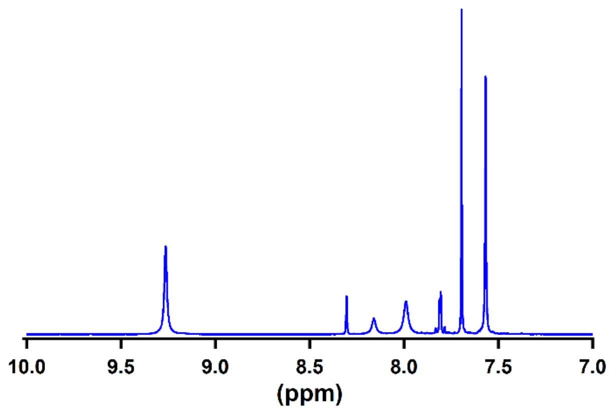

Supplementary Figure 49 | ^{1}H NMR spectrum of the digested MC-COF-TP- $E_1^{2}E_4^{1}$ (400 MHz, d_6-DMSO). δ (ppm) 9.26 (s, 6H), 8.30 (s, 1H), 8.16 (s, 1H), 8.00 (s, 4H), 7.81 (s, 2H), 7.69 (s, 4H), 7.57 (s, 6H). Molar ratio based on integration of the peaks: TP: E_1: E_4 = 1: 1: 0.5.

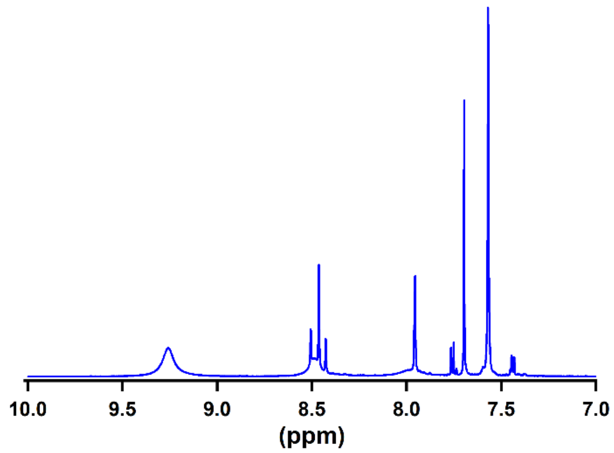

Supplementary Figure 50 | ^{1}H NMR spectrum of the digested MC-COF-TP-$E_1^{1}E_6^{2}$ (400 MHz, d_6-DMSO). δ (ppm) 9.26 (s, 6H), 8.51-8.43 (m, 4H), 7.95 (m, 4H), 7.69 (s, 2H), 7.57 (s, 6H). Molar ratio based on integration of the peaks: TP: E_1: E_6 = 1: 0.5: 1.

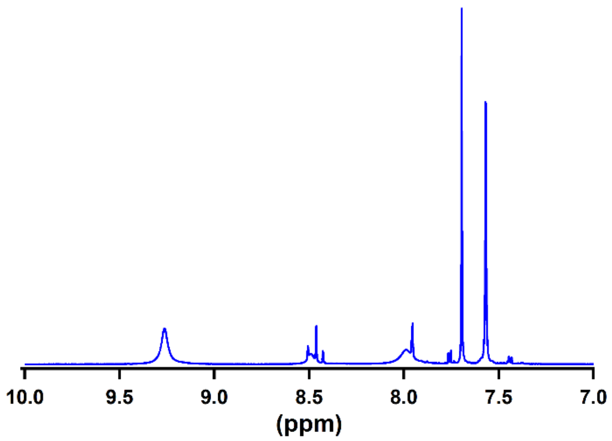

Supplementary Figure 51 | ^{1}H NMR spectrum of the digested MC-COF-TP-$E_1^2E_6^1$ (400 MHz, d_6-DMSO). δ (ppm) 9.26 (s, 6H), 8.51-8.43 (m, 2H), 7.95 (m, 5H), 7.69 (s, 4H), 7.57 (s, 6H). Molar ratio based on integration of the peaks: TP: E_1: E_6 = 1: 1: 0.5.

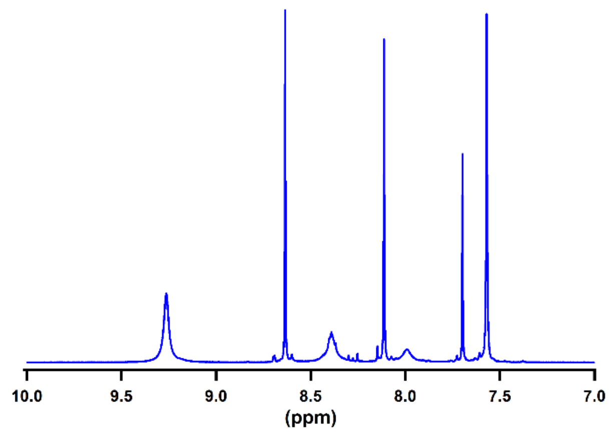

Supplementary Figure 52 | ^{1}H NMR spectrum of the digested MC-COF-TP-$E_1^1E_7^2$ (400 MHz, d_6-DMSO). δ (ppm) 9.26 (s, 6H), 8.63 (s, 4H), 8.38 (s, 4H), 8.11 (s, 4H), 7.99 (s, 2H), 7.69 (s, 2H), 7.57 (s, 6H). Molar ratio based on integration of the peaks: TP: E_1: E_7=1:0.5:1.

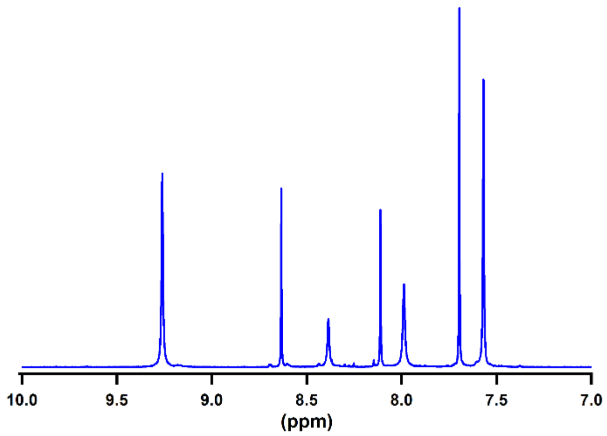

Supplementary Figure 53 | ^{1}H NMR spectrum of the digested MC-COF-TP- $E_1{}^2E_7{}^1$ (400 MHz, d_6-DMSO). δ (ppm) 9.26 (s, 6H), 8.63 (s, 2H), 8.38 (s, 2H), 8.11 (s, 2H), 7.99 (s, 4H), 7.69 (s, 4H), 7.57 (s, 6H). Molar ratio based on integration of the peaks: TP: E_1: E_7=1:1:0.5.

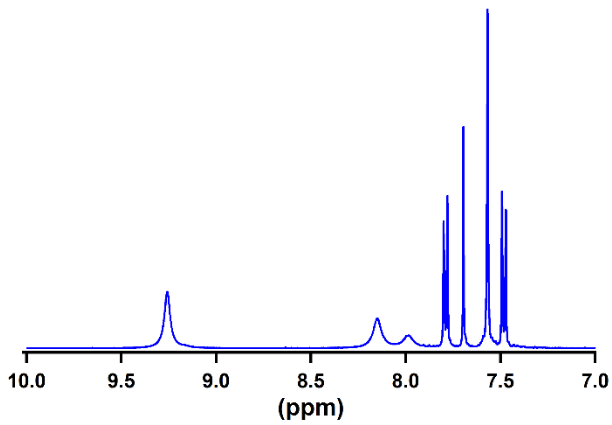

Supplementary Figure 54 | ^{1}H NMR spectrum of the digested MC-COF-TP- $E_1{}^1E_8{}^2$ (400 MHz, d_6-DMSO). δ (ppm) 9.26 (s, 6H), 8.15 (s, 4H), 7.98 (m, 2H), 7.79 (m, 4H), 7.69 (s, 2H), 7.57 (s, 6H), 7.48 (m, 4H). Molar ratio based on integration of the peaks: TP: E_1: E_8 = 1: 0.5: 1.

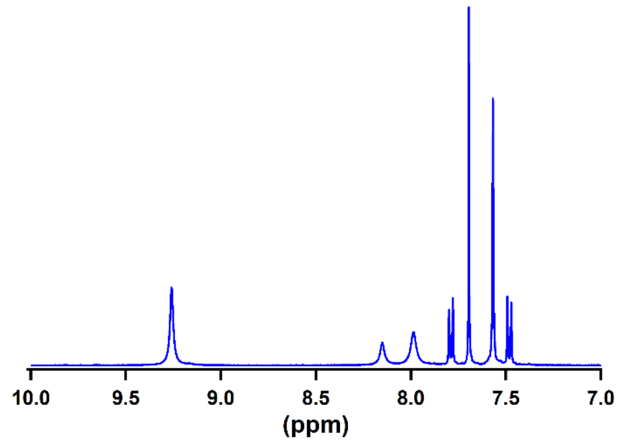

Supplementary Figure 55 | ^{1}H NMR spectrum of the digested MC-COF-TP- $E_1^{2}E_8^{1}$ (400 MHz, d_6-DMSO). δ (ppm) 9.26 (s, 6H), 8.15 (s, 2H), 7.98 (m, 4H), 7.79 (d, 2H), 7.69 (s, 4H), 7.57 (s, 6H), 7.48 (m, 4H). Molar ratio based on integration of the peaks: TP: E_1: E_8 = 1: 1: 0.5.

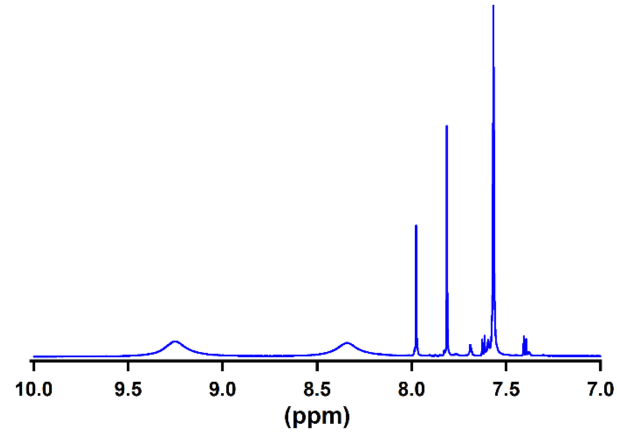

Supplementary Figure 56 | ^{1}H NMR spectrum of the digested MC-COF-TP-$E_2^{1}E_3^{2}$ (400 MHz, d_6-DMSO). δ (ppm) 9.26 (s, 6H), 8.35 (s, 6H), 7.97 (s, 1H), 7.81 (s, 2H), 7.57 (s, 6H). Molar ratio based on integration of the peaks: TP: E_2: E_3 =1: 0.5: 1.

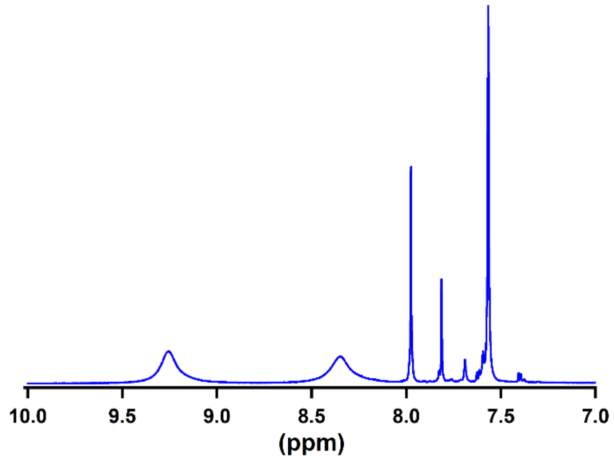

Supplementary Figure 57 | ^{1}H NMR spectrum of the digested MC-COF-TP-$E_2^{2}E_3^{1}$ (400 MHz, d_6-DMSO). δ (ppm) 9.26 (s, 6H), 8.35 (s, 6H), 7.97 (s, 2H), 7.81 (s, 1H), 7.57 (s, 6H). Molar ratio based on integration of the peaks: TP: E_2: E_3 =1: 1: 0.5.

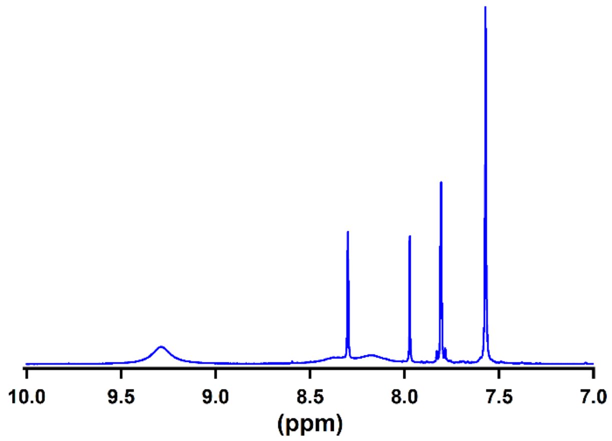

Supplementary Figure 58 | ^{1}H NMR spectrum of the digested MC-COF-TP-$E_2^{1}E_4^{2}$ (400 MHz, d_6-DMSO). δ (ppm) 9.26 (s, 6H), 8.35-7.99 (s, 6H), 7.97 (s, 1H), 7.80 (t, 4H), 7.57 (s, 6H). Molar ratio based on integration of the peaks: TP: E_2: E_4 =1: 0.5: 1.

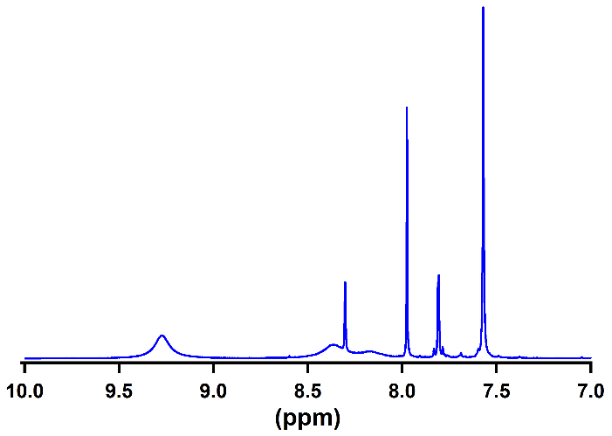

Supplementary Figure 59 | ^{1}H NMR spectrum of the digested MC-COF-TP-$E_2^2E_4^1$ (400 MHz, d_6-DMSO). δ (ppm) 9.26 (s, 6H), 8.35-7.99 (s, 6H), 7.97 (s, 2H), 7.80 (t, 2H), 7.57 (s, 6H). Molar ratio based on integration of the peaks: TP: E_2: E_4 =1: 1: 0.5.

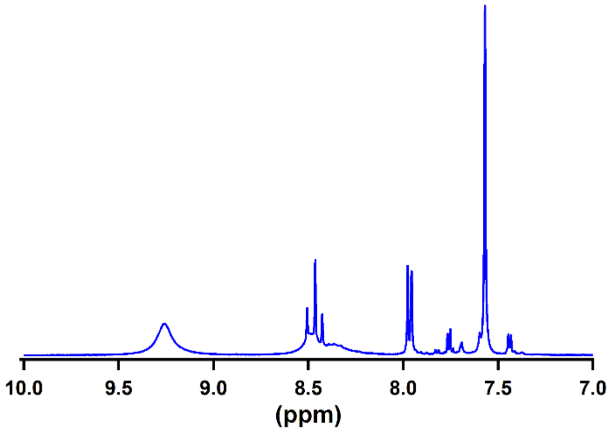

Supplementary Figure 60 | ^{1}H NMR spectrum of the digested MC-COF-TP- $E_2^1E_6^2$ (400 MHz, d_6-DMSO). δ (ppm) 9.26 (s, 6H), 8.50-8.33 (m, 8H), 7.96 (d, 3H), 7.57 (s, 6H). Molar ratio based on integration of the peaks: TP: E_2: E_6 =1: 0.5: 1.

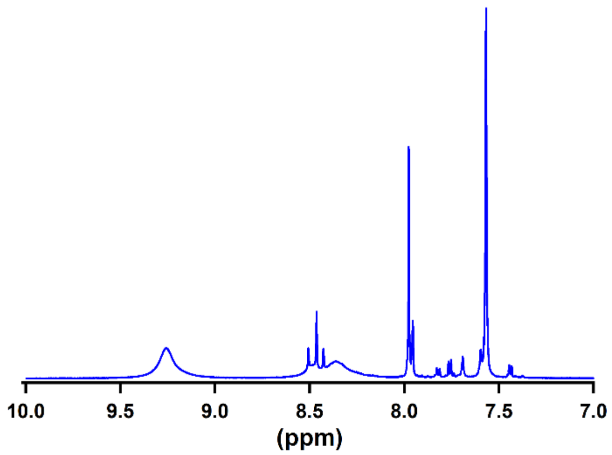

Supplementary Figure 61 | ^{1}H NMR spectrum of the digested MC-COF-TP- $E_2{}^2E_6{}^1$ (400 MHz, d_6-DMSO). δ (ppm) 9.26 (s, 6H), 8.50-8.33 (m, 7H), 7.96 (d, 3H), 7.57 (s, 6H). Molar ratio based on integration of the peaks: TP: E_2: E_6 =1: 1: 0.5.

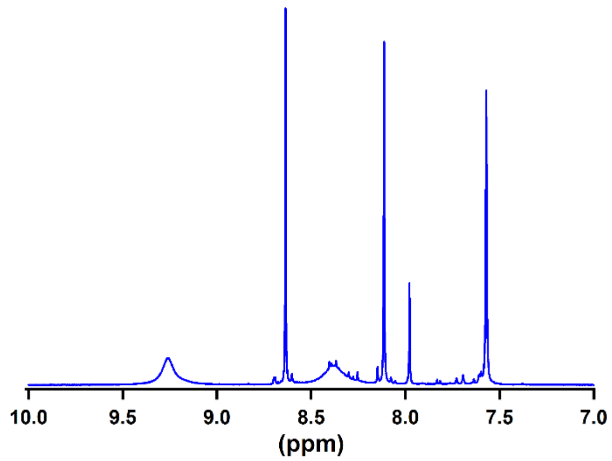

Supplementary Figure 62 | ^{1}H NMR spectrum of the digested MC-COF-TP- $E_2{}^1E_7{}^2$ (400 MHz, d_6-DMSO). δ (ppm) 9.26 (s, 6H), 8.63 (s, 4H), 8.39 (s, 4H), 8.09 (s, 4H), 7.98 (s, 1H), 7.57 (s, 6H). Molar ratio based on integration of the peaks: TP: E_2: E_7 =1: 0.5: 1.

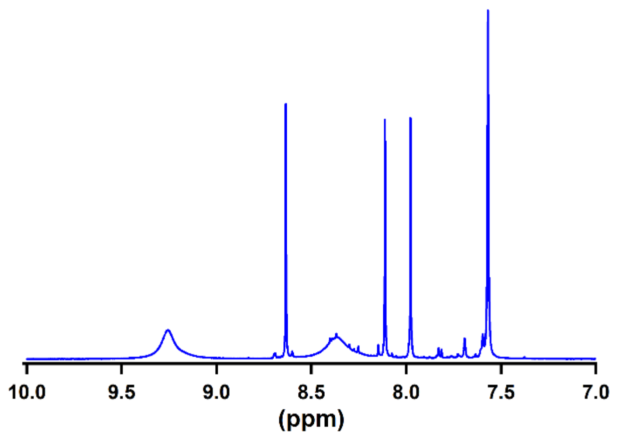

Supplementary Figure 63 | ^{1}H NMR spectrum of the digested MC-COF-TP- $E_2{}^2E_7{}^1$ (400 MHz, d_6-DMSO). δ (ppm)9.26 (s, 6H), 8.63 (s, 2H), 8.39 (s, 6H), 8.09 (s, 2H), 7.98 (s, 2H), 7.57 (s, 6H). Molar ratio based on integration of the peaks: TP: E_2: E_7 =1: 1: 0.5.

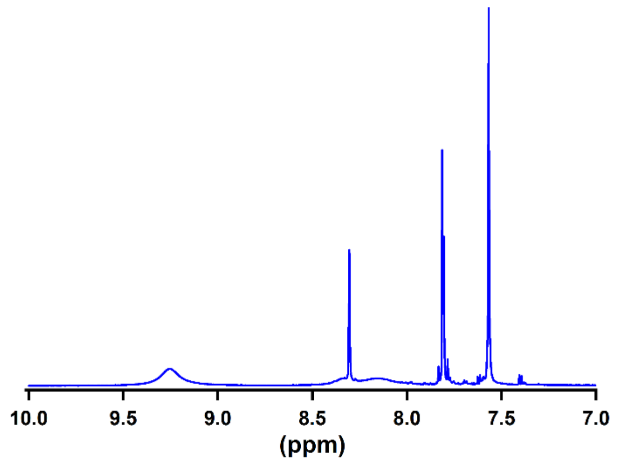

Supplementary Figure 64 | ^{1}H NMR spectrum of the digested MC-COF-TP-$E_3{}^1E_4{}^2$ (400 MHz, d_6-DMSO). δ (ppm) 9.26 (s, 6H), 8.35-8.18 (m, 6H), 7.78 (s, 6H), 7.57 (s, 6H). Molar ratio based on integration of the peaks: TP: E_3: E_4 =1: 0.5: 1.

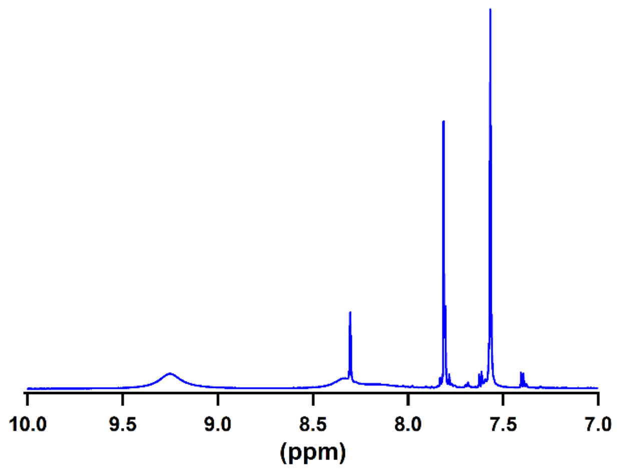

Supplementary Figure 65 | ^{1}H NMR spectrum of the digested MC-COF-TP-$E_3^2E_4^1$ (400 MHz, d_6-DMSO). δ (ppm) 9.26 (s, 6H), 8.35-8.18 (m, 6H), 7.78 (s, 4H), 7.57 (s, 6H). Molar ratio based on integration of the peaks: TP: E_3: E_4 =1: 1: 0.5.

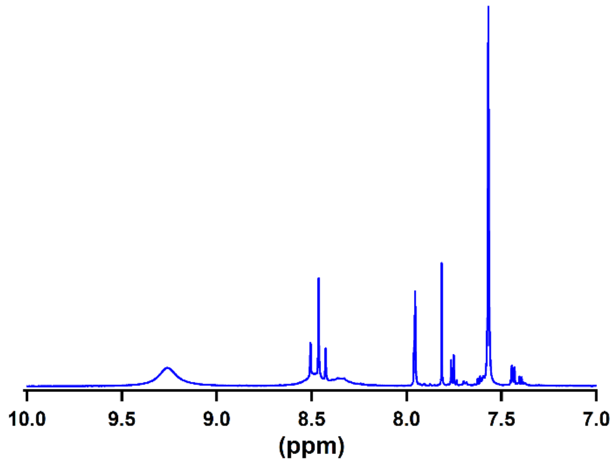

Supplementary Figure 66 | ^{1}H NMR spectrum of the digested MC-COF-TP-$E_3^1E_6^2$ (400 MHz, d_6-DMSO). δ (ppm) 9.26 (s, 6H), 8.35-8.18 (m, 8H), 7.95 (s, 2H), 7.81 (s, 1H), 7.57 (s, 6H). Molar ratio based on integration of the peaks: TP: E_3: E_6 =1: 0.5: 1.

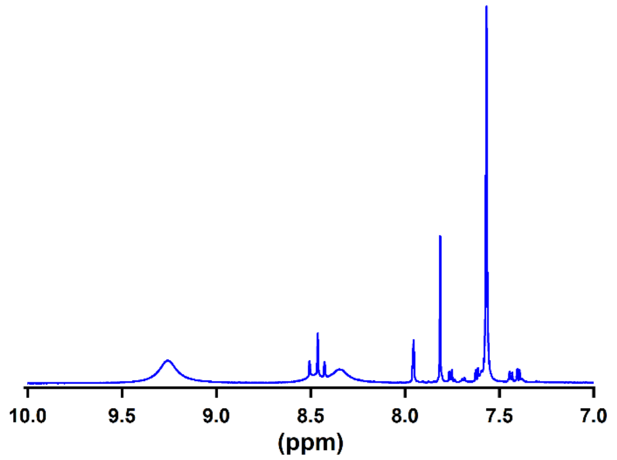

Supplementary Figure 67 | ^{1}H NMR spectrum of the digested MC-COF-TP-$E_3^2E_6^1$ (400 MHz, d_6-DMSO). δ (ppm) 9.26 (s, 6H), 8.35-8.18 (m, 7H), 7.95 (s, 1H), 7.81 (s, 2H), 7.57 (s, 6H). Molar ratio based on integration of the peaks: TP: E_3: E_6 =1: 1: 0.5.

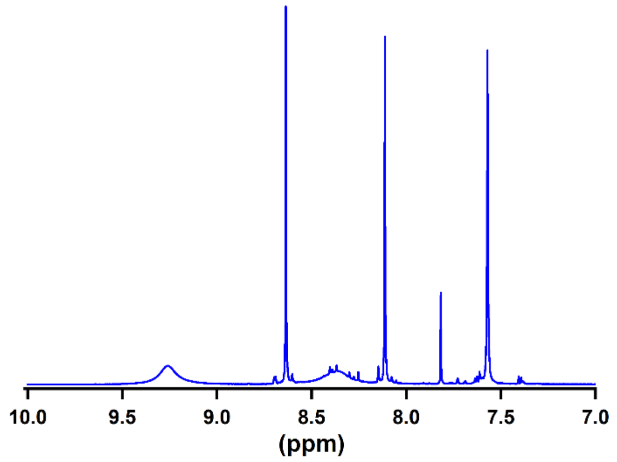

Supplementary Figure 68 | ^{1}H NMR spectrum of the digested MC-COF-TP-$E_3^1E_7^2$ (400 MHz, d_6-DMSO). δ (ppm) 9.26 (s, 6H), 8.60 (s, 4H), 8.40-8.25 (m, 6H), 8.11 (s, 4H), 7.81 (s, 1H), 7.57 (s, 6H). Molar ratio based on integration of the peaks: TP: E_3: E_7 =1: 0.5: 1.

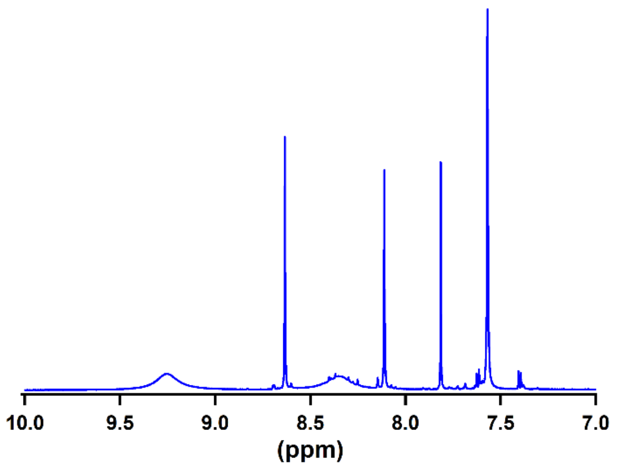

Supplementary Figure 69 | ^{1}H NMR spectrum of the digested MC-COF-TP-$E_3^2E_7^1$ (400 MHz, d_6-DMSO). δ (ppm) 9.26 (s, 6H), 8.60 (s, 2H), 8.40-8.25 (m, 6H), 8.11 (s, 2H), 7.81 (s, 2H), 7.57 (s, 6H). Molar ratio based on integration of the peaks: TP: E_3: E_7 =1: 1: 0.5.

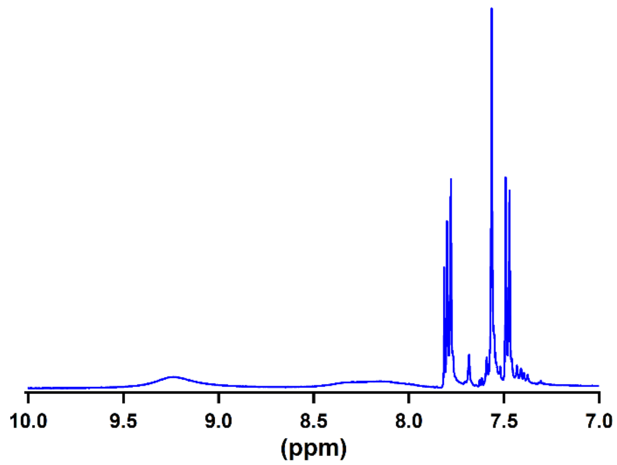

Supplementary Figure 70 | ^{1}H NMR spectrum of the digested MC-COF-TP-$E_3^1E_8^2$ (400 MHz, d_6-DMSO). δ (ppm) 9.26 (s, 6H), 8.47-7.82 (b, 6H), 7.81-7.78 (m, 4H), 7.57-7.50 (m, 8H), 7.49 (d, 2H). Molar ratio based on integration of the peaks: TP: E_3: E_8 =1: 0.5: 1.

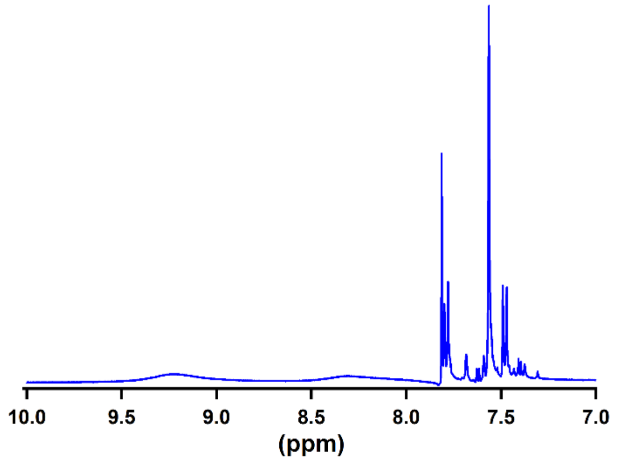

Supplementary Figure 71 | ^{1}H NMR spectrum of the digested MC-COF-TP-$E_3{}^2E_8{}^1$ (400 MHz, d_6-DMSO). δ (ppm) 9.26 (s, 6H), 8.47-7.82 (b, 6H), 7.81-7.78 (m, 6H), 8.11 (s, 2H), 7.81 (s, 2H), 7.57 (s, 6H). Molar ratio based on integration of the peaks: TP: E_3: E_8 =1: 1: 0.5.

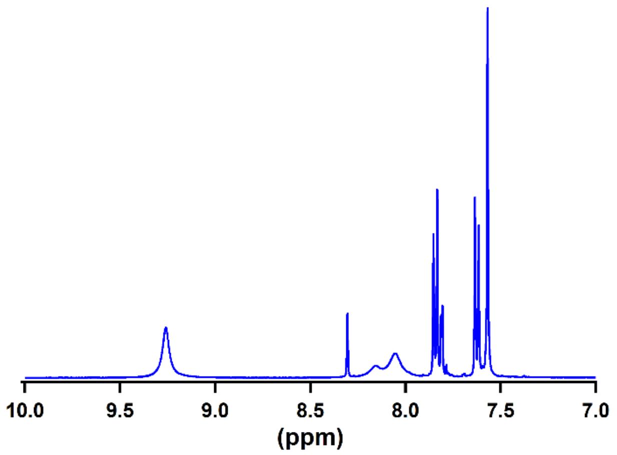

Supplementary Figure 72 | ^{1}H NMR spectrum of the digested MC-COF-TP-$E_4{}^1E_5{}^2$ (400 MHz, d_6-DMSO). δ (ppm) 9.26 (s, 6H), 8.30 (s, 1H), 8.20-7.94 (m, 6H), 7.85-7.80 (m, 6H), 7.63-7.57 (m, 10H). Molar ratio based on integration of the peaks: TP: E_4: E_5 =1: 0.5: 1.

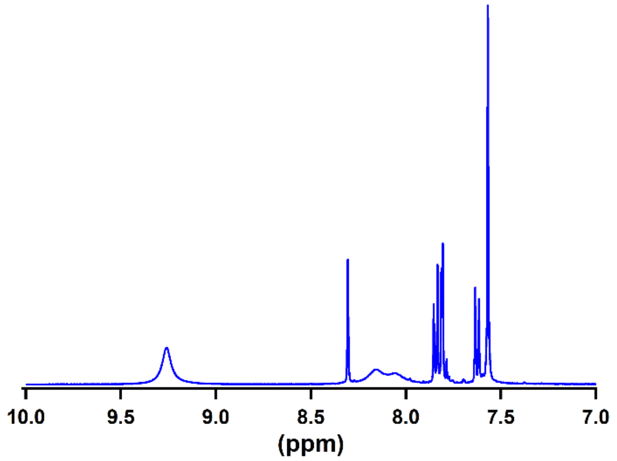

Supplementary Figure 73 | ^{1}H NMR spectrum of the digested MC-COF-TP-$E_4^{1}E_5^{2}$ (400 MHz, d_6-DMSO). δ (ppm) 9.26 (s, 6H), 8.30 (s, 2H), 8.20-7.94 (m, 6H), 7.85-7.80 (m, 6H), 7.63-7.57 (m, 8H). Molar ratio based on integration of the peaks: TP: E_4: E_5 =1: 1: 0.5.

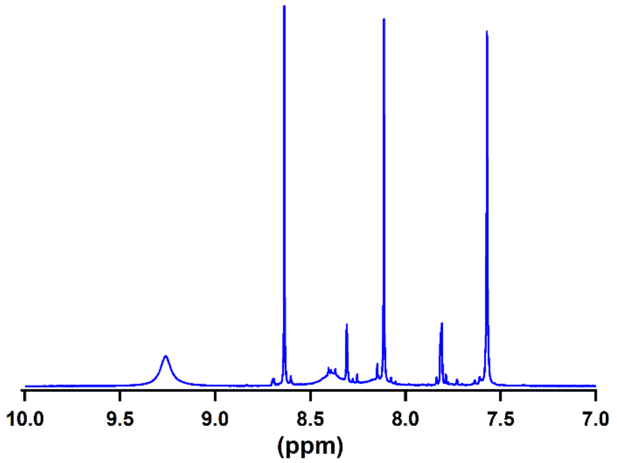

Supplementary Figure 74 | ^{1}H NMR spectrum of the digested MC-COF-TP- $E_4^{1}E_7^{2}$ (400 MHz, d_6-DMSO). δ (ppm) 9.26 (s, 6H), 8.65 (s, 4H), 8.43-8.25 (m, 5H), 8.15 (s, 6H), 7.81 (s, 2H), 7.57 (m, 6H). Molar ratio based on integration of the peaks: TP: E_4: E_7 =1: 0.5: 1.

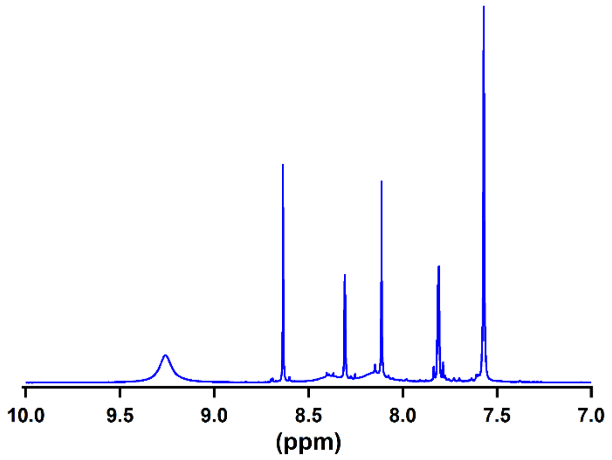

Supplementary Figure 75 | ^{1}H NMR spectrum of the digested MC-COF-TP- $E_4{}^1E_7{}^2$ (400 MHz, Dd_6-DMSO). δ (ppm) 9.26 (s, 6H), 8.65 (s, 2H), 8.43-8.25 (m, 4H), 8.15 (s, 5H), 7.81 (s, 4H), 7.57 (m, 6H). Molar ratio based on integration of the peaks: TP: E_4: E_7 =1: 1: 0.5.

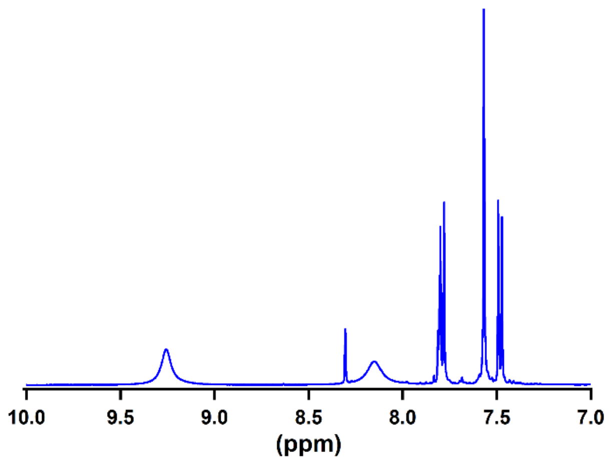

Supplementary Figure 76 | ^{1}H NMR spectrum of the digested MC-COF-TP-$E_4{}^1E_8{}^2$ (400 MHz, d_6-DMSO). δ (ppm) 9.26 (s, 6H), 8.32-8.12 (m, 6H), 7.80 (m, 6H), 7.57 (m, 6H), 7.48 (m, 4H). Molar ratio based on integration of the peaks: TP: E_4: E_8 =1: 0.5: 1.

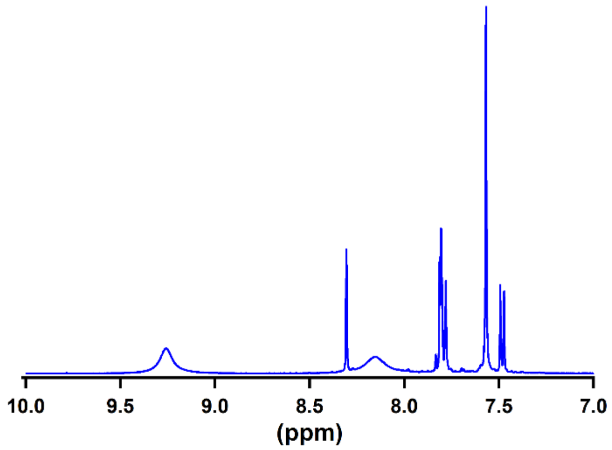

Supplementary Figure 77 | ^{1}H NMR spectrum of the digested MC-COF-TP-$E_4{}^1E_8{}^2$ (400 MHz, d_6-DMSO). δ (ppm) 9.26 (s, 6H), 8.32-8.12 (m, 8H), 7.80 (m, 6H), 7.57 (m, 6H), 7.48 (m, 2H). Molar ratio based on integration of the peaks: TP: E_4: E_8 =1: 1: 0.5.

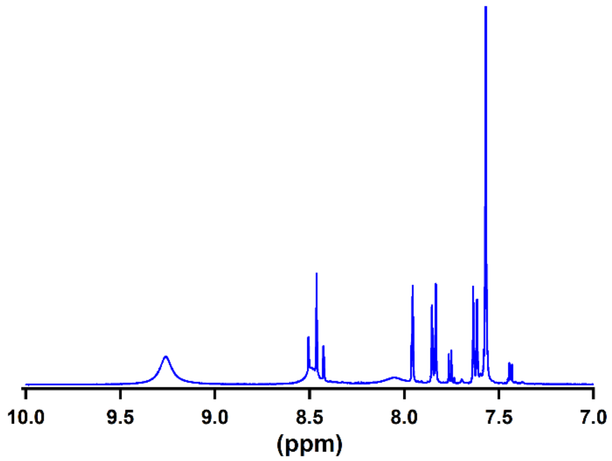

Supplementary Figure 78 | ^{1}H NMR spectrum of the digested MC-COF-TP-$E_5{}^1E_6{}^2$ (400 MHz, d_6-DMSO). δ (ppm) 9.26 (s, 6H), 8.50-8.42 (m, 6H), 8.10-7.95 (m, 4H), 7.84 (d, 2H), 7.63-7.57 (m, 8H). Molar ratio based on integration of the peaks: TP: E_5: E_6 =1: 0.5: 1.

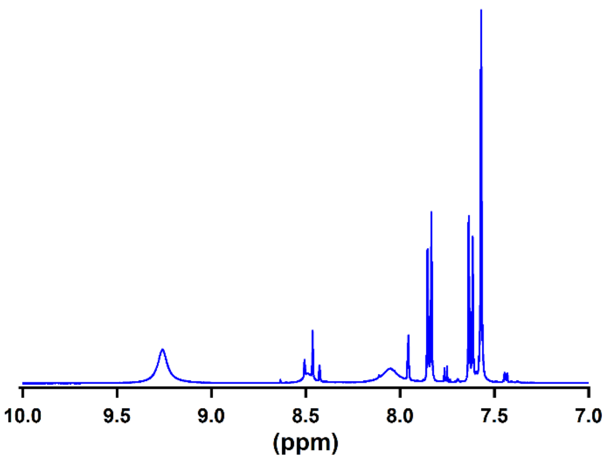

Supplementary Figure 79 | ^{1}H NMR spectrum of the digested MC-COF-TP-$E_5{}^1E_6{}^2$ (400 MHz, d_6-DMSO). δ (ppm) 9.26 (s, 6H), 8.50-8.42 (m, 3H), 8.10-7.95 (m, 5H), 7.84 (d, 4H), 7.63-7.57 (m, 10H). Molar ratio based on integration of the peaks: TP: E_5: E_6 =1: 1: 0.5.

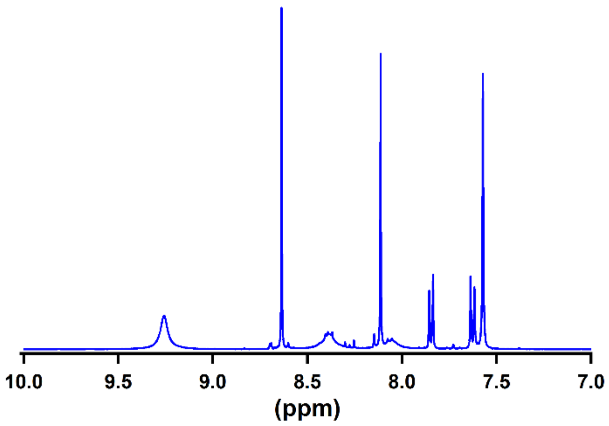

Supplementary Figure 80 | ^{1}H NMR spectrum of the digested MC-COF-TP-$E_5{}^1E_7{}^2$ (400 MHz, d_6-DMSO). δ (ppm) 9.26 (s, 6H), 8.64 (s, 4H), 8.39 (s, 4H), 8.15-8.05 (m, 6H), 7.84 (d, 2H), 7.64-7.57 (m, 8H). Molar ratio based on integration of the peaks: TP: E_5: E_7 =1: 0.5: 1.

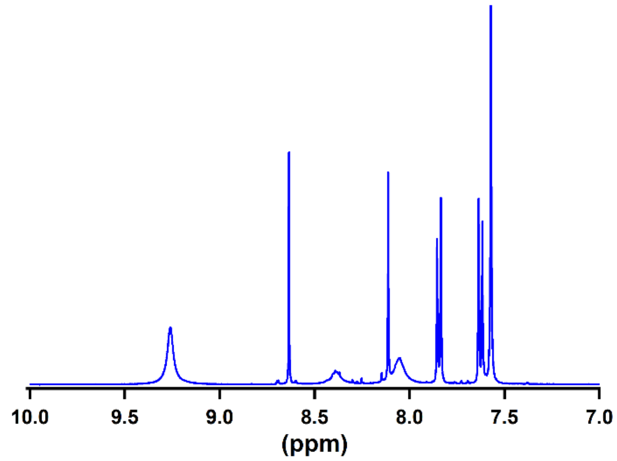

Supplementary Figure 81 | ^{1}H NMR spectrum of the digested MC-COF-TP-$E_5^1E_7^2$ (400 MHz, d_6-DMSO). δ (ppm) 9.26 (s, 6H), 8.64 (s, 2H), 8.39 (s, 2H), 8.15-8.05 (m, 6H), 7.84 (d, 4H), 7.64-7.57 (m, 10H). Molar ratio based on integration of the peaks: TP: E_5: E_7 =1: 1: 0.5.

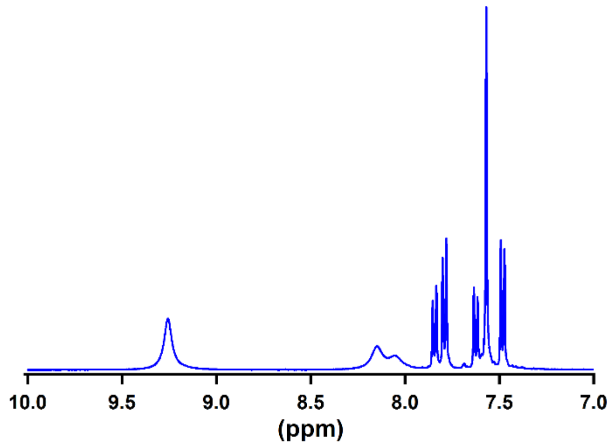

Supplementary Figure 82 | ^{1}H NMR spectrum of the digested MC-COF-TP-$E_5^1E_8^2$ (400 MHz, d_6-DMSO). δ (ppm) 9.26 (s, 6H), 8.15-8.05 (m, 6H), 7.85-7.78 (m, 6H), 7.63-7.57 (m, 8H), 7.48 (d, 4H). Molar ratio based on integration of the peaks: TP: E_5: E_8 =1: 0.5: 1.

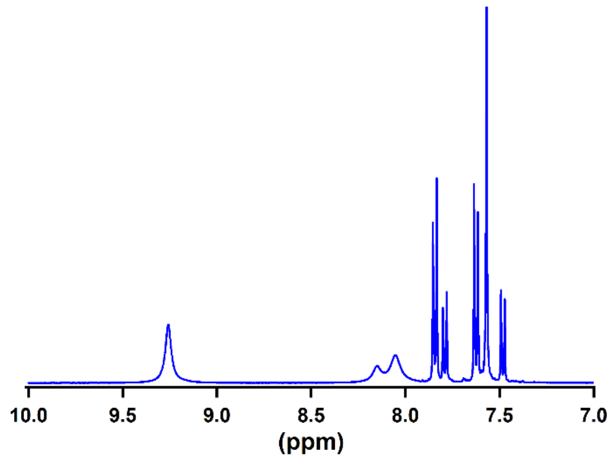

Supplementary Figure 83 | ^{1}H NMR spectrum of the digested MC-COF-TP-$E_5{}^1E_8{}^2$ (400 MHz, d_6-DMSO). δ (ppm) 9.26 (s, 6H), 8.15-8.05 (m, 6H), 7.85-7.78 (m, 6H), 7.63-7.57 (m, 10H), 7.48 (d, 2H). Molar ratio based on integration of the peaks: TP: E_5: E_8 =1: 1: 0.5.

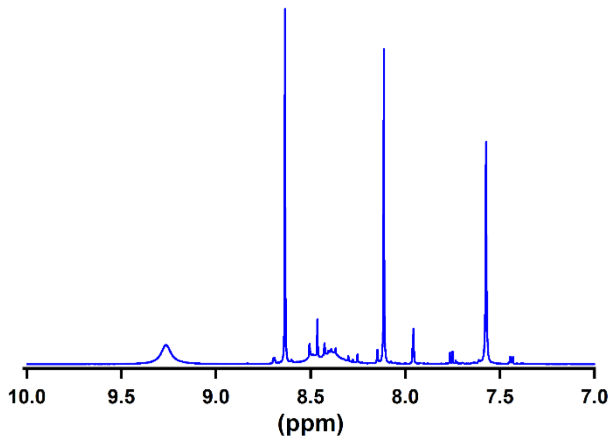

Supplementary Figure 84 | ^{1}H NMR spectrum of the digested MC-COF-TP-$E_6{}^1E_7{}^2$ (400 MHz, d_6-DMSO). δ (ppm) 9.26 (s, 6H), 8.62 (s, 4H), 8.51-8.37 (m, 7H), 8.11 (s, 4H), 7.95 (s, 1H), 7.57 (s, 6H). Molar ratio based on integration of the peaks: TP: E_6: E_7 =1: 0.5: 1.

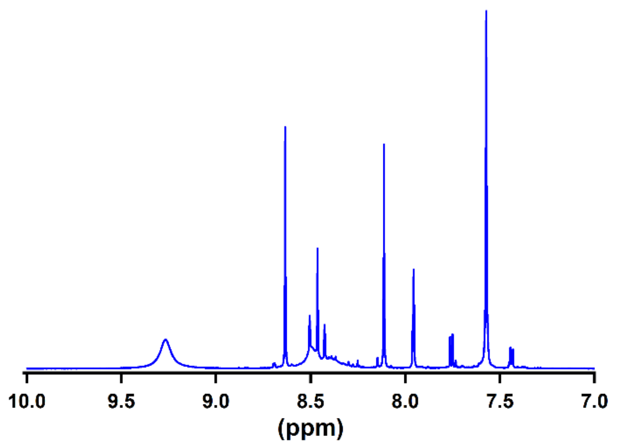

Supplementary Figure 85 | ^{1}H NMR spectrum of the digested MC-COF-TP-$E_6{}^1E_7{}^2$ (400 MHz, d_6-DMSO). δ (ppm) 9.26 (s, 6H), 8.62 (s, 2H), 8.51-8.37 (m, 6H), 8.11 (s, 2H), 7.95 (s, 2H), 7.57 (s, 6H). Molar ratio based on integration of the peaks: TP: E_6: E_7 =1: 1: 0.5.

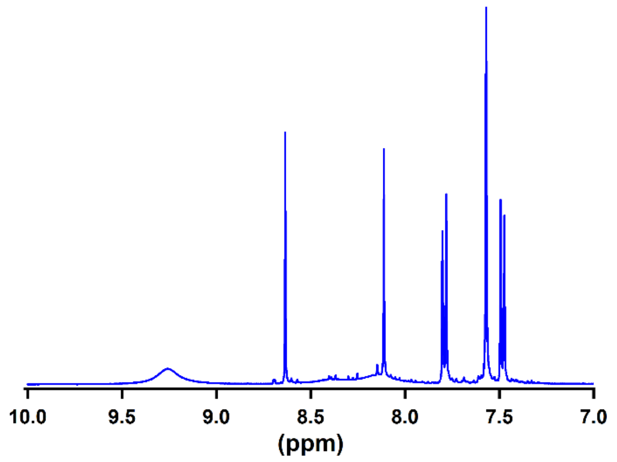

Supplementary Figure 86 | ^{1}H NMR spectrum of the digested MC-COF-TP-$E_7{}^1E_8{}^2$ (400 MHz, d_6-DMSO). δ (ppm) 9.26 (s, 6H), 8.62 (s, 4H), 8.40-8.07 (m, 8H), 7.79 (s, 4H), 7.57 (s, 6H), 7.48 (d, 4H). Molar ratio based on integration of the peaks: TP: E_7: E_8 =1: 0.5: 1.

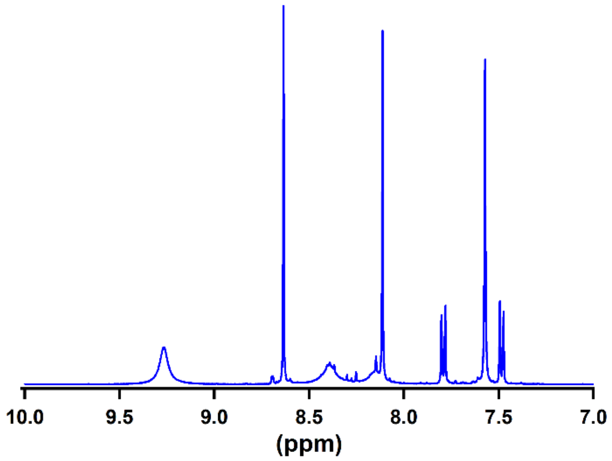

Supplementary Figure 87 | ^{1}H NMR spectrum of the digested MC-COF-TP-$E_7{}^1E_8{}^2$ (400 MHz, d_6-DMSO). δ (ppm) 9.26 (s, 6H), 8.62 (s, 4H), 8.40-8.07 (m, 10H), 7.79 (s, 2H), 7.57 (s, 6H), 7.48 (d, 2H). Molar ratio based on integration of the peaks: TP: E_7: E_8 =1: 1: 0.5.

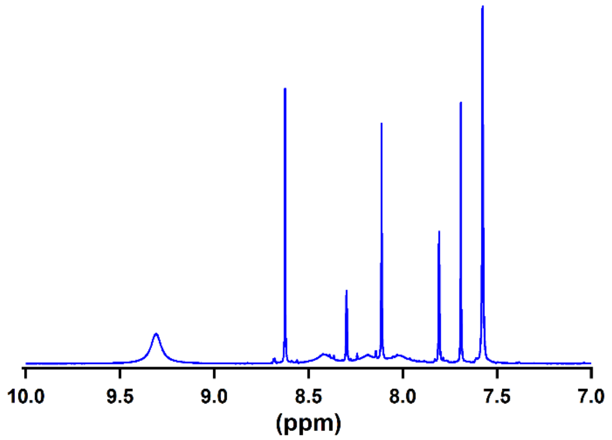

Supplementary Figure 88 | ^{1}H NMR spectrum of the digested MC-COF-TP-$E_1E_4E_7$ (400 MHz, d_6-DMSO). δ (ppm) 9.31 (s, 6H), 8.62 (d, 2H), 8.42 (s, 2H), 8.30 (s, 2H), 8.18 (s, 2H), 8.11 (s, 2H), 8.03 (s, 2H), 7.81 (s, 2H), 7.69 (s, 2H), 7.57 (s, 6H). Molar ratio based on integration of the peaks: TP: E_1: E_4: E_7 =1: 0.5: 0.5: 0.5.

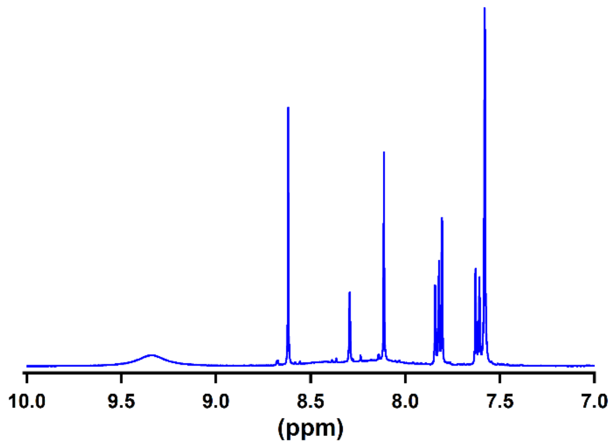

Supplementary Figure 89 | ^{1}H NMR spectrum of the digested MC-COF-TP-$E_4E_5E_8$ (400 MHz, d_6-DMSO). δ (ppm) 9.31 (s, 6H), 8.62 (d, 2H), 8.29-8.11 (m, 6H), 7.82 (t, 4H), 7.62-7.58 (m, 8H). Molar ratio based on integration of the peaks: TP: E_4: E_5: E_7 = 1: 0.5: 0.5: 0.5.

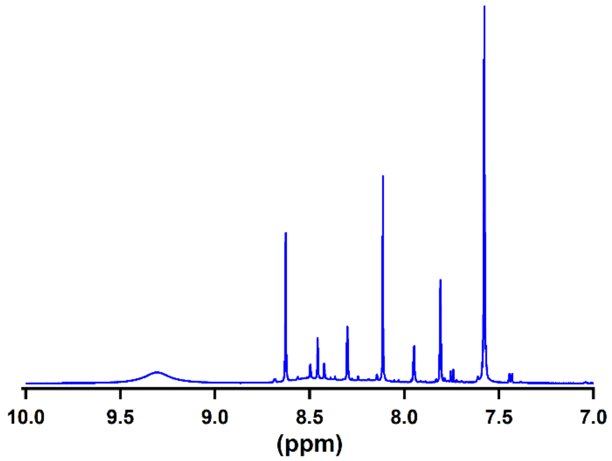

Supplementary Figure 90 | ^{1}H NMR spectrum of the digested MC-COF-TP- $E_4E_6E_7$ (400 MHz, d_6-DMSO). δ (ppm) 9.30 (s, 6H), 8.62 (s, 2H), 8.56-8.36 (m, 4H), 8.30 (s, 2H), 8.11 (s, 3H), 7.94 (s, 1H), 7.81 (s, 2H), 7.58 (s, 6H). Molar ratio based on integration of the peaks: TP: E_4: E_6: E_7 = 1: 0.5: 0.5: 0.5.

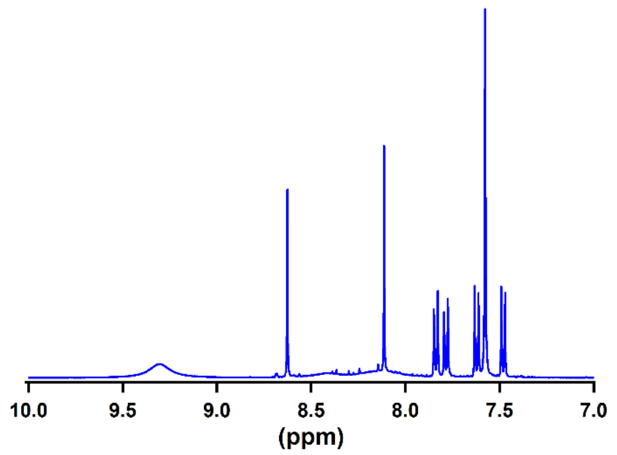

Supplementary Figure 91 | ^{1}H NMR spectrum of the digested MC-COF-TP- $E_5E_7E_8$ (400 MHz, d_6-DMSO). δ (ppm) 9.30 (s, 6H), 8.62 (s, 2H), 8.42-8.14 (m, 7H), 7.83 (d, 2H), 7.98 (d, 2H), 7.62 (d, 2H), 7.57 (s, 6H), 7.48 (d, 2H). Molar ratio based on integration of the peaks: TP: E_5: E_7: E_8 = 1: 0.5: 0.5: 0.5.

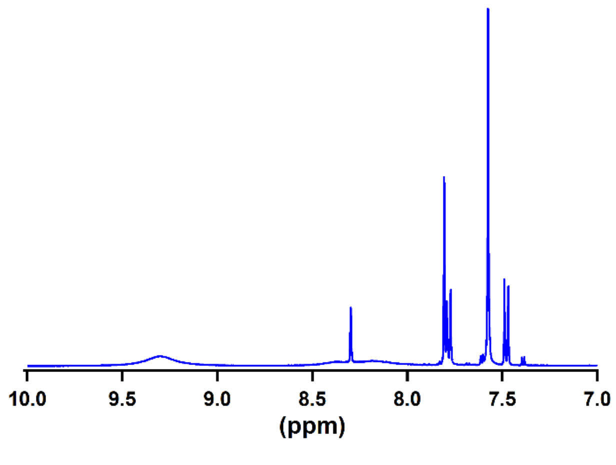

Supplementary Figure 92 | ^{1}H NMR spectrum of the digested MC-COF-TP- $E_3E_4E_8$ (400 MHz, d_6-DMSO). δ (ppm) 9.30 (s, 6H), 8.30-8.18 (m, 5H), 7.81-7.77 (m, 6H), 7.57 (s, 6H), 7.47 (d, 2H). Molar ratio based on integration of the peaks: TP: E_3: E_4: E_8 = 1: 0.5: 0.5: 0.5.

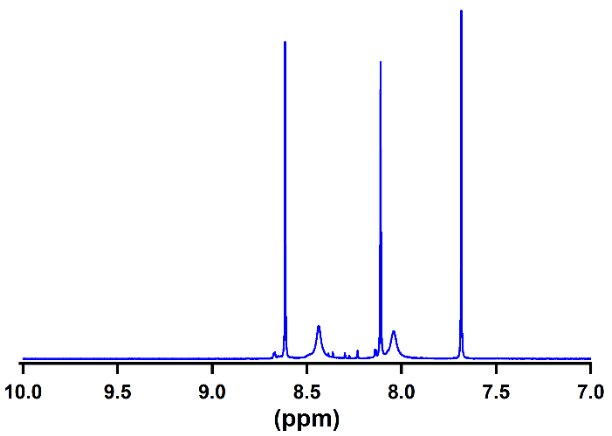

Supplementary Figure 93 | ^{1}H NMR spectrum of the digested MC-COF-NiPc-E_1E_7 (400 MHz, d_6-DMSO). δ (ppm) 8.61 (s, 4H), 8.43 (s, 4H), 8.11 (s, 4H), 8.04 (s, 4H), 7.68 (s, 4H). Molar ratio based on integration of the peaks: E_1: E_7=1: 1.

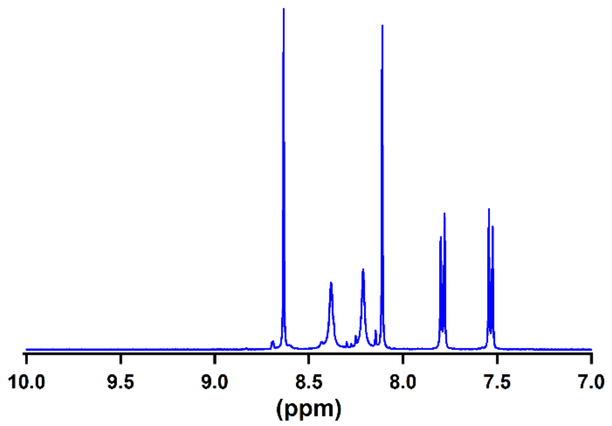

Supplementary Figure 94 | ^{1}H NMR spectrum of the digested MC-COF-NiPc-E_7E_9 (400 MHz, d_6-DMSO). δ (ppm) 8.63 (s, 4H), 8.38 (s, 4H), 8.21 (s, 4H), 8.11 (s, 4H), 7.78 (d, 4H), 7.53 (d, 4H). Molar ratio based on integration of the peaks: E_7: E_9 = 1: 1.

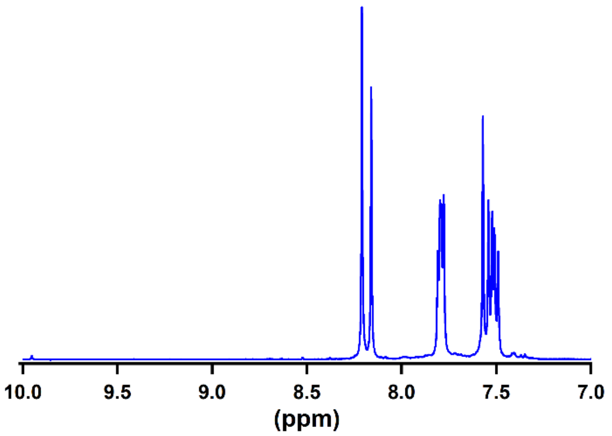

Supplementary Figure 95 | ^{1}H NMR spectrum of the digested MC-COF-NiPc-E_9E_{10} (400 MHz, d_6-DMSO). δ (ppm) 8.21-8.16 (d, 8H), 7.81-7.78 (m, 8H), 7.57-7.49 (m, 12H). Molar ratio based on integration of the peaks: E_9: E_{10} = 1: 1.

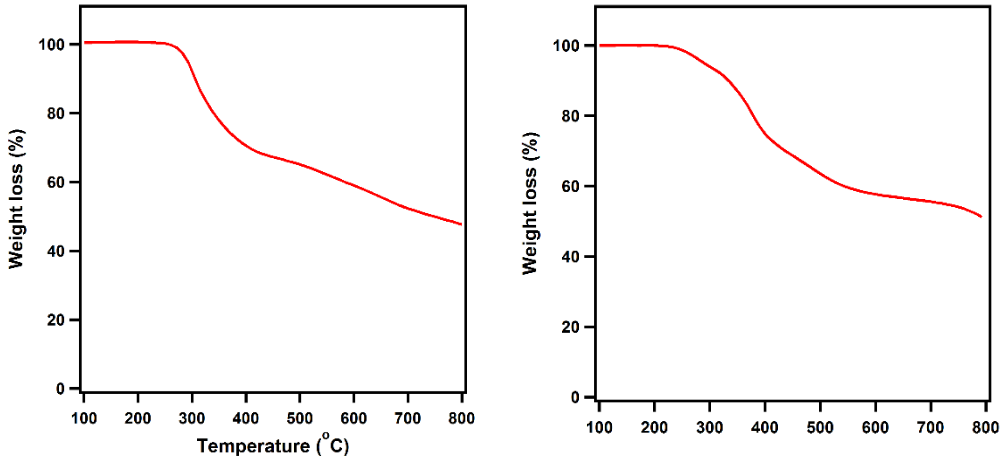

Supplementary Figure 96 | TGA curves of MC-COF-TP-$E_1^1E_2^2$ (left) and MC-COF-TP-$E_1^2E_2^1$ (right).

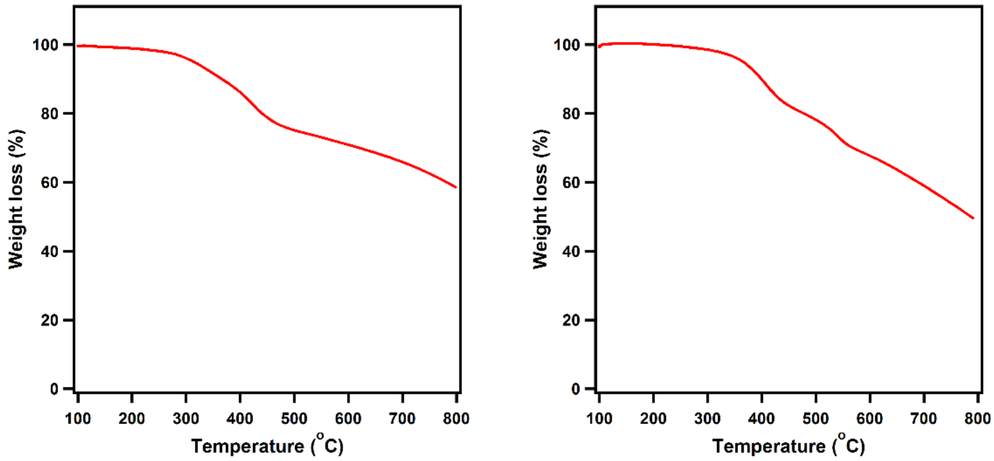

Supplementary Figure 97 | TGA curves of MC-COF-TP-$E_1^1E_3^2$ (left) and MC-COF-TP-$E_1^2E_3^1$ (right).

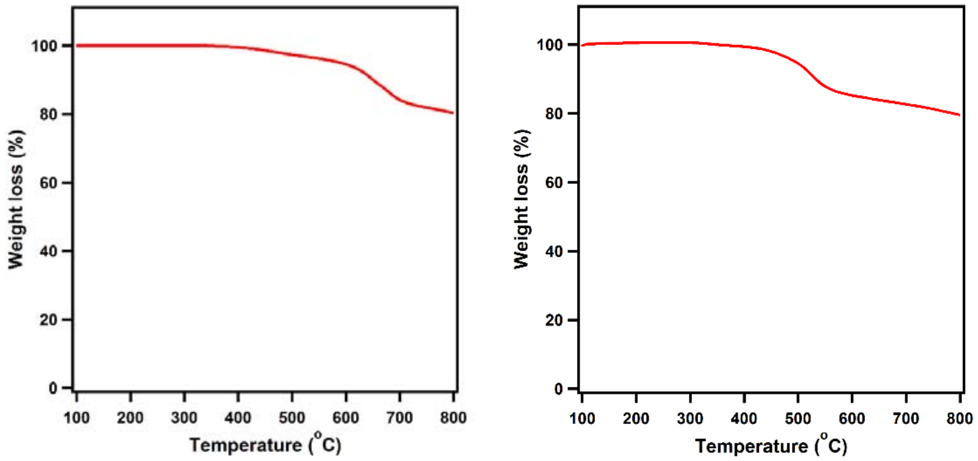

Supplementary Figure 98 | TGA curves of MC-COF-TP-$E_1^1E_4^2$ (left) and MC-COF-TP-$E_1^2E_4^1$ (right).

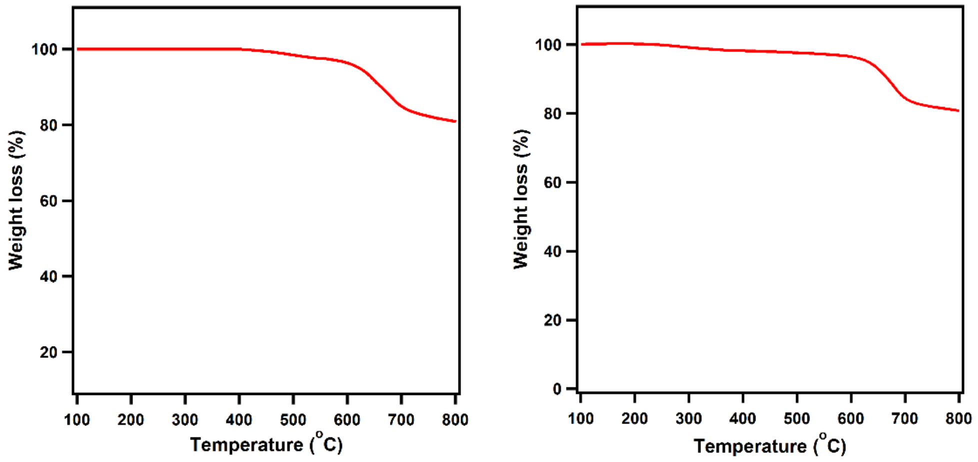

Supplementary Figure 99 | TGA curves of MC-COF-TP-$E_1^1E_6^2$ (left) and MC-COF-TP-$E_1^2E_6^1$ (right).

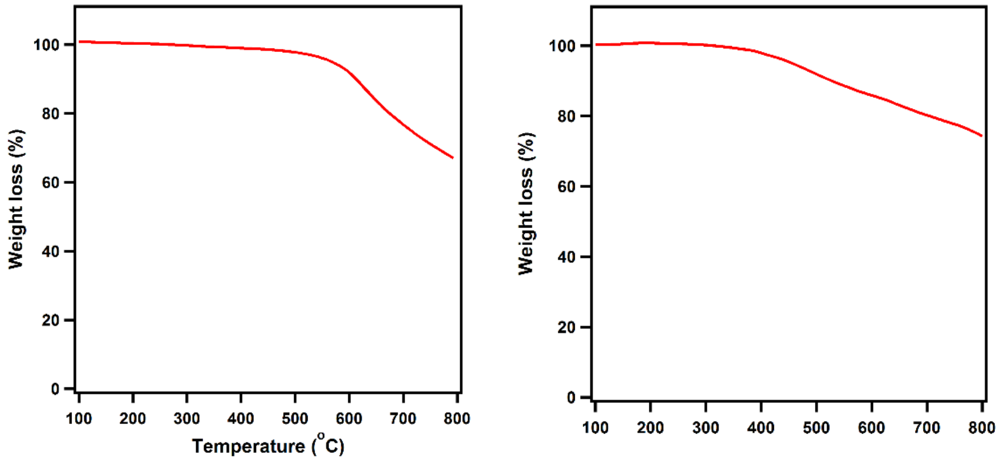

Supplementary Figure 100 | TGA curves of MC-COF-TP-$E_1^1E_7^2$ (left) and MC-COF-TP-$E_1^2E_7^1$ (right).

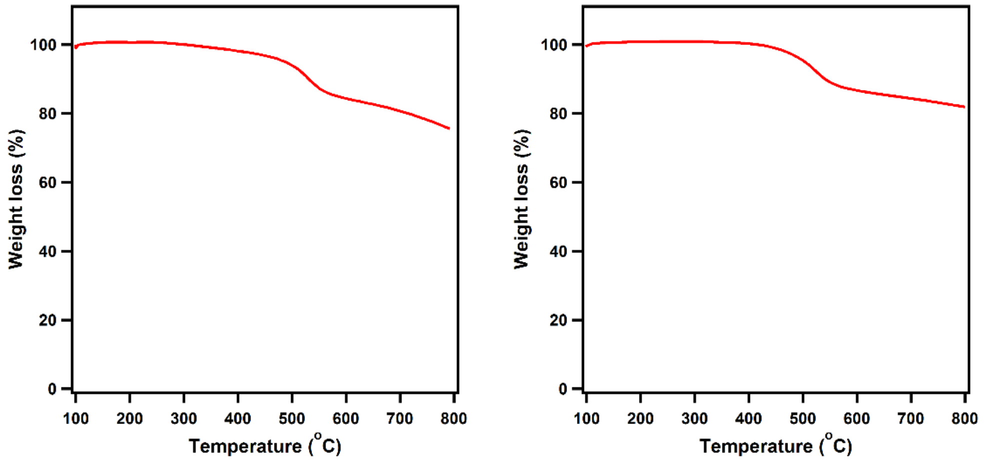

Supplementary Figure 101 | TGA curves of MC-COF-TP-$E_1^1E_8^2$ (left) and MC-COF-TP-$E_1^2E_8^1$ (right).

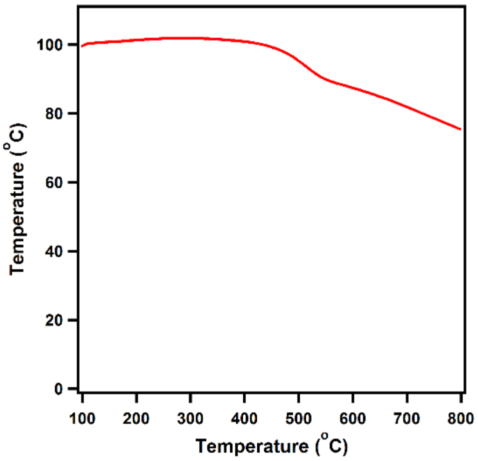

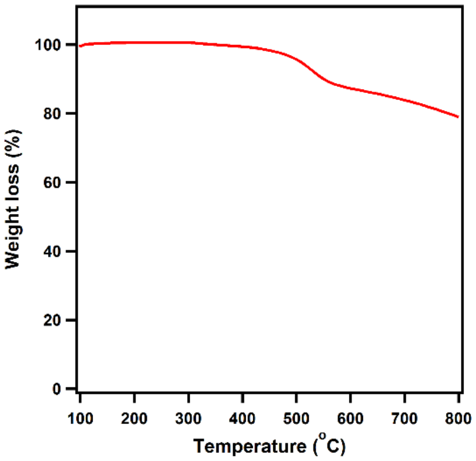

Supplementary Figure 102 | TGA curves of MC-COF-TP-$E_2^1E_3^2$ (left) and MC-COF-TP-$E_2^2E_3^1$ (right).

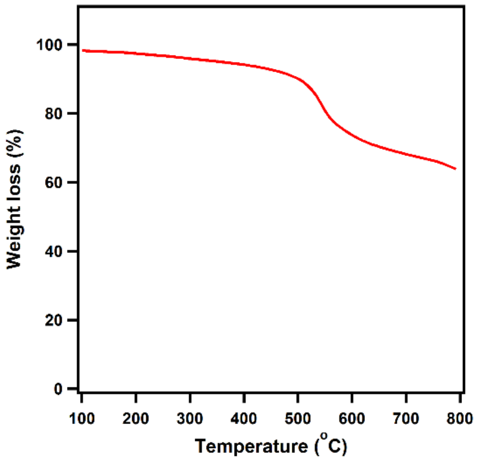

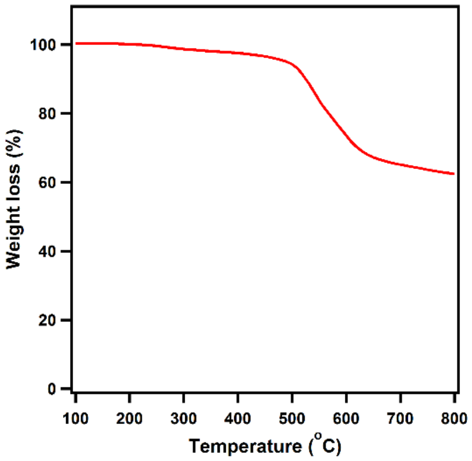

Supplementary Figure 103 | TGA curves of MC-COF-TP-$E_2^1E_5^2$ (left) and MC-COF-TP-$E_2^2E_5^1$ (right).

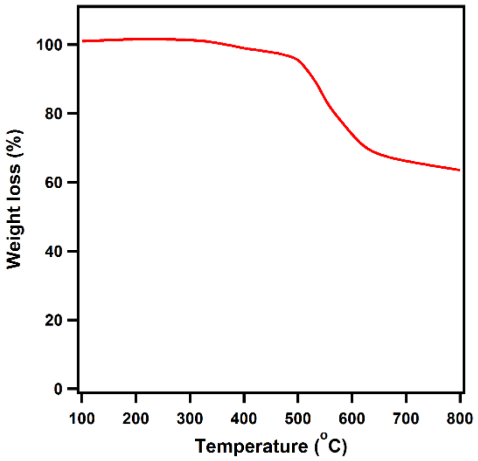

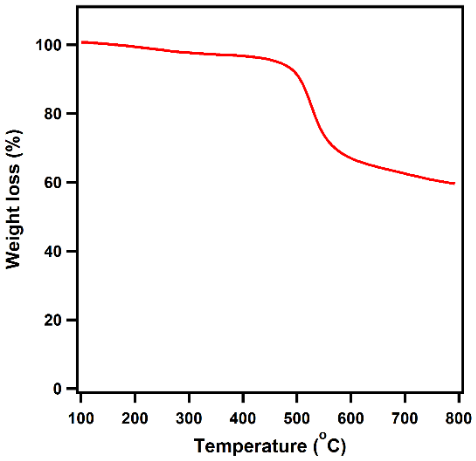

Supplementary Figure 104 | TGA curves of MC-COF-TP-$E_2^1E_6^2$ (left) and MC-COF-TP-$E_2^2E_6^1$ (right).

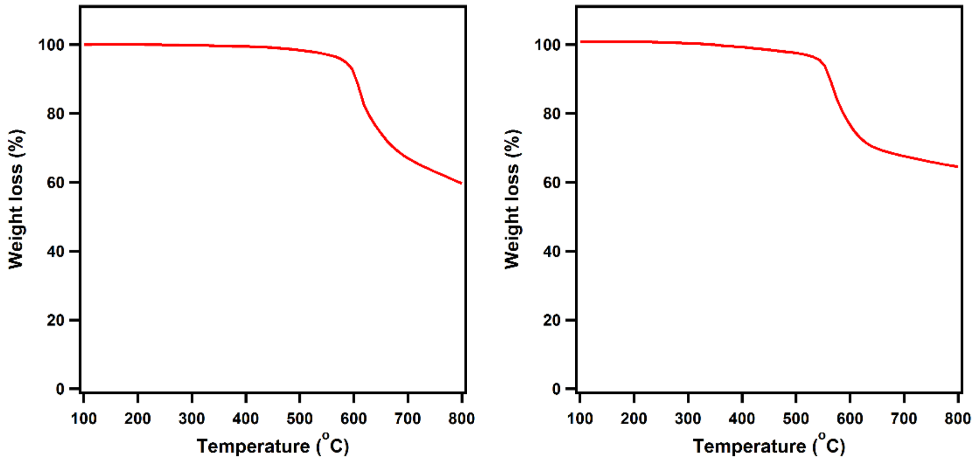

Supplementary Figure 105 | TGA curves of MC-COF-TP-$E_2^1E_7^2$ (left) and MC-COF-TP-$E_2^2E_7^1$ (right).

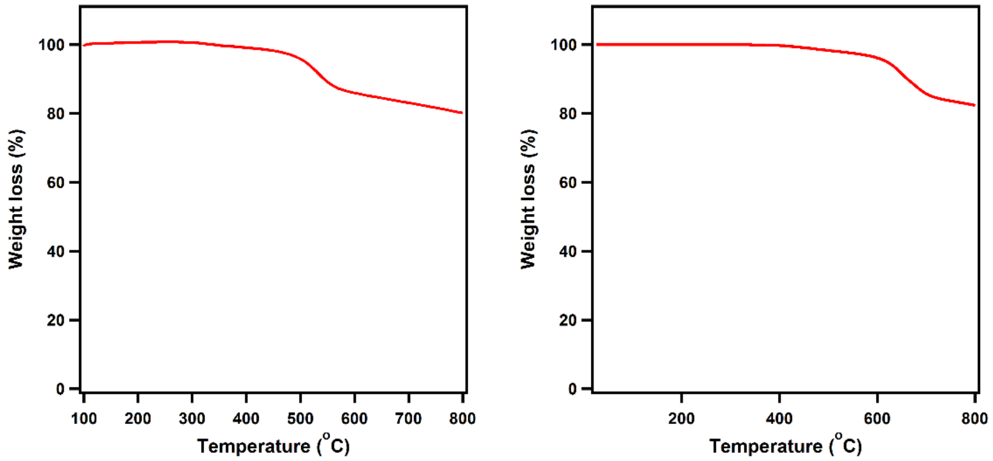

Supplementary Figure 106 | TGA curves of MC-COF-TP-$E_3^1E_4^2$ (left) and MC-COF-TP-$E_3^2E_4^1$ (right).

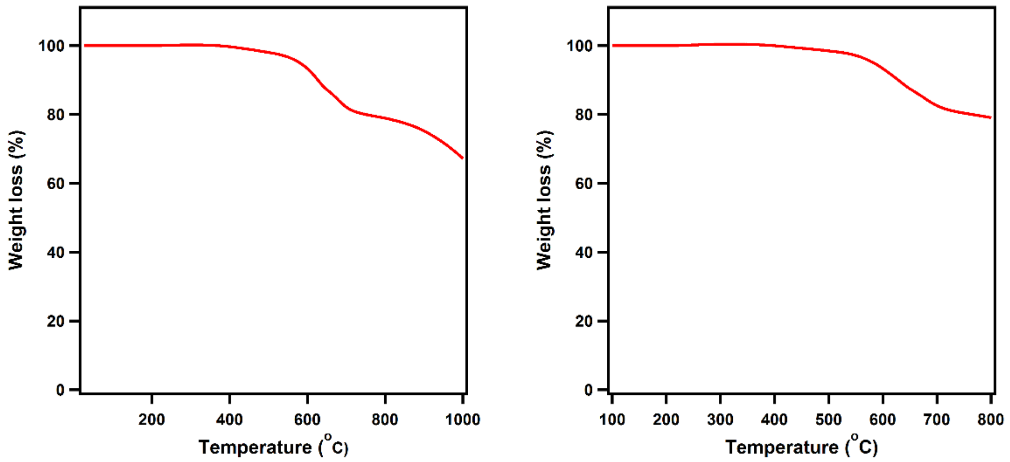

Supplementary Figure 107 | TGA curves of MC-COF-TP-$E_3^1E_6^2$ (left) and MC-COF-TP-$E_3^2E_6^1$ (right).

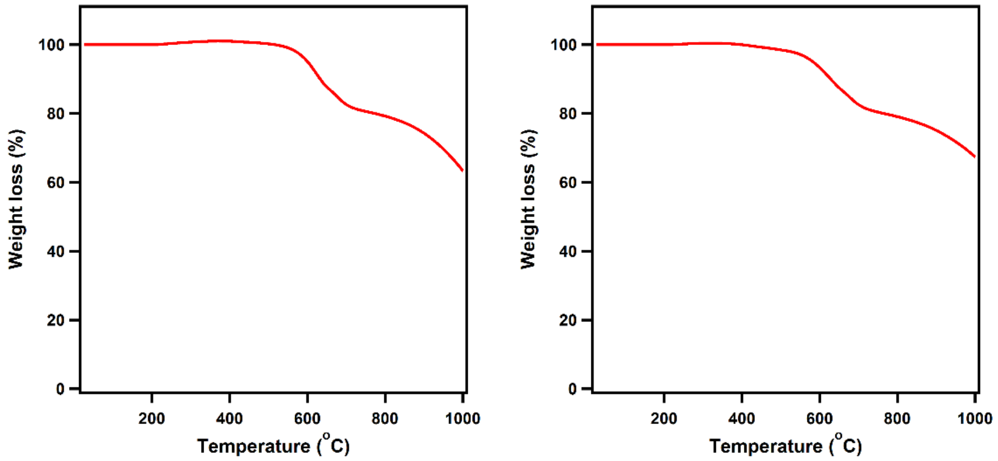

Supplementary Figure 108 | TGA curves of MC-COF-TP-$E_3{}^1E_7{}^2$ (left) and MC-COF-TP-$E_3{}^2E_7{}^1$ (right).

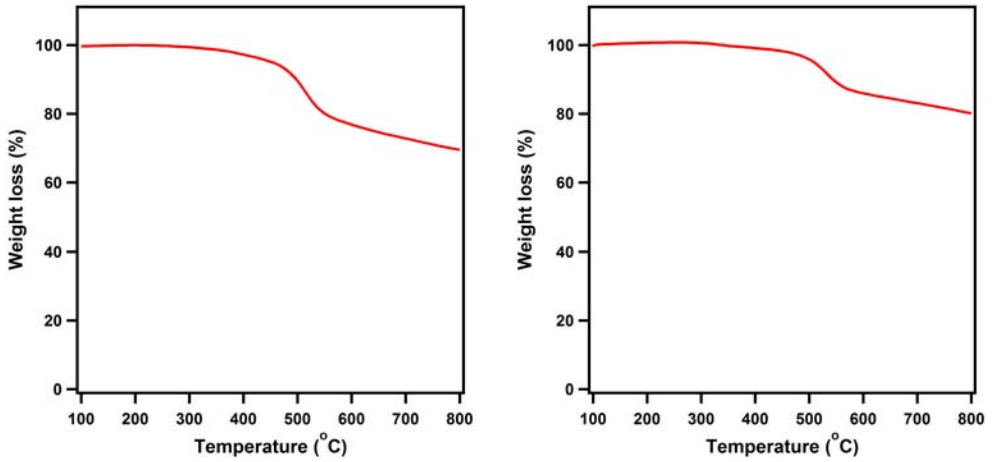

Supplementary Figure 109 | TGA curves of MC-COF-TP-$E_3{}^1E_8{}^2$ (left) and MC-COF-TP-$E_3{}^2E_8{}^1$ (right).

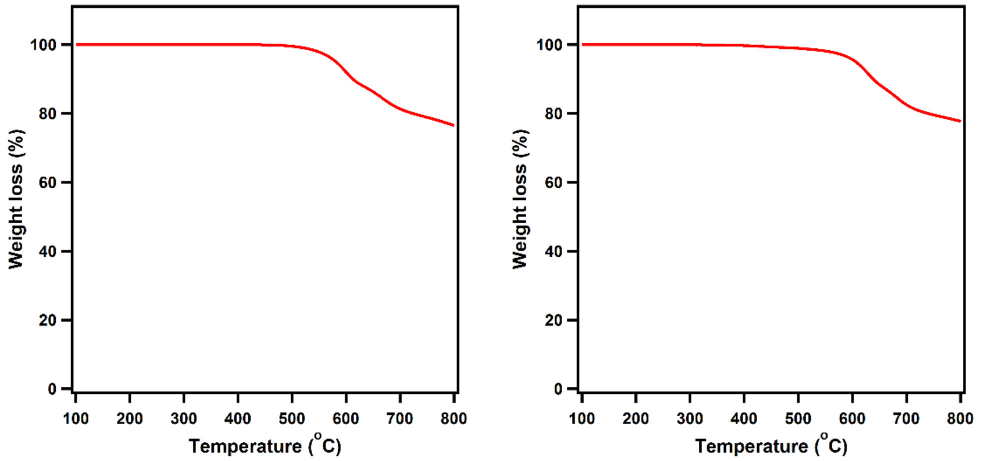

Supplementary Figure 110 | TGA curves of MC-COF-TP-$E_4{}^1E_5{}^2$ (left) and MC-COF-TP-$E_4{}^2E_5{}^1$ (right).

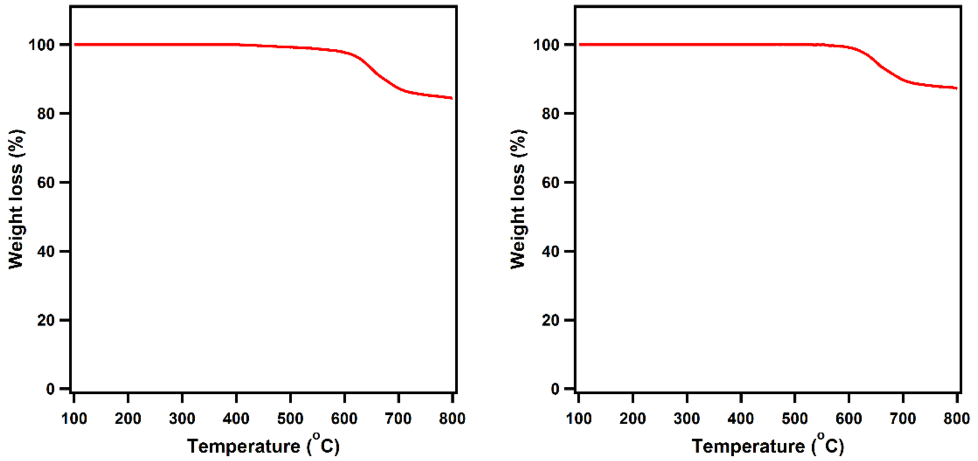

Supplementary Figure 111 | TGA curves of MC-COF-TP-$E_4^1E_7^2$ (left) and MC-COF-TP-$E_4^2E_7^1$ (right).

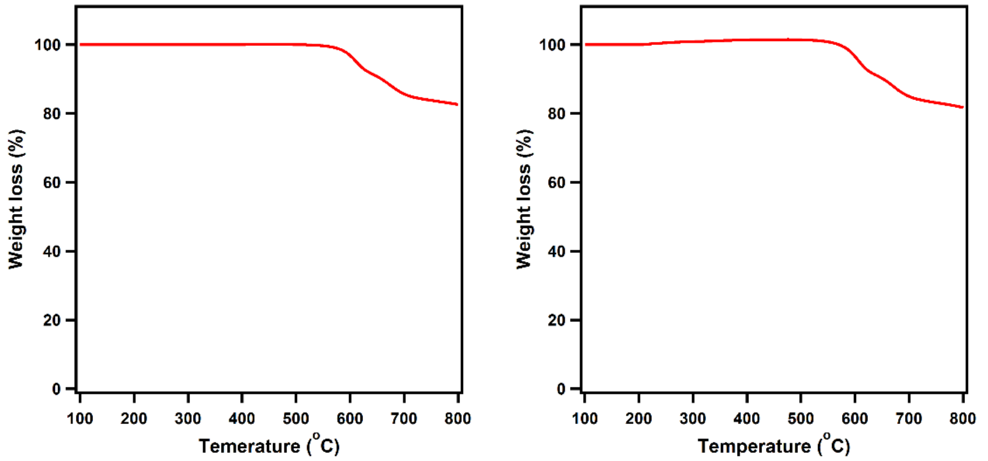

Supplementary Figure 112 | TGA curves of MC-COF-TP-$E_4^1E_8^2$ (left) and MC-COF-TP-$E_4^2E_8^1$ (right).

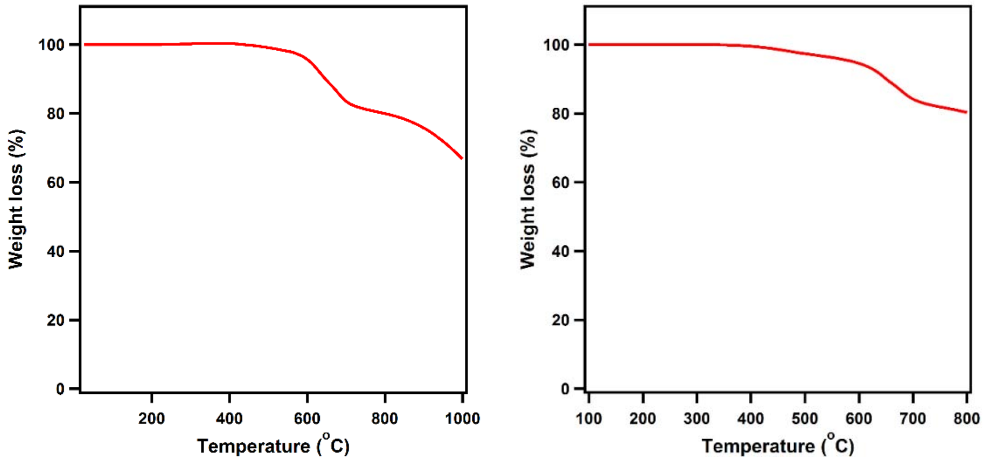

Supplementary Figure 113 | TGA curves of MC-COF-TP-$E_5^1E_6^2$ (left) and MC-COF-TP-$E_5^2E_6^1$ (right).

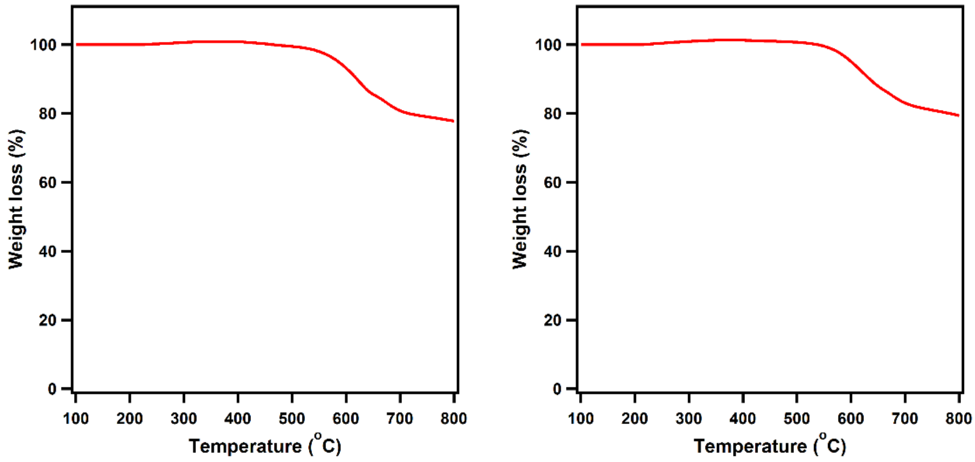

Supplementary Figure 114 | TGA curves of MC-COF-TP-$E_5{}^1E_7{}^2$ (left) and MC-COF-TP-$E_5{}^2E_7{}^1$ (right).

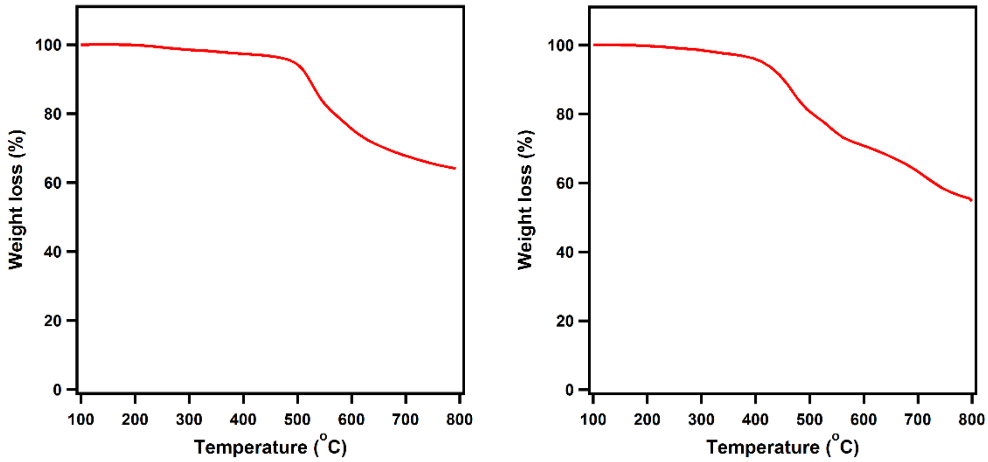

Supplementary Figure 115 | TGA curves of MC-COF-TP-$E_5{}^1E_8{}^2$ (left) and MC-COF- $E_5{}^2E_8{}^1$ (right).

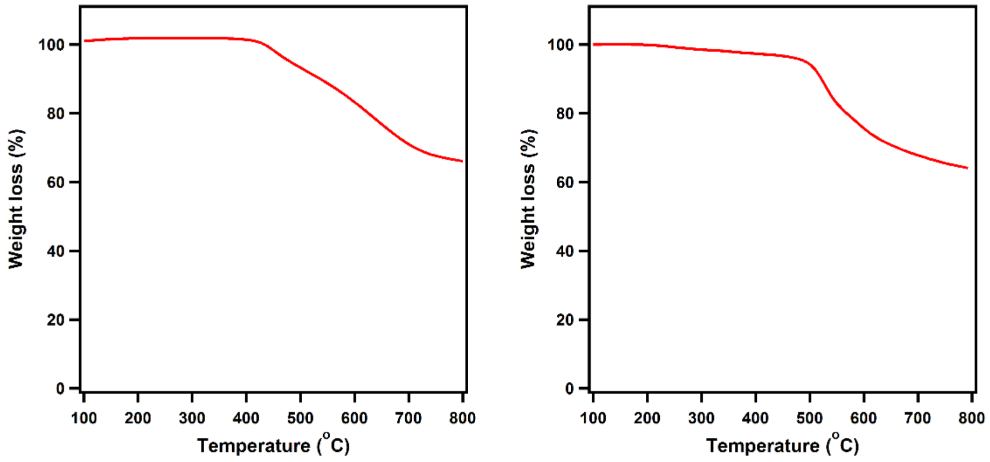

Supplementary Figure 116 | TGA curves of MC-COF-TP-$E_6{}^1E_7{}^2$ (left) and MC-COF- $E_6{}^2E_7{}^1$ (right).

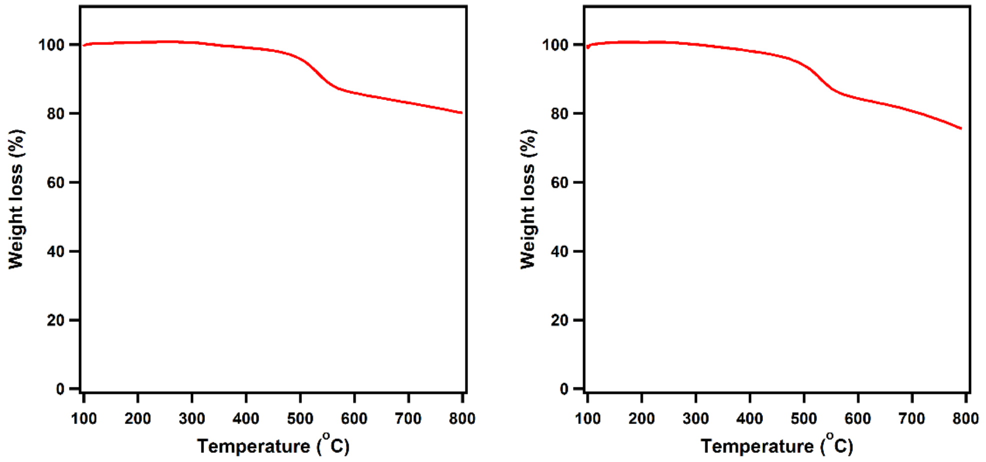

Supplementary Figure 117 | TGA curves of MC-COF-TP-$E_7^1E_8^2$ (left) and MC-COF- $E_7^2E_8^1$ (right).

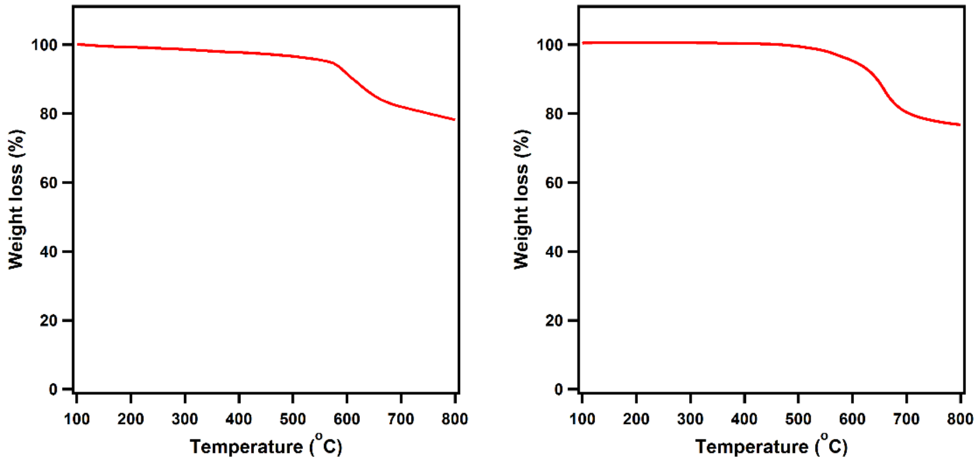

Supplementary Figure 118 | TGA curves of MC-COF-TP-$E_1E_3E_7$ (left) and MC-COF-TP-$E_1E_4E_7$ (right).

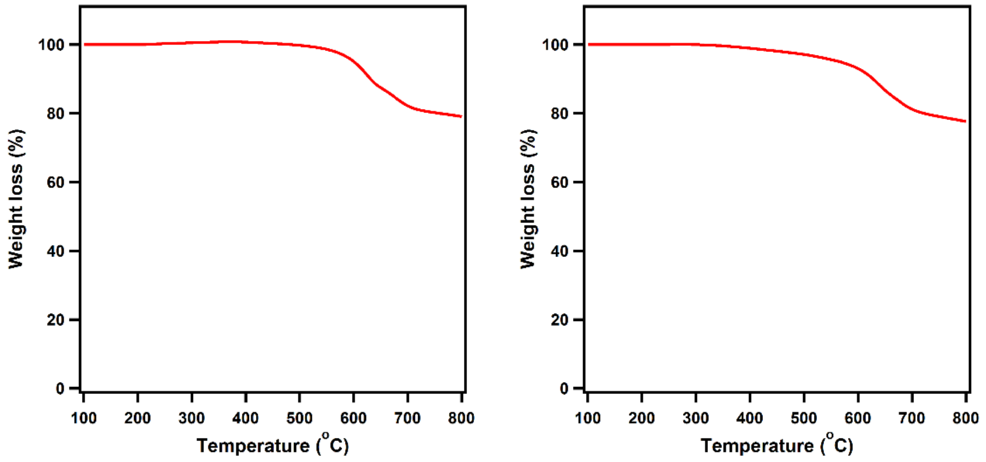

Supplementary Figure 119 | TGA curves of MC-COF-TP-$E_4E_5E_7$ (left) and MC-COF-TP-$E_4E_6E_7$ (right).

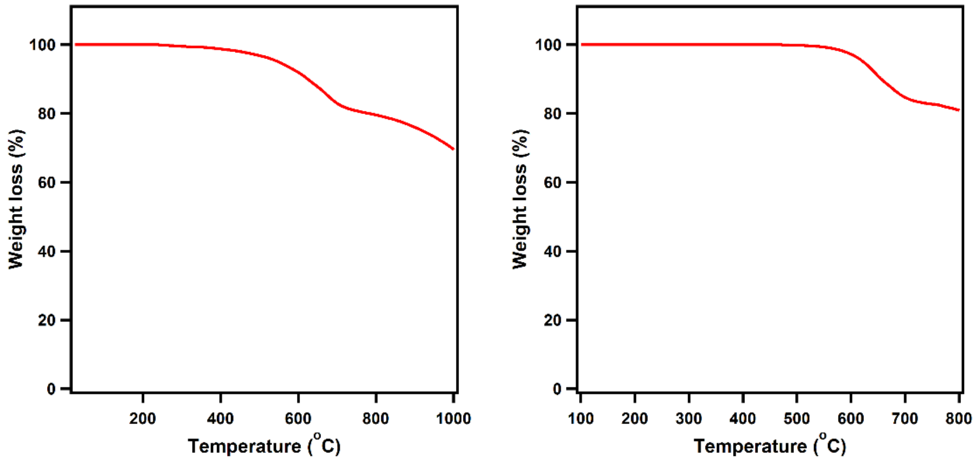

Supplementary Figure 120 | TGA curves of MC-COF-TP-$E_5E_7E_8$ (left) and MC-COF-TP-$E_3E_4E_8$ (right).

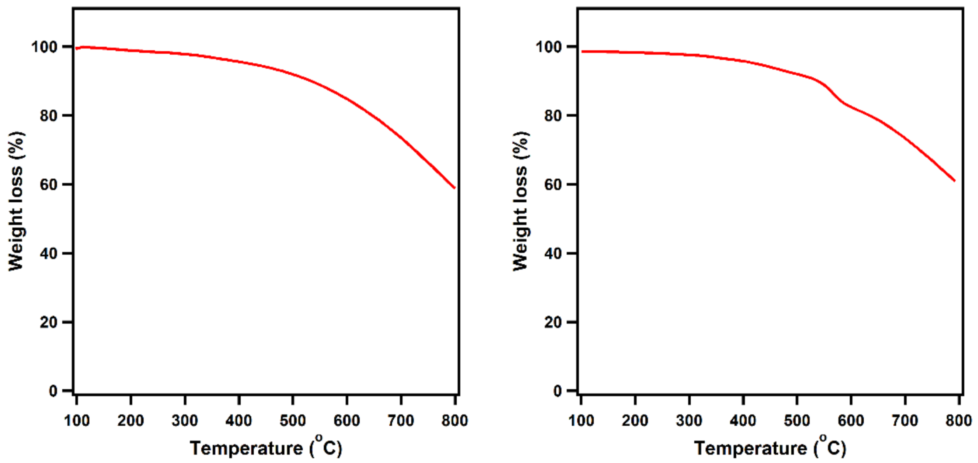

Supplementary Figure 121 | TGA curves of MC-COF-NiPc-E_1E_7 (left) and MC-COF-NiPc-E_7E_9 (right).

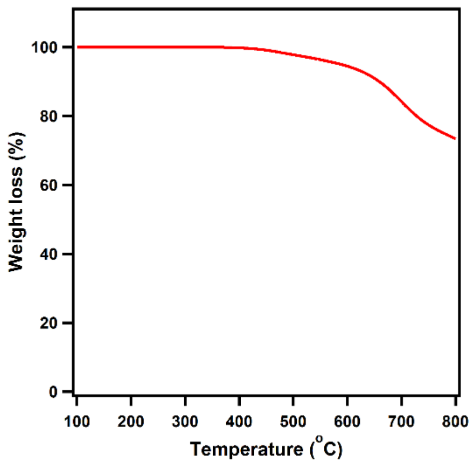

Supplementary Figure 122 | TGA curves of MC-COF-NiPc-E_9E_{10}.

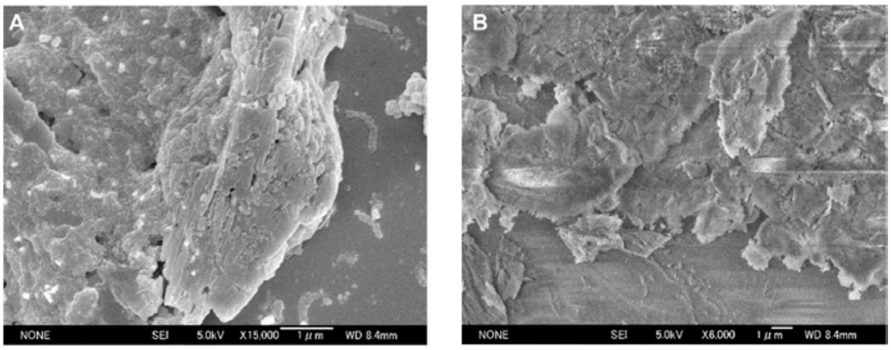

Supplementary Figure 123 | FE-SEM images of MC-COF-TP-$E_1{}^1E_2{}^2$ (A) and MC-COF-TP-$E_1{}^2E_2{}^1$ (B).

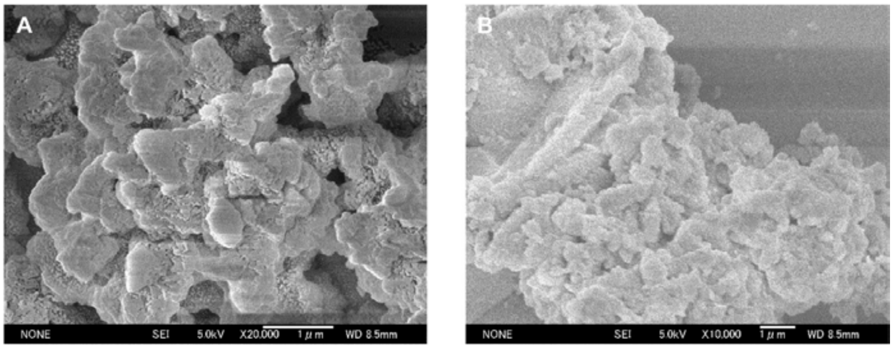

Supplementary Figure 124 | FE-SEM images of MC-COF-TP-$E_1{}^1E_3{}^2$ (A) and MC-COF-TP-$E_1{}^2E_3{}^1$ (B).

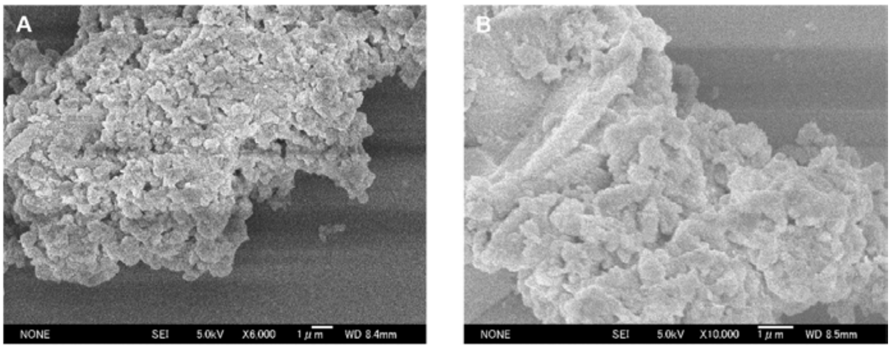

Supplementary Figure 125 | FE-SEM images of MC-COF-TP-$E_1{}^1E_4{}^2$ (A) and MC-COF-TP-$E_1{}^2E_4{}^1$ (B).

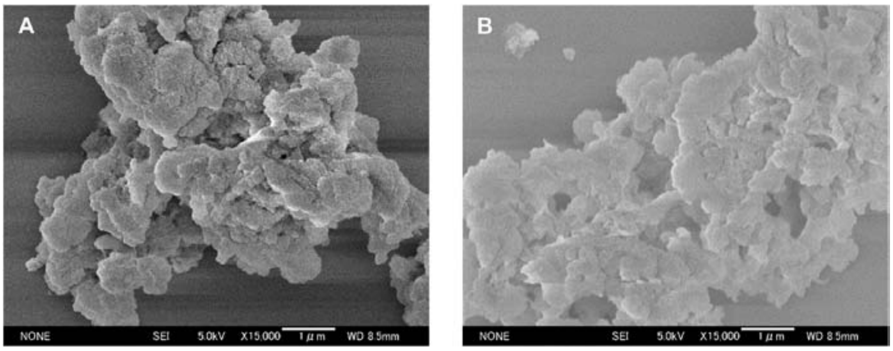

Supplementary Figure 126 | FE-SEM images of MC-COF-TP-$E_1^1E_6^2$ (A) and MC-COF-TP-$E_1^2E_6^1$ (B).

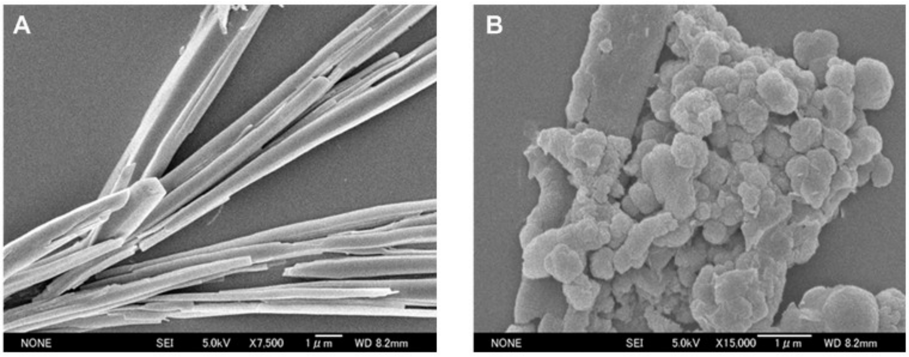

Supplementary Figure 127 | FE-SEM images of MC-COF-TP-$E_1^1E_7^2$ (A) and MC-COF-TP-$E_1^2E_7^1$ (B).

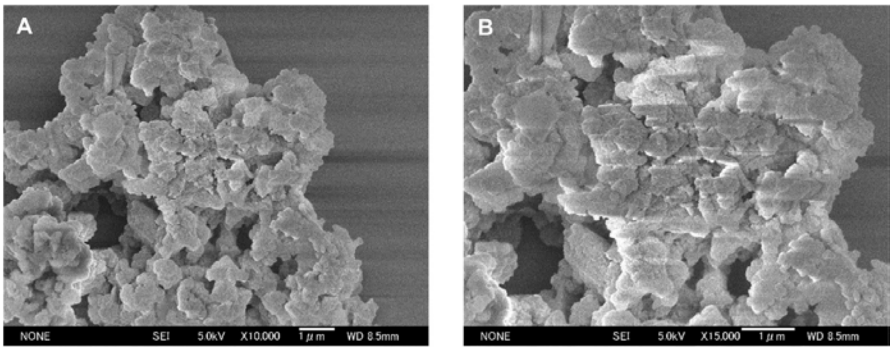

Supplementary Figure 128 | FE-SEM images of MC-COF-TP-$E_1^1E_8^2$ (A) and MC-COF-TP-$E_1^2E_8^1$ (B).

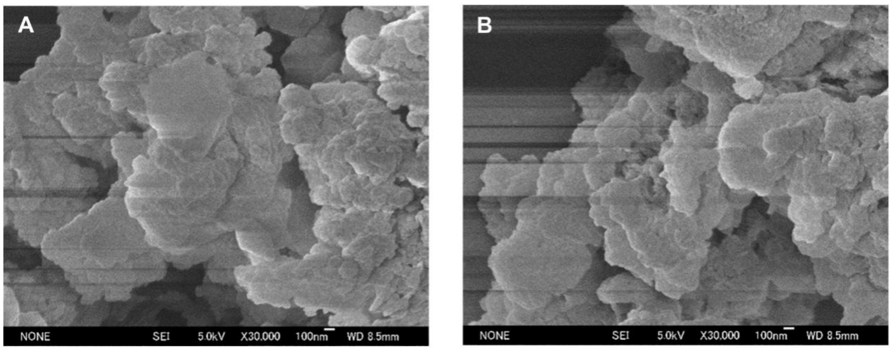

Supplementary Figure 129 | FE-SEM images of MC-COF-TP-$E_2^1E_3^2$ (A) and MC-COF-TP-$E_2^2E_3^1$ (B).

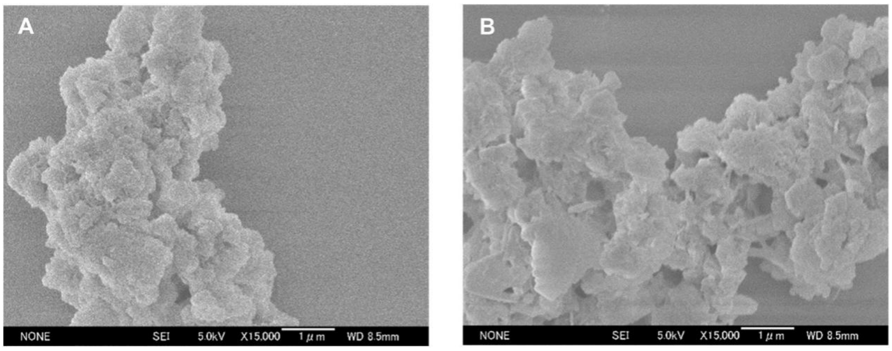

Supplementary Figure 130 | FE-SEM images of MC-COF-TP-$E_2^1E_4^2$ (A) and MC-COF-TP-$E_2^2E_4^1$ (B).

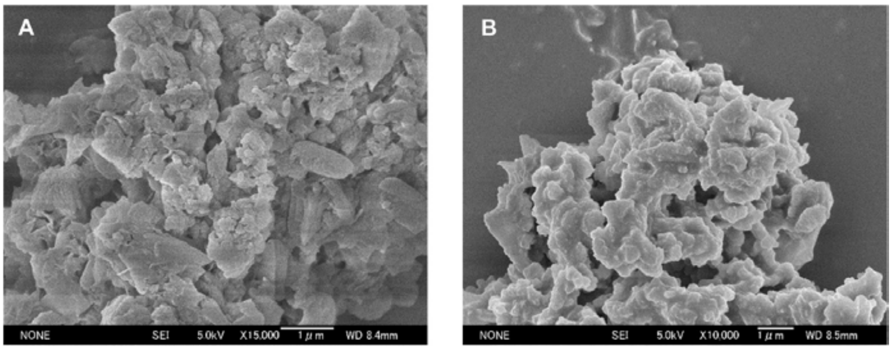

Supplementary Figure 131 | FE-SEM images of MC-COF-TP-$E_2^1E_6^2$ (A) and MC-COF-TP-$E_2^2E_6^1$ (B).

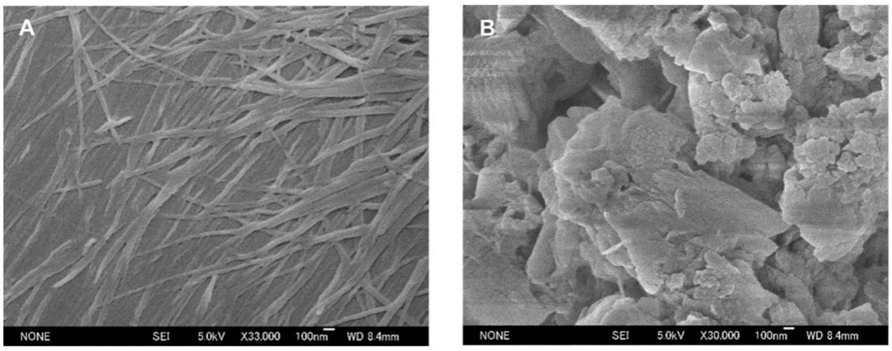

Supplementary Figure 132 | FE-SEM images of MC-COF-TP-$E_2^1E_7^2$ (A) and MC-COF-TP-$E_2^2E_7^1$ (B).

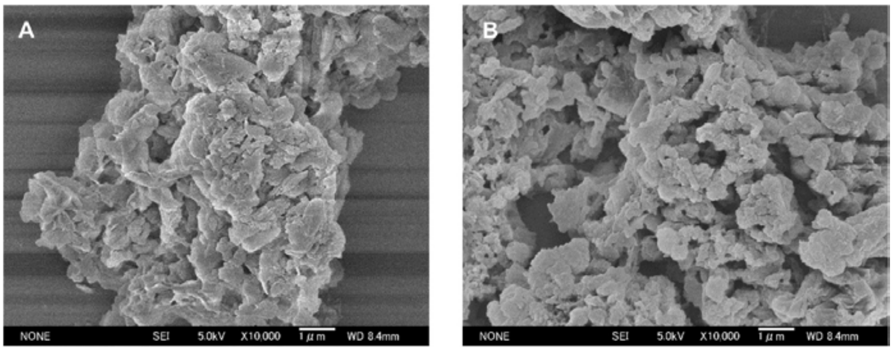

Supplementary Figure 133 | FE-SEM images of MC-COF-TP-$E_3^1E_4^2$ (A) and MC-COF-TP-$E_3^2E_4^1$ (B).

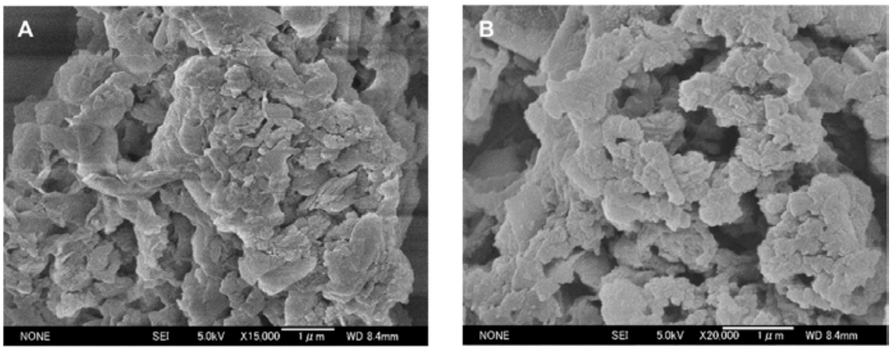

Supplementary Figure 134 | FE-SEM images of MC-COF-TP-$E_3^1E_6^2$ (A) and MC-COF-TP-$E_3^2E_6^1$ (B).

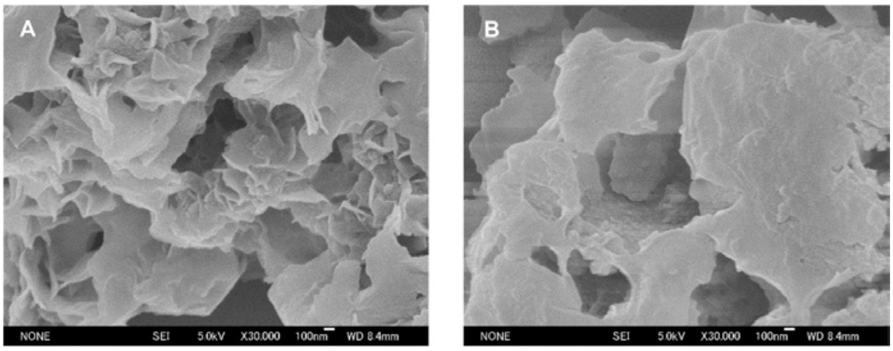

Supplementary Figure 135 | FE-SEM images of MC-COF-TP-$E_3^1E_8^2$ (A) and MC-COF-TP-$E_3^2E_8^1$ (B).

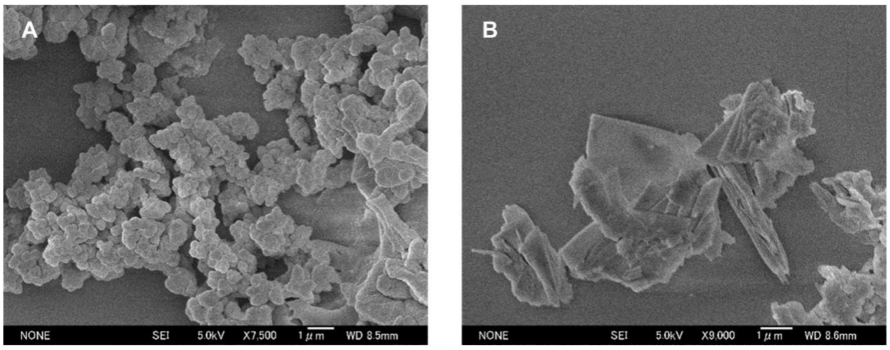

Supplementary Figure 136 | FE-SEM images of MC-COF-TP-$E_4^1E_5^2$ (A) and MC-COF-TP-$E_4^2E_5^1$ (B).

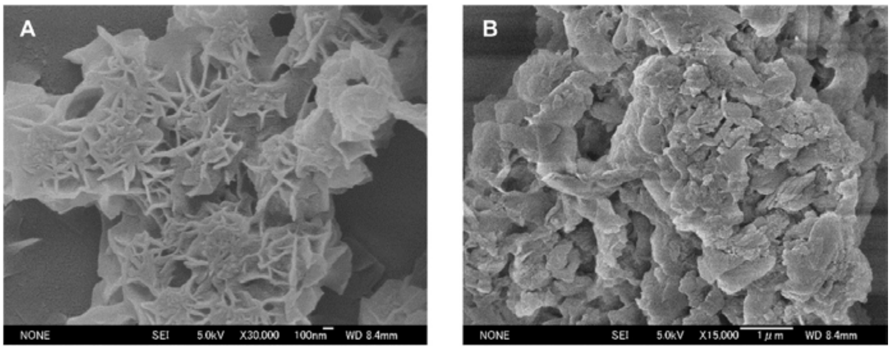

Supplementary Figure 137 | FE-SEM images of MC-COF-TP-$E_4^1E_7^2$ (A) and MC-COF-TP-$E_4^2E_7^1$ (B).

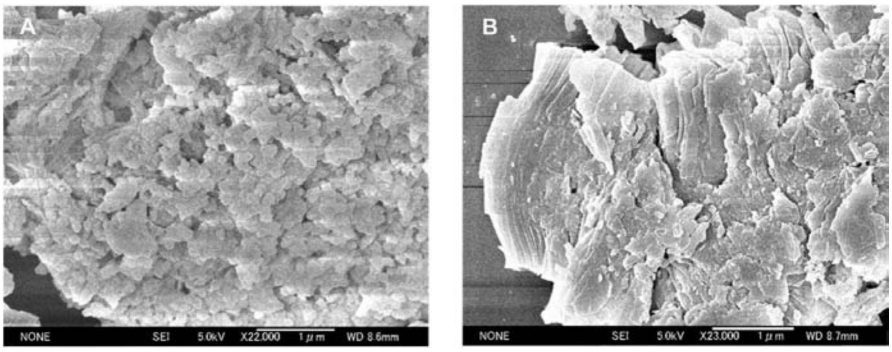

Supplementary Figure 138 | FE-SEM images of MC-COF-TP-$E_4^1E_8^2$ (A) and MC-COF-TP-$E_4^2E_8^1$ (B).

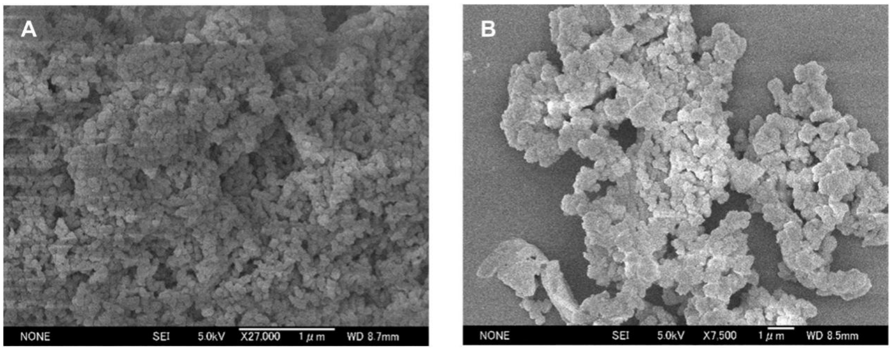

Supplementary Figure 139 | FE-SEM images of MC-COF-TP-$E_5^1E_6^2$ (A) and MC-COF-TP-$E_5^2E_6^1$ (B).

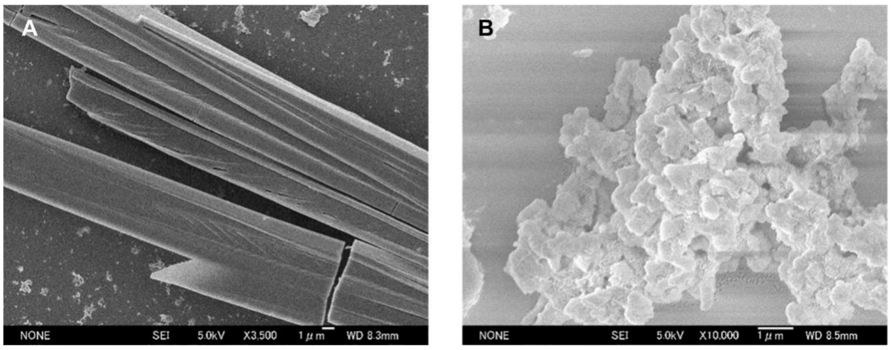

Supplementary Figure 140 | FE-SEM images of MC-COF-TP-$E_5^1E_7^2$ (A) and MC-COF- $E_5^2E_7^1$ (B).

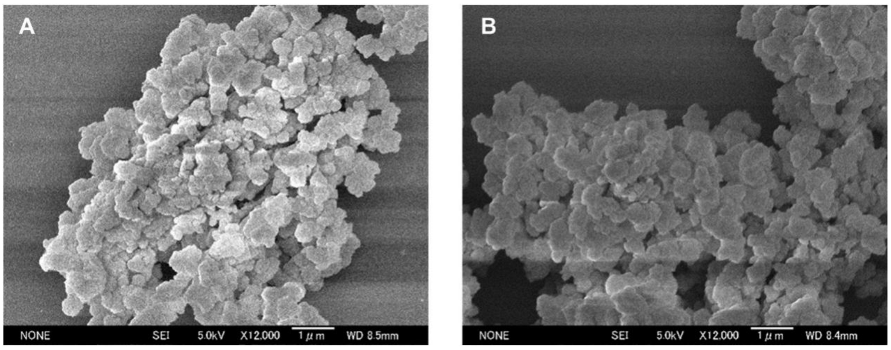

Supplementary Figure 141 | FE-SEM images of MC-COF-TP-$E_5{}^1E_8{}^2$ (A) and MC-COF- $E_5{}^2E_8{}^1$ (B).

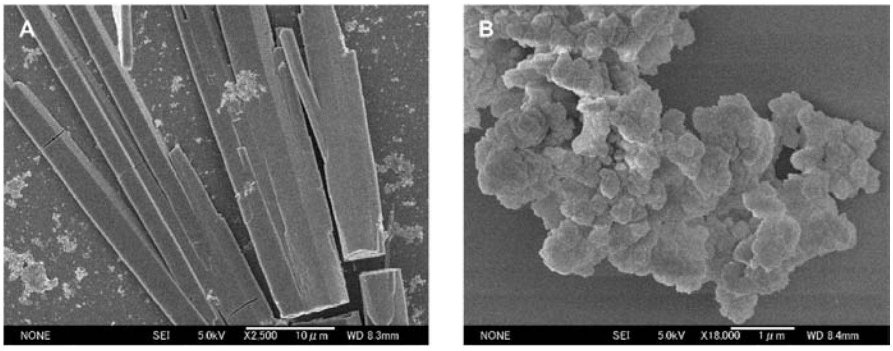

Supplementary Figure 142 | FE-SEM images of MC-COF-TP-$E_6{}^1E_7{}^2$ (A) and MC-COF- $E_6{}^2E_7{}^1$ (B).

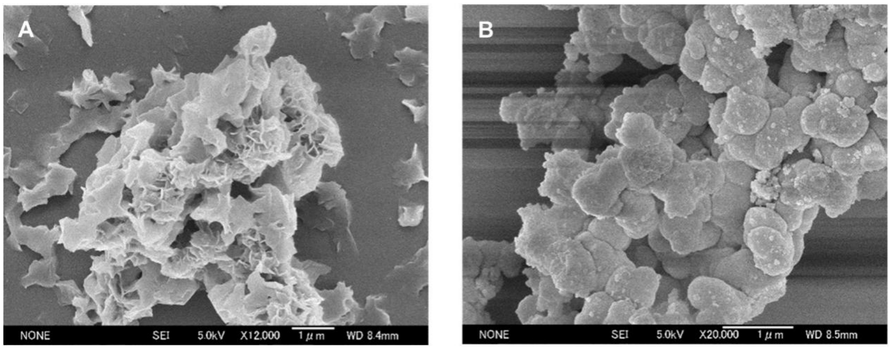

Supplementary Figure 143 | FE-SEM images of MC-COF-TP-$E_7{}^1E_8{}^2$ (A) and MC-COF- $E_7{}^2E_8{}^1$ (B).

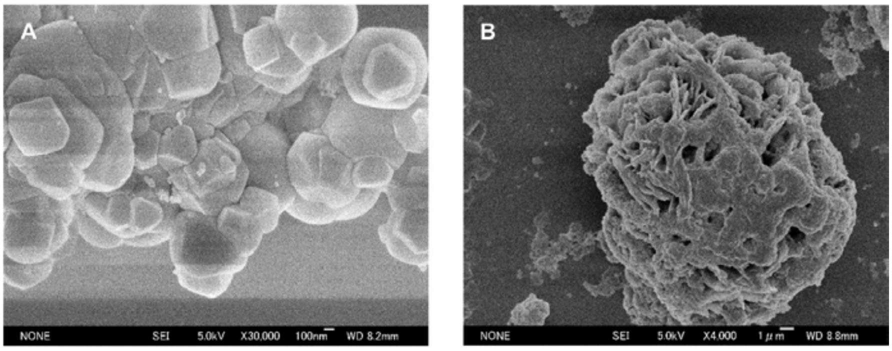

Supplementary Figure 144 | FE-SEM image of MC-COF-TP- $E_1E_3E_7$ (A) and MC-COF-TP-$E_1E_4E_7$ (B)

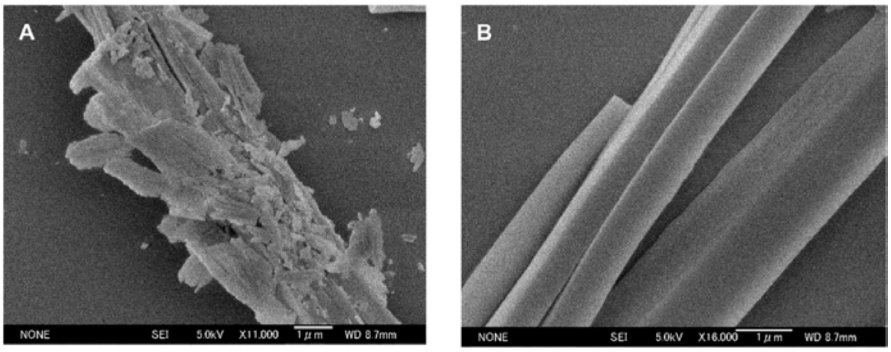

Supplementary Figure 145 | FE-SEM image of MC-COF-TP-$E_4E_5E_7$ (A) and MC-COF-TP-$E_4E_6E_7$ (B).

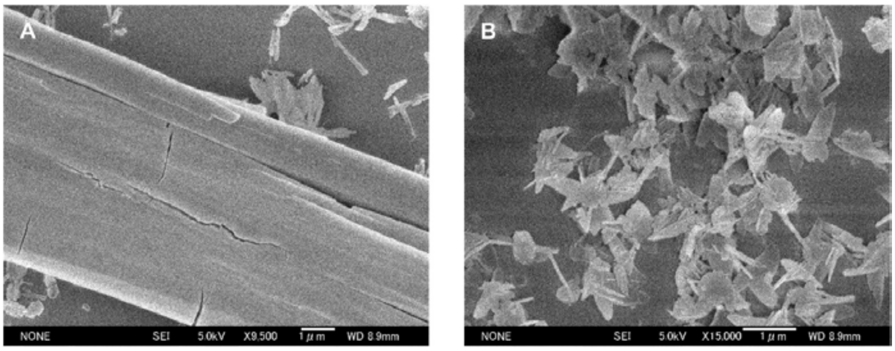

Supplementary Figure 146 | FE-SEM image of MC-COF-TP-$E_5E_7E_8$ (A) and MC-COF-TP-$E_3E_4E_8$ (B).

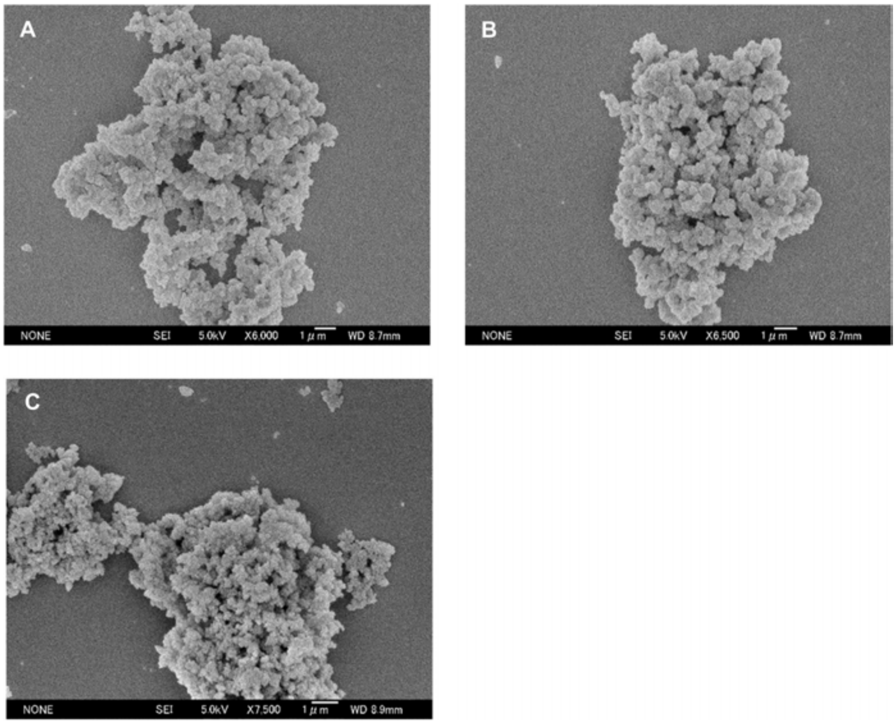

Supplementary Figure 147 | FE-SEM image of MC-COF-NiPc-E_1E_7 (A), MC-COF-NiPc-E_7E_9 (B), and MC-COF-NiPc-E_9E_{10} (C).

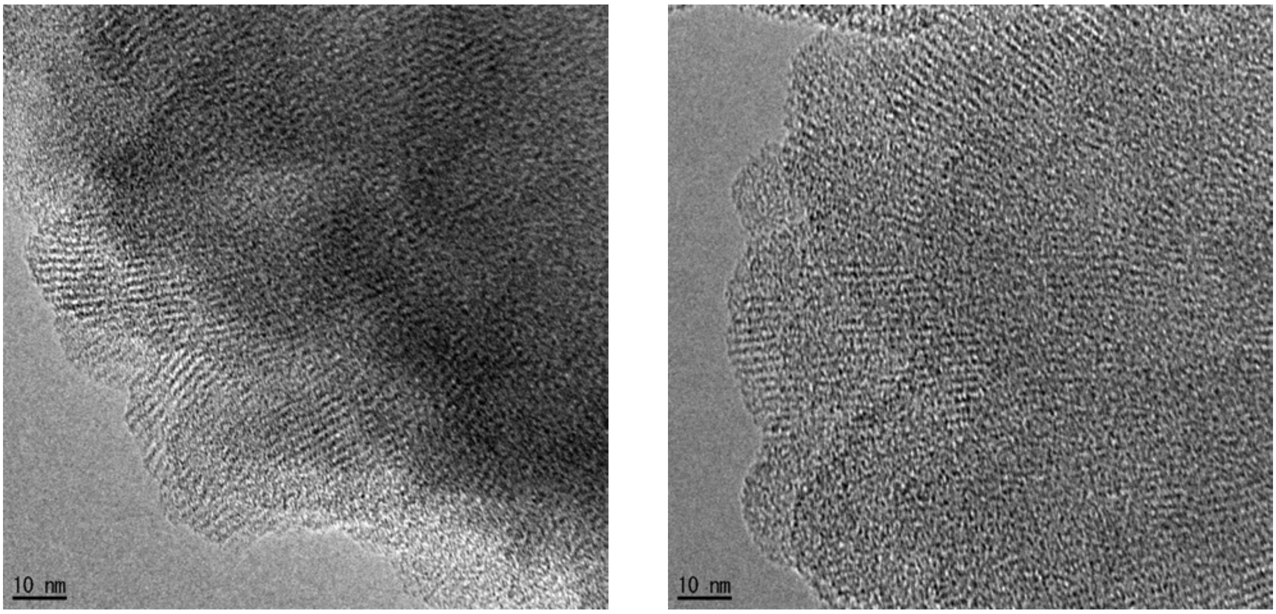

Supplementary Figure 148 | HR TEM images of MC-COF-TP-$E_1^{1}E_7^{2}$.

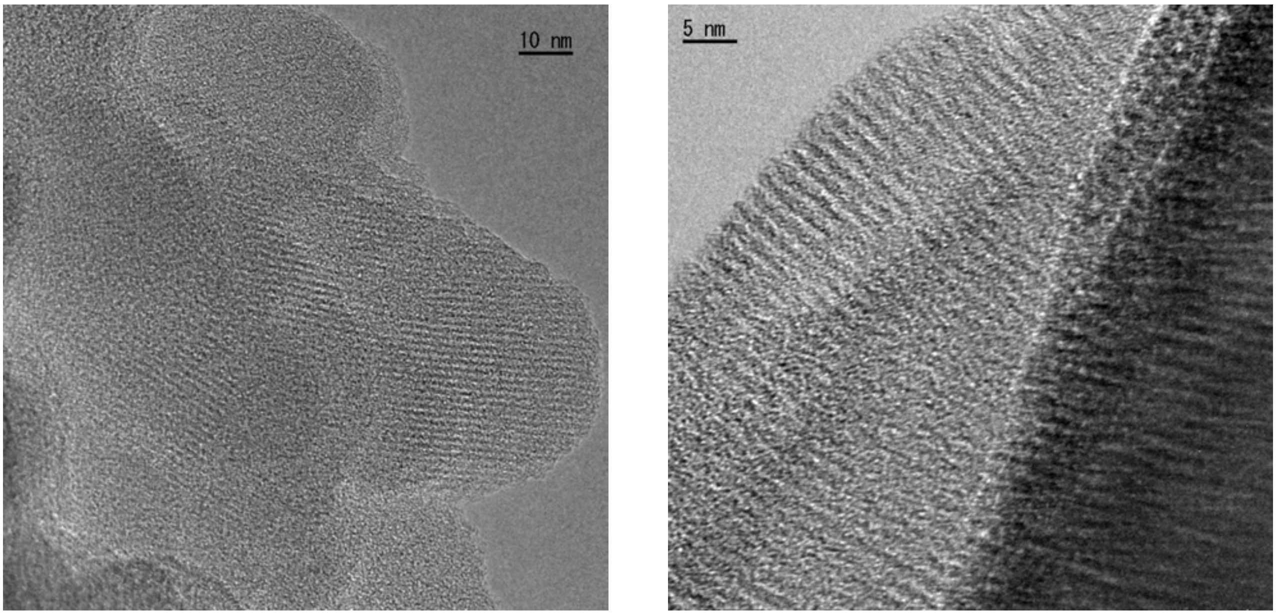

Supplementary Figure 149 | HR TEM images of MC-COF-TP-$E_1^{2}E_7^{1}$.

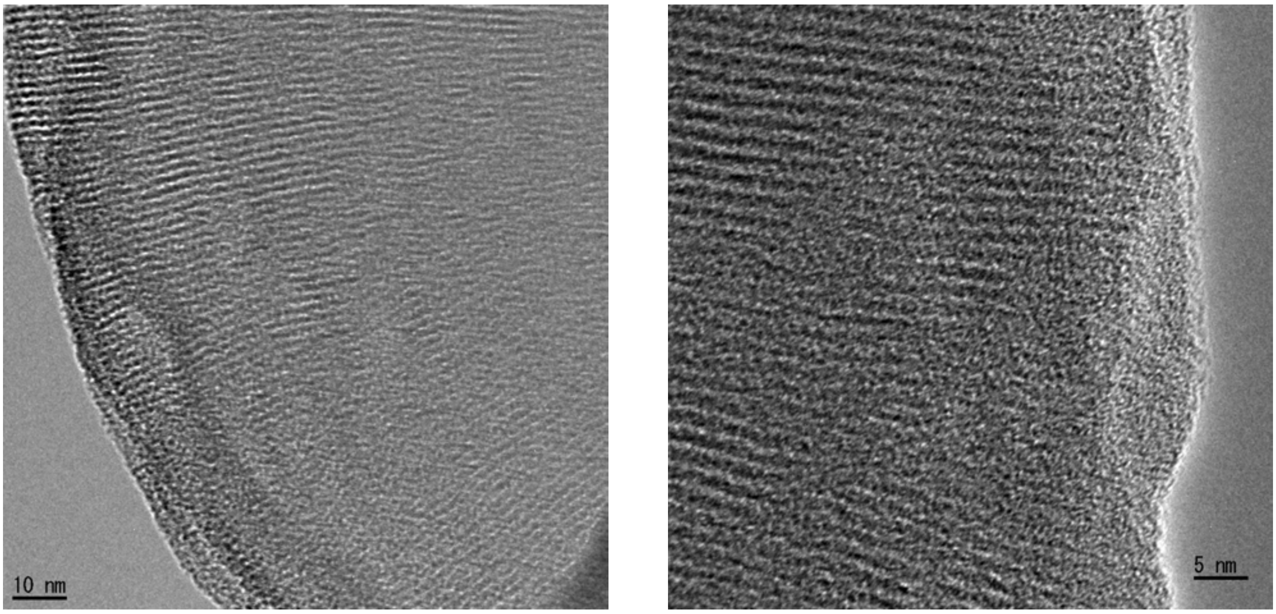

Supplementary Figure 150 | HR TEM images of MC-COF-TP-$E_1E_3E_7$.

Section H Nitrogen sorption curves and pore size distribution profiles

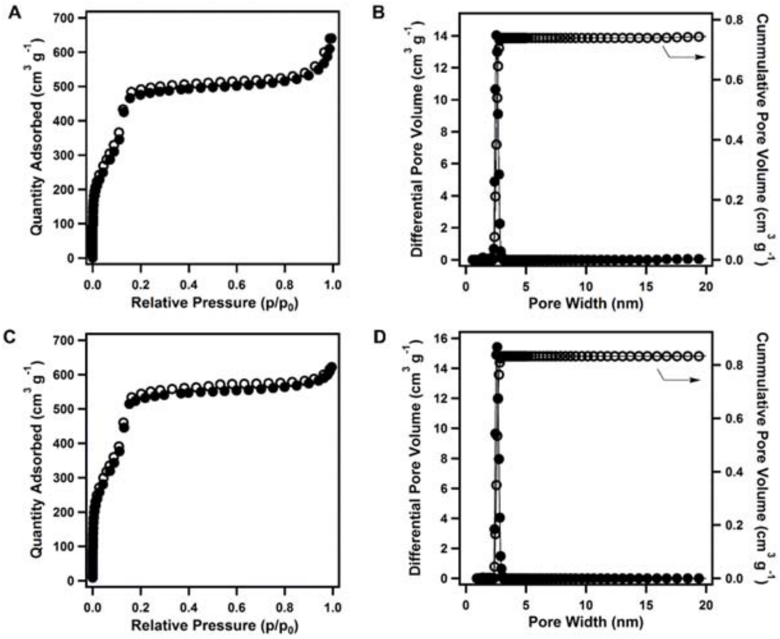

Supplementary Figure 151 | A) Nitrogen-sorption isotherm curves of MC-COF-TP-$E_1{}^1E_2{}^2$ measured at 77 K. B) Profiles of the pore size and pore-size distribution of MC-COF-TP-$E_1{}^1E_2{}^2$. C) Nitrogen-sorption isotherm curves of MC-COF-TP-$E_1{}^2E_2{}^1$ measured at 77 K. D) Profiles of the pore size and pore-size distribution of MC-COF-TP-$E_1{}^2E_2{}^1$.

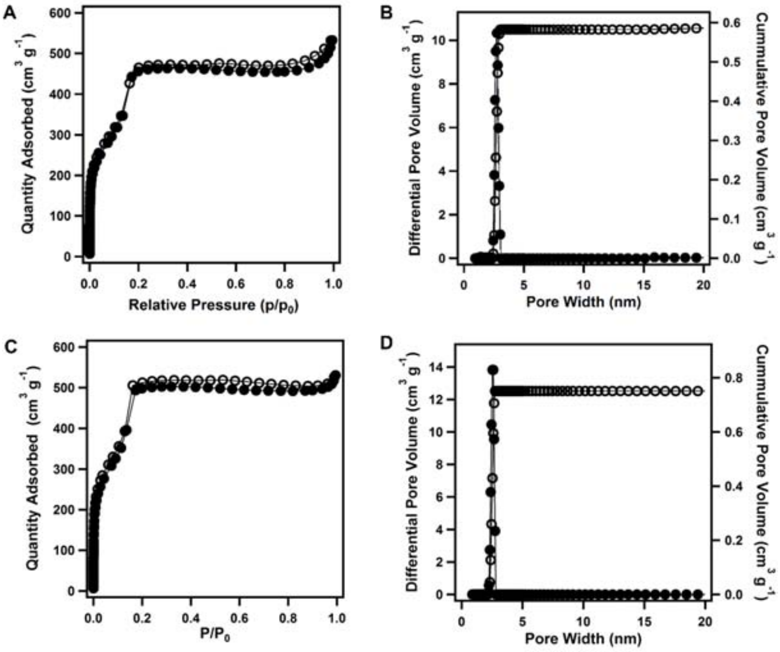

Supplementary Figure 152 | A) Nitrogen-sorption isotherm curves of MC-COF-TP-$E_1{}^1E_3{}^2$ measured at 77 K. B) Profiles of the pore size and pore-size distribution of MC-COF-TP-$E_1{}^1E_3{}^2$. C) Nitrogen-sorption isotherm curves of MC-COF-TP-$E_1{}^2E_3{}^1$ measured at 77 K. D) Profiles of the pore size and pore-size distribution of MC-COF-TP-$E_1{}^2E_3{}^1$.

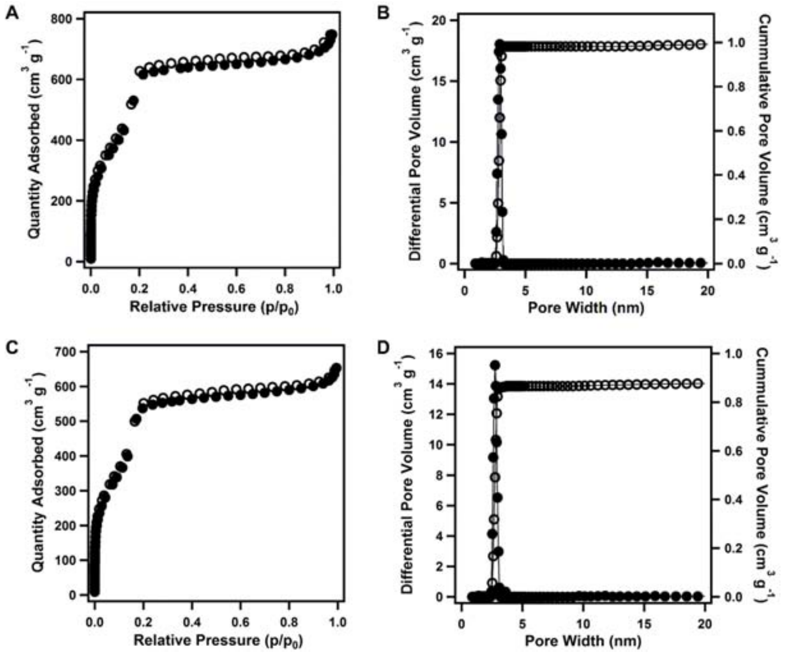

Supplementary Figure 153 | A) Nitrogen-sorption isotherm curves of MC-COF-TP-$E_1^{1}E_4^{2}$ measured at 77 K. B) Profiles of the pore size and pore-size distribution of MC-COF-TP-$E_1^{1}E_4^{2}$. C) Nitrogen-sorption isotherm curves of MC-COF-TP-$E_1^{2}E_4^{1}$ measured at 77 K. D) Profiles of the pore size and pore-size distribution of MC-COF-TP-$E_1^{2}E_4^{1}$.

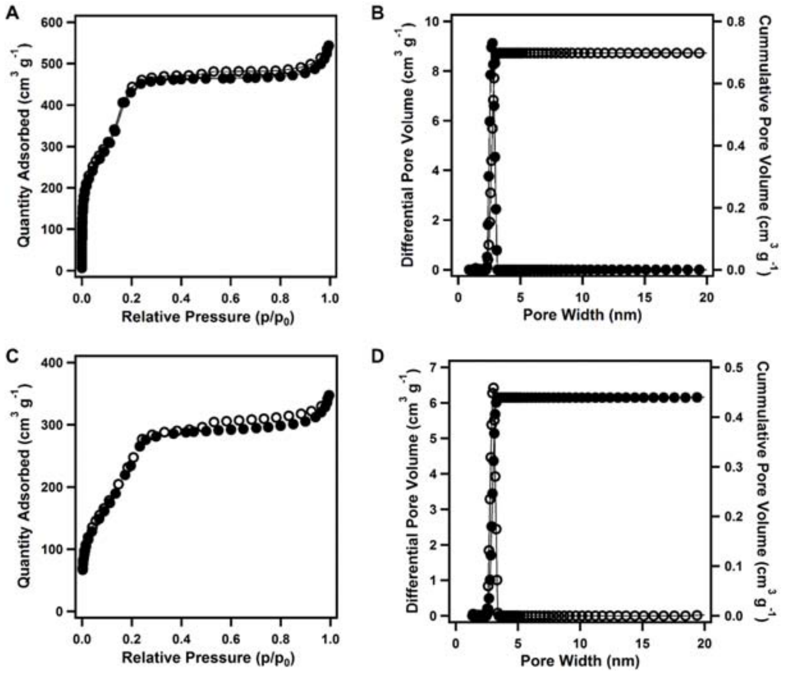

Supplementary Figure 154 | A) Nitrogen-sorption isotherm curves of MC-COF-TP-$E_1^{1}E_6^{2}$ measured at 77 K. B) Profiles of the pore size and pore-size distribution of MC-COF-TP-$E_1^{1}E_6^{2}$. C) Nitrogen-sorption isotherm curves of MC-COF-TP-$E_1^{2}E_6^{1}$ measured at 77 K. D) Profiles of the pore size and pore-size distribution of MC-COF-TP-$E_1^{2}E_6^{1}$.

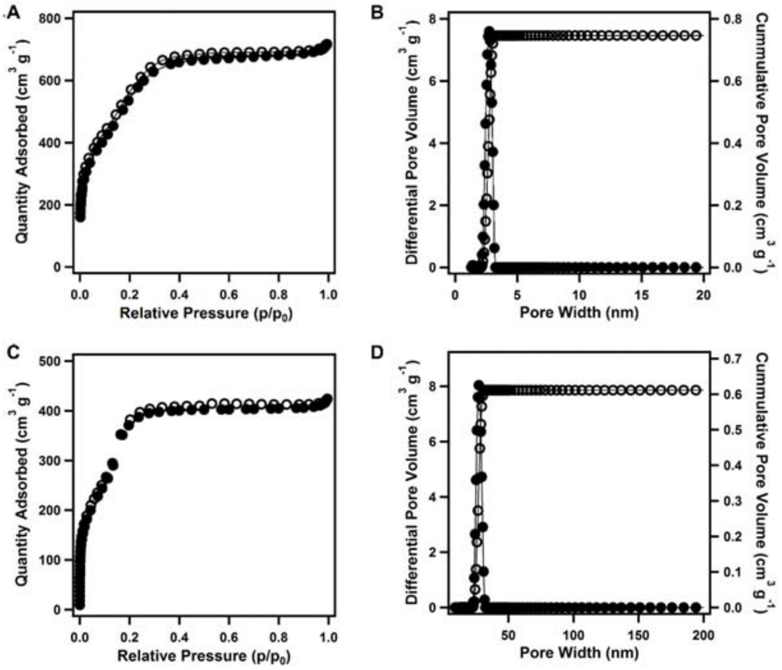

Supplementary Figure 155 | A) Nitrogen-sorption isotherm curves of MC-COF-TP-$E_1^{1}E_8^{2}$ measured at 77 K. B) Profiles of the pore size and pore-size distribution of MC-COF-TP-$E_1^{1}E_8^{2}$. C) Nitrogen-sorption isotherm curves of MC-COF-TP-$E_1^{2}E_8^{1}$ measured at 77 K. D) Profiles of the pore size and pore-size distribution of MC-COF-TP-$E_1^{2}E_8^{1}$.

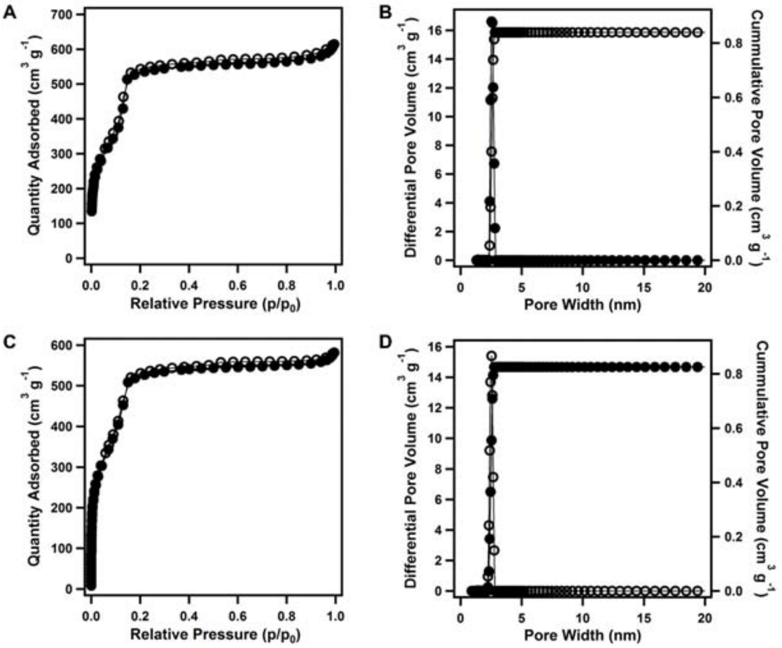

Supplementary Figure 156 | A) Nitrogen-sorption isotherm curves of MC-COF-TP-$E_2^{1}E_3^{2}$ measured at 77 K. B) Profiles of the pore size and pore-size distribution of MC-COF-TP-$E_2^{1}E_3^{2}$. C) Nitrogen-sorption isotherm curves of MC-COF-TP-$E_2^{2}E_3^{1}$ measured at 77 K. D) Profiles of the pore size and pore-size distribution of MC-COF-TP-$E_2^{2}E_3^{1}$.

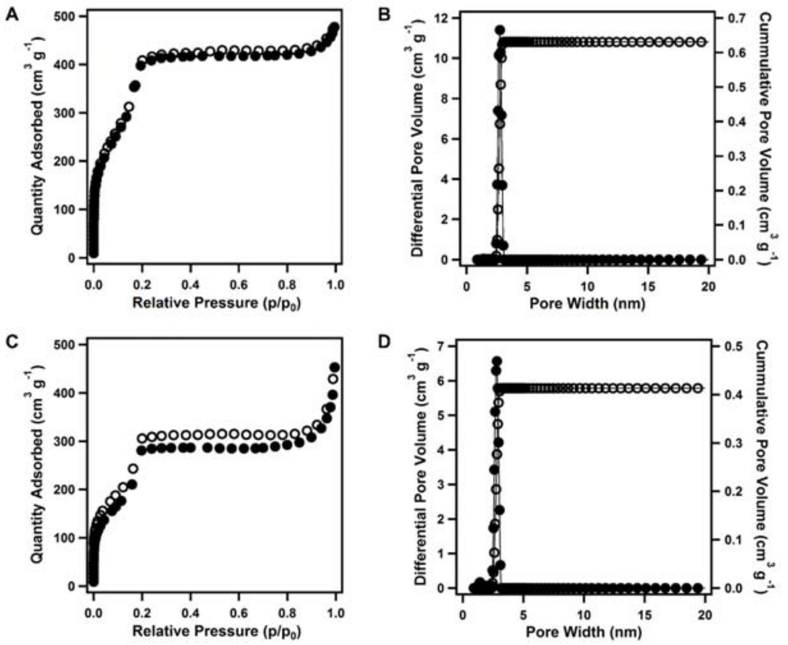

Supplementary Figure 157 | A) Nitrogen-sorption isotherm curves of MC-COF-TP-$E_2^1E_4^2$ measured at 77 K. B) Profiles of the pore size and pore-size distribution of MC-COF-TP-$E_2^1E_4^2$. C) Nitrogen-sorption isotherm curves of MC-COF-TP-$E_2^2E_4^1$ measured at 77 K. D) Profiles of the pore size and pore-size distribution of MC-COF-TP-$E_2^2E_4^1$.

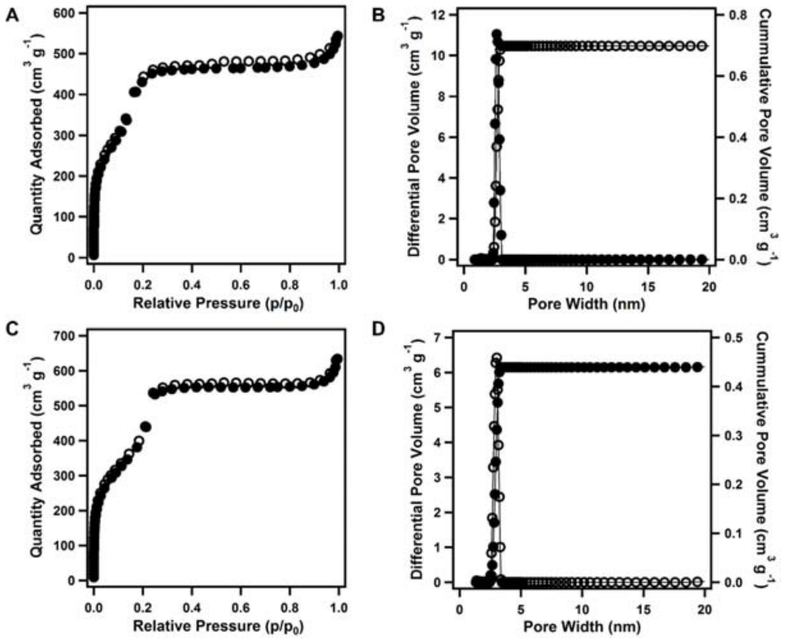

Supplementary Figure 158 | A) Nitrogen-sorption isotherm curves of MC-COF-TP-$E_2^1E_6^2$ measured at 77 K. B) Profiles of the pore size and pore-size distribution of MC-COF-TP-$E_2^1E_6^2$. C) Nitrogen-sorption isotherm curves of MC-COF-TP-$E_2^2E_6^1$ measured at 77 K. D) Profiles of the pore size and pore-size distribution of MC-COF-TP-$E_2^2E_6^1$.

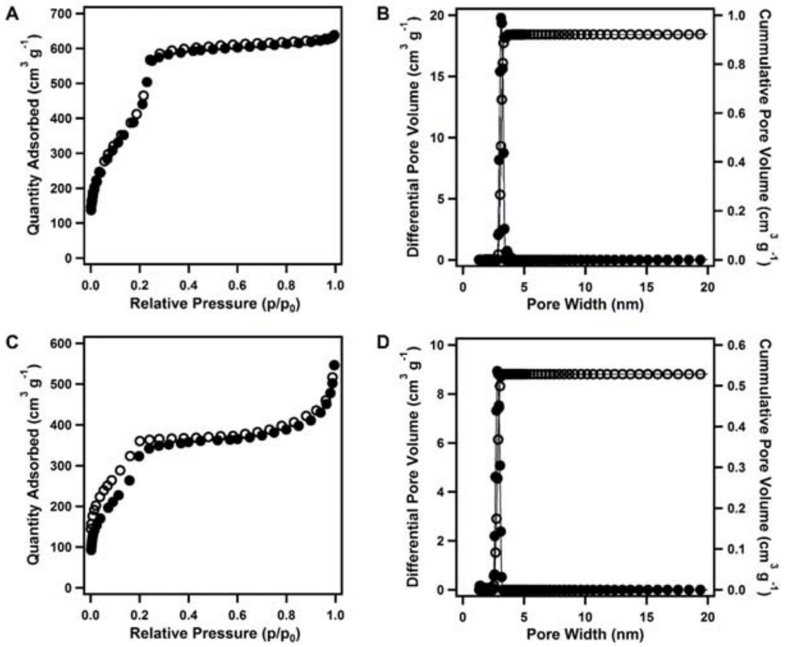

Supplementary Figure 159 | A) Nitrogen-sorption isotherm curves of MC-COF-TP-$E_2^1E_7^2$ measured at 77 K. B) Profiles of the pore size and pore-size distribution of MC-COF-TP-$E_2^1E_7^2$. C) Nitrogen-sorption isotherm curves of MC-COF-TP-$E_2^2E_7^1$ measured at 77 K. D) Profiles of the pore size and pore-size distribution of MC-COF-TP-$E_2^2E_7^1$.

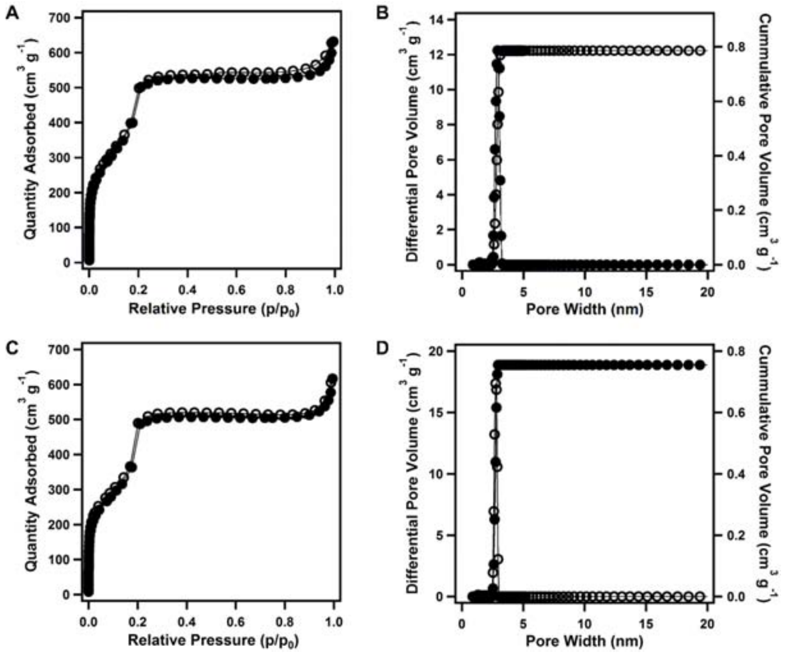

Supplementary Figure 160 | A) Nitrogen-sorption isotherm curves of MC-COF-TP-$E_3^1E_4^2$ measured at 77 K. B) Profiles of the pore size and pore-size distribution of MC-COF-TP-$E_3^1E_4^2$. C) Nitrogen-sorption isotherm curves of MC-COF-TP-$E_3^2E_4^1$ measured at 77 K. D) Profiles of the pore size and pore-size distribution of MC-COF-TP-$E_3^2E_4^1$.

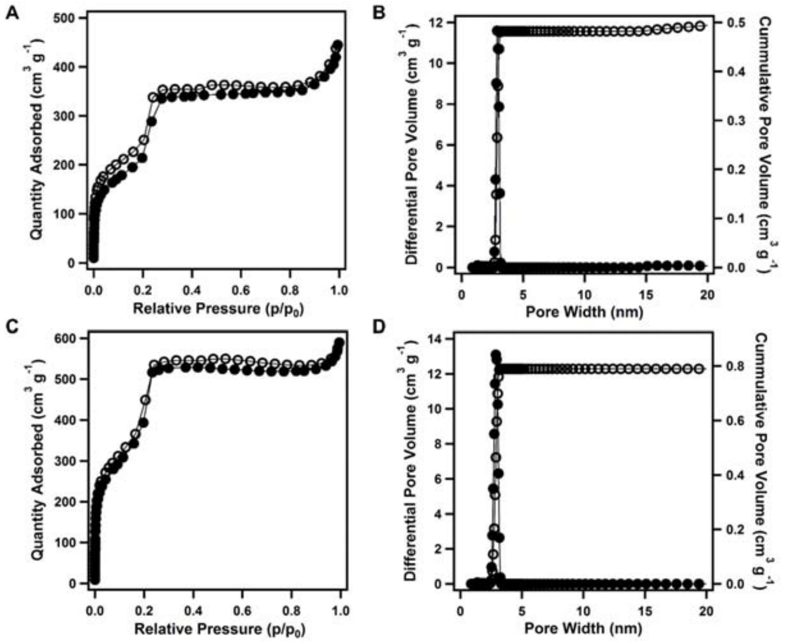

Supplementary Figure 161 | A) Nitrogen-sorption isotherm curves of MC-COF-TP-$E_3^1E_6^2$ measured at 77 K. B) Profiles of the pore size and pore-size distribution of MC-COF-TP-$E_3^1E_6^2$. C) Nitrogen-sorption isotherm curves of MC-COF-TP-$E_3^2E_6^1$ measured at 77 K. D) Profiles of the pore size and pore-size distribution of MC-COF-TP-$E_3^2E_6^1$.

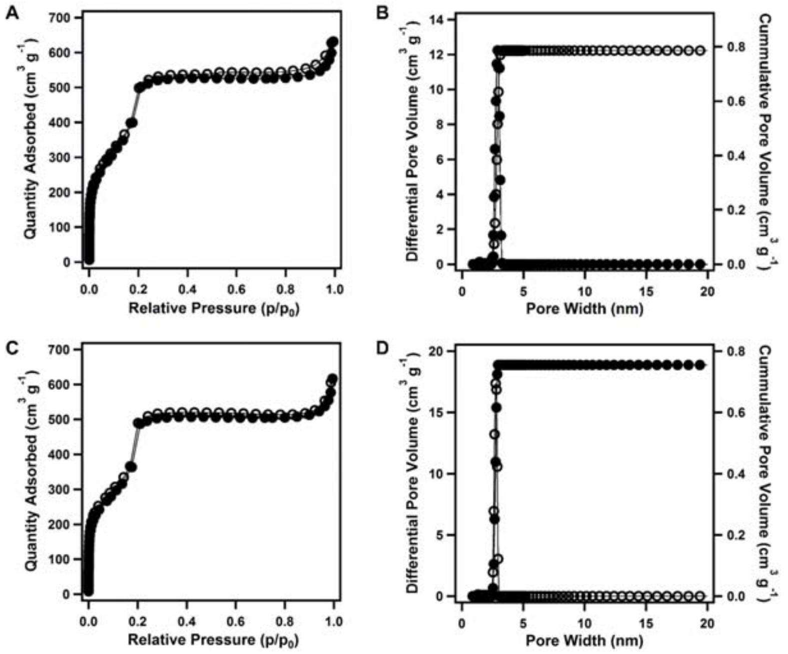

Supplementary Figure 162 | A) Nitrogen-sorption isotherm curves of MC-COF-TP-$E_3^1E_7^2$ measured at 77 K. B) Profiles of the pore size and pore-size distribution of MC-COF-TP-$E_3^1E_7^2$. C) Nitrogen-sorption isotherm curves of MC-COF-TP-$E_3^2E_7^1$ measured at 77 K. D) Profiles of the pore size and pore-size distribution of MC-COF-TP-$E_3^2E_7^1$.

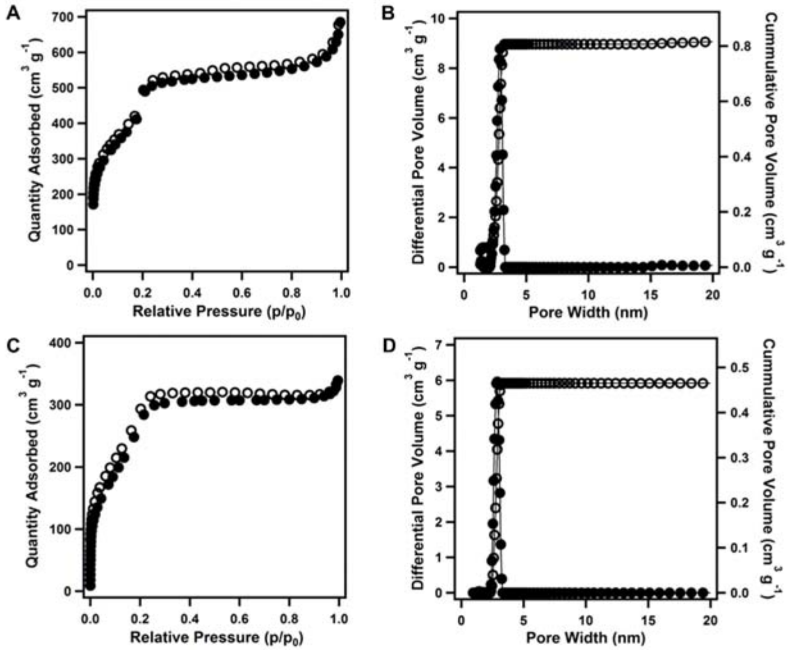

Supplementary Figure 163 | A) Nitrogen-sorption isotherm curves of MC-COF-TP-$E_3^1E_8^2$ measured at 77 K. B) Profiles of the pore size and pore-size distribution of MC-COF-TP-$E_3^1E_8^2$. C) Nitrogen-sorption isotherm curves of MC-COF-TP-$E_3^2E_8^1$ measured at 77 K. D) Profiles of the pore size and pore-size distribution of MC-COF-TP-$E_3^2E_8^1$.

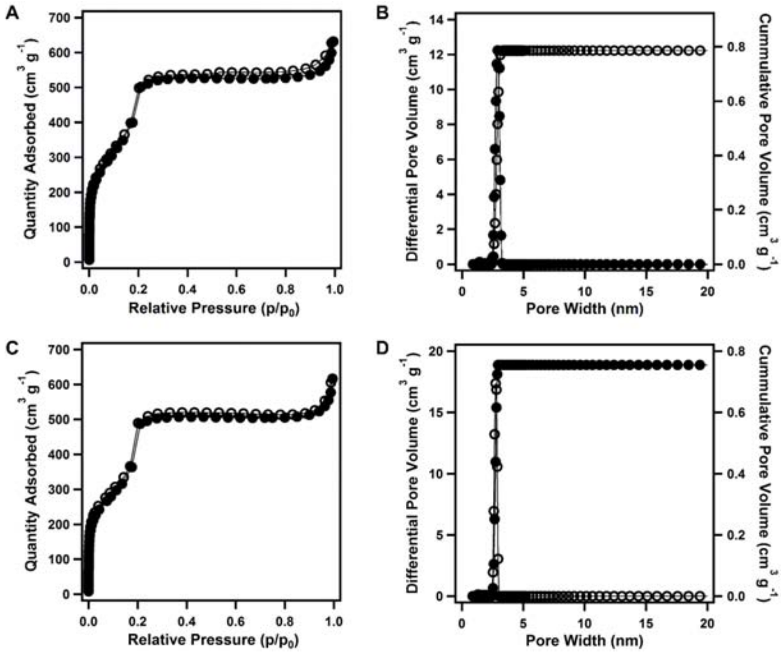

Supplementary Figure 164 | A) Nitrogen-sorption isotherm curves of MC-COF-TP-$E_4^1E_5^2$ measured at 77 K. B) Profiles of the pore size and pore-size distribution of MC-COF-TP-$E_4^1E_5^2$. C) Nitrogen-sorption isotherm curves of MC-COF-TP-$E_4^2E_5^1$ measured at 77 K. D) Profiles of the pore size and pore-size distribution of MC-COF-TP-$E_4^2E_5^1$.

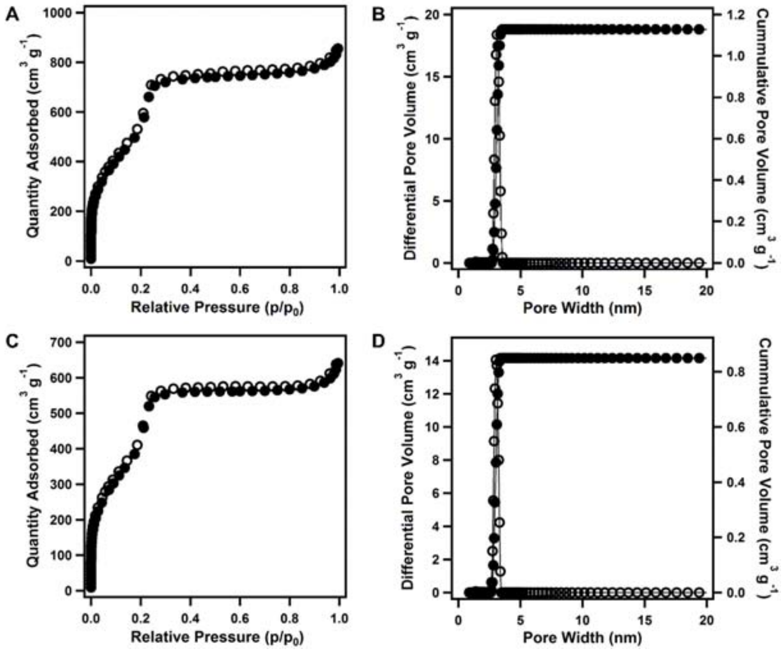

Supplementary Figure 165 | A) Nitrogen-sorption isotherm curves of MC-COF-TP-$E_4{}^1E_7{}^2$ measured at 77 K. B) Profiles of the pore size and pore-size distribution of MC-COF-TP-$E_4{}^1E_7{}^2$. C) Nitrogen-sorption isotherm curves of MC-COF-TP-$E_4{}^2E_7{}^1$ measured at 77 K. D) Profiles of the pore size and pore-size distribution of MC-COF-TP-$E_4{}^2E_7{}^1$.

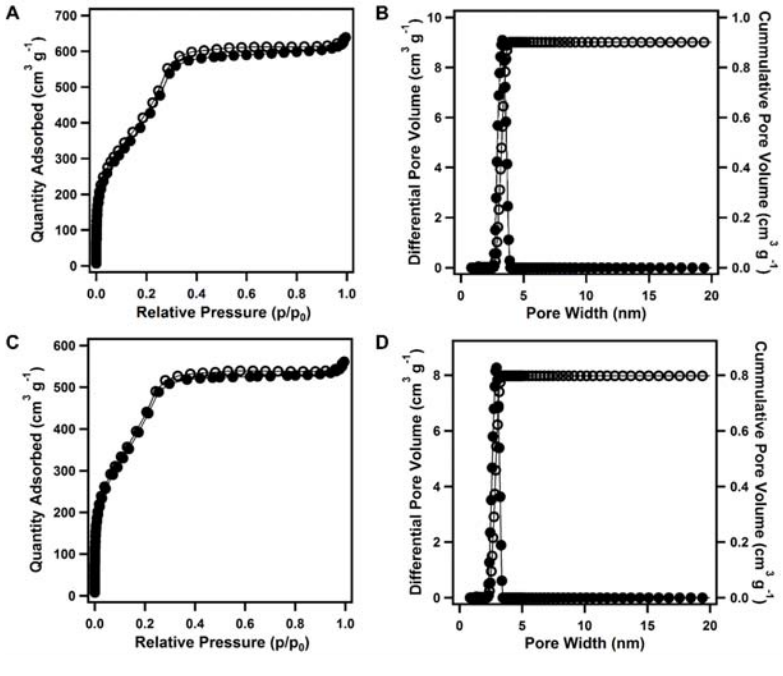

Supplementary Figure 166 | A) Nitrogen-sorption isotherm curves of MC-COF-TP-$E_4{}^1E_8{}^2$ measured at 77 K. B) Profiles of the pore size and pore-size distribution of MC-COF-TP-$E_4{}^1E_8{}^2$. C) Nitrogen-sorption isotherm curves of MC-COF-TP-$E_4{}^2E_8{}^1$ measured at 77 K. D) Profiles of the pore size and pore-size distribution of MC-COF-TP-$E_4{}^2E_8{}^1$.

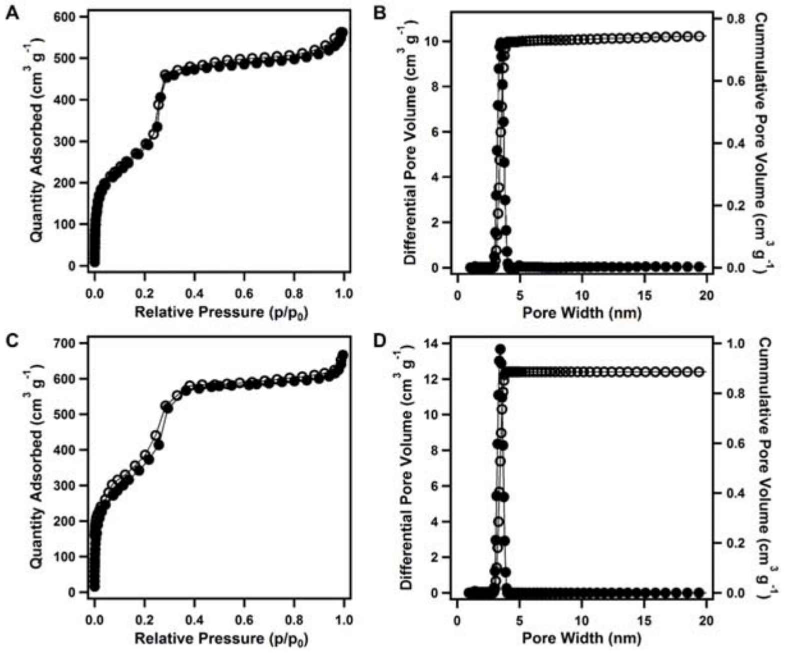

Supplementary Figure 167 | A) Nitrogen-sorption isotherm curves of MC-COF-TP-$E_5^1E_6^2$ measured at 77 K. B) Profiles of the pore size and pore-size distribution of MC-COF-TP-$E_5^1E_6^2$. C) Nitrogen-sorption isotherm curves of MC-COF-TP-$E_5^2E_6^1$ measured at 77 K. D) Profiles of the pore size and pore-size distribution of MC-COF-TP-$E_5^2E_6^1$.

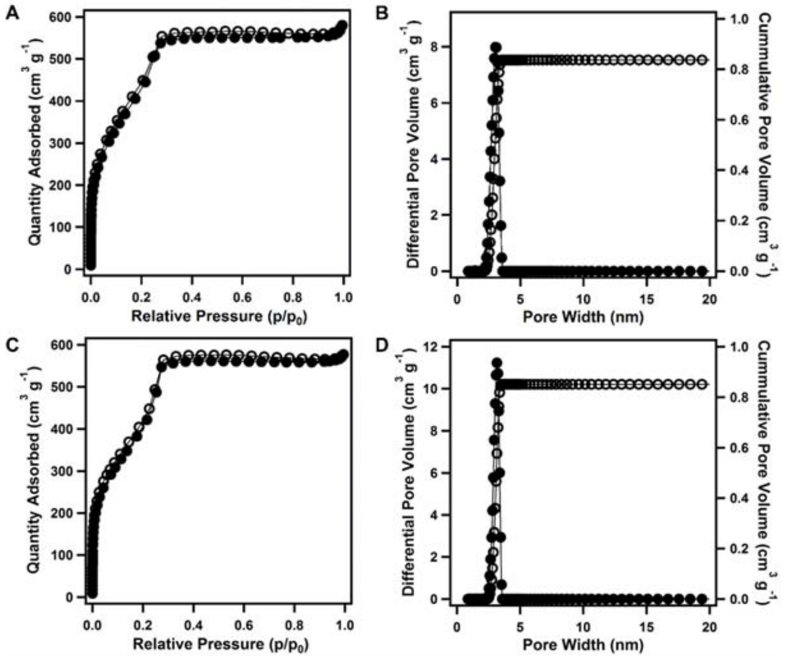

Supplementary Figure 168 | A) Nitrogen-sorption isotherm curves of MC-COF-TP-$E_5^1E_7^2$ measured at 77 K. B) Profiles of the pore size and pore-size distribution of MC-COF-TP-$E_5^1E_7^2$. C) Nitrogen-sorption isotherm curves of MC-COF- $E_5^2E_7^1$ measured at 77 K. D) Profiles of the pore size and pore-size distribution of MC-COF- $E_5^2E_7^1$.

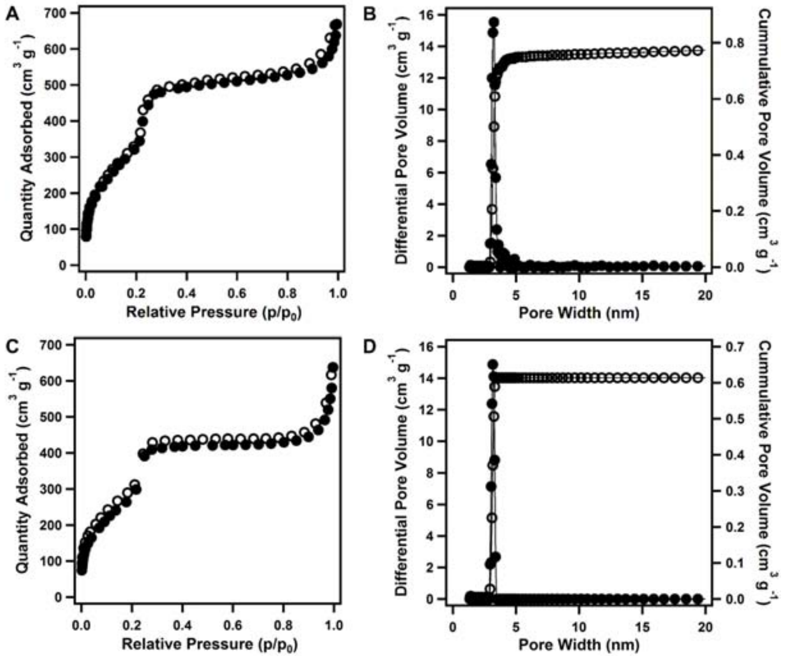

Supplementary Figure 169 | A) Nitrogen-sorption isotherm curves of MC-COF-TP-$E_5^1E_8^2$ measured at 77 K. B) Profiles of the pore size and pore-size distribution of MC-COF-TP-$E_5^1E_8^2$. C) Nitrogen-sorption isotherm curves of MC-COF- $E_5^2E_8^1$ measured at 77 K. D) Profiles of the pore size and pore-size distribution of MC-COF- $E_5^2E_8^1$.

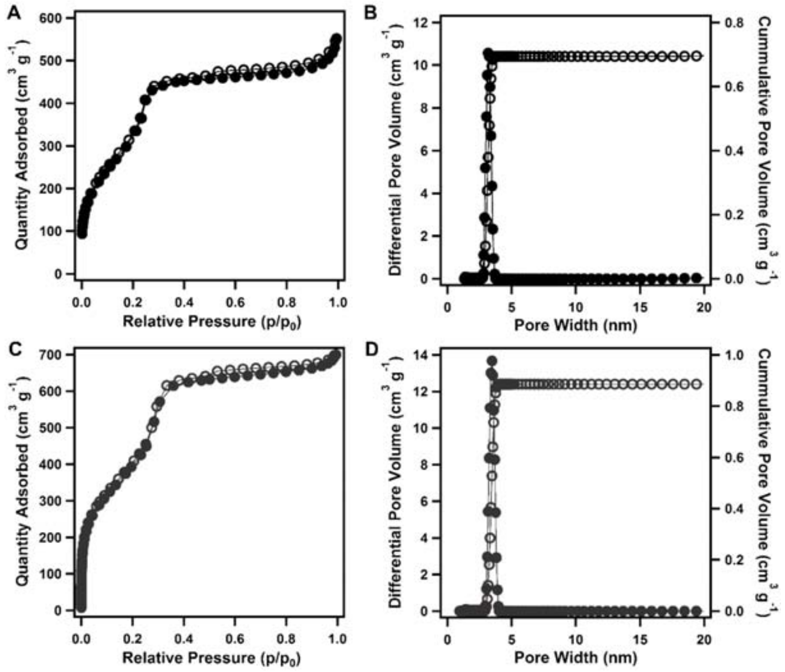

Supplementary Figure 170 | A) Nitrogen-sorption isotherm curves of MC-COF-TP-$E_6^1E_7^2$ measured at 77 K. B) Profiles of the pore size and pore-size distribution of MC-COF-TP-$E_6^1E_7^2$. C) Nitrogen-sorption isotherm curves of MC-COF- $E_6^2E_7^1$ measured at 77 K. D) Profiles of the pore size and pore-size distribution of MC-COF- $E_6^2E_7^1$.

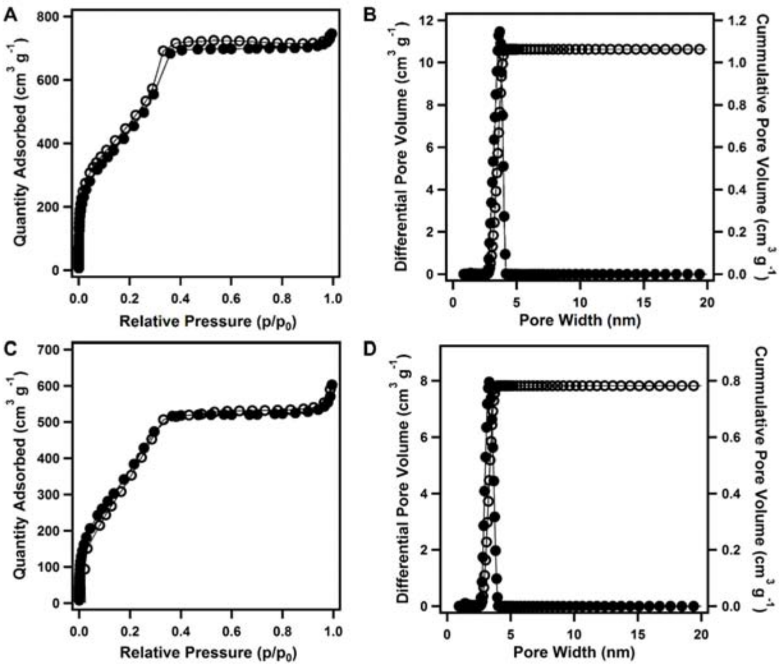

Supplementary Figure 171 | A) Nitrogen-sorption isotherm curves of MC-COF-TP-$E_7^1E_8^2$ measured at 77 K. B) Profiles of the pore size and pore-size distribution of MC-COF-TP-$E_7^1E_8^2$. C) Nitrogen-sorption isotherm curves of MC-COF- $E_7^2E_8^1$ measured at 77 K. D) Profiles of the pore size and pore-size distribution of MC-COF- $E_7^2E_8^1$.

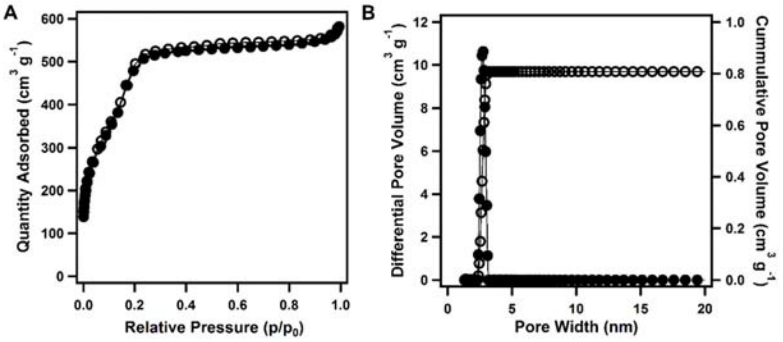

Supplementary Figure 172 | A) Nitrogen-sorption isotherm curves of MC-COF-TP-$E_1E_4E_7$ measured at 77 K. B) Profiles of the pore size and pore-size distribution of MC-COF-TP-$E_1E_4E_7$.

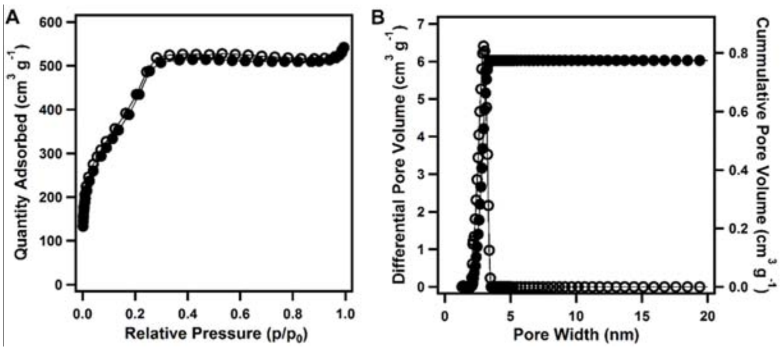

Supplementary Figure 173 | A) Nitrogen-sorption isotherm curves of MC-COF-TP-$E_4E_5E_7$ measured at 77 K. B) Profiles of the pore size and pore-size distribution of MC-COF-TP-$E_4E_5E_7$.

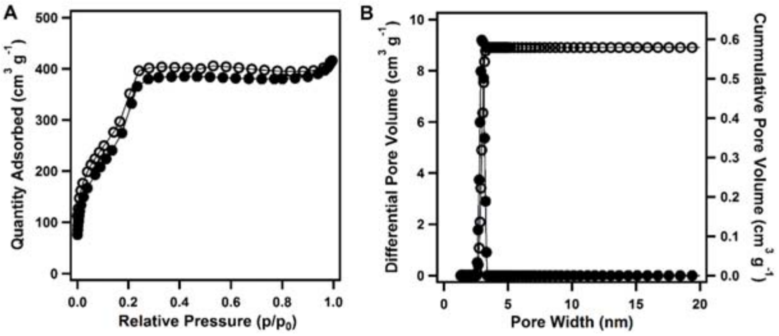

Supplementary Figure 174 | A) Nitrogen-sorption isotherm curves of MC-COF-TP-$E_4E_6E_7$ measured at 77 K. B) Profiles of the pore size and pore-size distribution of MC-COF-TP-$E_4E_6E_7$.

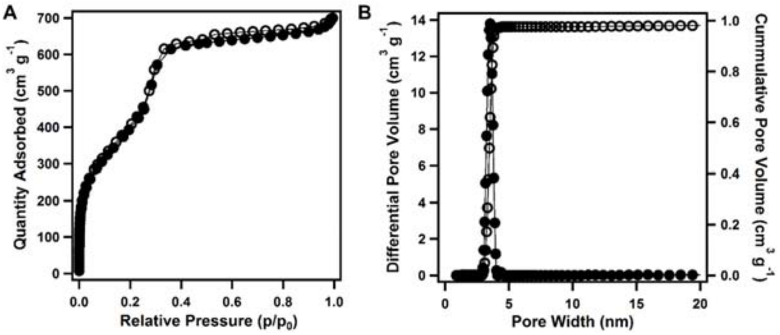

Supplementary Figure 175 | A) Nitrogen-sorption isotherm curves of MC-COF-TP-$E_5E_7E_8$ measured at 77 K. B) Profiles of the pore size and pore-size distribution of MC-COF-TP-$E_5E_7E_8$.

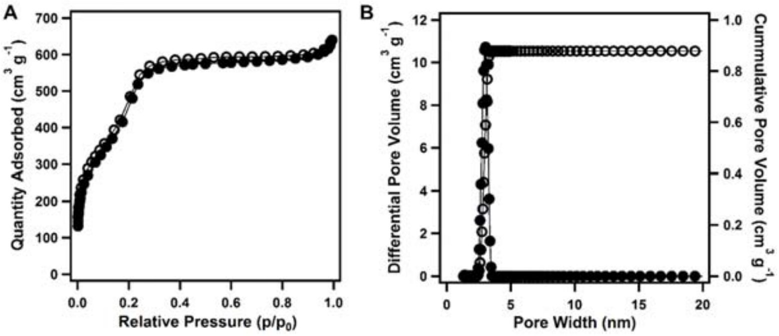

Supplementary Figure 176 | A) Nitrogen-sorption isotherm curves of MC-COF-TP-$E_3E_4E_8$ measured at 77 K. B) Profiles of the pore size and pore-size distribution of MC-COF-TP-$E_3E_4E_8$.

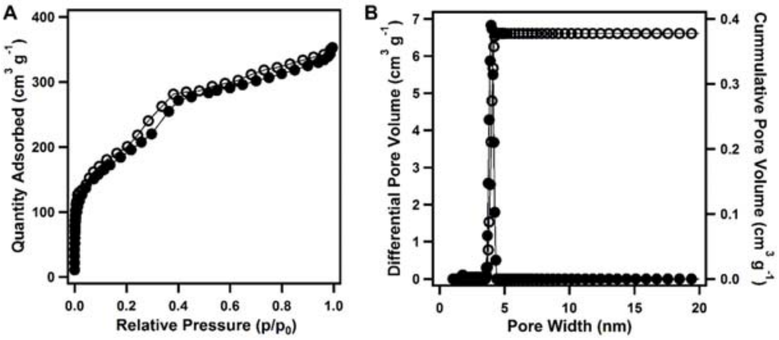

Supplementary Figure 177 | A) Nitrogen-sorption isotherm curves of MC-COF-NiPc-E_7E_9 measured at 77 K. B) Profiles of the pore size and pore-size distribution of MC-COF-NiPc-E_7E_9.

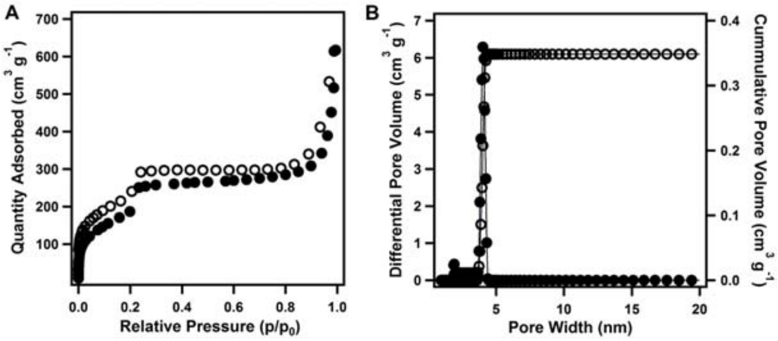

Supplementary Figure 178 | A) Nitrogen-sorption isotherm curves of MC-COF-NiPc-E_9E_{10} measured at 77 K. B) Profiles of the pore size and pore-size distribution of MC-COF-NiPc-E_9E_{10}.

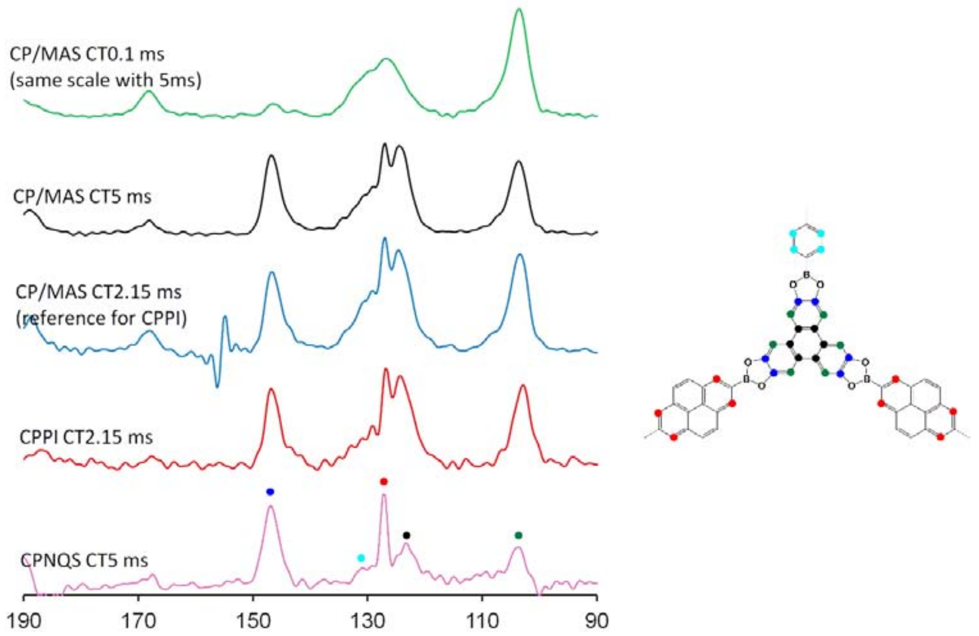

Supplementary Figure 179 | ^{13}C CP/MAS NMR spectra of MC-TP-COF-$E_1{}^1E_7{}^2$ with contact time of 0.1 ms (green curve), 5 ms (black curve), and 2.15 ms (blue curve); ^{13}C CPPI NMR spectra of MC-TP-COF-$E_1{}^1E_7{}^2$ with contact time of 2.15 ms (red curve); ^{13}C CPNQS NMR spectra of MC-TP-COF-$E_1{}^1E_7{}^2$ with contact time of 5 ms (pink curve).

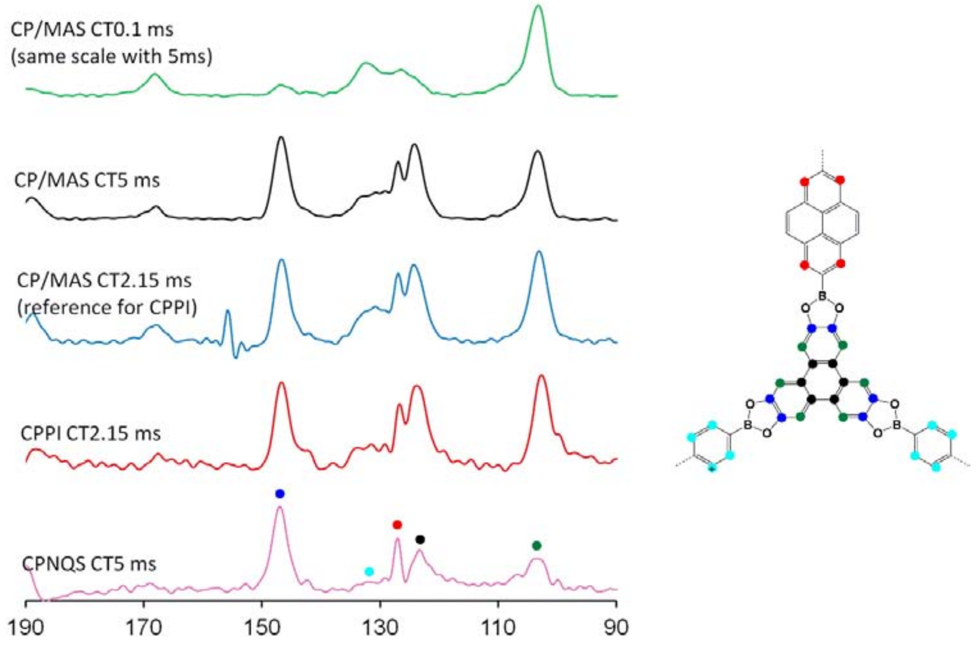

Supplementary Figure 180 | ^{13}C CP/MAS NMR spectra of MC-TP-COF-$E_1{}^2E_7{}^1$ with contact time of 0.1 ms (green curve), 5 ms (black curve), and 2.15 ms (blue curve); ^{13}C CPPI NMR spectra of MC-TP-COF-$E_1{}^2E_7{}^1$ with contact time of 2.15 ms (red curve); ^{13}C CPNQS NMR spectra of MC-TP-COF-$E_1{}^2E_7{}^1$ with contact time of 5 ms (pink curve).

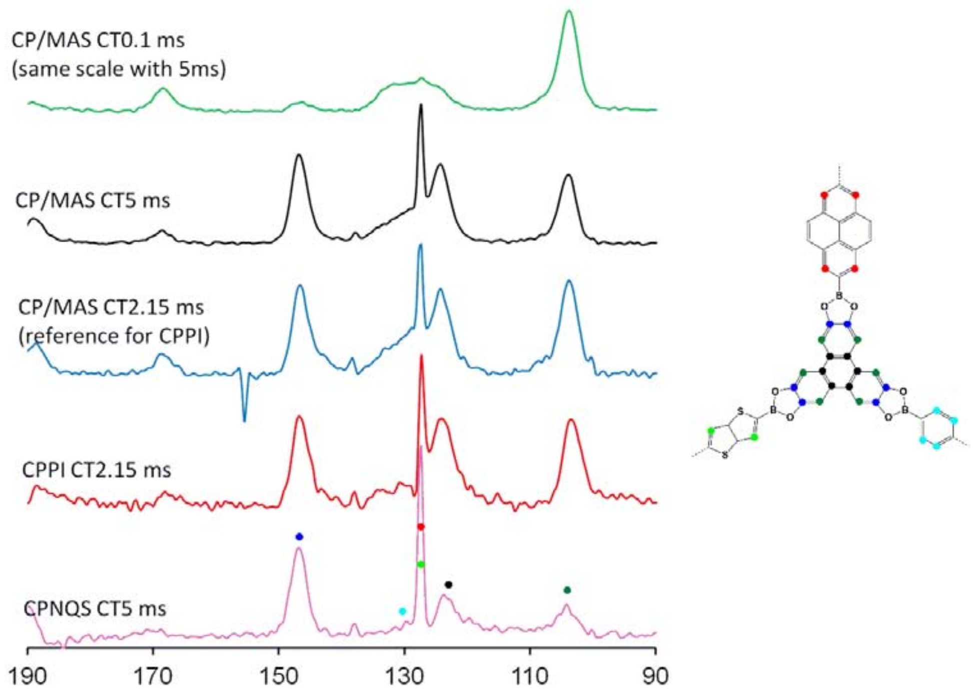

Supplementary Figure 181 | ^{13}C CP/MAS NMR spectra of MC-TP-COF-$E_1E_3E_7$ with contact time of 0.1 ms (green curve), 5 ms (black curve), and 2.15 ms (blue curve); ^{13}C CPPI NMR spectra of MC-TP-COF-$E_1E_3E_7$ with contact time of 2.15 ms (red curve); ^{13}C CPNQS NMR spectra of MC-TP-COF-$E_1E_3E_7$ with contact time of 5 ms (pink curve).

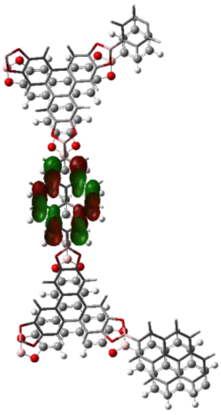 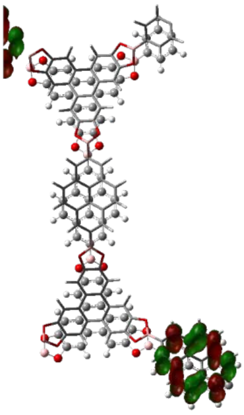

HOMO **LUMO**

Supplementary Figure 182 | Frontier molecular orbitals (MOs) of the slipped-AA MC-COF-TP-$E_1^1E_7^2$ structure. The bottom layer is depicted using ball-and-stick and the top layer is shown using tube representation.

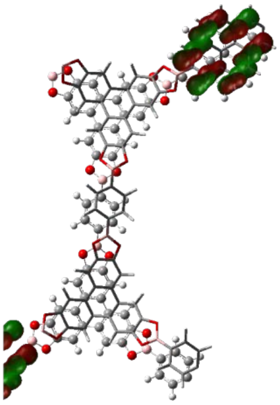 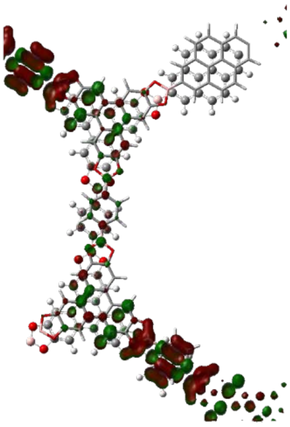

HOMO **LUMO**

Supplementary Figure 183 | Frontier molecular orbitals (MOs) of the slipped-AA MC-COF-TP-$E_1^2E_7^1$ structure. The bottom layer is depicted using ball-and-stick and the top layer is shown using tube representation.

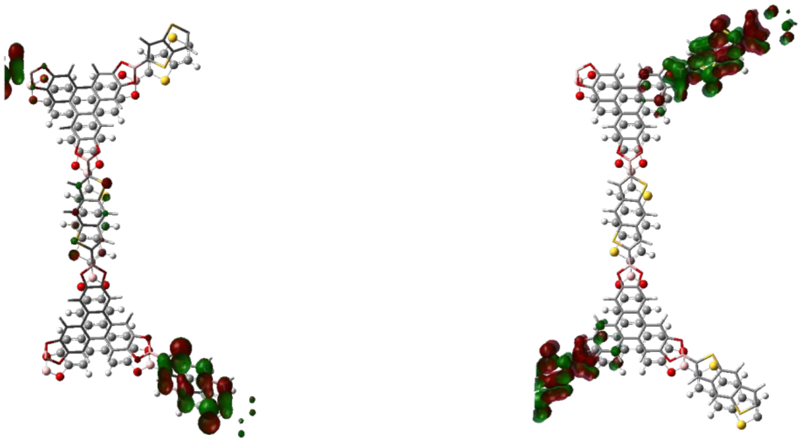

HOMO **LUMO**

Supplementary Figure 184 | Frontier molecular orbitals (MOs) of the slipped-AA MC-COF-TP-$E_3^1E_6^2$ structure. The bottom layer is depicted using ball-and-stick and the top layer is shown using tube representation.

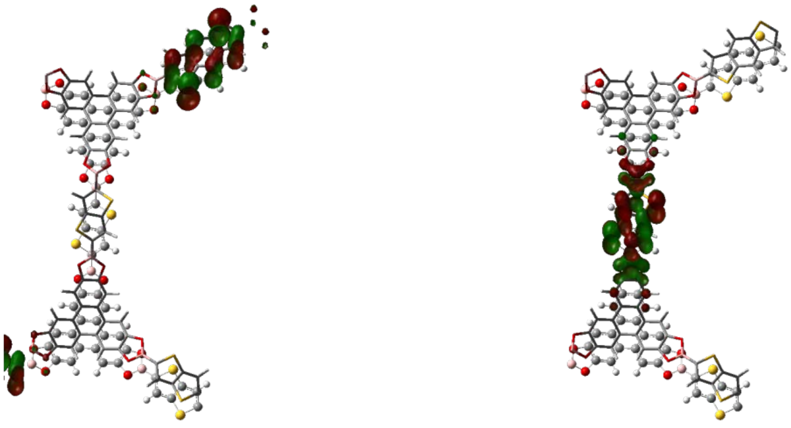

HOMO **LUMO**

Supplementary Figure 185 | Frontier molecular orbitals (MOs) of the slipped-AA MC-COF-TP-$E_3^2E_6^1$ structure. The bottom layer is depicted using ball-and-stick and the top layer is shown using tube representation.

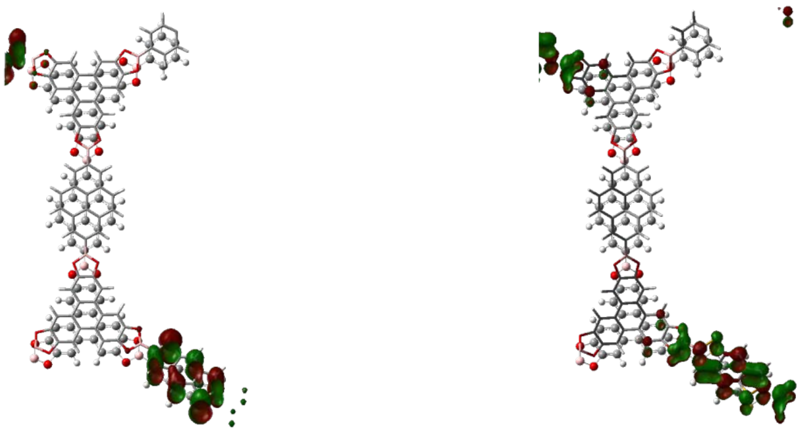

HOMO **LUMO**

Supplementary Figure 186 | Frontier molecular orbitals (MOs) of the slipped-AA MC-COF-TP-$E_1E_3E_7$ structure. The bottom layer is depicted using ball-and-stick and the top layer is shown using tube representation.

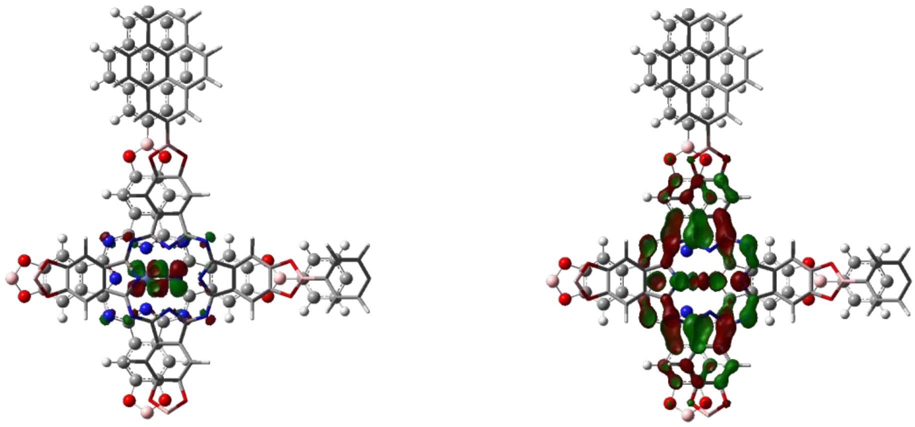

HOMO **LUMO**

Supplementary Figure 187 | Frontier molecular orbitals (MOs) of the slipped-AA MC-COF-NiPc-E_1E_7 structure. The bottom layer is depicted using ball-and-stick and the top layer is shown using tube representation.

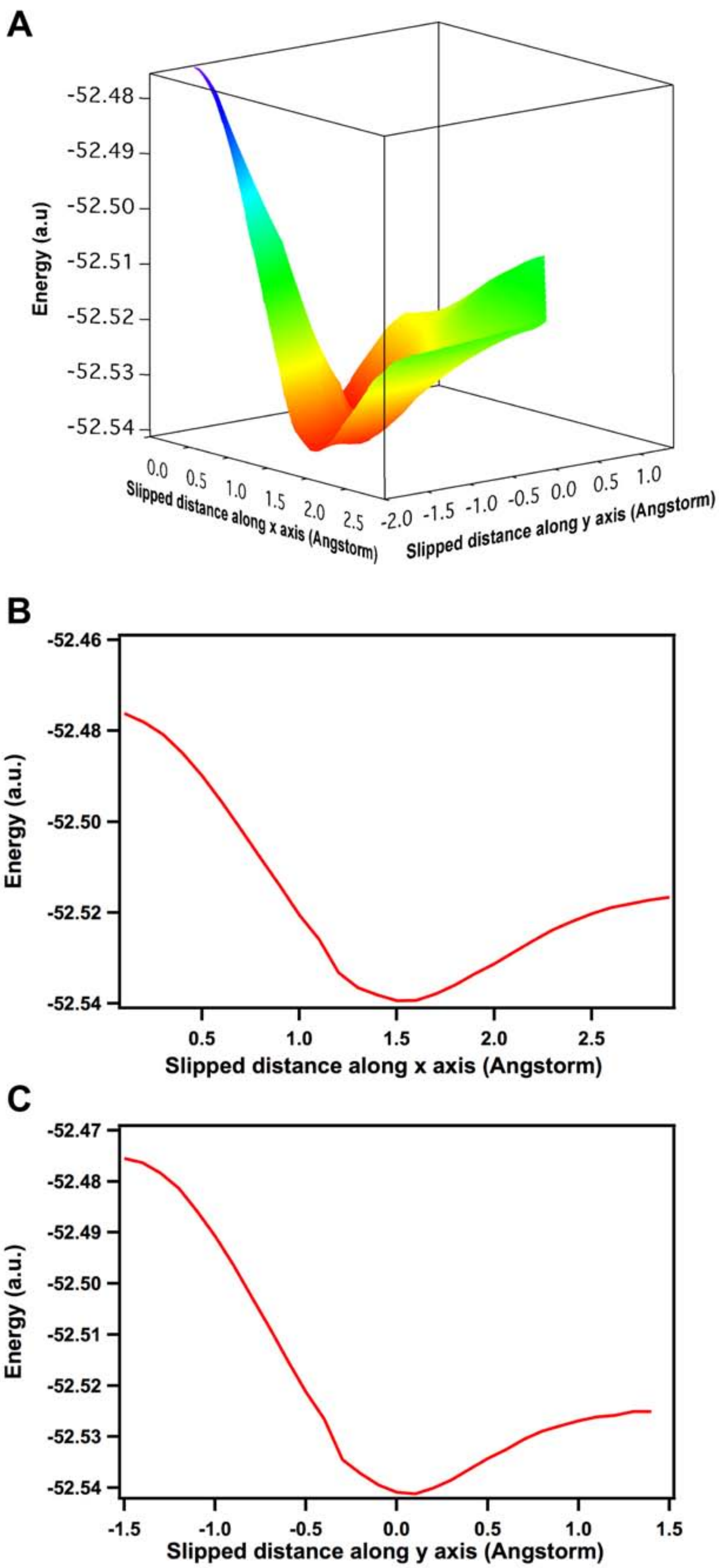

Supplementary Figure 188 | DFT scan for the MC-COF-NiPc-E_1E_7 structure along x and y directions for the energy graph in a 3D version (A), energy dependency on slipped distance along x (B) and y (C).

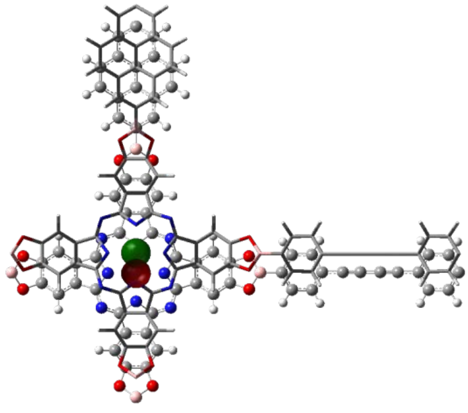

HOMO

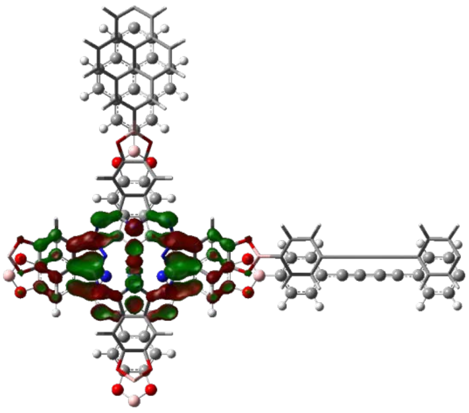

LUMO

Supplementary Figure 189 | Frontier molecular orbitals (MOs) of the slipped-AA MC-COF-NiPc-E_7E_9 structure. The bottom layer is depicted using ball-and-stick and the top layer is shown using tube representation.

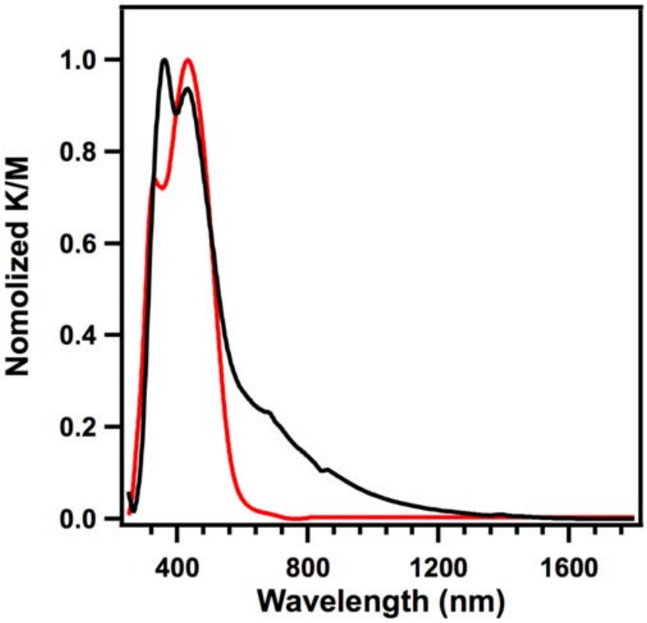

Supplementary Figure 190 | UV spectrum of MC-COF-TP-$E_1^1E_2^2$ (red) and MC-COF-TP-$E_1^2E_2^1$ (black).

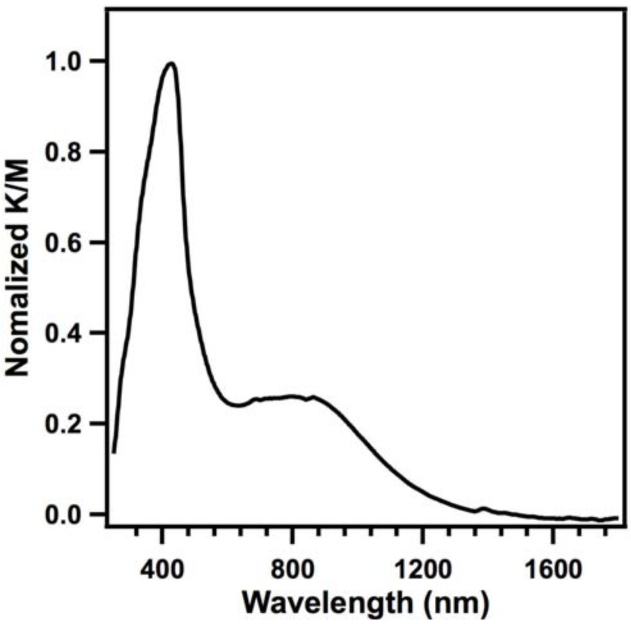

Supplementary Figure 191 | UV spectrum of MC-COF-TP-$E_2^2E_3^1$.

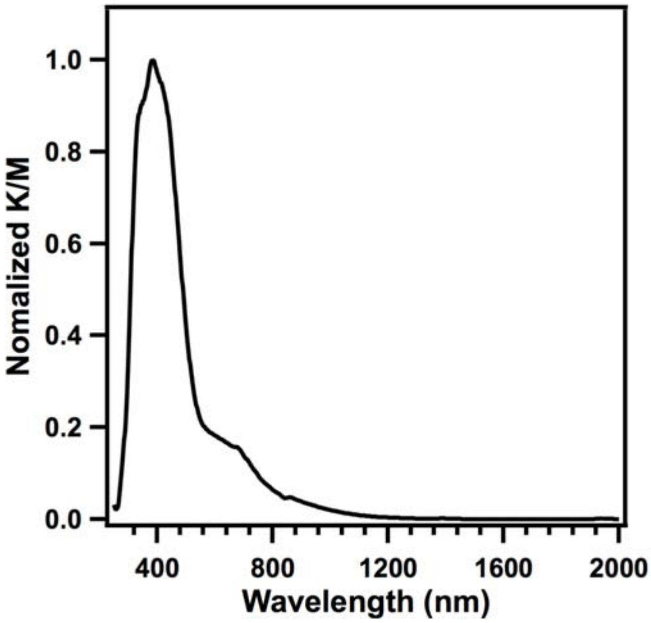

Supplementary Figure 192 | UV spectrum of MC-COF-TP-$E_2{}^2E_4{}^1$.

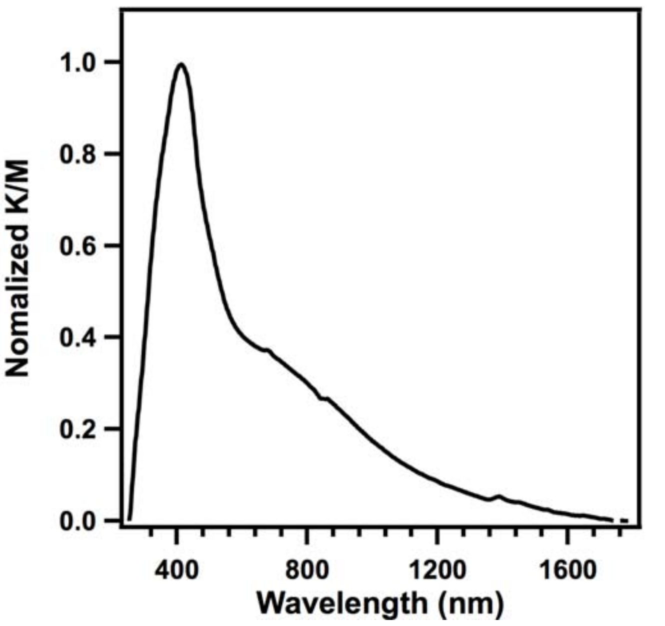

Supplementary Figure 193 | UV spectrum of MC-COF-TP-$E_2{}^2E_7{}^1$.

Suppelmentary Tables

Supplementary Table 1 | The main PXRD peak positions of MC-COFs

**MC-COFs**	**Main PXRD peaks (degree)**					
	(100)	(110)	(200)	(210)	(300)	(001)
**MC-COF-TP-$E_1^1E_2^2$**	3.35	5.24	6.71	8.96		26.3
**MC-COF-TP-$E_1^2E_2^1$**	3.42	5.28	6.74	8.98		26.2
**MC-COF-TP-$E_1^1E_3^2$**	3.28	5.76	6.68	9.13		26.1
**MC-COF-TP-$E_1^2E_3^1$**	3.38	5.83	6.83	9.05		26.3
**MC-COF-TP-$E_1^1E_4^2$**	3.16	5.68	6.62	8.93		26.3
**MC-COF-TP-$E_1^2E_4^1$**	3.40	5.92	6.79	8.75		26.4
**MC-COF-TP-$E_1^1E_6^2$**	2.77	5.32	6.38	8.53		26.5
**MC-COF-TP-$E_1^2E_6^1$**	3.21	5.67	6.75	8.82		26.4
**MC-COF-TP-$E_1^1E_7^2$**	2.76	4.90	5.38	7.52		26.1
**MC-COF-TP-$E_1^2E_7^1$**	2.94	5.32	5.84	7.84		26.4
**MC-COF-TP-$E_1^1E_8^2$**	2.81	4.74	5.17	7.31		26.3
**MC-COF-TP-$E_1^2E_8^1$**	3.14	5.13	5.63	7.57		26.4
**MC-COF-TP-$E_2^1E_3^2$**	3.26	5.76	6.72	9.16		26.5
**MC-COF-TP-$E_2^2E_3^1$**	3.37	5.82	6.85	9.05		26.4
**MC-COF-TP-$E_2^1E_4^2$**	3.15	5.67	6.64	8.95		26.4
**MC-COF-TP-$E_2^2E_4^1$**	3.42	5.94	6.81	8.77		26.5
**MC-COF-TP-$E_2^1E_6^2$**	2.75	5.31	6.35	8.49		26.6
**MC-COF-TP-$E_2^2E_6^1$**	3.18	5.65	6.72	8.80		26.4
**MC-COF-TP-$E_3^1E_4^2$**	3.08	5.56	6.36	8.46		26.3
**MC-COF-TP-$E_3^2E_4^1$**	3.16	5.47	6.61	8.78		26.5
**MC-COF-TP-$E_3^1E_6^2$**	2.78	5.26	5.86	8.34		26.4
**MC-COF-TP-$E_3^2E_6^1$**	3.09	5.43	6.16	7.96		26.3

**MC-COF-TP-$E_3^1E_7^2$**	5.76	5.03	5.84	7.84		26.3
**MC-COF-TP-$E_3^2E_7^1$**	6.13	5.43	6.15	8.11		26.5
**MC-COF-TP-$E_3^1E_8^2$**	2.56	5.04	5.92	7.85		26.4
**MC-COF-TP-$E_3^2E_8^1$**	2.84	5.38	6.13	8.13		26.4
**MC-COF-TP-$E_4^1E_5^2$**	2.66	4.87	5.52	7.97		26.4
**MC-COF-TP-$E_4^2E_5^1$**	2.83	5.13	5.85	8.21		26.4
**MC-COF-TP-$E_4^1E_7^2$**	2.77	4.89	5.87	7.78		26.5
**MC-COF-TP-$E_4^2E_7^1$**	2.95	5.11	6.14	8.10		26.3
**MC-COF-TP-$E_4^1E_8^2$**	2.47	4.67	5.67	7.56		26.4
**MC-COF-TP-$E_4^2E_8^1$**	2.81	5.12	6.01	7.97		26.4
**MC-COF-TP-$E_5^1E_6^2$**	2.52	4.75	5.16	7.17		26.3
**MC-COF-TP-$E_5^2E_6^1$**	2.54	4.91	5.31	7.41		26.5
**MC-COF-TP-$E_5^1E_7^2$**	2.64	4.65	5.13	7.31		26.4
**MC-COF-TP-$E_5^2E_7^1$**	2.67	4.71	5.34	7.42		26.3
**MC-COF-TP- $E_5^1E_8^2$**	2.38	4.59	5.03	7.22		26.4
**MC-COF-TP- $E_5^2E_8^1$**	2.46	4.67	5.23	7.33		26.3
**MC-COF-TP- $E_6^1E_7^2$**	2.63	5.75	5.44	7.36		26.4
**MC-COF-TP- $E_6^2E_7^1$**	2.67	5.87	5.45	7.39		26.4
**MC-COF-TP-$E_1E_3E_7$**	2.84	4.92	5.58	8.47		26.2
**MC-COF-TP-$E_1E_4E_7$**	3.04	5.38	6.31	8.42		26.6
**MC-COF-TP-$E_4E_5E_7$**	2.74	4.73	5.13	7.22		26.5
**MC-COF-TP-$E_4E_6E_7$**	2.61	5.10	5.47	7.62		26.7
**MC-COF-TP-$E_5E_7E_8$**	2.54	4.65	5.13	7.22		26.6
**MC-COF-TP-$E_3E_4E_7$**	2.96	4.83	5.51	7.43		26.6
**MC-COF-NiPc-E_1E_7**	3.72		7.64		11.13	26.7
**MC-COF-NiPc-E_7E_9**	3.17		6.43		9.13	26.8
**MC-COF-NiPc-E_9E_{10}**	2.49		5.05		7.65	26.9

Supplementary Table 2 | Elemental analysis of MC-COFs

MC-COFs		C%	H%	N%	S%
MC-COF-TP-$E_1^1E_2^2$	Calcd.	67.88	2.53	4.40	5.03
	Found	65.34	2.95	4.12	4.77
MC-COF-TP-$E_1^2E_2^1$	Calcd.	71.12	2.82	2.30	2.64
	Found	69.82	3.11	2.05	2.43
MC-COF-TP-$E_1^1E_3^2$	Calcd.	71.72	3.70	-	4.91
	Found	70.51	4.12	-	4.81
MC-COF-TP-$E_1^2E_3^1$	Calcd.	68.37	3.53	-	9.36
	Found	66.31	3.86	-	8.89
MC-COF-TP-$E_1^1E_4^2$	Calcd.	76.38	3.20	-	-
	Found	74.68	3.64	-	-
MC-COF-TP-$E_1^2E_4^1$	Calcd.	75.57	3.17	-	-
	Found	73.89	3.64	-	-
MC-COF-TP-$E_1^1E_6^2$	Calcd.	69.52	2.63	-	9.28
	Found	67.34	2.92	-	9.05
MC-COF-TP-$E_1^2E_6^1$	Calcd.	71.87	2.86	-	5.05
	Found	69.52	3.16	-	4.85
MC-COF-TP-$E_1^1E_7^2$	Calcd.	77.36	3.84	-	-
	Found	74.12	4.18	-	-
MC-COF-TP-$E_1^2E_7^1$	Calcd.	78.58	3.15	-	-
	Found	76.34	3.42	-	-
MC-COF-TP-$E_1^1E_8^2$	Calcd.	76.38	3.20	-	-
	Found	74.16	3.66	--	-
MC-COF-TP-$E_1^2E_8^1$	Calcd.	77.82	3.27	-	-
	Found	73.56	3.81	-	-
MC-COF-TP-$E_2^1E_3^2$	Calcd.	64.53	2.26	2.09	11.96
	Found	62.25	2.51	1.96	10.62
MC-COF-TP-$E_2^2E_3^1$	Calcd.	67.66	2.52	2.19	7.53
	Found	65.34	2.86	1.96	7.23
MC-COF-TP-$E_2^1E_4^2$	Calcd.	73.01	2.91	2.13	2.44
	Found	71.64	3.16	2.05	2.13
MC-COF-TP-$E_2^2E_4^1$	Calcd.	72.10	2.87	2.21	2.53
	Found	70.38	3.06	2.11	2.23
MC-COF-TP-$E_2^1E_6^2$	Calcd.	66.62	2.52	1.94	11.11
	Found	63.25	2.87	1.67	10.87
MC-COF-TP-$E_2^2E_6^1$	Calcd.	65.85	2.33	4.04	9.25
	Found	63.15	2.65	3.73	8.84
MC-COF-TP-$E_3^1E_4^2$	Calcd.	73.45	3.73	-	4.56
	Found	71.14	3.97	-	4.21
MC-COF-TP-$E_3^2E_4^1$	Calcd.	69.34	3.55	-	9.03
	Found	67.12	4.01	-	8.72

**MC-COF-TP-$E_3^1E_6^2$**	Calcd.	65.28	2.60	-	13.76
	Found	62.34	3.04	-	12.67
**MC-COF-TP-$E_3^2E_6^1$**	Calcd.	66.43	2.51	-	13.30
	Found	63.26	2.97	-	11.35
**MC-COF-TP-$E_3^1E_7^2$**	Calcd.	70.82	3.83	-	8.59
	Found	67.64	4.26	-	8.14
**MC-COF-TP-$E_3^2E_7^1$**	Calcd.	75.72	3.63	-	4.12
	Found	71.67	4.13	-	3.87
**MC-COF-TP-$E_3^1E_8^2$**	Calcd.	75.05	3.62	-	4.26
	Found	72.35	4.16	-	3.98
**MC-COF-TP-$E_3^2E_8^1$**	Calcd.	70.34	3.43	-	8.73
	Found	67.66	3.85	-	8.52
**MC-COF-TP-$E_4^1E_5^2$**	Calcd.	78.17	4.05	-	-
	Found	76.61	4.57	-	-
**MC-COF-TP-$E_4^2E_5^1$**	Calcd.	78.87	3.92	-	-
	Found	75.64	4.37	-	-
**MC-COF-TP-$E_4^1E_7^2$**	Calcd.	79.53	3.80	-	-
	Found	76.31	4.25	-	-
**MC-COF-TP-$E_4^2E_7^1$**	Calcd.	78.21	3.21	-	-
	Found	74.64	3.87	-	-
**MC-COF-TP-$E_4^1E_8^2$**	Calcd.	78.47	3.29	-	-
	Found	76.15	3.85	-	-
**MC-COF-TP-$E_4^2E_8^1$**	Calcd.	78.53	3.98	-	-
	Found	75.64	4.38	-	-
**MC-COF-TP-$E_5^1E_6^2$**	Calcd.	70.67	3.87	-	8.48
	Found	67.16	4.35	-	-
**MC-COF-TP-$E_5^2E_6^1$**	Calcd.	74.44	3.94	-	4.32
	Found	72.16	4.36	-	-
**MC-COF-TP-$E_5^1E_7^2$**	Calcd.	79.69	3.92	-	-
	Found	75.65	4.43	-	-
**MC-COF-TP-$E_5^2E_7^1$**	Calcd.	79.10	3.98	-	-
	Found	76.26	4.38	-	-
**MC-COF-TP-$E_5^1E_8^2$**	Calcd.	79.35	3.33	-	-
	Found	76.16	3.91	-	-
**MC-COF-TP-$E_5^2E_8^1$**	Calcd.	78.72	3.37	-	-
	Found	75.63	4.06	-	-
**MC-COF-TP-$E_6^1E_7^2$**	Calcd.	76.03	3.03	-	4.18
	Found	73.56	3.67	-	4.05
**MC-COF-TP-$E_6^2E_7^1$**	Calcd.	71.76	2.68	-	8.51
	Found	69.16	3.05	-	8.16
**MC-COF-TP-$E_1E_3E_7$**	Calcd.	73.91	3.66	-	4.48
	Found	71.25	4.05	-	4.26
**MC-COF-TP-$E_1E_4E_7$**	Calcd.	78.02	3.84	-	

	Found	74.55	4.46	-	-
**MC-COF-TP-$E_4E_5E_7$**	Calcd.	78.87	3.92	-	-
	Found	75.55	4.38	-	-
**MC-COF-TP-$E_4E_6E_7$**	Calcd.	75.44	3.56	-	4.20
	Found	71.55	4.16	-	3.84
**MC-COF-TP-$E_5E_7E_8$**	Calcd.	74.43	3.92	-	-
	Found	71.64	4.38	-	-
**MC-COF-TP-$E_3E_4E_7$**	Calcd.	74.22	3.74	-	4.40
	Found	71.86	4.06	-	4.16
**MC-COF-NiPc-E_1E_7**	Calcd.	72.65	3.59	6.65	-
	Found	71.25	4.16	6.35	-
**MC-COF-NiPc-E_7E_9**	Calcd.	74.31	3.56	6.19	-
	Found	72.55	4.06	5.87	-
**MC-COF-NiPc-E_9E_{10}**	Calcd.	75.13	3.63	5.94	-
	Found	73.43	4.05	5.61	-

Supplementary Table 3 | Ratio of the links determined for MC-COF crystals and their porosity (shown in parentheses)

COFs	S_{BET} (m^2 g^{-1})	Pore size (nm)	V_{total} (cm^3 g^{-1})	Ratio
MC-COF-TP-E_1E_2	1862 / 1984	2.8 / 2.7	0.74 / 0.83	1:2 / 2:1
MC-COF-TP-E_1E_3	1634 / 1900	3.0 / 2.8	0.59 / 0.77	1:2 / 2:1
MC-COF-TP-E_1E_4	2054 / 1962	3.2 / 2.9	1.01 / 0.86	1:2 / 2:1
MC-COF-TP-E_1E_6	1535 / 1206	3.2 / 2.9	0.68 / 0.44	1:2 / 2:1
MC-COF-TP-E_1E_7	1892 / 1534	3.2 / 2.8	0.76 / 0.61	1:2 / 2:1
MC-COF-TP-E_1E_8	1965 / 1376	3.6 / 3.1	1.05 / 0.56	1:2 / 2:1
MC-COF-TP-E_2E_3	1955 / 1931	2.8 / 2.7	0.82 / 0.81	1:2 / 2:1
MC-COF-TP-E_2E_4	1544 / 1136	2.9 / 2.7	0.63 / 0.41	1:2 / 2:1
MC-COF-TP-E_2E_6	1768 / 1267	2.9 / 2.8	0.70 / 0.44	1:2 / 2:1
MC-COF-TP-E_3E_4	1543 / 1261	3.0 / 2.9	0.63 / 0.42	1:2 / 2:1
MC-COF-TP-E_3E_6	1531 / 1824	2.9 / 2.7	0.50 / 0.80	1:2 / 2:1
MC-COF-TP-E_3E_7	1863 / 1809	3.2 / 2.8	0.79 / 0.76	1:2 / 2:1
MC-COF-TP-E_3E_8	1713 / 1221	3.8 / 3.4	0.81 / 0.47	1:2 / 2:1
MC-COF-TP-E_4E_5	1834 / 1786	3.0 / 2.7	0.80 / 0.77	1:2 / 2:1
MC-COF-TP-E_4E_7	1965 / 1902	3.3 / 3.1	1.15 / 0.84	1:2 / 2:1
MC-COF-TP-E_4E_8	1935 / 1762	4.2 / 3.9	0.90 / 0.80	1:2 / 2:1
MC-COF-TP-E_5E_6	1782 / 1913	4.4 / 3.8	0.74 / 0.88	1:2 / 2:1
MC-COF-TP-E_5E_7	1832 / 1768	4.4 / 3.7	0.82 / 0.83	1:2 / 2:1
MC-COF-TP-E_5E_8	1756 / 1642	4.4 / 4.0	0.78 / 0.61	1:2 / 2:1
MC-COF-TP-E_6E_7	1714 / 1854	4.2 / 3.9	0.70 / 0.87	1:2 / 2:1
MC-COF-TP-$E_1E_3E_7$	1887	2.9	0.91	1:1:1
MC-COF-TP-$E_1E_4E_7$	1867	3.0	0.81	1:1:1
MC-COF-TP-$E_4E_5E_7$	1787	4.2	0.78	1:1:1
MC-COF-TP-$E_4E_6E_7$	1315	3.6	0.58	1:1:1
MC-COF-TP-$E_5E_7E_8$	1966	3.3	1.00	1:1:1
MC-COF-TP-$E_3E_4E_7$	1813	3.2	0.87	1:1:1
MC-COF-NiPc-E_1E_7	672	2.6	0.34	1:1
MC-COF-NiPc-E_7E_9	765	4.5	0.37	1:1
MC-COF-NiPc-E_9E_{10}	512	4.8	0.35	1:1

Supplementary Table 4 | Summary of ^{13}C CP/MAS NMR of selected MC-COFs and mixtures of monomers

Compounds	Chemical Shift (ppm)				
Selected MC-COFs					
MC-COF-TP-$E_1^1E_7^2$	147.5	131.5	127.4	123.5	104.7
MC-COF-TP-$E_1^2E_7^1$	147.1	131.7	126.9	125.2	103.6
MC-COF-TP-$E_1E_3E_7$	146.4	131.1	128.2	124.8	104.4
Mixtures of Monomers					
TP, E_1, E_3, E_7	144.3	132.2	128.7	125.3	105.1

Supplementary Table 5 | The total DFTB energies, Lennard-Jones contributions (LJ), and the crystal stacking energies per monolayer as well as the corresponding HOMO-LUMO energy gap for MC-COF-TP-$E_1^1E_7^2$

**Stacking mode**	***c*** **(Å)**	**Total DFTB Energy (a.u.)**	**LJ energy (a.u.)**	**Per unit crystal stacking energy (kcal mol^{-1})**	**HOMO-LUMO gap (eV)**
**monolayer**		-205.382398	0.872		2.534
**AA**	3.51	-411.149406	1.346	-120.67	2.174
**AB**	3.25	-410.972105	1.560	-65.04	2.515
**slipAA-*a***	3.45	-411.161217	1.343	-124.38	2.340
**slipAA-*ab***	3.43	-411.162374	1.345	-124.74	2.447
**slipAA-*b***	3.45	-411.161301	1.342	-124.41	2.299

Supplementary Table 6 | The total DFTB energies, Lennard-Jones contributions (LJ), and the crystal stacking energies per monolayer as well as the corresponding HOMO-LUMO energy gap for MC-COF-TP-$E_1^2E_7^1$

**Stacking mode**	***c*** **(Å)**	**Total DFTB Energy (a.u.)**	**LJ energy (a.u.)**	**Per unit crystal stacking energy (kcal mol^{-1})**	**HOMO-LUMO gap (eV)**
**monolayer**		-205.238142	0.848		2.528
**AA**	3.52	-410.844036	1.315	-115.38	2.190
**AB**	3.16	-410.634462	1.562	-49.63	2.506
**slipAA-*a***	3.44	-410.857753	1.312	-119.69	2.337
**slipAA-*ab***	3.42	-410.859851	1.312	-120.35	2.377
**slipAA-*b***	3.44	-410.857753	1.312	-119.69	2.337

Supplementary Table 7 | The total DFTB energies, Lennard-Jones contributions (LJ), and the crystal stacking energies per monolayer as well as the corresponding HOMO-LUMO energy gap for MC-COF-TP- $E_3^1E_6^2$

**Stacking mode**	***c*** **(Å)**	**Total DFTB Energy (a.u.)**	**LJ energy (a.u.)**	**Per unit crystal stacking energy (kcal mol^{-1})**	**HOMO-LUMO gap (eV)**
**monolayer**		-177.105511	0.726		2.222
**AA**	3.58	-354.529845	1.122	-100.03	1.590
**AB**	3.34	-354.354672	1.309	-45.07	2.127
**slipAA-*a***	3.42	-354.569600	1.116	-112.51	1.852
**slipAA-*ab***	3.41	-354.572248	1.117	-113.34	1.898
**slipAA-*b***	3.41	-354.574895	1.116	-114.17	1.968

Supplementary Table 8 | The total DFTB energies, Lennard-Jones contributions (LJ), and the crystal stacking energies per monolayer as well as the corresponding HOMO-LUMO energy gap for MC-COF-TP-$E_3^2E_6^1$

**Stacking mode**	***c*** **(Å)**	**Total DFTB Energy (a.u.)**	**LJ energy (a.u.)**	**Per unit crystal stacking energy (kcal mol^{-1})**	**HOMO-LUMO gap (eV)**
**monolayer**		-169.517884	0.689		2.289
**AA**	3.58	-339.276376	1.064	-75.49	1.611
**AB**	3.27	-339.110451	1.255	-23.43	2.244
**slipAA-*a***	3.41	-339.317438	1.059	-88.38	1.900
**slipAA-*ab***	3.42	-339.317006	1.060	-88.24	2.022
**slipAA-*b***	3.41	-339.320167	1.059	-89.23	1.835

Supplementary Table 9 | The total DFTB energies, Lennard-Jones contributions (LJ), and the crystal stacking energies per monolayer as well as the corresponding HOMO-LUMO energy gap for MC-COF-TP-$E_1E_3E_7$

Stacking mode	*c* (Å)	Total DFTB Energy (a.u.)	LJ energy (a.u.)	Per unit crystal stacking energy (kcal mol^{-1})	HOMO-LUMO gap (eV)
**monolayer**		-179.865157	0.757		2.399
**AA**	3.53	-360.207255	1.165	-149.64	1.657
**AB**	3.27	-360.041474	1.344	-97.63	2.309
**slipAA-*a***	3.42	-360.231584	1.160	-157.28	2.031
**slipAA-*ab***	3.41	-360.230862	1.162	-157.05	2.094
**slipAA-*b***	3.43	-360.231703	1.160	-157.31	1.940

Supplementary Table 10 | The total DFTB energies, Lennard-Jones contributions (LJ), and the crystal stacking energies per monolayer as well as the corresponding HOMO-LUMO energy gap for MC-COF-NiPc-E_1E_7

Stacking mode	*c* (Å)	Total DFTB Energy (a.u.)	LJ energy (a.u.)	Per unit crystal stacking energy (kcal mol^{-1})	HOMO-LUMO gap (eV)
**monolayer**		-153.036581	0.582		1.054
**AA**	3.48	-306.349381	0.883	-86.67	0.799
**AB**	3.30	-306.208421	1.030	-42.44	1.066
**slipAA-*a***	3.37	-306.364449	0.881	-91.39	1.005
**slipAA-*ab***	3.39	-306.362199	0.881	-90.69	1.042
**slipAA-*b***	3.41	-306.361919	0.880	-90.60	0.917

Supplementary Table 11 | The total DFTB energies, Lennard-Jones contributions (LJ), and the crystal stacking energies per monolayer as well as the corresponding HOMO-LUMO energy gap for MC-COF-NiPc-E_7E_9

**Stacking mode**	***c* (Å)**	**Total DFTB Energy (a.u.)**	**LJ energy (a.u.)**	**Per unit crystal stacking energy (kcal mol^{-1})**	**HOMO-LUMO gap (eV)**
**monolayer**		-171.569292	0.652		1.057
**AA**	3.50	-343.441579	0.995	-95.07	0.813
**AB**	3.23	-343.247775	1.194	-34.26	1.066
**slipAA-*a***	3.42	-343.454144	0.992	-99.01	0.920
**slipAA-*ab***	3.39	-343.455623	0.993	-99.47	1.042
**slipAA-*b***	3.38	-343.459222	0.992	-100.60	1.008

Supplementary Table 12 | Coordinates for the 0.8-Å slipped AA stacking structure of the MC-COF-TP-$E_1{}^1E_7{}^2$.

		x	*y*	*z*			
C1	1	0.05718	0.79477	0.99615	Biso	1	C
C2	1	0.0595	0.83575	0.99627	Biso	1	C
C3	1	0.10173	0.83866	0.99596	Biso	1	C
C4	1	0.14041	0.80062	0.99256	Biso	1	C
C5	1	0.02058	0.87409	0.99745	Biso	1	C
C6	1	0.10404	0.87991	0.9995	Biso	1	C
C7	1	0.06433	0.91903	0.00033	Biso	1	C
C8	1	0.023	0.91622	0.99829	Biso	1	C
C9	1	0.06697	0.95984	0.00216	Biso	1	C
C10	1	0.14495	0.88303	0.0015	Biso	1	C
C11	1	0.18181	0.84534	0.99874	Biso	1	C
C12	1	0.17963	0.80491	0.99369	Biso	1	C
C13	1	0.01664	0.79335	0.99706	Biso	1	C
C14	1	0.97856	0.83087	0.99801	Biso	1	C
C15	1	0.9795	0.87132	0.99832	Biso	1	C
C16	1	0.98436	0.95429	0.99737	Biso	1	C
C17	1	0.02872	0.99611	0.0016	Biso	1	C
C18	1	0.98821	0.99343	0.99904	Biso	1	C
H1	1	0.14832	0.91384	0.0051	Biso	1	H
H2	1	0.0978	0.96362	0.00374	Biso	1	H
H3	1	0.95201	0.95383	0.99505	Biso	1	H
H4	1	0.14032	0.76836	0.98888	Biso	1	H
H5	1	0.08604	0.76461	0.9958	Biso	1	H
H6	1	0.94893	0.89974	0.99972	Biso	1	H
O1	1	0.22492	0.83979	0.99949	Biso	1	O
O2	1	0.22139	0.77333	0.99061	Biso	1	O
O3	1	0.00575	0.75795	0.99783	Biso	1	O
O4	1	0.94319	0.81957	0.99926	Biso	1	O
O5	1	0.95601	0.03472	0.9985	Biso	1	O
O6	1	0.02262	0.03905	0.00271	Biso	1	O
B1	1	0.24847	0.79549	0.99428	Biso	1	B
B2	1	0.96067	0.77481	0.99928	Biso	1	B
B3	1	0.97791	0.06198	0.00077	Biso	1	B
C19	1	0.95657	0.10989	0.00088	Biso	1	C
C20	1	0.9116	0.13117	0.99958	Biso	1	C
C21	1	0.89194	0.17631	0.99936	Biso	1	C
C22	1	0.91904	0.19993	0.00054	Biso	1	C
C23	1	0.96523	0.17783	0.00196	Biso	1	C
C24	1	0.98303	0.13269	0.00209	Biso	1	C

C25	1	0.84566	0.19946	0.9979	Biso	1	C
C26	1	0.89987	0.24579	0.00031	Biso	1	C
C27	1	0.85369	0.26792	0.99884	Biso	1	C
C28	1	0.82731	0.24337	0.99761	Biso	1	C
C29	1	0.83586	0.31306	0.99873	Biso	1	C
H7	1	0.80101	0.33322	0.99771	Biso	1	H
C30	1	0.86231	0.33587	0.00011	Biso	1	C
C31	1	0.90725	0.31454	0.00152	Biso	1	C
C32	1	0.92695	0.26941	0.0016	Biso	1	C
C33	1	0.97322	0.2463	0.00297	Biso	1	C
C34	1	0.99161	0.20238	0.00313	Biso	1	C
H8	1	0.02706	0.18522	0.00424	Biso	1	H
H9	1	0.99383	0.26463	0.00392	Biso	1	H
H10	1	0.82499	0.18119	0.99696	Biso	1	H
H11	1	0.89333	0.11061	0.99872	Biso	1	H
H12	1	0.01783	0.11234	0.00309	Biso	1	H
H13	1	0.79186	0.26057	0.99641	Biso	1	H
H14	1	0.92544	0.33517	0.00254	Biso	1	H
C35	1	0.52415	0.67337	0.99697	Biso	1	C
C36	1	0.50405	0.71796	0.01193	Biso	1	C
C37	1	0.4587	0.73907	0.01136	Biso	1	C
C38	1	0.43352	0.71369	0.99357	Biso	1	C
C39	1	0.45438	0.66785	0.97731	Biso	1	C
C40	1	0.49983	0.64851	0.97982	Biso	1	C
C41	1	0.43685	0.78499	0.02878	Biso	1	C
C42	1	0.38739	0.73423	0.9926	Biso	1	C
C43	1	0.36653	0.78004	0.00976	Biso	1	C
C44	1	0.39267	0.80464	0.02819	Biso	1	C
C45	1	0.32106	0.79938	0.009	Biso	1	C
H15	1	0.30228	0.83415	0.02211	Biso	1	H
C46	1	0.29676	0.77448	0.99285	Biso	1	C
C47	1	0.31688	0.72994	0.97605	Biso	1	C
C48	1	0.36222	0.70884	0.97509	Biso	1	C
C49	1	0.38405	0.66291	0.95767	Biso	1	C
C50	1	0.42823	0.64324	0.9587	Biso	1	C
H16	1	0.4444	0.60808	0.9446	Biso	1	H
H17	1	0.36455	0.64362	0.94265	Biso	1	H
H18	1	0.45633	0.80429	0.04367	Biso	1	H
H19	1	0.52611	0.7345	0.02498	Biso	1	H
H20	1	0.51875	0.61374	0.96784	Biso	1	H
H21	1	0.37649	0.83978	0.04263	Biso	1	H
H22	1	0.29468	0.71354	0.96336	Biso	1	H
C51	1	0.76419	0.65092	0.0043	Biso	1	C

C52	1	0.7616	0.61006	0.00358	Biso	1	C
C53	1	0.71923	0.60753	0.0025	Biso	1	C
C54	1	0.68079	0.64582	0.00485	Biso	1	C
C55	1	0.80032	0.57153	0.00299	Biso	1	C
C56	1	0.71656	0.56646	0.99858	Biso	1	C
C57	1	0.75605	0.52712	0.99898	Biso	1	C
C58	1	0.79755	0.52953	0.00196	Biso	1	C
C59	1	0.75304	0.48645	0.99733	Biso	1	C
C60	1	0.67548	0.56373	0.99496	Biso	1	C
C61	1	0.63885	0.60162	0.99668	Biso	1	C
C62	1	0.64143	0.64187	0.00222	Biso	1	C
C63	1	0.80485	0.652	0.00431	Biso	1	C
C64	1	0.84277	0.61431	0.00373	Biso	1	C
C65	1	0.84154	0.57399	0.00306	Biso	1	C
C66	1	0.83597	0.49118	0.0036	Biso	1	C
C67	1	0.79105	0.44992	0.9987	Biso	1	C
C68	1	0.83176	0.45219	0.00179	Biso	1	C
H23	1	0.67181	0.53307	0.99075	Biso	1	H
H24	1	0.72204	0.48301	0.9951	Biso	1	H
H25	1	0.86843	0.49136	0.00653	Biso	1	H
H26	1	0.68114	0.67797	0.00895	Biso	1	H
H27	1	0.73549	0.68121	0.00444	Biso	1	H
H28	1	0.87198	0.54541	0.00222	Biso	1	H
O7	1	0.59563	0.60755	0.99404	Biso	1	O
O8	1	0.59979	0.67374	0.00375	Biso	1	O
O9	1	0.81603	0.68725	0.0044	Biso	1	O
O10	1	0.87833	0.62535	0.00355	Biso	1	O
O11	1	0.86364	0.41075	0.00272	Biso	1	O
O12	1	0.79672	0.40713	0.99772	Biso	1	O
B4	1	0.57241	0.65189	0.99851	Biso	1	B
B5	1	0.86113	0.6701	0.00388	Biso	1	B
B6	1	0.84137	0.38381	0.00016	Biso	1	B
C69	1	0.88734	0.69676	0.00335	Biso	1	C
C70	1	0.86579	0.74154	0.00093	Biso	1	C
C71	1	0.88921	0.76732	0.99968	Biso	1	C
C72	1	0.9345	0.7481	0.00087	Biso	1	C
C73	1	0.95606	0.70333	0.00367	Biso	1	C
C74	1	0.93265	0.67755	0.00493	Biso	1	C
H29	1	0.83032	0.75382	0.99993	Biso	1	H
H30	1	0.87424	0.80256	0.99754	Biso	1	H
H31	1	0.99154	0.69104	0.00487	Biso	1	H
H32	1	0.94765	0.64231	0.00725	Biso	1	H
C75	1	0.04057	0.83178	0.4968	Biso	1	C

C76	1	0.04328	0.87258	0.49712	Biso	1	C
C77	1	0.0857	0.87501	0.49745	Biso	1	C
C78	1	0.1241	0.83668	0.4945	Biso	1	C
C79	1	0.00463	0.91118	0.49795	Biso	1	C
C80	1	0.08847	0.91604	0.50116	Biso	1	C
C81	1	0.04903	0.95544	0.50139	Biso	1	C
C82	1	0.00749	0.95313	0.49884	Biso	1	C
C83	1	0.05215	0.99607	0.50315	Biso	1	C
C84	1	0.12957	0.9187	0.50389	Biso	1	C
C85	1	0.16616	0.88078	0.50165	Biso	1	C
C86	1	0.16351	0.84055	0.49636	Biso	1	C
C87	1	0.99986	0.83081	0.49728	Biso	1	C
C88	1	0.962	0.86857	0.49796	Biso	1	C
C89	1	0.96335	0.90884	0.49836	Biso	1	C
C90	1	0.96913	0.99153	0.49754	Biso	1	C
C91	1	0.01418	0.03264	0.50212	Biso	1	C
C92	1	0.97343	0.03047	0.49929	Biso	1	C
H33	1	0.13331	0.94933	0.50778	Biso	1	H
H34	1	0.08318	0.99943	0.50519	Biso	1	H
H35	1	0.93663	0.99143	0.4949	Biso	1	H
H36	1	0.12368	0.80456	0.49051	Biso	1	H
H37	1	0.06922	0.80144	0.49661	Biso	1	H
H38	1	0.93296	0.93747	0.49934	Biso	1	H
O13	1	0.20939	0.87479	0.50357	Biso	1	O
O14	1	0.20514	0.80865	0.49428	Biso	1	O
O15	1	0.98854	0.79565	0.49758	Biso	1	O
O16	1	0.92636	0.85767	0.49857	Biso	1	O
O17	1	0.94162	0.07194	0.49852	Biso	1	O
O18	1	0.00861	0.07541	0.50312	Biso	1	O
B7	1	0.23256	0.83045	0.49902	Biso	1	B
B8	1	0.94344	0.81292	0.49837	Biso	1	B
B9	1	0.96398	0.09881	0.50087	Biso	1	B
C93	1	0.9431	0.14675	0.50082	Biso	1	C
C94	1	0.89817	0.16808	0.49934	Biso	1	C
C95	1	0.87846	0.21321	0.49916	Biso	1	C
C96	1	0.90555	0.23683	0.50044	Biso	1	C
C97	1	0.95173	0.21469	0.50191	Biso	1	C
C98	1	0.96957	0.16956	0.50211	Biso	1	C
C99	1	0.8322	0.23633	0.4977	Biso	1	C
C100	1	0.88638	0.28269	0.50024	Biso	1	C
C101	1	0.84019	0.30479	0.49885	Biso	1	C
C102	1	0.81381	0.28024	0.49756	Biso	1	C
C103	1	0.82239	0.34993	0.49883	Biso	1	C

H39	1	0.78761	0.37031	0.49787	Biso	1	H
C104	1	0.84885	0.37273	0.50013	Biso	1	C
C105	1	0.89383	0.35145	0.50136	Biso	1	C
C106	1	0.91348	0.30631	0.50145	Biso	1	C
C107	1	0.95976	0.28316	0.5028	Biso	1	C
C108	1	0.97811	0.23925	0.50306	Biso	1	C
H40	1	0.01356	0.22204	0.50417	Biso	1	H
H41	1	0.98043	0.30142	0.50369	Biso	1	H
H42	1	0.81159	0.21799	0.49667	Biso	1	H
H43	1	0.88	0.14743	0.49836	Biso	1	H
H44	1	0.00442	0.14942	0.50317	Biso	1	H
H45	1	0.77836	0.2974	0.4964	Biso	1	H
H46	1	0.91212	0.37198	0.50226	Biso	1	H
C109	1	0.50822	0.70786	0.50434	Biso	1	C
C110	1	0.4881	0.7524	0.52115	Biso	1	C
C111	1	0.44276	0.77351	0.5221	Biso	1	C
C112	1	0.41758	0.74812	0.50457	Biso	1	C
C113	1	0.43845	0.70231	0.48741	Biso	1	C
C114	1	0.48392	0.68296	0.48817	Biso	1	C
C115	1	0.42092	0.81944	0.53958	Biso	1	C
C116	1	0.37145	0.76866	0.5036	Biso	1	C
C117	1	0.35059	0.8145	0.51987	Biso	1	C
C118	1	0.37674	0.83911	0.53854	Biso	1	C
C119	1	0.30514	0.83383	0.51737	Biso	1	C
H47	1	0.28622	0.8686	0.5294	Biso	1	H
C120	1	0.28082	0.80897	0.50025	Biso	1	C
C121	1	0.30093	0.76438	0.48528	Biso	1	C
C122	1	0.34628	0.74328	0.48583	Biso	1	C
C123	1	0.36813	0.69736	0.46845	Biso	1	C
C124	1	0.4123	0.67771	0.46904	Biso	1	C
H48	1	0.42849	0.64256	0.45465	Biso	1	H
H49	1	0.34864	0.67805	0.45361	Biso	1	H
H50	1	0.44042	0.83873	0.55466	Biso	1	H
H51	1	0.5103	0.76879	0.53389	Biso	1	H
H52	1	0.50269	0.64819	0.4751	Biso	1	H
H53	1	0.36058	0.87426	0.5527	Biso	1	H
H54	1	0.27888	0.74782	0.47228	Biso	1	H
C125	1	0.74757	0.68793	0.505	Biso	1	C
C126	1	0.74539	0.64689	0.50441	Biso	1	C
C127	1	0.70321	0.64388	0.50386	Biso	1	C
C128	1	0.66449	0.68188	0.50663	Biso	1	C
C129	1	0.78437	0.60862	0.50348	Biso	1	C
C130	1	0.70098	0.60259	0.50009	Biso	1	C

C131	1	0.74076	0.56353	0.49988	Biso	1	C
C132	1	0.78204	0.56644	0.50241	Biso	1	C
C133	1	0.73822	0.52268	0.49815	Biso	1	C
C134	1	0.66011	0.5994	0.4972	Biso	1	C
C135	1	0.6232	0.63705	0.49948	Biso	1	C
C136	1	0.62532	0.67751	0.50476	Biso	1	C
C137	1	0.78806	0.68947	0.50463	Biso	1	C
C138	1	0.82621	0.65201	0.50379	Biso	1	C
C139	1	0.82539	0.61151	0.50316	Biso	1	C
C140	1	0.82073	0.52843	0.50368	Biso	1	C
C141	1	0.77652	0.48646	0.49909	Biso	1	C
C142	1	0.81698	0.48924	0.50195	Biso	1	C
H55	1	0.6568	0.56856	0.49331	Biso	1	H
H56	1	0.70743	0.51883	0.49637	Biso	1	H
H57	1	0.85305	0.52896	0.5063	Biso	1	H
H58	1	0.66451	0.71417	0.5104	Biso	1	H
H59	1	0.71866	0.71804	0.50529	Biso	1	H
H60	1	0.85601	0.58315	0.5019	Biso	1	H
O19	1	0.5801	0.64256	0.49804	Biso	1	O
O20	1	0.58354	0.70905	0.5073	Biso	1	O
O21	1	0.79881	0.72495	0.50426	Biso	1	O
O22	1	0.86149	0.66345	0.50298	Biso	1	O
O23	1	0.84925	0.44798	0.50271	Biso	1	O
O24	1	0.78272	0.44349	0.49805	Biso	1	O
B10	1	0.5565	0.68685	0.50317	Biso	1	B
B11	1	0.84389	0.70821	0.50308	Biso	1	B
B12	1	0.82743	0.42064	0.50027	Biso	1	B
C143	1	0.86994	0.73504	0.50169	Biso	1	C
C144	1	0.84837	0.77982	0.49889	Biso	1	C
C145	1	0.87179	0.80559	0.49761	Biso	1	C
C146	1	0.91711	0.78637	0.49916	Biso	1	C
C147	1	0.93865	0.7416	0.50162	Biso	1	C
C148	1	0.91523	0.71582	0.5029	Biso	1	C
H61	1	0.81289	0.79217	0.49766	Biso	1	H
H62	1	0.85677	0.84084	0.49524	Biso	1	H
H63	1	0.97412	0.72936	0.50257	Biso	1	H
H64	1	0.93015	0.68059	0.50503	Biso	1	H

Supplementary Table 13 | Coordinates for the 0.8-Å slipped AA stacking structure of the MC-COF-TP-$E_1{}^2E_7{}^1$

		x	*y*	*z*			
C1	1	0.09256	0.77818	0.00895	Biso	1	C
C2	1	0.08972	0.82172	0.0055	Biso	1	C
C3	1	0.1359	0.81761	0.00621	Biso	1	C
C4	1	0.18358	0.77013	0.00799	Biso	1	C
C5	1	0.04176	0.86951	0.00014	Biso	1	C
C6	1	0.13304	0.86143	0.00363	Biso	1	C
C7	1	0.08405	0.91021	0.00187	Biso	1	C
C8	1	0.03886	0.91427	0.9994	Biso	1	C
C9	1	0.08153	0.95355	0.00268	Biso	1	C
C10	1	0.17775	0.85773	0.00237	Biso	1	C
C11	1	0.22339	0.81095	0.00387	Biso	1	C
C12	1	0.22628	0.76799	0.00672	Biso	1	C
C13	1	0.04804	0.78365	0.00571	Biso	1	C
C14	1	0.00108	0.83046	0.99892	Biso	1	C
C15	1	0.99681	0.87361	0.99626	Biso	1	C
C16	1	0.99117	0.96172	0.99731	Biso	1	C
C17	1	0.03451	0.99902	0.00067	Biso	1	C
C18	1	0.99021	0.00306	0.99789	Biso	1	C
H1	1	0.1774	0.89018	0.99948	Biso	1	H
H2	1	0.11504	0.95228	0.00536	Biso	1	H
H3	1	0.95558	0.9667	0.99566	Biso	1	H
H4	1	0.1877	0.73557	0.0098	Biso	1	H
H5	1	0.12835	0.74089	0.01423	Biso	1	H
H6	1	0.95934	0.90932	0.99098	Biso	1	H
O1	1	0.27171	0.79753	0.00223	Biso	1	O
O2	1	0.27653	0.72693	0.00691	Biso	1	O
O3	1	0.04065	0.74762	0.00776	Biso	1	O
O4	1	0.96353	0.82452	0.99621	Biso	1	O
O5	1	0.94921	0.05285	0.99673	Biso	1	O
O6	1	0.02202	0.04616	0.00138	Biso	1	O
B1	1	0.30354	0.74596	0.00394	Biso	1	B
B2	1	0.98867	0.77355	0.00177	Biso	1	B
B3	1	0.96964	0.07843	0.99897	Biso	1	B
C19	1	0.93955	0.13352	0.99883	Biso	1	C
C20	1	0.96448	0.15491	0.0033	Biso	1	C
C21	1	0.937	0.20615	0.00348	Biso	1	C
C22	1	0.88443	0.23598	0.99908	Biso	1	C
C23	1	0.85951	0.21458	0.99437	Biso	1	C
C24	1	0.88696	0.16337	0.99422	Biso	1	C

H7	1	0.00569	0.12883	0.0068	Biso	1	H
H8	1	0.95397	0.22546	0.00719	Biso	1	H
H9	1	0.8183	0.24073	0.99079	Biso	1	H
H10	1	0.87007	0.14398	0.99048	Biso	1	H
C25	1	0.35961	0.71499	0.00239	Biso	1	C
C26	1	0.38761	0.66343	0.00318	Biso	1	C
C27	1	0.43984	0.63386	0.00151	Biso	1	C
C28	1	0.46404	0.65601	0.99904	Biso	1	C
C29	1	0.43605	0.7076	0.99829	Biso	1	C
C30	1	0.38381	0.73717	0.99995	Biso	1	C
H11	1	0.36552	0.64932	0.00512	Biso	1	H
H12	1	0.46433	0.59324	0.00203	Biso	1	H
H13	1	0.45794	0.72192	0.99631	Biso	1	H
H14	1	0.35948	0.77778	0.99944	Biso	1	H
C31	1	0.73119	0.59133	0.99262	Biso	1	C
C32	1	0.73413	0.54778	0.99574	Biso	1	C
C33	1	0.68792	0.55201	0.99532	Biso	1	C
C34	1	0.6404	0.59961	0.99447	Biso	1	C
C35	1	0.78208	0.49999	0.00018	Biso	1	C
C36	1	0.69063	0.50828	0.99696	Biso	1	C
C37	1	0.73963	0.45941	0.99795	Biso	1	C
C38	1	0.78488	0.45529	0.00031	Biso	1	C
C39	1	0.7422	0.41603	0.99669	Biso	1	C
C40	1	0.64573	0.5123	0.99796	Biso	1	C
C41	1	0.60017	0.55921	0.99711	Biso	1	C
C42	1	0.59757	0.60201	0.99537	Biso	1	C
C43	1	0.77571	0.58582	0.9955	Biso	1	C
C44	1	0.82268	0.53907	0.0018	Biso	1	C
C45	1	0.82703	0.49586	0.00384	Biso	1	C
C46	1	0.83255	0.40781	0.00186	Biso	1	C
C47	1	0.78923	0.37049	0.9983	Biso	1	C
C48	1	0.83353	0.36646	0.00096	Biso	1	C
H15	1	0.64591	0.47993	0.9998	Biso	1	H
H16	1	0.70864	0.41736	0.99416	Biso	1	H
H17	1	0.86816	0.40279	0.0034	Biso	1	H
H18	1	0.63644	0.63408	0.99365	Biso	1	H
H19	1	0.69543	0.62863	0.98794	Biso	1	H
H20	1	0.86451	0.46015	0.0086	Biso	1	H
O7	1	0.5517	0.57298	0.99837	Biso	1	O
O8	1	0.54745	0.64326	0.99555	Biso	1	O
O9	1	0.78293	0.62197	0.9939	Biso	1	O
O10	1	0.86016	0.54516	0.00471	Biso	1	O
O11	1	0.87452	0.31671	0.00176	Biso	1	O

O12	1	0.80175	0.32336	0.99729	Biso	1	O
B4	1	0.52014	0.62455	0.99751	Biso	1	B
B5	1	0.83487	0.59616	0.99985	Biso	1	B
B6	1	0.85412	0.29109	0.99944	Biso	1	B
C49	1	0.85952	0.62095	0.00114	Biso	1	C
C50	1	0.91158	0.59425	0.01427	Biso	1	C
C51	1	0.93321	0.61912	0.0154	Biso	1	C
C52	1	0.90092	0.6721	0.00187	Biso	1	C
C53	1	0.84751	0.69917	0.98815	Biso	1	C
C54	1	0.82802	0.67236	0.98882	Biso	1	C
C55	1	0.98669	0.59302	0.03001	Biso	1	C
C56	1	0.92211	0.69807	0.00189	Biso	1	C
C57	1	0.97551	0.671	0.01634	Biso	1	C
C58	1	0.00699	0.61789	0.03068	Biso	1	C
C59	1	0.99499	0.6978	0.01562	Biso	1	C
H21	1	0.03498	0.68175	0.02603	Biso	1	H
C60	1	0.96362	0.74915	0.00139	Biso	1	C
C61	1	0.91157	0.77586	0.98731	Biso	1	C
C62	1	0.88989	0.75102	0.98729	Biso	1	C
C63	1	0.83642	0.7771	0.97289	Biso	1	C
C64	1	0.81606	0.75228	0.97371	Biso	1	C
H22	1	0.77509	0.77282	0.9623	Biso	1	H
H23	1	0.81185	0.81772	0.96088	Biso	1	H
H24	1	0.0113	0.55238	0.04145	Biso	1	H
H25	1	0.93381	0.55381	0.02433	Biso	1	H
H26	1	0.78799	0.68852	0.97903	Biso	1	H
H27	1	0.04795	0.5974	0.04261	Biso	1	H
H28	1	0.88926	0.81629	0.97597	Biso	1	H
C65	1	0.09926	0.81125	0.50736	Biso	1	C
C66	1	0.09625	0.85484	0.50424	Biso	1	C
C67	1	0.14243	0.85066	0.50467	Biso	1	C
C68	1	0.18999	0.8031	0.50556	Biso	1	C
C69	1	0.04827	0.9026	0.49977	Biso	1	C
C70	1	0.13965	0.89443	0.503	Biso	1	C
C71	1	0.09062	0.94327	0.50205	Biso	1	C
C72	1	0.04539	0.94734	0.49966	Biso	1	C
C73	1	0.08801	0.98667	0.50337	Biso	1	C
C74	1	0.1845	0.8905	0.50195	Biso	1	C
C75	1	0.23009	0.84362	0.50283	Biso	1	C
C76	1	0.23277	0.80078	0.50464	Biso	1	C
C77	1	0.05478	0.81669	0.50447	Biso	1	C
C78	1	0.00777	0.86341	0.49814	Biso	1	C
C79	1	0.00335	0.90666	0.49608	Biso	1	C

C80	1	0.9977	0.99481	0.49811	Biso	1	C
C81	1	0.04097	0.03218	0.50177	Biso	1	C
C82	1	0.99669	0.03618	0.49904	Biso	1	C
H29	1	0.18426	0.9229	0.50003	Biso	1	H
H30	1	0.12155	0.98537	0.50597	Biso	1	H
H31	1	0.96212	0.9998	0.49653	Biso	1	H
H32	1	0.19401	0.76859	0.50643	Biso	1	H
H33	1	0.13504	0.77397	0.51207	Biso	1	H
H34	1	0.96585	0.94235	0.4913	Biso	1	H
O13	1	0.27852	0.82995	0.50154	Biso	1	O
O14	1	0.28292	0.75957	0.5045	Biso	1	O
O15	1	0.04763	0.78049	0.50608	Biso	1	O
O16	1	0.97034	0.85724	0.49522	Biso	1	O
O17	1	0.95568	0.08593	0.49826	Biso	1	O
O18	1	0.02843	0.07932	0.50282	Biso	1	O
B7	1	0.31015	0.77838	0.50248	Biso	1	B
B8	1	0.9957	0.80623	0.50011	Biso	1	B
B9	1	0.97606	0.11157	0.50064	Biso	1	B
C83	1	0.94577	0.16668	0.501	Biso	1	C
C84	1	0.9707	0.18808	0.5057	Biso	1	C
C85	1	0.94324	0.23929	0.50585	Biso	1	C
C86	1	0.89066	0.26914	0.50123	Biso	1	C
C87	1	0.86572	0.24775	0.49677	Biso	1	C
C88	1	0.8932	0.19651	0.4966	Biso	1	C
H35	1	0.01191	0.16194	0.50928	Biso	1	H
H36	1	0.96015	0.25867	0.50957	Biso	1	H
H37	1	0.82451	0.27384	0.49326	Biso	1	H
H38	1	0.87624	0.17719	0.49289	Biso	1	H
C89	1	0.36625	0.747	0.50097	Biso	1	C
C90	1	0.39425	0.69541	0.50173	Biso	1	C
C91	1	0.44648	0.66584	0.50007	Biso	1	C
C92	1	0.47068	0.68802	0.49764	Biso	1	C
C93	1	0.44268	0.73958	0.49685	Biso	1	C
C94	1	0.39045	0.76915	0.49851	Biso	1	C
H39	1	0.37233	0.68112	0.5037	Biso	1	H
H40	1	0.47084	0.62523	0.50058	Biso	1	H
H41	1	0.46473	0.75374	0.49492	Biso	1	H
H42	1	0.36598	0.80977	0.49799	Biso	1	H
C95	1	0.7379	0.62439	0.49104	Biso	1	C
C96	1	0.74066	0.58089	0.49449	Biso	1	C
C97	1	0.69445	0.58506	0.4938	Biso	1	C
C98	1	0.64681	0.63258	0.49208	Biso	1	C
C99	1	0.78859	0.53307	0.4998	Biso	1	C

C100	1	0.69724	0.54128	0.49634	Biso	1	C
C101	1	0.7462	0.49247	0.49811	Biso	1	C
C102	1	0.79142	0.48836	0.50054	Biso	1	C
C103	1	0.74868	0.44915	0.49735	Biso	1	C
C104	1	0.65248	0.54506	0.49756	Biso	1	C
C105	1	0.60688	0.59188	0.4961	Biso	1	C
C106	1	0.60407	0.63479	0.49333	Biso	1	C
C107	1	0.78245	0.61886	0.49426	Biso	1	C
C108	1	0.82937	0.57202	0.50102	Biso	1	C
C109	1	0.83358	0.5289	0.50366	Biso	1	C
C110	1	0.83909	0.44089	0.50261	Biso	1	C
C111	1	0.79569	0.40366	0.49936	Biso	1	C
C112	1	0.84001	0.39959	0.50207	Biso	1	C
H43	1	0.65277	0.51265	0.50038	Biso	1	H
H44	1	0.71515	0.45045	0.49474	Biso	1	H
H45	1	0.87469	0.43588	0.50421	Biso	1	H
H46	1	0.64275	0.6671	0.49032	Biso	1	H
H47	1	0.70213	0.66171	0.48578	Biso	1	H
H48	1	0.87103	0.49318	0.50892	Biso	1	H
O19	1	0.55851	0.60539	0.49773	Biso	1	O
O20	1	0.55385	0.67591	0.49318	Biso	1	O
O21	1	0.78992	0.65483	0.49222	Biso	1	O
O22	1	0.86698	0.57788	0.50373	Biso	1	O
O23	1	0.88099	0.34979	0.50324	Biso	1	O
O24	1	0.80817	0.35652	0.49869	Biso	1	O
B10	1	0.52675	0.65697	0.49609	Biso	1	B
B11	1	0.8419	0.62884	0.4982	Biso	1	B
B12	1	0.86054	0.32423	0.50107	Biso	1	B
C113	1	0.86704	0.65316	0.4986	Biso	1	C
C114	1	0.91909	0.62644	0.51268	Biso	1	C
C115	1	0.94076	0.65129	0.5127	Biso	1	C
C116	1	0.90854	0.70423	0.4981	Biso	1	C
C117	1	0.85514	0.73131	0.48365	Biso	1	C
C118	1	0.83567	0.7045	0.48438	Biso	1	C
C119	1	0.99423	0.62521	0.52711	Biso	1	C
C120	1	0.92973	0.73021	0.49812	Biso	1	C
C121	1	0.98314	0.70314	0.51185	Biso	1	C
C122	1	0.01459	0.65004	0.52629	Biso	1	C
C123	1	0.00263	0.72995	0.51118	Biso	1	C
H49	1	0.04267	0.71374	0.52099	Biso	1	H
C124	1	0.97113	0.78137	0.49885	Biso	1	C
C125	1	0.91907	0.80806	0.48571	Biso	1	C
C126	1	0.89743	0.78319	0.48459	Biso	1	C

C127	1	0.84395	0.80929	0.46997	Biso	1	C
C128	1	0.82365	0.78442	0.46931	Biso	1	C
H50	1	0.7827	0.80491	0.45738	Biso	1	H
H51	1	0.81935	0.84993	0.45853	Biso	1	H
H52	1	0.0188	0.58459	0.53912	Biso	1	H
H53	1	0.94141	0.58601	0.52402	Biso	1	H
H54	1	0.79569	0.72049	0.47399	Biso	1	H
H55	1	0.05556	0.62949	0.53771	Biso	1	H
H56	1	0.89684	0.84849	0.47565	Biso	1	H

Supplementary Table 14 | Coordinates for the 0.8-Å slipped AA stacking structure of the MC-COF-TP-$E_3{}^1E_6{}^2$

		x	*y*	*z*			
C1	1	0.04602	0.80824	0.99724	Biso	1	C
C2	1	0.04706	0.84814	0.99655	Biso	1	C
C3	1	0.08773	0.85101	0.99733	Biso	1	C
C4	1	0.12592	0.81375	0.99337	Biso	1	C
C5	1	0.00842	0.88546	0.99621	Biso	1	C
C6	1	0.08914	0.89103	0.00244	Biso	1	C
C7	1	0.04961	0.92921	0.00223	Biso	1	C
C8	1	0.00979	0.92635	0.99723	Biso	1	C
C9	1	0.05078	0.96906	0.00543	Biso	1	C
C10	1	0.12882	0.89361	0.00642	Biso	1	C
C11	1	0.16539	0.85662	0.00382	Biso	1	C
C12	1	0.16388	0.8175	0.99649	Biso	1	C
C13	1	0.0069	0.80685	0.9969	Biso	1	C
C14	1	0.969	0.84343	0.99597	Biso	1	C
C15	1	0.96877	0.88281	0.99585	Biso	1	C
C16	1	0.97129	0.96327	0.99399	Biso	1	C
C17	1	0.01263	0.00435	0.00291	Biso	1	C
C18	1	0.9737	0.00144	0.99687	Biso	1	C
H1	1	0.13143	0.9235	0.01125	Biso	1	H
H2	1	0.08046	0.97274	0.00968	Biso	1	H
H3	1	0.94018	0.96259	0.98878	Biso	1	H
H4	1	0.12636	0.78254	0.98738	Biso	1	H
H5	1	0.07473	0.77886	0.9988	Biso	1	H
H6	1	0.9384	0.91047	0.99623	Biso	1	H
O1	1	0.2075	0.85044	0.00602	Biso	1	O
O2	1	0.20482	0.78623	0.99326	Biso	1	O
O3	1	0.99751	0.77229	0.99833	Biso	1	O
O4	1	0.93517	0.83244	0.99654	Biso	1	O
O5	1	0.94116	0.04139	0.99485	Biso	1	O
O6	1	0.00506	0.04638	0.00478	Biso	1	O
B1	1	0.2309	0.80719	0.99923	Biso	1	B
B2	1	0.95344	0.78884	0.99816	Biso	1	B
B3	1	0.96113	0.06826	0.99983	Biso	1	B
C19	1	0.85127	0.26364	0.99793	Biso	1	C
C20	1	0.89344	0.26137	0.00261	Biso	1	C
C21	1	0.92931	0.22303	0.00457	Biso	1	C
C22	1	0.92295	0.18621	0.00187	Biso	1	C
C23	1	0.8808	0.18843	0.99718	Biso	1	C
C24	1	0.84494	0.22674	0.99525	Biso	1	C

C25	1	0.82004	0.30615	0.99674	Biso	1	C
C26	1	0.83706	0.3351	0.00035	Biso	1	C
H7	1	0.96138	0.22171	0.00834	Biso	1	H
H8	1	0.81285	0.2281	0.99163	Biso	1	H
H9	1	0.78571	0.31584	0.99332	Biso	1	H
C27	1	0.95427	0.14371	0.00318	Biso	1	C
C28	1	0.93733	0.11474	0.99972	Biso	1	C
H10	1	0.98859	0.13399	0.00669	Biso	1	H
S1	1	0.88212	0.1384	0.99484	Biso	1	S
S2	1	0.89226	0.31114	0.00517	Biso	1	S
C29	1	0.34632	0.73021	0.98744	Biso	1	C
C30	1	0.35621	0.76578	0.00491	Biso	1	C
C31	1	0.39807	0.76135	0.0128	Biso	1	C
C32	1	0.43066	0.72062	0.00186	Biso	1	C
C33	1	0.42081	0.68505	0.98368	Biso	1	C
C34	1	0.37897	0.68945	0.97671	Biso	1	C
C35	1	0.30152	0.7427	0.98426	Biso	1	C
C36	1	0.27809	0.78543	0.99804	Biso	1	C
H11	1	0.40545	0.78858	0.02852	Biso	1	H
H12	1	0.3716	0.66217	0.96315	Biso	1	H
H13	1	0.28599	0.72148	0.97148	Biso	1	H
C37	1	0.47548	0.70817	0.00707	Biso	1	C
C38	1	0.49909	0.66549	0.99368	Biso	1	C
H14	1	0.49086	0.7295	0.02097	Biso	1	H
S3	1	0.46703	0.63873	0.97353	Biso	1	S
S4	1	0.31018	0.81199	0.01542	Biso	1	S
C39	1	0.73248	0.64153	0.00178	Biso	1	C
C40	1	0.73065	0.60194	0.00253	Biso	1	C
C41	1	0.68967	0.59967	0.0012	Biso	1	C
C42	1	0.6516	0.63713	0.00455	Biso	1	C
C43	1	0.76892	0.5642	0.00353	Biso	1	C
C44	1	0.68789	0.55981	0.99611	Biso	1	C
C45	1	0.72702	0.52126	0.99686	Biso	1	C
C46	1	0.76709	0.5235	0.0026	Biso	1	C
C47	1	0.72523	0.48165	0.99351	Biso	1	C
C48	1	0.64809	0.55754	0.99153	Biso	1	C
C49	1	0.61174	0.59464	0.99363	Biso	1	C
C50	1	0.61346	0.63368	0.00098	Biso	1	C
C51	1	0.77197	0.64217	0.00279	Biso	1	C
C52	1	0.80943	0.60514	0.00437	Biso	1	C
C53	1	0.8089	0.56611	0.00442	Biso	1	C
C54	1	0.80526	0.48616	0.00642	Biso	1	C
C55	1	0.76302	0.44598	0.99666	Biso	1	C

C56	1	0.80229	0.44823	0.00328	Biso	1	C
H15	1	0.64519	0.5278	0.98632	Biso	1	H
H16	1	0.69531	0.47844	0.98849	Biso	1	H
H17	1	0.83658	0.48638	0.01217	Biso	1	H
H18	1	0.65148	0.66823	0.0103	Biso	1	H
H19	1	0.70406	0.67119	0.99971	Biso	1	H
H20	1	0.83898	0.53812	0.00464	Biso	1	H
O7	1	0.56969	0.60081	0.98995	Biso	1	O
O8	1	0.5724	0.66502	0.00284	Biso	1	O
O9	1	0.78238	0.67618	0.00208	Biso	1	O
O10	1	0.84386	0.61519	0.00499	Biso	1	O
O11	1	0.83433	0.40802	0.00565	Biso	1	O
O12	1	0.7699	0.40415	0.99473	Biso	1	O
B4	1	0.54629	0.64403	0.9957	Biso	1	B
B5	1	0.82653	0.65875	0.00356	Biso	1	B
B6	1	0.81377	0.38164	0.00026	Biso	1	B
C57	1	0.92827	0.76355	0.9998	Biso	1	C
C58	1	0.94402	0.72014	0.00464	Biso	1	C
C59	1	0.91109	0.70653	0.0047	Biso	1	C
C60	1	0.86995	0.74027	0.99963	Biso	1	C
C61	1	0.85246	0.68331	0.00358	Biso	1	C
C62	1	0.83696	0.72665	0.99894	Biso	1	C
H21	1	0.97805	0.70002	0.00813	Biso	1	H
H22	1	0.80294	0.74683	0.99471	Biso	1	H
S5	1	0.90766	0.65911	0.00902	Biso	1	S
S6	1	0.87327	0.78758	0.99549	Biso	1	S
C63	1	0.02857	0.84649	0.49749	Biso	1	C
C64	1	0.03003	0.88625	0.4973	Biso	1	C
C65	1	0.07084	0.88889	0.49913	Biso	1	C
C66	1	0.10912	0.85164	0.49628	Biso	1	C
C67	1	0.99156	0.92379	0.49644	Biso	1	C
C68	1	0.07223	0.92893	0.50418	Biso	1	C
C69	1	0.0329	0.96727	0.50317	Biso	1	C
C70	1	0.993	0.96467	0.49755	Biso	1	C
C71	1	0.03433	0.00704	0.50628	Biso	1	C
C72	1	0.11185	0.93158	0.50905	Biso	1	C
C73	1	0.14841	0.89467	0.50699	Biso	1	C
C74	1	0.14707	0.85547	0.4998	Biso	1	C
C75	1	0.98924	0.8455	0.49658	Biso	1	C
C76	1	0.95158	0.88233	0.49536	Biso	1	C
C77	1	0.95176	0.92151	0.49544	Biso	1	C
C78	1	0.95464	0.00181	0.49386	Biso	1	C
C79	1	0.99636	0.04251	0.50326	Biso	1	C

C80	1	0.95725	0.03991	0.49693	Biso	1	C
H23	1	0.11445	0.96146	0.51436	Biso	1	H
H24	1	0.06411	0.01052	0.51097	Biso	1	H
H25	1	0.92344	0.00132	0.48829	Biso	1	H
H26	1	0.10954	0.82042	0.49096	Biso	1	H
H27	1	0.05715	0.81697	0.49903	Biso	1	H
H28	1	0.92154	0.94934	0.49534	Biso	1	H
O13	1	0.19034	0.8889	0.51045	Biso	1	O
O14	1	0.18828	0.8244	0.49781	Biso	1	O
O15	1	0.97917	0.81126	0.49736	Biso	1	O
O16	1	0.91738	0.87194	0.49505	Biso	1	O
O17	1	0.92498	0.08	0.49469	Biso	1	O
O18	1	0.98914	0.08443	0.50516	Biso	1	O
B7	1	0.21407	0.84573	0.50456	Biso	1	B
B8	1	0.93503	0.82837	0.49645	Biso	1	B
B9	1	0.94523	0.10665	0.4999	Biso	1	B
C81	1	0.83578	0.30207	0.49838	Biso	1	C
C82	1	0.87793	0.29985	0.50304	Biso	1	C
C83	1	0.9138	0.26155	0.50497	Biso	1	C
C84	1	0.90748	0.22464	0.50232	Biso	1	C
C85	1	0.86532	0.22689	0.49764	Biso	1	C
C86	1	0.82943	0.26523	0.49568	Biso	1	C
C87	1	0.80447	0.34457	0.49704	Biso	1	C
C88	1	0.82143	0.37353	0.50043	Biso	1	C
H29	1	0.94588	0.26019	0.50856	Biso	1	H
H30	1	0.79736	0.26654	0.49191	Biso	1	H
H31	1	0.77015	0.3543	0.49359	Biso	1	H
C89	1	0.93873	0.18215	0.50352	Biso	1	C
C90	1	0.92174	0.15317	0.4999	Biso	1	C
H32	1	0.97305	0.17248	0.50698	Biso	1	H
S7	1	0.86654	0.1771	0.49508	Biso	1	S
S8	1	0.87663	0.34986	0.50533	Biso	1	S
C91	1	0.32976	0.7695	0.49672	Biso	1	C
C92	1	0.33959	0.80509	0.5148	Biso	1	C
C93	1	0.38144	0.80068	0.52173	Biso	1	C
C94	1	0.41409	0.75992	0.51109	Biso	1	C
C95	1	0.40419	0.72436	0.49343	Biso	1	C
C96	1	0.36234	0.72878	0.48551	Biso	1	C
C97	1	0.28496	0.7819	0.49218	Biso	1	C
C98	1	0.26129	0.82456	0.50587	Biso	1	C
H33	1	0.38881	0.82796	0.53542	Biso	1	H
H34	1	0.35496	0.70156	0.46975	Biso	1	H
H35	1	0.26959	0.76055	0.47876	Biso	1	H

C99	1	0.45891	0.74738	0.51484	Biso	1	C
C100	1	0.48229	0.70462	0.50127	Biso	1	C
H36	1	0.47447	0.76857	0.52805	Biso	1	H
S9	1	0.45016	0.67812	0.48324	Biso	1	S
S10	1	0.29331	0.85138	0.52533	Biso	1	S
C101	1	0.71502	0.67978	0.50188	Biso	1	C
C102	1	0.71361	0.64004	0.50321	Biso	1	C
C103	1	0.67276	0.63755	0.5031	Biso	1	C
C104	1	0.63479	0.67502	0.50771	Biso	1	C
C105	1	0.75205	0.60252	0.50363	Biso	1	C
C106	1	0.67096	0.59771	0.49793	Biso	1	C
C107	1	0.71028	0.55932	0.49779	Biso	1	C
C108	1	0.75028	0.56181	0.50282	Biso	1	C
C109	1	0.70874	0.51963	0.49429	Biso	1	C
C110	1	0.63109	0.59552	0.49426	Biso	1	C
C111	1	0.59474	0.63271	0.49678	Biso	1	C
C112	1	0.59664	0.67165	0.50435	Biso	1	C
C113	1	0.75431	0.6808	0.50237	Biso	1	C
C114	1	0.79201	0.64402	0.50367	Biso	1	C
C115	1	0.79187	0.60479	0.50384	Biso	1	C
C116	1	0.78858	0.52469	0.50614	Biso	1	C
C117	1	0.74671	0.48414	0.4969	Biso	1	C
C118	1	0.78581	0.48669	0.50322	Biso	1	C
H37	1	0.62818	0.56577	0.48966	Biso	1	H
H38	1	0.67891	0.51624	0.48963	Biso	1	H
H39	1	0.81982	0.52508	0.51146	Biso	1	H
H40	1	0.63465	0.7061	0.51438	Biso	1	H
H41	1	0.68647	0.7093	0.49979	Biso	1	H
H42	1	0.82211	0.57697	0.50352	Biso	1	H
O19	1	0.55251	0.63929	0.49422	Biso	1	O
O20	1	0.55585	0.7032	0.50737	Biso	1	O
O21	1	0.76406	0.71513	0.50111	Biso	1	O
O22	1	0.82608	0.65466	0.50353	Biso	1	O
O23	1	0.81811	0.44662	0.50535	Biso	1	O
O24	1	0.75393	0.4422	0.49496	Biso	1	O
B10	1	0.52946	0.68259	0.50085	Biso	1	B
B11	1	0.80814	0.69825	0.50195	Biso	1	B
B12	1	0.79783	0.42002	0.5002	Biso	1	B
C119	1	0.90932	0.8036	0.49704	Biso	1	C
C120	1	0.92484	0.76025	0.50192	Biso	1	C
C121	1	0.89186	0.74662	0.50146	Biso	1	C
C122	1	0.85071	0.78036	0.49639	Biso	1	C
C123	1	0.83352	0.72334	0.50096	Biso	1	C

C124	1	0.81778	0.76675	0.49631	Biso	1	C
H43	1	0.95886	0.74007	0.50611	Biso	1	H
H44	1	0.78375	0.78685	0.49276	Biso	1	H
S11	1	0.88852	0.6993	0.50549	Biso	1	S
S12	1	0.85411	0.82778	0.49186	Biso	1	S

Supplementary Table 15 | Coordinates for the 0.8-Å slipped AA stacking structure of the MC-COF-TP-$E_3^2E_6^1$

		x	*y*	*z*			
C1	1	0.07421	0.79039	0.95354	Biso	1	C
C2	1	0.07272	0.83125	0.95324	Biso	1	C
C3	1	0.11585	0.82913	0.94412	Biso	1	C
C4	1	0.15895	0.78624	0.93654	Biso	1	C
C5	1	0.02921	0.8743	0.9624	Biso	1	C
C6	1	0.11465	0.8701	0.94405	Biso	1	C
C7	1	0.07026	0.91406	0.95607	Biso	1	C
C8	1	0.02801	0.91615	0.96554	Biso	1	C
C9	1	0.06929	0.95462	0.96125	Biso	1	C
C10	1	0.15656	0.86813	0.93513	Biso	1	C
C11	1	0.19785	0.82578	0.92908	Biso	1	C
C12	1	0.19898	0.78566	0.93003	Biso	1	C
C13	1	0.0327	0.79378	0.96315	Biso	1	C
C14	1	0.99004	0.83593	0.97064	Biso	1	C
C15	1	0.98722	0.87642	0.97016	Biso	1	C
C16	1	0.98486	0.95882	0.9801	Biso	1	C
C17	1	0.02666	0.99548	0.97558	Biso	1	C
C18	1	0.98529	0.99754	0.98471	Biso	1	C
H1	1	0.15736	0.89854	0.93416	Biso	1	H
H2	1	0.10067	0.95471	0.95551	Biso	1	H
H3	1	0.95169	0.96214	0.98978	Biso	1	H
H4	1	0.16166	0.75398	0.93706	Biso	1	H
H5	1	0.10664	0.75671	0.94678	Biso	1	H
H6	1	0.95313	0.90854	0.97631	Biso	1	H
O1	1	0.2429	0.81463	0.92476	Biso	1	O
O2	1	0.2447	0.74869	0.92627	Biso	1	O
O3	1	0.02498	0.75943	0.96751	Biso	1	O
O4	1	0.95484	0.82867	0.9794	Biso	1	O
O5	1	0.94846	0.0426	0.99998	Biso	1	O
O6	1	0.0165	0.03924	0.98526	Biso	1	O
B1	1	0.27079	0.76737	0.92483	Biso	1	B
B2	1	0.97712	0.7817	0.97805	Biso	1	B
B3	1	0.9685	0.06729	0.00038	Biso	1	B
C19	1	0.94145	0.11773	0.01542	Biso	1	C
C20	1	0.95962	0.14398	0.02085	Biso	1	C
C21	1	0.92439	0.19098	0.03386	Biso	1	C
C22	1	0.87905	0.20016	0.03861	Biso	1	C
C23	1	0.86198	0.27354	0.05417	Biso	1	C
C24	1	0.84387	0.24718	0.0505	Biso	1	C

H7	1	0.99695	0.12874	0.01498	Biso	1	H
H8	1	0.80653	0.26244	0.05593	Biso	1	H
S1	1	0.9222	0.24053	0.04442	Biso	1	S
S2	1	0.88129	0.15044	0.02727	Biso	1	S
C25	1	0.48155	0.65153	0.97258	Biso	1	C
C26	1	0.45238	0.69858	0.96433	Biso	1	C
C27	1	0.40513	0.71391	0.95005	Biso	1	C
C28	1	0.39864	0.67769	0.94692	Biso	1	C
C29	1	0.32235	0.7401	0.92925	Biso	1	C
C30	1	0.35144	0.69298	0.93481	Biso	1	C
H9	1	0.4658	0.72007	0.96937	Biso	1	H
H10	1	0.33791	0.67159	0.93105	Biso	1	H
S3	1	0.3528	0.76556	0.9383	Biso	1	S
S4	1	0.45104	0.62594	0.96166	Biso	1	S
C31	1	0.72976	0.60135	0.04081	Biso	1	C
C32	1	0.73095	0.56063	0.04279	Biso	1	C
C33	1	0.6877	0.56295	0.03624	Biso	1	C
C34	1	0.6447	0.60583	0.02195	Biso	1	C
C35	1	0.77437	0.51742	0.04868	Biso	1	C
C36	1	0.68878	0.52203	0.0413	Biso	1	C
C37	1	0.73309	0.47793	0.05229	Biso	1	C
C38	1	0.77548	0.4756	0.05309	Biso	1	C
C39	1	0.73399	0.43736	0.05848	Biso	1	C
C40	1	0.64685	0.52409	0.03168	Biso	1	C
C41	1	0.60571	0.56638	0.01721	Biso	1	C
C42	1	0.60465	0.60646	0.01261	Biso	1	C
C43	1	0.77148	0.59765	0.04221	Biso	1	C
C44	1	0.81402	0.55533	0.04598	Biso	1	C
C45	1	0.81653	0.51503	0.05004	Biso	1	C
C46	1	0.81866	0.43269	0.05735	Biso	1	C
C47	1	0.77662	0.39627	0.06146	Biso	1	C
C48	1	0.81818	0.39397	0.06023	Biso	1	C
H11	1	0.64597	0.49373	0.03379	Biso	1	H
H12	1	0.70246	0.43748	0.05999	Biso	1	H
H13	1	0.85197	0.42917	0.05705	Biso	1	H
H14	1	0.64218	0.63798	0.01576	Biso	1	H
H15	1	0.6974	0.63514	0.03779	Biso	1	H
H16	1	0.85053	0.4828	0.05382	Biso	1	H
O7	1	0.56082	0.57736	0.00325	Biso	1	O
O8	1	0.55896	0.64331	0.99593	Biso	1	O
O9	1	0.77973	0.63171	0.03866	Biso	1	O
O10	1	0.84956	0.56214	0.04463	Biso	1	O
O11	1	0.85501	0.34875	0.06046	Biso	1	O

O12	1	0.78676	0.35245	0.06267	Biso	1	O
B4	1	0.53295	0.62452	0.99024	Biso	1	B
B5	1	0.82774	0.60903	0.03948	Biso	1	B
B6	1	0.8349	0.3241	0.06043	Biso	1	B
C49	1	0.94053	0.69939	0.004	Biso	1	C
C50	1	0.89365	0.73867	0.00432	Biso	1	C
C51	1	0.85574	0.73492	0.01208	Biso	1	C
C52	1	0.86487	0.69102	0.01986	Biso	1	C
C53	1	0.91175	0.65175	0.02049	Biso	1	C
C54	1	0.94965	0.65548	0.01255	Biso	1	C
C55	1	0.97305	0.71194	0.99413	Biso	1	C
C56	1	0.95254	0.75825	0.98767	Biso	1	C
H17	1	0.82004	0.76511	0.01172	Biso	1	H
H18	1	0.98536	0.6253	0.0127	Biso	1	H
H19	1	0.01078	0.68816	0.99199	Biso	1	H
C57	1	0.83237	0.67837	0.02739	Biso	1	C
C58	1	0.85272	0.63211	0.03359	Biso	1	C
H20	1	0.79463	0.70219	0.02766	Biso	1	H
S5	1	0.91296	0.60204	0.03079	Biso	1	S
S6	1	0.89249	0.78823	0.99352	Biso	1	S
C59	1	0.05076	0.83353	0.45688	Biso	1	C
C60	1	0.04942	0.87433	0.45816	Biso	1	C
C61	1	0.09256	0.8722	0.44921	Biso	1	C
C62	1	0.13572	0.82935	0.43979	Biso	1	C
C63	1	0.00596	0.91745	0.46814	Biso	1	C
C64	1	0.09126	0.91324	0.45062	Biso	1	C
C65	1	0.04693	0.95724	0.46501	Biso	1	C
C66	1	0.00469	0.95935	0.47345	Biso	1	C
C67	1	0.04591	0.99787	0.47237	Biso	1	C
C68	1	0.13311	0.91135	0.44018	Biso	1	C
C69	1	0.17442	0.86907	0.43186	Biso	1	C
C70	1	0.17571	0.82886	0.43225	Biso	1	C
C71	1	0.00915	0.83705	0.46518	Biso	1	C
C72	1	0.96657	0.87929	0.47288	Biso	1	C
C73	1	0.9639	0.91967	0.47451	Biso	1	C
C74	1	0.96152	0.00209	0.48808	Biso	1	C
C75	1	0.00331	0.03876	0.48818	Biso	1	C
C76	1	0.96189	0.04085	0.49526	Biso	1	C
H21	1	0.13383	0.9418	0.43955	Biso	1	H
H22	1	0.0773	0.99793	0.46638	Biso	1	H
H23	1	0.92831	0.00544	0.49454	Biso	1	H
H24	1	0.13846	0.79707	0.4394	Biso	1	H
H25	1	0.08315	0.79982	0.44978	Biso	1	H

H26	1	0.92987	0.95183	0.48125	Biso	1	H
O13	1	0.21936	0.85819	0.42574	Biso	1	O
O14	1	0.2216	0.79202	0.42656	Biso	1	O
O15	1	0.00107	0.80287	0.46743	Biso	1	O
O16	1	0.93116	0.87232	0.47959	Biso	1	O
O17	1	0.92507	0.08591	0.51015	Biso	1	O
O18	1	0.99311	0.08254	0.4991	Biso	1	O
B7	1	0.24751	0.81095	0.4244	Biso	1	B
B8	1	0.95312	0.82541	0.47662	Biso	1	B
B9	1	0.94505	0.11066	0.51237	Biso	1	B
C77	1	0.91793	0.16115	0.52648	Biso	1	C
C78	1	0.93599	0.18755	0.53122	Biso	1	C
C79	1	0.90076	0.23454	0.54305	Biso	1	C
C80	1	0.85544	0.24368	0.54691	Biso	1	C
C81	1	0.83824	0.31703	0.5591	Biso	1	C
C82	1	0.82012	0.29072	0.55589	Biso	1	C
H27	1	0.97332	0.17233	0.52575	Biso	1	H
H28	1	0.78279	0.30594	0.55923	Biso	1	H
S7	1	0.89844	0.28433	0.55137	Biso	1	S
S8	1	0.85771	0.19408	0.53624	Biso	1	S
C83	1	0.45834	0.69547	0.46585	Biso	1	C
C84	1	0.42926	0.74257	0.45846	Biso	1	C
C85	1	0.38208	0.75782	0.44531	Biso	1	C
C86	1	0.37563	0.72158	0.44246	Biso	1	C
C87	1	0.29908	0.78395	0.4268	Biso	1	C
C88	1	0.32834	0.73689	0.43174	Biso	1	C
H29	1	0.44274	0.76399	0.46352	Biso	1	H
H30	1	0.31491	0.7154	0.42897	Biso	1	H
S9	1	0.32959	0.80957	0.43446	Biso	1	S
S10	1	0.42793	0.66996	0.45574	Biso	1	S
C89	1	0.70635	0.64445	0.53903	Biso	1	C
C90	1	0.70766	0.60369	0.53906	Biso	1	C
C91	1	0.66443	0.60603	0.53055	Biso	1	C
C92	1	0.62152	0.64896	0.5171	Biso	1	C
C93	1	0.75112	0.56053	0.54407	Biso	1	C
C94	1	0.6654	0.56518	0.53287	Biso	1	C
C95	1	0.70969	0.52114	0.54495	Biso	1	C
C96	1	0.7521	0.51879	0.54785	Biso	1	C
C97	1	0.71043	0.48071	0.55158	Biso	1	C
C98	1	0.62344	0.56732	0.52118	Biso	1	C
C99	1	0.58236	0.6097	0.50753	Biso	1	C
C100	1	0.58145	0.6497	0.50587	Biso	1	C
C101	1	0.74801	0.64084	0.54028	Biso	1	C

C102	1	0.79062	0.5986	0.5424	Biso	1	C
C103	1	0.79325	0.55821	0.54543	Biso	1	C
C104	1	0.79524	0.47595	0.55449	Biso	1	C
C105	1	0.75304	0.43967	0.55749	Biso	1	C
C106	1	0.7946	0.43734	0.55802	Biso	1	C
H31	1	0.62247	0.53701	0.52152	Biso	1	H
H32	1	0.6789	0.48085	0.55173	Biso	1	H
H33	1	0.82857	0.47239	0.55644	Biso	1	H
H34	1	0.61902	0.68112	0.51401	Biso	1	H
H35	1	0.67397	0.67821	0.53719	Biso	1	H
H36	1	0.82729	0.526	0.54785	Biso	1	H
O19	1	0.53738	0.62096	0.49278	Biso	1	O
O20	1	0.53594	0.68671	0.49026	Biso	1	O
O21	1	0.75592	0.67506	0.53726	Biso	1	O
O22	1	0.82599	0.60567	0.54043	Biso	1	O
O23	1	0.8314	0.39211	0.56148	Biso	1	O
O24	1	0.76307	0.39592	0.56096	Biso	1	O
B10	1	0.50975	0.66817	0.48245	Biso	1	B
B11	1	0.80386	0.65262	0.53656	Biso	1	B
B12	1	0.81118	0.36759	0.56195	Biso	1	B
C107	1	0.91613	0.74331	0.4992	Biso	1	C
C108	1	0.86924	0.78257	0.4993	Biso	1	C
C109	1	0.83136	0.77882	0.50729	Biso	1	C
C110	1	0.84051	0.7349	0.51533	Biso	1	C
C111	1	0.88739	0.69563	0.5154	Biso	1	C
C112	1	0.92529	0.69941	0.50746	Biso	1	C
C113	1	0.94861	0.75597	0.49011	Biso	1	C
C114	1	0.92824	0.80224	0.48408	Biso	1	C
H37	1	0.79564	0.80899	0.5072	Biso	1	H
H38	1	0.961	0.66923	0.50769	Biso	1	H
H39	1	0.98635	0.73215	0.48875	Biso	1	H
C115	1	0.80801	0.7223	0.52376	Biso	1	C
C116	1	0.82854	0.67599	0.52996	Biso	1	C
H40	1	0.77027	0.74605	0.52491	Biso	1	H
S11	1	0.88859	0.64605	0.52589	Biso	1	S
S12	1	0.868	0.83229	0.489	Biso	1	S

Supplementary Table 16 | Coordinates for the 0.8-Å slipped AA stacking structure of the MC-COF-TP-$E_1E_3E_7$

		x	*y*	*z*			
C1	1	0.02965	0.80655	0.9991	Biso	1	C
C2	1	0.03186	0.84738	0.99893	Biso	1	C
C3	1	0.07417	0.85055	0.99955	Biso	1	C
C4	1	0.11277	0.81279	0.99758	Biso	1	C
C5	1	0.99284	0.88522	0.99861	Biso	1	C
C6	1	0.07674	0.89149	0.00239	Biso	1	C
C7	1	0.03685	0.93015	0.00239	Biso	1	C
C8	1	0.99539	0.92709	0.99925	Biso	1	C
C9	1	0.03937	0.97084	0.00459	Biso	1	C
C10	1	0.11795	0.89453	0.00437	Biso	1	C
C11	1	0.15485	0.85708	0.00287	Biso	1	C
C12	1	0.15224	0.81698	0.99924	Biso	1	C
C13	1	0.98907	0.80475	0.99872	Biso	1	C
C14	1	0.95079	0.84181	0.9983	Biso	1	C
C15	1	0.95168	0.88209	0.9983	Biso	1	C
C16	1	0.95659	0.96469	0.99714	Biso	1	C
C17	1	0.00094	0.00668	0.00294	Biso	1	C
C18	1	0.96032	0.00372	0.99896	Biso	1	C
H1	1	0.12146	0.92516	0.00689	Biso	1	H
H2	1	0.07027	0.97483	0.00746	Biso	1	H
H3	1	0.92421	0.96389	0.99379	Biso	1	H
H4	1	0.11245	0.7808	0.99454	Biso	1	H
H5	1	0.05863	0.77677	0.99987	Biso	1	H
H6	1	0.92095	0.91012	0.9984	Biso	1	H
O1	1	0.19826	0.85129	0.00395	Biso	1	O
O2	1	0.19381	0.78536	0.99785	Biso	1	O
O3	1	0.97843	0.76936	0.99912	Biso	1	O
O4	1	0.91537	0.83028	0.99841	Biso	1	O
O5	1	0.92791	0.04452	0.99749	Biso	1	O
O6	1	0.99485	0.04928	0.0041	Biso	1	O
B1	1	0.22136	0.80718	0.00083	Biso	1	B
B2	1	0.93316	0.78577	0.99896	Biso	1	B
B3	1	0.94993	0.0718	0.00067	Biso	1	B
C19	1	0.92911	0.11944	0.0004	Biso	1	C
C20	1	0.88387	0.1416	0.99853	Biso	1	C
C21	1	0.86493	0.18644	0.99836	Biso	1	C
C22	1	0.89256	0.20955	0.00012	Biso	1	C
C23	1	0.93869	0.18708	0.00197	Biso	1	C
C24	1	0.956	0.14228	0.00206	Biso	1	C
C25	1	0.8186	0.20996	0.99648	Biso	1	C

C26	1	0.87414	0.25504	0.00003	Biso	1	C
C27	1	0.82803	0.27752	0.99811	Biso	1	C
C28	1	0.80094	0.25355	0.99635	Biso	1	C
C29	1	0.81073	0.32232	0.998	Biso	1	C
H7	1	0.77544	0.34086	0.99653	Biso	1	H
C30	1	0.83763	0.34514	0.9998	Biso	1	C
C31	1	0.88283	0.32297	0.00176	Biso	1	C
C32	1	0.90177	0.27813	0.00187	Biso	1	C
C33	1	0.94809	0.25465	0.00372	Biso	1	C
C34	1	0.96578	0.21106	0.00372	Biso	1	C
H8	1	0.00129	0.19371	0.00516	Biso	1	H
H9	1	0.96938	0.27239	0.00515	Biso	1	H
H10	1	0.79726	0.19228	0.99508	Biso	1	H
H11	1	0.86345	0.12298	0.99724	Biso	1	H
H12	1	0.99128	0.12374	0.00345	Biso	1	H
H13	1	0.76543	0.27094	0.99485	Biso	1	H
H14	1	0.90324	0.3416	0.00315	Biso	1	H
C35	1	0.34077	0.73319	0.99701	Biso	1	C
C36	1	0.34942	0.77129	0.00189	Biso	1	C
C37	1	0.39175	0.76994	0.00368	Biso	1	C
C38	1	0.42621	0.72961	0.00046	Biso	1	C
C39	1	0.41765	0.69146	0.99522	Biso	1	C
C40	1	0.37532	0.69285	0.99352	Biso	1	C
C41	1	0.29509	0.74289	0.99637	Biso	1	C
C42	1	0.26975	0.7861	0.00043	Biso	1	C
H15	1	0.39792	0.7993	0.00754	Biso	1	H
H16	1	0.36915	0.66349	0.98949	Biso	1	H
H17	1	0.28095	0.7188	0.99288	Biso	1	H
C43	1	0.47188	0.72003	0.00163	Biso	1	C
C44	1	0.49737	0.67692	0.99731	Biso	1	C
H18	1	0.48584	0.74427	0.00572	Biso	1	H
S1	1	0.46605	0.64629	0.99173	Biso	1	S
S2	1	0.3013	0.81627	0.00534	Biso	1	S
C45	1	0.7372	0.6582	0.00085	Biso	1	C
C46	1	0.73509	0.61732	0.00076	Biso	1	C
C47	1	0.69281	0.61413	0.99952	Biso	1	C
C48	1	0.6541	0.65182	0.00108	Biso	1	C
C49	1	0.77416	0.5795	0.00127	Biso	1	C
C50	1	0.69045	0.57312	0.99657	Biso	1	C
C51	1	0.73032	0.53449	0.99712	Biso	1	C
C52	1	0.77175	0.53756	0.0005	Biso	1	C
C53	1	0.72773	0.49382	0.99522	Biso	1	C
C54	1	0.64938	0.56985	0.994	Biso	1	C

C55	1	0.61243	0.60717	0.99521	Biso	1	C
C56	1	0.61476	0.6474	0.99901	Biso	1	C
C57	1	0.77774	0.66003	0.00152	Biso	1	C
C58	1	0.81608	0.62302	0.00204	Biso	1	C
C59	1	0.81528	0.58271	0.00192	Biso	1	C
C60	1	0.81052	0.49986	0.0027	Biso	1	C
C61	1	0.76609	0.45794	0.99713	Biso	1	C
C62	1	0.80676	0.46083	0.00093	Biso	1	C
H19	1	0.64594	0.5392	0.99128	Biso	1	H
H20	1	0.69679	0.4899	0.9924	Biso	1	H
H21	1	0.84292	0.50064	0.00597	Biso	1	H
H22	1	0.65431	0.68387	0.00423	Biso	1	H
H23	1	0.70819	0.68796	0.99996	Biso	1	H
H24	1	0.84602	0.5547	0.00194	Biso	1	H
O7	1	0.56923	0.61247	0.99383	Biso	1	O
O8	1	0.57295	0.67875	0.00028	Biso	1	O
O9	1	0.78833	0.69549	0.00122	Biso	1	O
O10	1	0.85145	0.63462	0.0021	Biso	1	O
O11	1	0.83906	0.42	0.00245	Biso	1	O
O12	1	0.77203	0.41541	0.99619	Biso	1	O
B4	1	0.54579	0.65646	0.99716	Biso	1	B
B5	1	0.83362	0.67911	0.00149	Biso	1	B
B6	1	0.81694	0.39278	0.9995	Biso	1	B
C63	1	0.85938	0.70637	0.00092	Biso	1	C
C64	1	0.83813	0.75105	0.00035	Biso	1	C
C65	1	0.86167	0.77681	0.99967	Biso	1	C
C66	1	0.90729	0.7586	0.99953	Biso	1	C
C67	1	0.92854	0.71393	0.00009	Biso	1	C
C68	1	0.90501	0.68818	0.00077	Biso	1	C
H25	1	0.80244	0.76506	0.00047	Biso	1	H
H26	1	0.84507	0.81175	0.99923	Biso	1	H
H27	1	0.96423	0.69995	0.99997	Biso	1	H
H28	1	0.9216	0.65324	0.00119	Biso	1	H
C69	1	0.01684	0.83901	0.49918	Biso	1	C
C70	1	0.01889	0.87991	0.49926	Biso	1	C
C71	1	0.06114	0.88316	0.50046	Biso	1	C
C72	1	0.09988	0.8455	0.4989	Biso	1	C
C73	1	0.97979	0.9177	0.49878	Biso	1	C
C74	1	0.06344	0.9242	0.50338	Biso	1	C
C75	1	0.02354	0.96279	0.50284	Biso	1	C
C76	1	0.98214	0.95967	0.49951	Biso	1	C
C77	1	0.02607	0.0035	0.50472	Biso	1	C
C78	1	0.10449	0.92752	0.50591	Biso	1	C

C79	1	0.14147	0.89024	0.5047	Biso	1	C
C80	1	0.1392	0.84998	0.50093	Biso	1	C
C81	1	0.97633	0.83711	0.49854	Biso	1	C
C82	1	0.93796	0.8741	0.49805	Biso	1	C
C83	1	0.93869	0.91443	0.49818	Biso	1	C
C84	1	0.94333	0.99733	0.49734	Biso	1	C
C85	1	0.98768	0.03934	0.50284	Biso	1	C
C86	1	0.94703	0.03639	0.49909	Biso	1	C
H29	1	0.10788	0.9582	0.50862	Biso	1	H
H30	1	0.05698	0.00746	0.5075	Biso	1	H
H31	1	0.91095	0.99651	0.4941	Biso	1	H
H32	1	0.09973	0.81344	0.49578	Biso	1	H
H33	1	0.04588	0.80927	0.50004	Biso	1	H
H34	1	0.90793	0.94241	0.49819	Biso	1	H
O13	1	0.18465	0.88499	0.50605	Biso	1	O
O14	1	0.18103	0.81867	0.49965	Biso	1	O
O15	1	0.9658	0.80162	0.49884	Biso	1	O
O16	1	0.90262	0.86244	0.49802	Biso	1	O
O17	1	0.91469	0.0772	0.4976	Biso	1	O
O18	1	0.98168	0.08189	0.50379	Biso	1	O
B7	1	0.20814	0.84101	0.50273	Biso	1	B
B8	1	0.9205	0.81795	0.4986	Biso	1	B
B9	1	0.93676	0.10446	0.50053	Biso	1	B
C87	1	0.91604	0.1521	0.50027	Biso	1	C
C88	1	0.87083	0.17427	0.49832	Biso	1	C
C89	1	0.8519	0.21911	0.49824	Biso	1	C
C90	1	0.87952	0.24221	0.50008	Biso	1	C
C91	1	0.92564	0.21973	0.502	Biso	1	C
C92	1	0.94293	0.17493	0.50209	Biso	1	C
C93	1	0.80558	0.24259	0.49641	Biso	1	C
C94	1	0.8611	0.2877	0.49999	Biso	1	C
C95	1	0.81498	0.31017	0.49813	Biso	1	C
C96	1	0.78788	0.28619	0.4964	Biso	1	C
C97	1	0.79766	0.35496	0.498	Biso	1	C
H35	1	0.76238	0.3735	0.49659	Biso	1	H
C98	1	0.82456	0.37781	0.49964	Biso	1	C
C99	1	0.86979	0.35564	0.50153	Biso	1	C
C100	1	0.88874	0.3108	0.50174	Biso	1	C
C101	1	0.93507	0.28729	0.50363	Biso	1	C
C102	1	0.95273	0.24369	0.50378	Biso	1	C
H36	1	0.98823	0.2263	0.50529	Biso	1	H
H37	1	0.95641	0.30497	0.50505	Biso	1	H
H38	1	0.78429	0.22486	0.49499	Biso	1	H

H39	1	0.85042	0.15564	0.49693	Biso	1	H
H40	1	0.97822	0.15639	0.50355	Biso	1	H
H41	1	0.75238	0.30353	0.49497	Biso	1	H
H42	1	0.89021	0.37426	0.50281	Biso	1	H
C103	1	0.32773	0.76789	0.4994	Biso	1	C
C104	1	0.33629	0.80603	0.50463	Biso	1	C
C105	1	0.37862	0.80465	0.50633	Biso	1	C
C106	1	0.41316	0.76431	0.50284	Biso	1	C
C107	1	0.40452	0.72621	0.49796	Biso	1	C
C108	1	0.36219	0.72756	0.49617	Biso	1	C
C109	1	0.28206	0.77747	0.49823	Biso	1	C
C110	1	0.25656	0.82058	0.50256	Biso	1	C
H43	1	0.38479	0.834	0.51036	Biso	1	H
H44	1	0.35602	0.6982	0.49231	Biso	1	H
H45	1	0.2681	0.75322	0.49416	Biso	1	H
C111	1	0.45885	0.75461	0.50349	Biso	1	C
C112	1	0.48418	0.71139	0.49944	Biso	1	C
H46	1	0.47299	0.77869	0.507	Biso	1	H
S3	1	0.45264	0.68123	0.49451	Biso	1	S
S4	1	0.28789	0.8512	0.50813	Biso	1	S
C113	1	0.72439	0.69066	0.50092	Biso	1	C
C114	1	0.72212	0.64986	0.50109	Biso	1	C
C115	1	0.67978	0.64675	0.50044	Biso	1	C
C116	1	0.64121	0.68453	0.50241	Biso	1	C
C117	1	0.76111	0.61198	0.50143	Biso	1	C
C118	1	0.67715	0.60583	0.49757	Biso	1	C
C119	1	0.71701	0.56713	0.49758	Biso	1	C
C120	1	0.75849	0.57014	0.50077	Biso	1	C
C121	1	0.71443	0.52647	0.49535	Biso	1	C
C122	1	0.63591	0.60285	0.49555	Biso	1	C
C123	1	0.59905	0.64033	0.49705	Biso	1	C
C124	1	0.60172	0.6804	0.5007	Biso	1	C
C125	1	0.765	0.6924	0.50132	Biso	1	C
C126	1	0.80325	0.6553	0.50179	Biso	1	C
C127	1	0.8023	0.61505	0.50179	Biso	1	C
C128	1	0.79726	0.53251	0.5029	Biso	1	C
C129	1	0.75283	0.4906	0.49702	Biso	1	C
C130	1	0.79348	0.4935	0.50105	Biso	1	C
H47	1	0.63236	0.57224	0.49301	Biso	1	H
H48	1	0.68351	0.52253	0.49244	Biso	1	H
H49	1	0.82967	0.53325	0.50629	Biso	1	H
H50	1	0.64158	0.71651	0.50548	Biso	1	H
H51	1	0.69544	0.72046	0.50012	Biso	1	H

H52	1	0.833	0.58699	0.50172	Biso	1	H
O19	1	0.55562	0.64617	0.49593	Biso	1	O
O20	1	0.56017	0.71206	0.50208	Biso	1	O
O21	1	0.77569	0.72776	0.50092	Biso	1	O
O22	1	0.8387	0.66678	0.50169	Biso	1	O
O23	1	0.82584	0.45268	0.50254	Biso	1	O
O24	1	0.75886	0.44801	0.49585	Biso	1	O
B10	1	0.53258	0.69029	0.49906	Biso	1	B
B11	1	0.82097	0.71129	0.50112	Biso	1	B
B12	1	0.80377	0.42544	0.49933	Biso	1	B
C131	1	0.84687	0.73843	0.50055	Biso	1	C
C132	1	0.82562	0.7831	0.49999	Biso	1	C
C133	1	0.84914	0.80886	0.49932	Biso	1	C
C134	1	0.89477	0.79066	0.49917	Biso	1	C
C135	1	0.91602	0.74598	0.49973	Biso	1	C
C136	1	0.89249	0.72023	0.50041	Biso	1	C
H53	1	0.78992	0.79708	0.50011	Biso	1	H
H54	1	0.83255	0.8438	0.49889	Biso	1	H
H55	1	0.95172	0.73197	0.49962	Biso	1	H
H56	1	0.90908	0.68529	0.50085	Biso	1	H

Supplementary Table 17 | Coordinates for the 0.8-Å slipped AA stacking structure of the MC-COF-NiPc-E_1E_7

		x	*y*	*z*			
C1	1	0.95904	0.84666	1	Biso	1	C
C2	1	0.02072	0.84709	1	Biso	1	C
C3	1	0.05449	0.80405	0	Biso	1	C
C4	1	0.02219	0.76095	0	Biso	1	C
C5	1	0.9609	0.76022	0	Biso	1	C
C6	1	0.92704	0.80258	1	Biso	1	C
C7	1	0.9407	0.89771	0.99999	Biso	1	C
C8	1	0.03833	0.8981	1	Biso	1	C
H1	1	0.10207	0.80465	0	Biso	1	H
H2	1	0.87946	0.80178	1	Biso	1	H
C9	1	0.80813	0.97392	0.99999	Biso	1	C
C10	1	0.80812	0.026	0.99999	Biso	1	C
C11	1	0.75644	0.05371	1	Biso	1	C
C12	1	0.70581	0.02579	0	Biso	1	C
C13	1	0.70583	0.97405	1	Biso	1	C
C14	1	0.75649	0.94616	0.99999	Biso	1	C
C15	1	0.86862	0.9587	0.99999	Biso	1	C
C16	1	0.8686	0.04124	0.99999	Biso	1	C
H3	1	0.75624	0.09388	1	Biso	1	H
H4	1	0.75634	0.906	0.99998	Biso	1	H
C17	1	0.02072	0.15286	0	Biso	1	C
C18	1	0.95904	0.15329	0	Biso	1	C
C19	1	0.92704	0.19737	0	Biso	1	C
C20	1	0.9609	0.23973	0	Biso	1	C
C21	1	0.02219	0.239	0	Biso	1	C
C22	1	0.05449	0.1959	0	Biso	1	C
C23	1	0.03834	0.10184	0	Biso	1	C
C24	1	0.9407	0.10224	1	Biso	1	C
H5	1	0.87946	0.19817	0	Biso	1	H
H6	1	0.10207	0.1953	0	Biso	1	H
C25	1	0.17091	0.97392	0	Biso	1	C
C26	1	0.17093	0.026	0.00001	Biso	1	C
C27	1	0.22254	0.05377	0	Biso	1	C
C28	1	0.2731	0.02578	1	Biso	1	C
C29	1	0.27308	0.97405	1	Biso	1	C
C30	1	0.22249	0.9461	1	Biso	1	C
C31	1	0.1105	0.95877	0	Biso	1	C
C32	1	0.11051	0.04116	0.00001	Biso	1	C
H7	1	0.22269	0.09394	0.00001	Biso	1	H

H8	1	0.22259	0.90594	0.99999	Biso	1	H
N1	1	0.88528	0.91187	0.99999	Biso	1	N
N2	1	0.88527	0.08807	1	Biso	1	N
N3	1	0.0938	0.0879	0.00001	Biso	1	N
N4	1	0.09379	0.91204	0	Biso	1	N
N5	1	0.98939	0.92793	1	Biso	1	N
N6	1	0.90411	0.99997	0.99999	Biso	1	N
N7	1	0.98939	0.07201	0	Biso	1	N
N8	1	0.07482	0.99997	0	Biso	1	N
O1	1	0.04246	0.71313	0	Biso	1	O
O2	1	0.94256	0.71175	0	Biso	1	O
O3	1	0.64876	0.04206	0.00001	Biso	1	O
O4	1	0.6488	0.95774	0	Biso	1	O
O5	1	0.94256	0.2882	0	Biso	1	O
O6	1	0.04246	0.28682	0	Biso	1	O
O7	1	0.3302	0.04206	1	Biso	1	O
O8	1	0.33016	0.95774	0.99999	Biso	1	O
B1	1	0.99308	0.31625	0	Biso	1	B
B2	1	0.36423	0.99989	0.99999	Biso	1	B
B3	1	0.99308	0.68371	0	Biso	1	B
B4	1	0.61469	0.99989	0	Biso	1	B
Ni1	1	0.98938	0.99997	1	Biso	1	Ni
C33	1	0.42907	0.99987	0.99999	Biso	1	C
C34	1	0.45927	0.95535	0.00001	Biso	1	C
C35	1	0.51963	0.95538	0.00001	Biso	1	C
C36	1	0.54984	0.99987	0	Biso	1	C
C37	1	0.51963	0.04436	0.99999	Biso	1	C
C38	1	0.45927	0.04439	0.99999	Biso	1	C
H9	1	0.43257	0.92206	0.00002	Biso	1	H
H10	1	0.54631	0.92207	0.00002	Biso	1	H
H11	1	0.54632	0.07767	0.99999	Biso	1	H
H12	1	0.43256	0.07767	0.99998	Biso	1	H
C39	1	0.99448	0.37097	1	Biso	1	C
C40	1	0.94199	0.39626	1	Biso	1	C
C41	1	0.94058	0.44774	1	Biso	1	C
C42	1	0.99454	0.47383	1	Biso	1	C
C43	1	0.04844	0.44765	1	Biso	1	C
C44	1	0.04723	0.39615	1	Biso	1	C
C45	1	0.88711	0.47494	1	Biso	1	C
C46	1	0.99454	0.52614	1	Biso	1	C
C47	1	0.94058	0.55223	1	Biso	1	C
C48	1	0.88711	0.52503	1	Biso	1	C
C49	1	0.94199	0.60371	0	Biso	1	C

H13	1	0.90324	0.62715	0	Biso	1	H
C50	1	0.99448	0.62899	0	Biso	1	C
C51	1	0.04723	0.60381	0	Biso	1	C
C52	1	0.04844	0.55232	1	Biso	1	C
C53	1	0.10188	0.52502	1	Biso	1	C
C54	1	0.10187	0.47495	1	Biso	1	C
H14	1	0.14321	0.4548	1	Biso	1	H
H15	1	0.1432	0.54518	0	Biso	1	H
H16	1	0.8458	0.45474	1	Biso	1	H
H17	1	0.90324	0.37283	1	Biso	1	H
H18	1	0.0866	0.37347	1	Biso	1	H
H19	1	0.8458	0.54523	0	Biso	1	H
H20	1	0.08659	0.6265	0	Biso	1	H
C55	1	0.0226	0.84709	0.5	Biso	1	C
C56	1	0.08428	0.84666	0.5	Biso	1	C
C57	1	0.11627	0.80258	0.5	Biso	1	C
C58	1	0.08242	0.76022	0.5	Biso	1	C
C59	1	0.02113	0.76095	0.5	Biso	1	C
C60	1	0.98882	0.80405	0.5	Biso	1	C
C61	1	0.00498	0.8981	0.5	Biso	1	C
C62	1	0.10261	0.89771	0.5	Biso	1	C
H21	1	0.16386	0.80178	0.5	Biso	1	H
H22	1	0.94125	0.80465	0.5	Biso	1	H
C63	1	0.8724	0.97392	0.49999	Biso	1	C
C64	1	0.87239	0.026	0.5	Biso	1	C
C65	1	0.82077	0.05377	0.5	Biso	1	C
C66	1	0.77021	0.02579	0.5	Biso	1	C
C67	1	0.77023	0.97405	0.5	Biso	1	C
C68	1	0.82082	0.9461	0.49999	Biso	1	C
C69	1	0.93281	0.95877	0.49999	Biso	1	C
C70	1	0.9328	0.04116	0.5	Biso	1	C
H23	1	0.82063	0.09394	0.50001	Biso	1	H
H24	1	0.82073	0.90594	0.49999	Biso	1	H
C71	1	0.08428	0.15329	0.5	Biso	1	C
C72	1	0.02259	0.15286	0.5	Biso	1	C
C73	1	0.98883	0.1959	0.5	Biso	1	C
C74	1	0.02113	0.239	0.5	Biso	1	C
C75	1	0.08242	0.23973	0.5	Biso	1	C
C76	1	0.11627	0.19737	0.5	Biso	1	C
C77	1	0.10262	0.10224	0.50001	Biso	1	C
C78	1	0.00498	0.10184	0.5	Biso	1	C
H25	1	0.94125	0.1953	0.5	Biso	1	H
H26	1	0.16385	0.19817	0.5	Biso	1	H

C79	1	0.23518	0.97392	0.5	Biso	1	C
C80	1	0.2352	0.026	0.5	Biso	1	C
C81	1	0.28688	0.05371	0.5	Biso	1	C
C82	1	0.33751	0.02579	0.5	Biso	1	C
C83	1	0.33749	0.97405	0.49999	Biso	1	C
C84	1	0.28683	0.94616	0.49999	Biso	1	C
C85	1	0.1747	0.9587	0.5	Biso	1	C
C86	1	0.17471	0.04124	0.50001	Biso	1	C
H27	1	0.28708	0.09388	0.50001	Biso	1	H
H28	1	0.28698	0.906	0.49999	Biso	1	H
N9	1	0.94952	0.91204	0.49999	Biso	1	N
N10	1	0.94952	0.0879	0.5	Biso	1	N
N11	1	0.15804	0.08807	0.50001	Biso	1	N
N12	1	0.15804	0.91187	0.5	Biso	1	N
N13	1	0.05393	0.92793	0.5	Biso	1	N
N14	1	0.96849	0.99997	0.5	Biso	1	N
N15	1	0.05393	0.07201	0.5	Biso	1	N
N16	1	0.1392	0.99997	0.5	Biso	1	N
O9	1	0.10076	0.71175	0.5	Biso	1	O
O10	1	0.00086	0.71313	0.5	Biso	1	O
O11	1	0.71312	0.04206	0.50001	Biso	1	O
O12	1	0.71315	0.95774	0.5	Biso	1	O
O13	1	0.00086	0.28682	0.5	Biso	1	O
O14	1	0.10075	0.2882	0.5	Biso	1	O
O15	1	0.39455	0.04206	0.49999	Biso	1	O
O16	1	0.39452	0.95774	0.49999	Biso	1	O
B5	1	0.05024	0.31625	0.5	Biso	1	B
B6	1	0.42862	0.99989	0.49999	Biso	1	B
B7	1	0.05024	0.68371	0.5	Biso	1	B
B8	1	0.67908	0.99989	0.50001	Biso	1	B
Ni2	1	0.05393	0.99997	0.5	Biso	1	Ni
C87	1	0.49347	0.99987	0.49999	Biso	1	C
C88	1	0.52368	0.95538	0.5	Biso	1	C
C89	1	0.58404	0.95535	0.50001	Biso	1	C
C90	1	0.61424	0.99987	0.5	Biso	1	C
C91	1	0.58404	0.04439	0.49999	Biso	1	C
C92	1	0.52368	0.04436	0.49998	Biso	1	C
H29	1	0.497	0.92207	0.50001	Biso	1	H
H30	1	0.61075	0.92206	0.50002	Biso	1	H
H31	1	0.61076	0.07767	0.49999	Biso	1	H
H32	1	0.49699	0.07767	0.49997	Biso	1	H
C93	1	0.04884	0.37097	0.5	Biso	1	C
C94	1	0.99609	0.39615	0.5	Biso	1	C

C95	1	0.99488	0.44765	0.5	Biso	1	C
C96	1	0.04878	0.47383	0.5	Biso	1	C
C97	1	0.10273	0.44774	0.5	Biso	1	C
C98	1	0.10133	0.39626	0.5	Biso	1	C
C99	1	0.94144	0.47495	0.5	Biso	1	C
C100	1	0.04878	0.52614	0.5	Biso	1	C
C101	1	0.99487	0.55232	0.5	Biso	1	C
C102	1	0.94144	0.52502	0.5	Biso	1	C
C103	1	0.99608	0.60381	0.5	Biso	1	C
H33	1	0.95672	0.6265	0.5	Biso	1	H
C104	1	0.04884	0.62899	0.5	Biso	1	C
C105	1	0.10133	0.60371	0.5	Biso	1	C
C106	1	0.10274	0.55223	0.5	Biso	1	C
C107	1	0.1562	0.52503	0.5	Biso	1	C
C108	1	0.1562	0.47494	0.5	Biso	1	C
H34	1	0.19751	0.45474	0.5	Biso	1	H
H35	1	0.19751	0.54523	0.5	Biso	1	H
H36	1	0.90011	0.4548	0.5	Biso	1	H
H37	1	0.95672	0.37347	0.5	Biso	1	H
H38	1	0.14008	0.37283	0.5	Biso	1	H
H39	1	0.90011	0.54517	0.5	Biso	1	H
H40	1	0.14007	0.62715	0.5	Biso	1	H

Supplementary Table 18 | Coordinates for the 0.8-Å slipped AA stacking structure of the MC-COF-NiPc-E_7E_9

		x	*y*	*z*			
C1	1	0.97817	0.83935	1	Biso	1	C
C2	1	0.02183	0.83935	1	Biso	1	C
C3	1	0.0451	0.79577	1	Biso	1	C
C4	1	0.0217	0.75304	1	Biso	1	C
C5	1	0.9783	0.75304	1	Biso	1	C
C6	1	0.9549	0.79577	1	Biso	1	C
C7	1	0.96541	0.8904	0	Biso	1	C
C8	1	0.03459	0.8904	0	Biso	1	C
H1	1	0.0788	0.79569	0	Biso	1	H
H2	1	0.9212	0.79569	1	Biso	1	H
C9	1	0.87151	0.96659	0	Biso	1	C
C10	1	0.87167	0.01865	0	Biso	1	C
C11	1	0.83535	0.04679	1	Biso	1	C
C12	1	0.79937	0.01916	1	Biso	1	C
C13	1	0.7991	0.96744	1	Biso	1	C
C14	1	0.83477	0.93919	0	Biso	1	C
C15	1	0.91432	0.95125	0	Biso	1	C
C16	1	0.91447	0.03366	0	Biso	1	C
H3	1	0.83554	0.08697	1	Biso	1	H
H4	1	0.8344	0.89902	0	Biso	1	H
C17	1	0.02184	0.14553	0	Biso	1	C
C18	1	0.97816	0.14553	0	Biso	1	C
C19	1	0.95485	0.18906	1	Biso	1	C
C20	1	0.9783	0.23174	1	Biso	1	C
C21	1	0.0217	0.23174	1	Biso	1	C
C22	1	0.04515	0.18906	1	Biso	1	C
C23	1	0.03456	0.09454	0	Biso	1	C
C24	1	0.96544	0.09454	0	Biso	1	C
H5	1	0.92116	0.18911	1	Biso	1	H
H6	1	0.07884	0.18911	0	Biso	1	H
C25	1	0.12849	0.96659	0	Biso	1	C
C26	1	0.12833	0.01865	0	Biso	1	C
C27	1	0.16465	0.04679	0	Biso	1	C
C28	1	0.20063	0.01916	0	Biso	1	C
C29	1	0.2009	0.96744	0	Biso	1	C
C30	1	0.16523	0.93919	0	Biso	1	C
C31	1	0.08568	0.95125	0	Biso	1	C
C32	1	0.08553	0.03366	0	Biso	1	C
H7	1	0.16446	0.08697	1	Biso	1	H

H8	1	0.1656	0.89902	1	Biso	1	H
N1	1	0.92617	0.90448	0	Biso	1	N
N2	1	0.92623	0.08044	0	Biso	1	N
N3	1	0.07377	0.08044	1	Biso	1	N
N4	1	0.07383	0.90448	0	Biso	1	N
N5	1	1	0.92038	0	Biso	1	N
N6	1	0.93956	0.9924	0	Biso	1	N
N7	1	1	0.06445	0	Biso	1	N
N8	1	0.06044	0.9924	0	Biso	1	N
O1	1	0.03535	0.7049	0	Biso	1	O
O2	1	0.96465	0.7049	1	Biso	1	O
O3	1	0.75908	0.03579	1	Biso	1	O
O4	1	0.7586	0.95151	1	Biso	1	O
O5	1	0.96466	0.27992	1	Biso	1	O
O6	1	0.03534	0.27992	1	Biso	1	O
O7	1	0.24092	0.03579	0	Biso	1	O
O8	1	0.2414	0.95151	0	Biso	1	O
B1	1	0	0.30865	1	Biso	1	B
B2	1	0.26528	0.99384	0	Biso	1	B
B3	1	0	0.67614	0	Biso	1	B
B4	1	0.73472	0.99384	1	Biso	1	B
Ni1	1	1	0.99237	0	Biso	1	Ni
C33	1	0.33254	0.03883	0	Biso	1	C
C34	1	0.37525	0.03964	0	Biso	1	C
C35	1	0.39712	0.99488	0	Biso	1	C
C36	1	0.37564	0.94986	0	Biso	1	C
C37	1	0.33291	0.9502	0	Biso	1	C
C38	1	0.31116	0.99436	0	Biso	1	C
H9	1	0.31366	0.07215	0	Biso	1	H
H10	1	0.39203	0.07456	0	Biso	1	H
H11	1	0.39269	0.91511	0	Biso	1	H
H12	1	0.3142	0.91674	0	Biso	1	H
C39	1	0.44117	0.99503	0	Biso	1	C
C40	1	0.47877	0.99504	0	Biso	1	C
C41	1	0.52123	0.99504	0	Biso	1	C
C42	1	0.55883	0.99503	0	Biso	1	C
C43	1	0.60288	0.99488	0	Biso	1	C
C44	1	0.62436	0.94986	0	Biso	1	C
C45	1	0.62475	0.03964	0	Biso	1	C
C46	1	0.66709	0.9502	1	Biso	1	C
H13	1	0.60731	0.91511	1	Biso	1	H
C47	1	0.66746	0.03883	1	Biso	1	C
H14	1	0.60797	0.07456	1	Biso	1	H

C48	1	0.68884	0.99436	1	Biso	1	C
H15	1	0.6858	0.91674	1	Biso	1	H
H16	1	0.68634	0.07215	1	Biso	1	H
C49	1	0	0.36336	0	Biso	1	C
C50	1	0.96273	0.3886	0	Biso	1	C
C51	1	0.96181	0.44009	0	Biso	1	C
C52	1	0	0.46622	0	Biso	1	C
C53	1	0.03819	0.44009	0	Biso	1	C
C54	1	0.03727	0.3886	0	Biso	1	C
C55	1	0.92396	0.46735	0	Biso	1	C
C56	1	0	0.51852	0	Biso	1	C
C57	1	0.96183	0.54467	0	Biso	1	C
C58	1	0.92398	0.51742	0	Biso	1	C
C59	1	0.96277	0.59615	0	Biso	1	C
H17	1	0.93507	0.61914	1	Biso	1	H
C60	1	0	0.62142	0	Biso	1	C
C61	1	0.03723	0.59615	0	Biso	1	C
C62	1	0.03817	0.54467	0	Biso	1	C
C63	1	0.07602	0.51742	0	Biso	1	C
C64	1	0.07604	0.46735	0	Biso	1	C
H18	1	0.10531	0.44718	0	Biso	1	H
H19	1	0.10527	0.53763	1	Biso	1	H
H20	1	0.89469	0.44718	1	Biso	1	H
H21	1	0.93507	0.36555	0	Biso	1	H
H22	1	0.06493	0.36555	0	Biso	1	H
H23	1	0.89473	0.53763	1	Biso	1	H
H24	1	0.06493	0.61914	1	Biso	1	H
C65	1	0.97816	0.89095	0.5	Biso	1	C
C66	1	0.02184	0.89095	0.5	Biso	1	C
C67	1	0.04515	0.84742	0.5	Biso	1	C
C68	1	0.0217	0.80474	0.5	Biso	1	C
C69	1	0.9783	0.80474	0.5	Biso	1	C
C70	1	0.95485	0.84742	0.5	Biso	1	C
C71	1	0.96544	0.94194	0.5	Biso	1	C
C72	1	0.03456	0.94194	0.5	Biso	1	C
H25	1	0.07884	0.84737	0.5	Biso	1	H
H26	1	0.92116	0.84737	0.5	Biso	1	H
C73	1	0.87167	0.01782	0.5	Biso	1	C
C74	1	0.87151	0.06989	0.5	Biso	1	C
C75	1	0.83477	0.09728	0.5	Biso	1	C
C76	1	0.7991	0.06902	0.5	Biso	1	C
C77	1	0.79937	0.0173	0.5	Biso	1	C
C78	1	0.83535	0.98968	0.5	Biso	1	C

C79	1	0.91447	0.00281	0.5	Biso	1	C
C80	1	0.91432	0.08522	0.5	Biso	1	C
H27	1	0.83439	0.13745	0.5	Biso	1	H
H28	1	0.83555	0.9495	0.5	Biso	1	H
C81	1	0.02183	0.19713	0.5	Biso	1	C
C82	1	0.97817	0.19713	0.5	Biso	1	C
C83	1	0.9549	0.24071	0.5	Biso	1	C
C84	1	0.9783	0.28344	0.5	Biso	1	C
C85	1	0.0217	0.28344	0.5	Biso	1	C
C86	1	0.0451	0.24071	0.5	Biso	1	C
C87	1	0.03459	0.14607	0.5	Biso	1	C
C88	1	0.96541	0.14607	0.5	Biso	1	C
H29	1	0.9212	0.24079	0.5	Biso	1	H
H30	1	0.0788	0.24079	0.5	Biso	1	H
C89	1	0.12833	0.01782	0.5	Biso	1	C
C90	1	0.12849	0.06989	0.5	Biso	1	C
C91	1	0.16523	0.09728	0.5	Biso	1	C
C92	1	0.2009	0.06902	0.5	Biso	1	C
C93	1	0.20063	0.0173	0.5	Biso	1	C
C94	1	0.16465	0.98968	0.5	Biso	1	C
C95	1	0.08553	0.00281	0.5	Biso	1	C
C96	1	0.08568	0.08522	0.5	Biso	1	C
H31	1	0.16561	0.13745	0.5	Biso	1	H
H32	1	0.16445	0.9495	0.5	Biso	1	H
N9	1	0.92623	0.95603	0.5	Biso	1	N
N10	1	0.92617	0.132	0.5	Biso	1	N
N11	1	0.07383	0.132	0.5	Biso	1	N
N12	1	0.07377	0.95603	0.5	Biso	1	N
N13	1	1	0.97203	0.5	Biso	1	N
N14	1	0.93956	0.04408	0.5	Biso	1	N
N15	1	1	0.1161	0.5	Biso	1	N
N16	1	0.06044	0.04408	0.5	Biso	1	N
O9	1	0.03535	0.75656	0.5	Biso	1	O
O10	1	0.96466	0.75656	0.5	Biso	1	O
O11	1	0.75859	0.08495	0.5	Biso	1	O
O12	1	0.75909	0.00067	0.5	Biso	1	O
O13	1	0.96465	0.33158	0.5	Biso	1	O
O14	1	0.03535	0.33158	0.5	Biso	1	O
O15	1	0.24141	0.08495	0.5	Biso	1	O
O16	1	0.24091	0.00067	0.5	Biso	1	O
B5	1	0	0.36034	0.5	Biso	1	B
B6	1	0.26528	0.04262	0.5	Biso	1	B
B7	1	0	0.72783	0.5	Biso	1	B

B8	1	0.73472	0.04262	0.5	Biso	1	B
Ni2	1	1	0.04411	0.5	Biso	1	Ni
C97	1	0.3329	0.08627	0.5	Biso	1	C
C98	1	0.37562	0.08663	0.5	Biso	1	C
C99	1	0.39712	0.04163	0.5	Biso	1	C
C100	1	0.37527	0.99686	0.5	Biso	1	C
C101	1	0.33255	0.99764	0.5	Biso	1	C
C102	1	0.31116	0.0421	0.5	Biso	1	C
H33	1	0.31417	0.11971	0.5	Biso	1	H
H34	1	0.39266	0.12139	0.5	Biso	1	H
H35	1	0.39206	0.96194	0.5	Biso	1	H
H36	1	0.31369	0.96431	0.5	Biso	1	H
C103	1	0.44117	0.04149	0.5	Biso	1	C
C104	1	0.47877	0.04148	0.5	Biso	1	C
C105	1	0.52123	0.04148	0.5	Biso	1	C
C106	1	0.55883	0.04149	0.5	Biso	1	C
C107	1	0.60288	0.04163	0.5	Biso	1	C
C108	1	0.62473	0.99686	0.5	Biso	1	C
C109	1	0.62438	0.08663	0.5	Biso	1	C
C110	1	0.66745	0.99764	0.5	Biso	1	C
H37	1	0.60794	0.96194	0.5	Biso	1	H
C111	1	0.6671	0.08627	0.5	Biso	1	C
H38	1	0.60734	0.12139	0.5	Biso	1	H
C112	1	0.68884	0.0421	0.5	Biso	1	C
H39	1	0.68631	0.96431	0.5	Biso	1	H
H40	1	0.68583	0.11971	0.5	Biso	1	H
C113	1	0	0.41506	0.5	Biso	1	C
C114	1	0.96277	0.44033	0.5	Biso	1	C
C115	1	0.96183	0.49181	0.5	Biso	1	C
C116	1	0	0.51796	0.5	Biso	1	C
C117	1	0.03817	0.49181	0.5	Biso	1	C
C118	1	0.03723	0.44033	0.5	Biso	1	C
C119	1	0.92398	0.51906	0.5	Biso	1	C
C120	1	0	0.57027	0.5	Biso	1	C
C121	1	0.96181	0.5964	0.5	Biso	1	C
C122	1	0.92396	0.56913	0.5	Biso	1	C
C123	1	0.96273	0.64788	0.5	Biso	1	C
H41	1	0.93507	0.67093	0.5	Biso	1	H
C124	1	0	0.67312	0.5	Biso	1	C
C125	1	0.03727	0.64788	0.5	Biso	1	C
C126	1	0.03819	0.5964	0.5	Biso	1	C
C127	1	0.07604	0.56913	0.5	Biso	1	C
C128	1	0.07602	0.51906	0.5	Biso	1	C

H42	1	0.10527	0.49885	0.5	Biso	1	H
H43	1	0.10531	0.5893	0.5	Biso	1	H
H44	1	0.89473	0.49885	0.5	Biso	1	H
H45	1	0.93507	0.41735	0.5	Biso	1	H
H46	1	0.06493	0.41735	0.5	Biso	1	H
H47	1	0.89469	0.5893	0.5	Biso	1	H
H48	1	0.06493	0.67093	0.5	Biso	1	H

Supplementary Note 1

Mesitylene, dioxane, and anhydrous acetone (99.5%) were purchased from Wako Chemicals. 2,3,6,7,10,11-Hexahydroxytriphenylene (TP), 2,6-dibromonaphthalene, 1,4-phenylenediboronic acid, 4,4'-biphenyldiboronic acid, 2,6-bis(4,4,5,5-tetramethyl-1,3,2-dioxaborolan-2-yl)benzo[1,2-b:4,5-b']dithiophene, 4,7-bis(4,4,5,5-tetramethyl-1,3,2-dioxaborolan-2-yl)-2,1,3-benzothiadiazole, and 2,7-bis(4,4,5,5-tetramethyl-1,3,2-dioxaborolan-2-yl)pyrene were purchased from TCI. Thieno[3,2-b]thiophene, 1,2-bis(4-(4,4,5,5-tetramethyl-1,3,2-dioxaborolan-2-yl)phenyl)acetylene, and 4-ethynylphenylboronic acid pinacol ester were purchased from Sigma-Aldrich. Thieno[3, 2-b]thiophene-2,5-diyl diboronic acid, 1,4-benzothiadiazole diboronic acid, 2,6-naphthalenediyl diboronic acid, benzo[1,2-b:4,5-b']dithiophene-2,6-diyl diboronic acid, and NiPc were synthesized according to literature procedures[1,2].

Supplemetary References

1. Meng, W. *et al*. Empirical and theoretical insights into the structural features and host-guest chemistry of M_8L_4 tube architectures. *J. Am. Chem. Soc.* **136**, 3972-3980 (2014).
2. Duhovic, S. & Dinca, M. Synthesis and electrical properties of covalent organic frameworks with heavy chalcogens. *Chem. Mater.* **27**, 5487-5490 (2015).
